# Supplementary material for: Enantioselective Heck/Tsuji−Trost reaction of flexible vinylic halides with 1,3-dienes
Source: Nat Commun. 2025 Jan 22;16:930. doi: 10.1038/s41467-025-56142-z (PMC11754474; doi:10.1038/s41467-025-56142-z)
Supplement: Supplementary file 1 — Supplementary Information [file 41467_2025_56142_MOESM1_ESM.pdf]

Supplementary Information

Enantioselective Heck/Tsuji−Trost Reaction of Flexible Vinylic Halides with 1,3-Dienes

Li-Zhi Zhang,[1,‡] Pei-Chao Zhang,[2,‡] Qian Wang,[3] Min Zhou*[1] and Junliang Zhang*[3,4]

[1]School of Ethnic Medicine, Yunnan Minzu University, Kunming 650031, P.R. China

[2]The Center for Basic Research and Innovation of Medicine and Pharmacy (MOE), School of Pharmacy, Second Military Medical University (Naval Medical University), Shanghai 200433, P. R. China

[3]College of Chemistry and Life Science, Advanced Institute of Materials Science, Changchun University of Technology, Changchun 130012, P. R. China

[4]Department of Chemistry, Fudan University, 2005 Songhu Road, Shanghai 200438, P. R. China

Contents

1. General information S2

2. Optimization of the reaction conditions. S3

3. X-ray of the product 3aa. S7

4. Non-linear effect S7

5. Synthesis of the Xu-Phos and starting materials. S8

6. General procedure for asymmetric Heck/Tsuji-Trost reaction of flexible vinylic halides with1,3-dienes. S10

7. Gram-scale synthesis. S42

8. Synthetic applications of the products. S43

9. NMR Spectra S48

10. References S122

1. General information

Unless otherwise noted, all reactions were carried out in standard Schlenk techniques with magnetic stirring bar under air. Materials obtained from commercial suppliers were used directly without further purification. **^{1}H NMR** spectra were recorded in $CDCl_3$ with Bruker spectrometers at 400 (100 MHz) or 500 (125 MHz). Chemical shifts are reported in ppm with tetramethylsilane (TMS: 0 ppm) with the solvent resonance as the internal standard. Data are reported as follows: chemical shift, multiplicity (s = singlet, d = doublet, t = triplet, q = quartet, m = multiplet), coupling constants (Hz), and integration. **^{13}C NMR** spectra were recorded on Bruker 400 (100 MHz) and 500 (125 MHz) spectrometers in $CDCl_3$ with complete proton decoupling. Chemical shifts are reported in ppm with the deuterium solvent as the internal standard ($CDCl_3$: 77.0 ppm). The $[\alpha]^{D}$ were measured on a PolAAr 3005 High Accuracy Polarimeter. The *ee* was recorded using LC-2030C 3D from Shimadzu Company. Flash column chromatography was performed over silica gel (10-40 mesh).

The starting materials **Xu-Phos**[1], vinylic halides **1**[2] and cyclic 1,3-dienes **2**[3] were synthesized according to published procedures. The spectral data of the substrates were consisted with that reported in the literature. The enantionmeric excesses of the products were determined by chiral stationary phase HPLC using Chiralpak IA, IC, ID, IE and IF columns.

Note: The prepared cyclic 1,3-dienes should be stored at low temperature and protected from light, and used as soon as possible. If the storage time of cyclic 1,3-diene is too long, it should be purified by flash column chromatography on silica gel to remove excess water and impurities before use. The yields reported represent single runs and have not been reproduced in this work.

2. Optimization of the reaction conditions.

2.1. Optimization of Sadphos

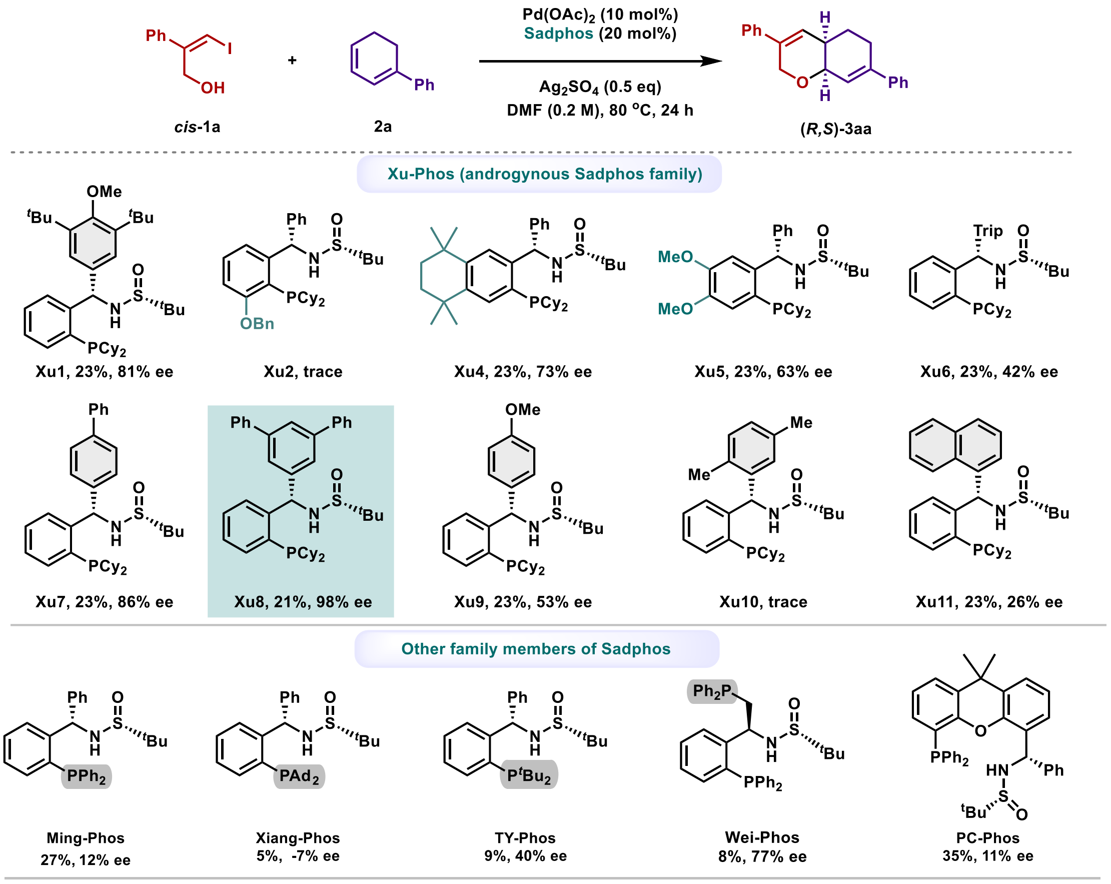

Supplementary Fig. 1 Optimization of Sadphos.

Unless otherwise noted, all reactions were carried out with **1a** (0.1 mmol), 0.4 mmol of **2a** (0.4 mmol), $Pd(OAc)_2$ (10 mol%), Xu-Phos (20 mol%) and Ag_2SO_4 (0.05 mmol), in 0.5 mL DMF at 80 °C for 24 h; yield and conversion were determined by GC analysis using anisole as a standard, e.e. were determined by HPLC analysis. Trip: 2-triptycene.

2.2. Optimization of solvent

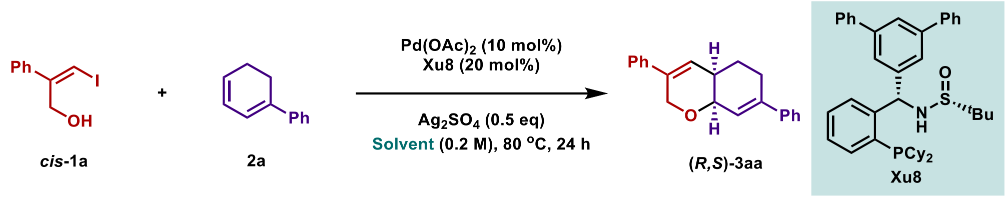

Entry	Solvent	Yield%	Ee%
1	DMF	21	98
**2**	**DMAc**	**28**	**95**
3	NMP	19	94
4	DMSO	36	35
5	Et_2O	31	36
6	CF_3Ph	24	35
7	DCE	trace	-
8	THF	43	27
9	DME	38	28

Supplementary Fig. 2 Optimization of solvent.

Unless otherwise noted, all reactions were carried out with **1a** (0.1 mmol), 0.4 mmol of **2a** (0.4 mmol), $Pd(OAc)_2$ (10 mol%), Xu8 (20 mol%) and Ag_2SO_4 (0.05 mmol), in 0.5 mL solvent at 80 °C for 24 h; yield and conversion were determined by GC analysis using anisole as a standard, e.e. were determined by HPLC analysis. DMAc: Dimethylacetamide; NMP: N-methyl pyrrolidone.

2.3. Optimization of silver salt

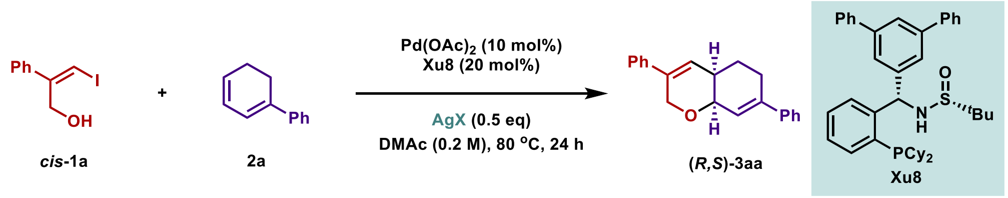

Entry	Silver salt	Yield%	Ee%
**1**	**Ag_2SO_4**	**29**	**96**
2	Ag_2CO_3	Trace	96
3	AgF	24	94
4	Ag_2O	19	96
5	$AgN(CF_3SO_2)_2$	36	55
6	AgOTs	35	51
7	$AgPF_6$	34	48
8	CF_3CO_2Ag	41	53
9	CH_3CO_2Ag	NR	--
10	CH_3SO_3Ag	23	52

Supplementary Fig. 3 Optimization of silver salt.

Unless otherwise noted, all reactions were carried out with **1a** (0.1 mmol), 0.4 mmol of **2a** (0.4 mmol), $Pd(OAc)_2$ (10 mol%), Xu8 (20 mol%) and silver salt (0.05 mmol), in 0.5 mL DMAc at 80 °C for 24 h; yield and conversion were determined by GC analysis using anisole as a standard, e.e. were determined by HPLC analysis.

2.4. Optimization of temperature

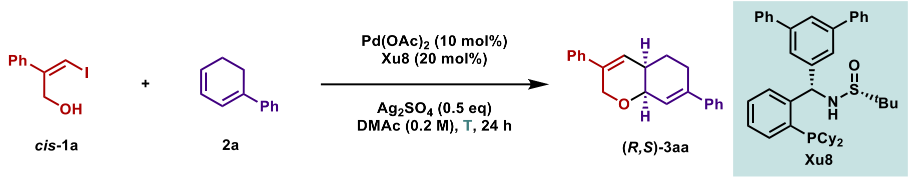

Entry	T/⁰C	Yield%	Ee%
1	50	NR	--
2	60	30	99
**3**	**70**	**32**	**97**
4	80	28	96
5	90	21	90
6	100	18	87
7	110	17	84
8	120	13	68

Supplementary Fig. 4 Optimization of temperature.

Unless otherwise noted, all reactions were carried out with **1a** (0.1 mmol), 0.4 mmol of **2a** (0.4 mmol), $Pd(OAc)_2$ (10 mol%), **Xu8** (20 mol%) and Ag_2SO_4 (0.05 mmol), in 0.5 mL DMAc at 50-120 ℃ for 24 h; yield and conversion were determined by GC analysis using anisole as a standard, e.e. were determined by HPLC analysis.

2.5. Optimization of Pd presursors

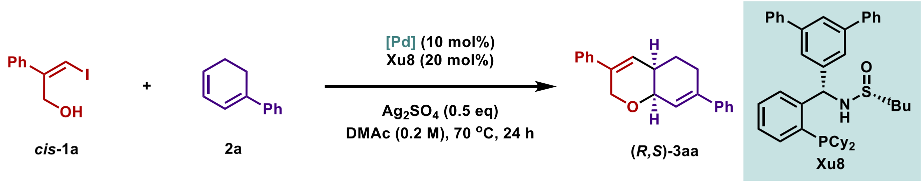

Entry	[Pd]	Yield%	Ee%
1	$Pd(PPh_3)_4$	NR	--
2	$Pd_2(dba)_3$	33	95
3	$Pd_2(dba)_3{\cdot}CHCl_3$	35	97
4	Pd $(dba)_2$	29	95
5	Pd $(TFA)_2$	41	98
6	$[Pd(allyl)Cl]_2$	30	95
7	$PdCl_2(PPh_3)_2$	20	95
8	$Pd(PhCN)_2Cl_2$	31	96
9	$PdBF_4(CH_3CN)_4$	48	98
**10**	**$Pd(CO_2{}^tBu)_2$**	**62**	**99**

Supplementary Fig. 5 Optimization of Pd presursors.

Unless otherwise noted, all reactions were carried out with **1a** (0.1 mmol), 0.4 mmol of **2a** (0.4 mmol), [Pd] (10 mol%), **Xu8** (20 mol%) and Ag_2SO_4 (0.05 mmol), in 0.5 mL DMAc at 70 ℃ for 24 h; yield and conversion were determined by GC analysis using anisole as a standard, e.e. were determined by HPLC analysis.

On the one hand, we supposed tha $Pd(OAc)_2$ as a soluble palladium salt in organic solvents, is widely utilized as a catalyst in a broad array of organic synthesis reactions. The $Pd(CO_2{}^tBu)_2$ has better fat-solubility to accelerate the initiation Heck step of this reaction. On the other hand, the pre-catalyst (Pd^{II}) must be activated in the presence of ligands (Sadphos), which results in the

release of Pd(0). The use of a bulky tert-butyl ester as a ligand is advantageous for the reduction elimination processes, and it facilitates the in situ generation of Pd(0) to accelerate the initiation Heck step of this reaction.

2.6. Screening of chiral ligands

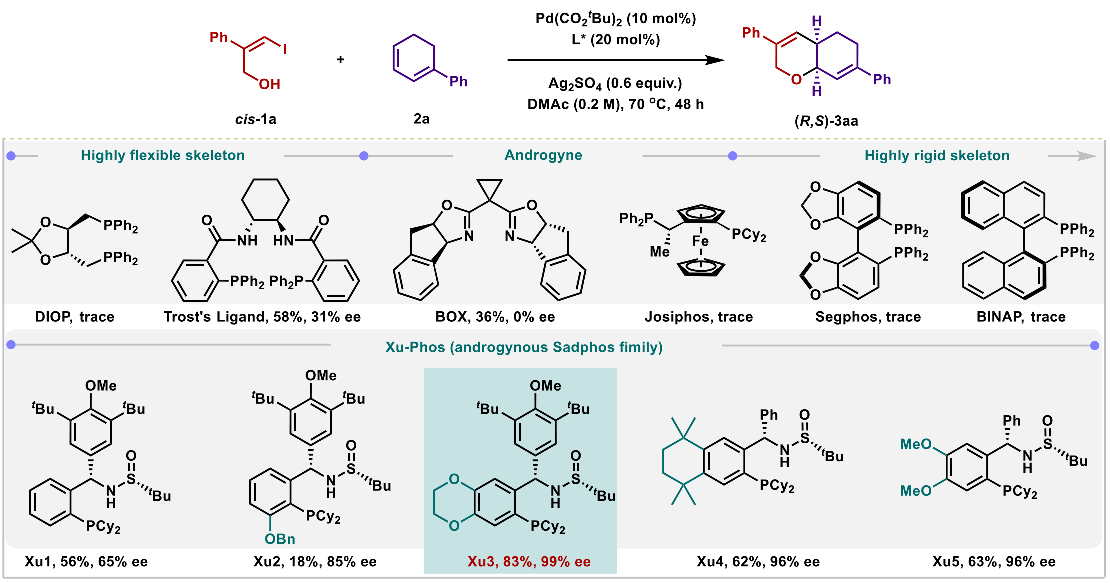

Supplementary Fig. 6 Screening of chiral ligands.

Unless otherwise noted, all reactions were carried out with **1a** (0.1 mmol), 0.4 mmol of **2a** (0.4 mmol), Pd(CO$_2{}^t$Bu)$_2$ (10 mol%), L* (20 mol%) and Ag_2SO_4 (0.06 mmol), in 0.5 mL DMAc at 70 °C for 48 h; yield and conversion were determined by GC analysis using anisole as a standard, e.e. were determined by HPLC analysis.

2.7. Optimization of Pd/ligand loadings

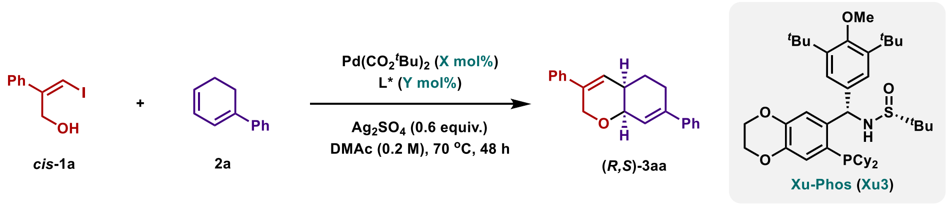

Entry	Pd:L (X:Y) (%)	GC Yield (%)	Ee (%)
1	10:10	71	94
2	10:12	80	97
3	10:20	83	99
4	5:10	54	97
5	6:12	60	96

Supplementary Fig. 7 Optimization of Pd/ligand loadings.

Unless otherwise noted, all reactions were carried out with **1a** (0.1 mmol), 0.4 mmol of **2a** (0.4 mmol), Pd(CO$_2{}^t$Bu)$_2$ (X mol%), **Xu3** (Y mol%) and Ag_2SO_4 (0.06 mmol), in 0.5 mL DMAc at 70 °C for 48 h; yield and conversion were determined by GC analysis using anisole as a standard, e.e. were determined by HPLC analysis.

2.8. The limitations of the substrate scope

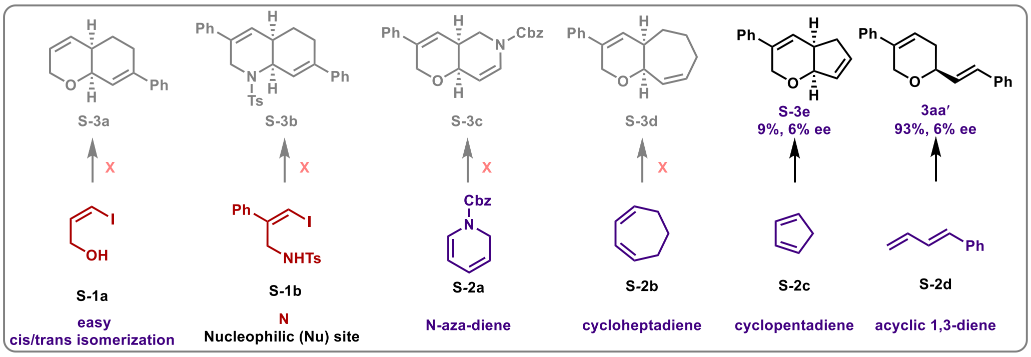

Supplementary Fig. 8 The limitations of the substrate scope.

3. X-ray of the product 3aa.

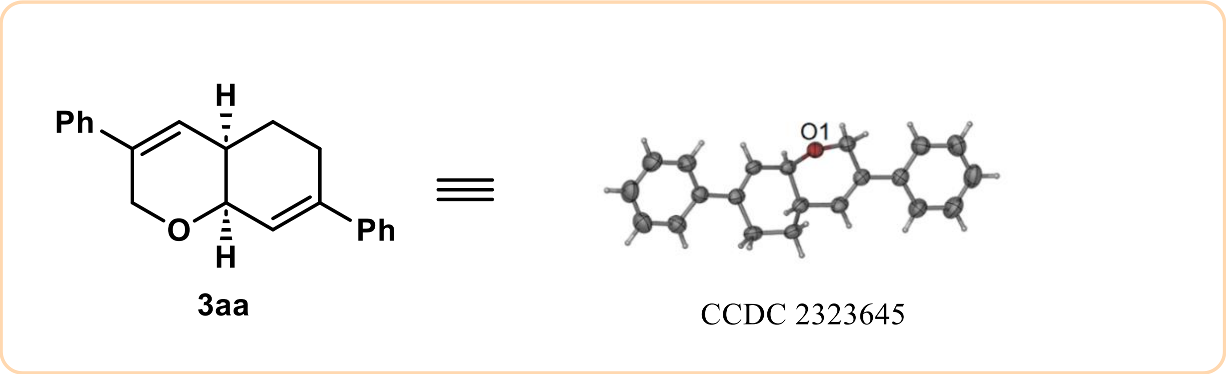

Supplementary Fig. 9 X-ray of the product 3aa.

4. Non-linear effect

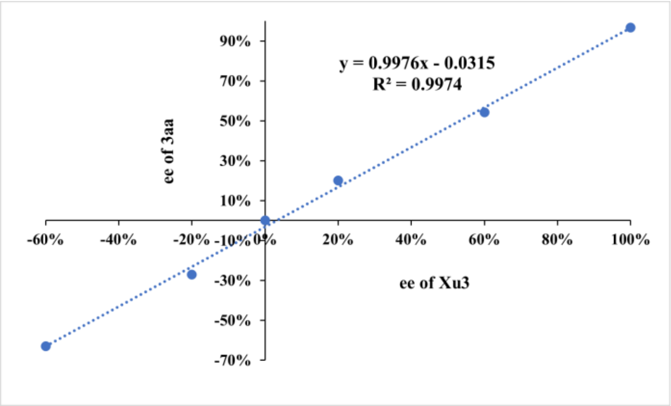

Supplementary Fig. 10 Non-linear effect.

The linear relationship between the ee of the **Xu3** and those of product **3aa**, which reveal that the enantioselectivity determining step might involve a single chiral sulfinamide phosphine ligand and one palladium species.

5. Synthesis of the Xu-Phos and starting materials.

5.1. Sythesis of the Xu-Phos[1].

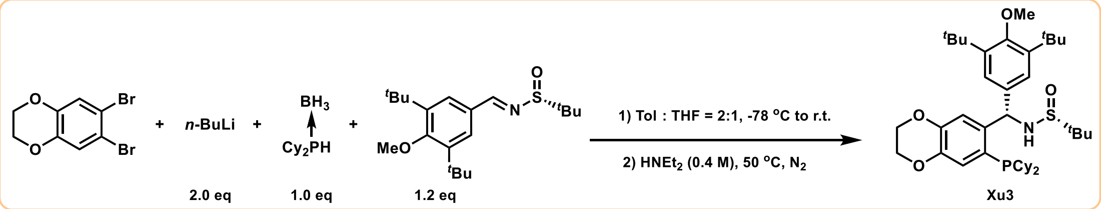

To a stirred solution of dicyclohexylphosphine borane ($Cy_2PH•BH_3$, 1.0 equiv, 8 mmol, CAS: 108756-88-3) in anhydrous Tol (16 mL) and THF (8 mL) under N_2 atmosphere was added *n*-BuLi (1.0 equiv, 8 mmol, 1.6 M in hexane, 5 mL) dropwise at -78 °C. The mixture maintained this temperature for 1 h. Then, the mixed solvents Tol : THF (6 mL:3 mL) dissolving the dibromobenzene (8 mmol, CAS: 25812-80-0) was added dropwise followed by *n*-BuLi (1.0 equiv, 8 mmol, 1.6 M, 5 mL) . After 10 minutes at -78 °C, (*E*, *Rs*)-sulfinyl imine (1.2 equiv, 9.6 mmol, CAS: 2241598-36-5) was added and the reaction mixture was slowly warmed to room temperature for 10 h. After reaction was completed, the reaction mixture was quenched by the addition of saturated aqueous NH_4Cl solution (10 mL) and extracted by EA (20 mL×3). The combined organic layers were washed by brine, dried over Na_2SO_4 and concentrated to dryness.

The crude product was dealed with $HNEt_2$ (20 mL) under N_2 atmosphere and the resulting solution was stirred at 50 °C for more 8 h. After the reaction was complete (monitored by TLC), solvent was removed under reduced pressure. The residue was purified by flash column chromatography on silica gel (PE: EA = 6:1) to afford the desired product **Xu3** (2.95g, 54% yield) as a white solid.

(*R*)-*N*-((*S*)-(3,5-di-*tert*-butyl-4-methoxyphenyl)(7-(dicyclohexylphosphaneyl)-2,3-dihydrobenzo[*b*][1,4]dioxin-6-yl)methyl)-2-methylpropane-2-sulfinamide (Xu3)

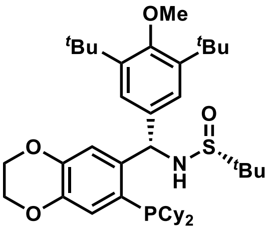

^{1}H NMR (400 MHz, $CDCl_3$) δ 7.26 (s, 2H), 7.14 (d, *J* = 3.3 Hz, 1H), 6.96 (s, 1H), 6.63 (d, *J* = 8.6 Hz, 1H), 4.32 (s, 4H), 3.68 (br s, 1H), 3.65 (s, 3H), 1.94 (m, 1H), 1.85 – 1.60 (overlapped, 10H), 1.58 – 1.42 (m, 4H), 1.42 – 1.35 (overlapped, 16H), 1.31 – 1.24 (overlapped, 10H), 1.23 – 1.13 (overlapped, 4H), 1.11 – 0.93 (overlapped, 3H), 0.57 (m, 1H);

^{13}C NMR (100 MHz, $CDCl_3$) δ 158.6, 144.1, 143.2, 142.6 (d, *J* = 23.7 Hz), 142.1, 136.9, 127.3 (d, *J* = 21.5 Hz), 126.9, 126.0, 121.1, 116.5 (d, *J* = 6.2 Hz), 64.5, 64.4, 64.1, 59.0 (d, *J* = 29.2 Hz), 55.8, 35.8, 35.7 (d, *J* = 13.1 Hz), 35.1 (d, *J* = 13.3 Hz), 32.1, 30.7 (dd, *J* = 18.0, 12.2 Hz), 29.2 (t, *J* = 9.2 Hz), 27.2 (m), 26.4 (d, *J* = 7.2 Hz), 22.8;

^{31}P NMR (130 MHz, $CDCl_3$) δ -18.4;

HRESIMS (positive ion mode) *m/z* 684.4214 $[M + H]^+$ (calcd. for $C_{40}H_{63}NO_4PS$, 684.4210);

$[\alpha]_D^{20}$ = 0.9 (*c* = 0.14, acetone).

5.2. Sythesis of the Vinylic Halides[2].

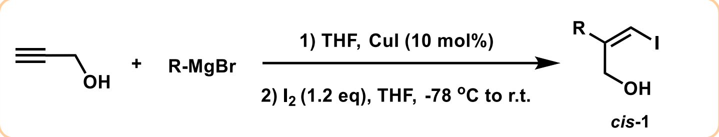

To a stirred solution of propargyl alcohol (50 mmol, CAS: 107-19-7) in anhydrous THF (100 mL) under N_2 atmosphere was added CuI (10 mol%, 5 mmol, CAS: 7681-65-4) and the mixture was cooled to -78 °C. Freshly prepared Grignard reagents (2.5 eq, 125 mmol) in THF (100 mL) was added using constant pressure funnel maintain the temperature below -60 °C for 1h. Then, the mixture was allowed to warm up to room temperture and vigorously stirred overnight. The reaction was cooled again to -78 °C and treated with I_2 (55 mmol, 1.1 equiv.) in anhydrous THF (100 mL). After warming up to room temperature and stirring at rt for additional 1h, the reaction mixture was kept in refrigerator at about 0-3 °C over 3 hours. The mixture was cooled to 0 °C and was quenched with saturated aqueous NH_4Cl (50 mL). The two phase mixture was poured through a separatory funnel and cambined organic layers were washed extracted with saturated aqueous $Na_2S_2O_3$ (2 × 50 mL) and saturated aqueous NaCl (100 mL), and dried over Na_2SO_4. It was purified by column chromatography to give **1** (42-85 % yield).

5.3. Sythesis of the cyclic 1,3-dienes[3].

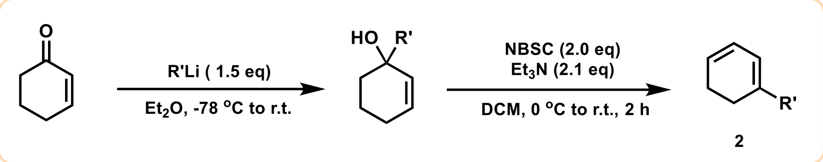

To a solution of 2-cyclohexen-1-one (10 mmol, 0.97 mL, CAS: 930-68-7, *Bide*) in 20 mL dry Et_2O at -78 °C for 10 min under N_2, was added lithium reagent (15 mmol, 1.5 eq, 1 M in Et_2O), the mixture was stirred at - 78 °C for 10 min, and allowed to warm to room temperature for another 30 min. When completed, the reaction mixture was quenched by the addition of saturated NH_4Cl solution and diluted with EtOAc. The organic layer was separated, and the aqueous layer was extracted twice with EA. The combined organic layers were dried over Na_2SO_4, filtered, concentrated, and purified by flash chromatography (PE: EA = 3:1) to isolated allyl alcohols as liquid (77-93 % yield).

To a solution of allyl alcohols (5 mmol) and Et_3N (10.0 mmol, 2.0 eq) in 20 mL dry DCM at 0 °C for 10 min under N_2, to the mixture was added 2-BSC (5.5 mmol, 1.1 eq, 2-nitrobenzenesulfenyl chloride, CAS: 7669-54-7, *Bide*) (When R= alkyl, allyl, vinyl; 2,4-NBSC instead of 2-BSC, 2,4-dinitrobenzenesulfenyl chloride, CAS: 528-76-7, *TCI*) at 0 °C for 10 min, and allowed to warm to room temperature for another 2 h. When completed, *n*-Hexane was added to the reaction mixture. The precipitate was filtered out (celite), and the solution was washed with H_2O and brine and dried over Na_2SO_4, filtered, concentrated, and purified by flash chromatography (*n*-Hexane) to isolated cyclic 1,3-dienes **2** as liquid or solid (57-83 % yield). (Note: The prepared 1,3-diene should be stored at low temperature and protected from light.)

6. General procedure for asymmetric Heck/Tsuji-Trost reaction of flexible vinylic halides with1,3-dienes.

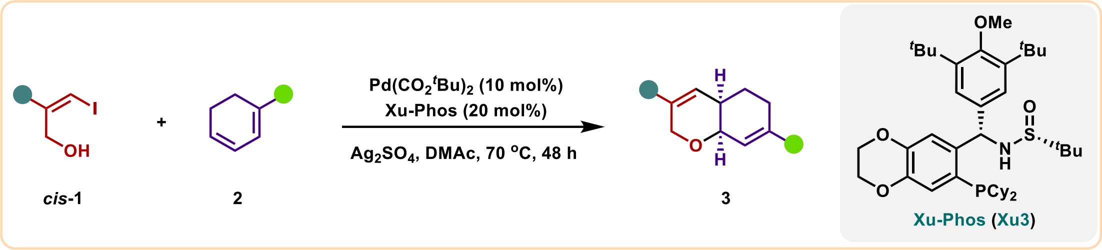

General procedure:

To a sealed tube was added Palladium pivalate (10 mol%, CAS: 106224-36-6, *Bide*) and **Xu3** (20 mol%) in 1mL dry DMAc and stirred at room temperature for 1 h under argon atmosphere. Then, **1** (0.2 mmol, 1.0 eq), **2** (0.8 mmol, 4.0 eq) and Ag_2SO_4 (0.12 mmol, 0.6 equiv) were added to the tube under argon atmosphere, and stirred at 70 °C for 48 h. After the reaction was complete (monitored by TLC), dilute with saturated salt water and EA, then extracted with EA (twice), dried over anhydrous Na_2SO_4, the solvent was removed under reduced pressure. The crude product was purified by column chromatography (*n*-Hexane/EA, 50:1 to 30:1) to give **3** as a white solid or colourless liquid (42-94 % yield).

6.1 (4a*R*,8a*S*)-3,7-diphenyl-4a,5,6,8a-tetrahydro-2*H*-chromene (3aa)

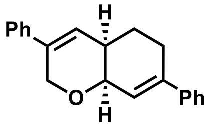

Isolated **3aa** in 83% yield, as white solid (m.p.: 157.9 °C).

^{1}H NMR (400 MHz, $CDCl_3$) δ 7.47 (d, J = 7.6 Hz, 2H), 7.41 – 7.26 (overlapped, 8H), 7.25 – 7.22 (overlapped, 2H), 4.62 (m, 2H), 4.25 (t, J = 4.5 Hz, 1H), 2.60 (dt, J = 17.4, 4.2 Hz, 1H), 2.54 – 2.42 (m, 1H), 2.35 (m, 1H), 1.96 (m, 1H), 1.80 (qd, J = 11.7, 4.9 Hz, 1H);

^{13}C NMR (100 MHz, $CDCl_3$) δ 142.6, 141.4, 138.1, 136.1, 128.5 (2C), 128.3 (2C), 127.5, 127.4, 125.5 (2C), 125.4, 124.9 (2C), 122.7, 69.6, 66.7, 34.3, 27.8, 25.2;

HRESIMS (positive ion mode) *m/z* 311.1401 $[M + Na]^+$ (calcd. for $C_{21}H_{20}ONa$, 311.1406);

$[\alpha]_D^{25}$ = -97.5 (c = 0.12 g/100mL, acetone);

HPLC conditions: Chiralpak IC column (*n*-Hexane:2-Propanol = 90:10, 0.5 ml/min, 254nm); tr (minor) = 18.5 min, tr (major) = 16.8 min; 99% *ee*.

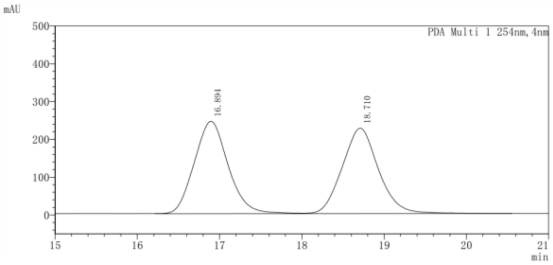

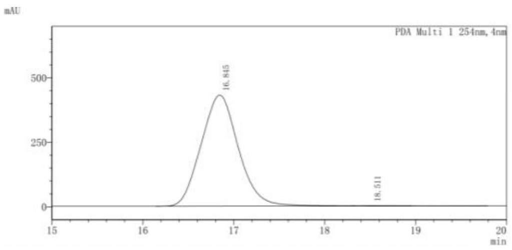

<Peak Table>
PDA Ch1 254nm

No.	Ret.Time(min)	Height	Height%	Area	Area%
1	16.894	244627	51.978	7020694	50.296
2	18.710	226012	48.022	6938149	49.704
Total		470640	100.000	13958843	100.000

<Peak Table>
PDA Ch1 254nm

No.	Ret.Time(min)	Height	Height%	Area	Area%
1	16.845	430392	99.881	12359172	99.926
2	18.511	514	0.119	9092	0.074
Total		430906	100.000	12368264	100.000

6.2 (4a*R*,8a*S*)-3-(4-chlorophenyl)-7-phenyl-4a,5,6,8a-tetrahydro-2*H*-chromene (3ab)

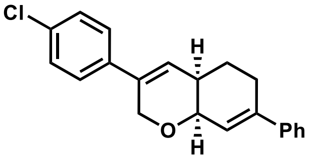

Isolated **3ab** in 88% yield, as white solid (m.p.: 179.2 °C).

^{1}H NMR (400 MHz, $CDCl_3$) δ 7.51 – 7.43 (overlapped, 2H), 7.41 – 7.26 (overlapped, 7H), 6.23 (overlapped, 2H), 4.58 (m, 2H), 4.25 (m, 1H), 2.60 (m, 1H), 2.50 (m, 1H), 2.36 (m, 1H), 1.95 (m, 1H), 1.80 (m, 1H);

^{13}C NMR (100 MHz, $CDCl_3$) δ 142.8, 141.4, 136.6, 135.2, 133.3, 128.7 (2C), 128.3 (2C), 127.6, 126.2 (2C), 126.1, 125.6 (2C), 122.6, 69.6, 66.6, 34.3, 27.8, 25.1;

HRESIMS (positive ion mode) *m/z* 345.1016 $[M + Na]^+$ (calcd. for $C_{21}H_{19}ClONa$, 345.1017);

$[\alpha]_D^{25}$ = -85.2 (*c* = 0.11 g/100mL, acetone);

HPLC conditions: Chiralpak IE column (*n*-Hexane:2-Propanol = 90:10, 0.5 ml/min, 254nm); tr (minor) = 13.7 min, tr (major) = 14.3 min; 92% *ee*.

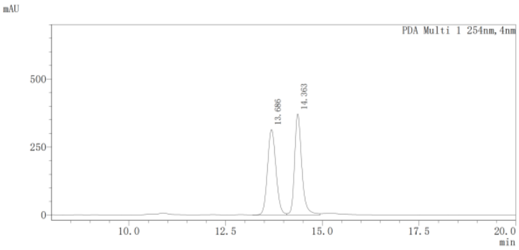

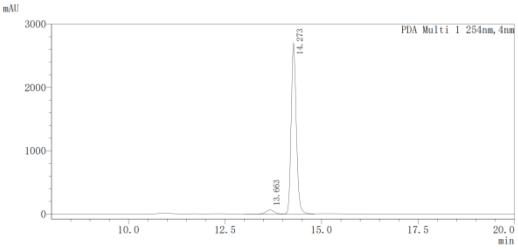

<Peak Table>
PDA Ch1 254nm

No.	Ret.Time(min)	Height	Height%	Area	Area%
1	13.686	314393	45.854	4780582	49.838
2	14.363	371253	54.146	4811620	50.162
Total		685646	100.000	9592202	100.000

<Peak Table>
PDA Ch1 254nm

No.	Ret.Time(min)	Height	Height%	Area	Area%
1	13.663	64659	2.341	1011798	3.897
2	14.273	2697412	97.659	24952025	96.103
Total		2762071	100.000	25963823	100.000

6.3 (4a*R*,8a*S*)-3-(4-fluorophenyl)-7-phenyl-4a,5,6,8a-tetrahydro-2*H*-chromene (3ac)

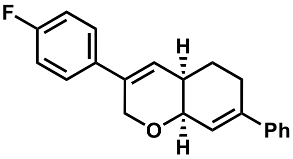

Isolated **3ac** in 93% yield, as white solid (m.p.: 140.8 °C).

^{1}H NMR (400 MHz, $CDCl_3$) δ 7.46 (m, 2H), 7.40 – 7.26 (overlapped, 5H), 7.03 (t, J = 8.7 Hz, 2H), 6.22 (d, J = 3.1 Hz, 1H), 6.15 (d, J = 4.4 Hz, 1H), 4.57 (m, 2H), 4.23 (t, J = 4.5 Hz, 1H), 2.59 (dt, J = 17.4, 4.3 Hz, 1H), 2.48 (m, 1H), 2.34 (m, 1H), 1.94 (m, 1H), 1.78 (dtd, J = 12.9, 11.0, 5.0 Hz, 1H);

^{13}C NMR (100 MHz, $CDCl_3$) δ 162.3 (d, J = 246.7 Hz), 142.7, 141.4, 135.3, 134.3 (d, J = 3.4 Hz), 128.3 (2C), 127.6, 126.6 (d, J = 7.9 Hz, 2C), 125.6 (2C), 125.5 (d, J = 1.5 Hz), 122.7, 115.39 (d, J = 21.4 Hz, 2C), 69.61, 66.8, 34.3, 27.8, 25.2;

^{19}F NMR (376 MHz, $CDCl_3$) δ -114.8;

HRESIMS (positive ion mode) *m/z* 329.1310 $[M + Na]^+$ (calcd. for $C_{21}H_{19}FONa$, 329.1312);

$[\alpha]_D^{25}$ = -51.2 (c = 0.08 g/100mL, MeOH);

HPLC conditions: Chiralpak IF column (*n*-Hexane:2-Propanol = 90:10, 0.5 ml/min, 254nm); tr (minor) = 13.6 min, tr (major) = 15.9 min; 97% *ee*.

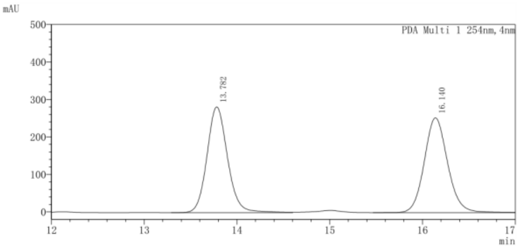

<Peak Table>
PDA Ch1 254nm

No.	Ret.Time(min)	Height	Height%	Area	Area%
1	13.782	282312	52.720	4358868	49.507
2	16.140	253183	47.280	4445758	50.493
Total		535496	100.000	8804626	100.000

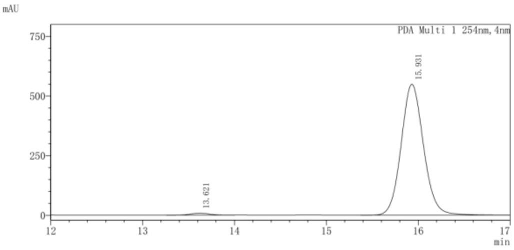

<Peak Table>
PDA Ch1 254nm

No.	Ret.Time(min)	Height	Height%	Area	Area%
1	13.621	9060	1.623	134245	1.411
2	15.931	549117	98.377	9376548	98.589
Total		558177	100.000	9510792	100.000

6.4 (4a*R*,8a*S*)-3-(4-(*tert*-butyl)phenyl)-7-phenyl-4a,5,6,8a-tetrahydro-2*H*-chromene (3ad)

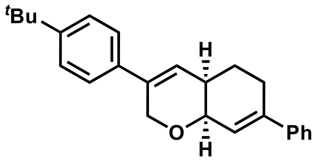

Isolated **3ad** in 88% yield, as white solid (m.p.: 186.6 °C).

^{1}H NMR (400 MHz, $CDCl_3$) δ 7.49 (d, J = 7.4 Hz, 2H), 7.44 – 7.30 (overlapped, 7H), 6.25 (d, J = 4.4 Hz, 1H), 6.22 (d, J = 4.8 Hz, 1H), 4.62 (m, 2H), 4.27 (t, J = 4.5 Hz, 1H), 2.61 (dt, J = 17.4, 4.3 Hz, 1H), 2.49 (m, 1H), 2.37 (m, 1H), 1.97 (m, 1H), 1.81 (m, 1H), 1.36 (s, 9H);

^{13}C NMR (100 MHz, $CDCl_3$) δ 150.6, 142.7, 141.5, 135.9, 135.3, 128.3 (2C), 127.6, 125.6 (2C), 125.5 (2C), 124.8, 124.6 (2C), 122.9, 69.6, 66.8, 34.5, 34.3, 31.3 (3C), 27.8, 25.3;

HRESIMS (positive ion mode) *m/z* 367.2031 $[M + Na]^+$ (calcd. for $C_{25}H_{28}ONa$, 367.2032);

$[\alpha]_D^{25}$ = -41.1 (c = 0.16 g/100mL, acetone);

HPLC conditions: Chiralpak IF column (*n*-Hexane:2-Propanol = 90:10, 0.5 ml/min, 254nm); tr (minor) = 11.6 min, tr (major) = 16.0 min; 90% *ee*.

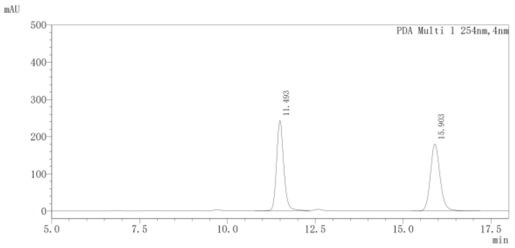

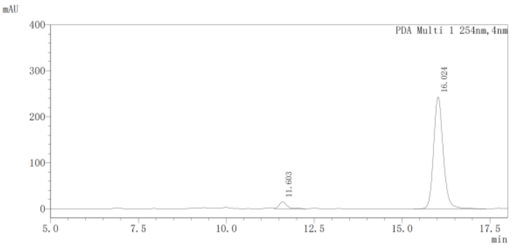

<Peak Table>
PDA Ch1 254nm

No.	Ret. Time(min)	Height	Height%	Area	Area%
1	11.493	242994	57.455	3494545	50.398
2	15.903	179939	42.545	3439384	49.602
Total		422932	100.000	6933928	100.000

<Peak Table>
PDA Ch1 254nm

No.	Ret. Time(min)	Height	Height%	Area	Area%
1	11.603	15221	5.899	232179	4.793
2	16.024	242825	94.101	4611624	95.207
Total		258046	100.000	4843803	100.000

6.5 (4a*R*,8a*S*)-3-(*m*-tolyl)-7-phenyl-4a,5,6,8a-tetrahydro-2*H*-chromene (3ae)

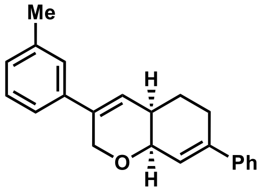

Isolated **3ae** in 94% yield, as white solid (m.p.: 114.7 °C).

^{1}H NMR (400 MHz, $CDCl_3$) δ 7.50 (m, 2H), 7.39 (m, 2H), 7.33 (m, 1H), 7.29 (m, 1H), 7.22 – 7.12 (overlapped, 3H), 6.27 (d, J = 3.4 Hz, 1H), 6.23 (d, J = 4.8 Hz, 1H), 4.63 (m, 2H), 4.27 (t, J = 4.5 Hz, 1H), 2.62 (m, 1H), 2.52 (m, 1H), 2.41 (s, 3H), 2.38 (overlapped, 1H), 1.98 (m, 1H), 1.82 (m, 1H);

^{13}C NMR (100 MHz, $CDCl_3$) δ 142.8, 141.6, 138.3, 138.2, 136.4, 128.5, 128.41 (2C), 128.38, 127.7, 125.8, 125.7 (2C), 125.4, 122.9, 122.2, 77.5, 67.0, 34.5, 27.9, 25.4, 21.6;

HRESIMS (positive ion mode) *m/z* 325.1561 [M + Na]$^+$ (calcd. for $C_{22}H_{22}ONa$, 325.1563);

$[\alpha]_D^{25}$ = -76.9 (c = 0.12 g/100mL, acetone);

HPLC conditions: Chiralpak IE column (*n*-Hexane:2-Propanol = 90:10, 0.5 ml/min, 254nm); tr (minor) = 12.3 min, tr (major) = 13.4 min; 99% *ee*.

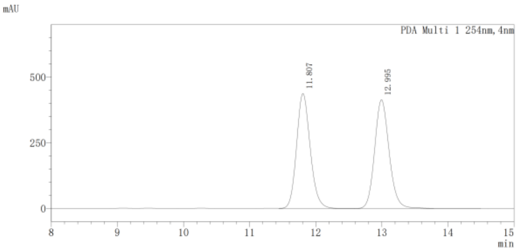

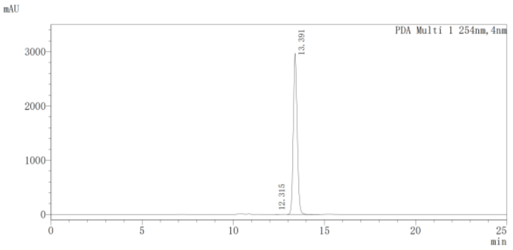

<Peak Table>
PDA Ch1 254nm

No.	Ret. Time(min)	Height	Height%	Area	Area%
1	11.807	438478	51.404	6332180	49.806
2	12.995	414532	48.596	6381544	50.194
Total		853010	100.000	12713724	100.000

<Peak Table>
PDA Ch1 254nm

No.	Ret. Time(min)	Height	Height%	Area	Area%
1	12.315	570	0.019	3391	0.008
2	13.391	2967775	99.981	43942156	99.992
Total		2968345	100.000	43945547	100.000

6.6 (4a*R*,8a*S*)-3-(4-fluoro-3-methylphenyl)-7-phenyl-4a,5,6,8a-tetrahydro-2*H*-chromene (3af)

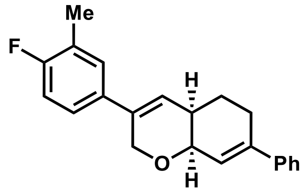

Isolated **3af** in 87% yield, as white solid (m.p.: 111.0 °C).

^{1}H NMR (400 MHz, $CDCl_3$) δ 7.46 (d, *J* = 7.6 Hz, 2H), 7.34 (overlapped, 2H), 7.29 (overlapped, 1H), 7.19 – 7.09 (overlapped, 2H), 6.97 (t, *J* = 8.9 Hz, 1H), 6.22 (d, *J* = 3.2 Hz, 1H), 6.12 (d, *J* = 4.9 Hz, 1H), 4.54 (m, 2H), 4.21 (br s, 1H), 2.58 (dt, *J* = 17.5, 4.3 Hz, 1H), 2.46 (m, 1H), 2.32 (m, 1H), 2.29 (s, 3H), 1.93 (m, 1H), 1.77 (m, 1H);

^{13}C NMR (100 MHz, $CDCl_3$) δ 160.9 (d, *J* = 245.3 Hz), 142.7, 141.4, 135.5, 134.1 (d, *J* = 3.5 Hz), 128.3 (2C), 128.1 (d, *J* = 5.1 Hz), 127.6, 125.6 (2C), 125.2 (d, *J* = 1.7 Hz), 124.8 (d, *J* = 17.4 Hz), 123.9 (d, *J* = 7.9 Hz), 122.7, 115.0 (d, *J* = 22.3 Hz), 69.6, 66.8, 34.3, 27.8, 25.2, 14.7 (d, *J* = 3.5 Hz);

^{19}F NMR (376 MHz, $CDCl_3$) δ -119.1;

HRESIMS (positive ion mode) *m/z* 343.1468 $[M + Na]^+$ (calcd. for $C_{22}H_{21}FONa$, 343.1469);

$[\alpha]_D^{24}$ = -54.3 (*c* = 0.15 g/100mL, acetone);

HPLC conditions: Chiralpak IC column (*n*-Hexane:2-Propanol = 90:10, 0.5 ml/min, 254nm); tr (minor) = 16.9 min, tr (major) = 13.6 min; 97% *ee*.

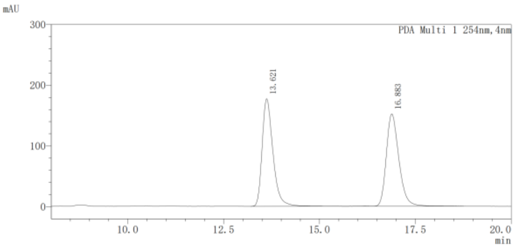

<Peak Table>

PDA Ch1 254nm

No.	Ret.Time(min)	Height	Height%	Area	Area%
1	13.621	177234	53.808	3474740	50.062
2	16.883	152147	46.192	3466127	49.938
Total		329381	100.000	6940867	100.000

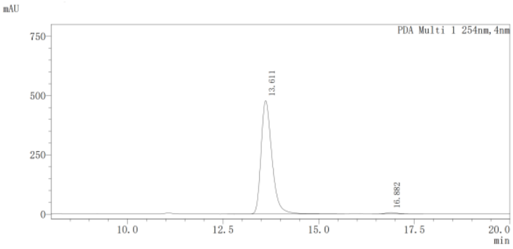

<Peak Table>

PDA Ch1 254nm

No.	Ret.Time(min)	Height	Height%	Area	Area%
1	13.611	478276	98.802	9468454	98.670
2	16.882	5800	1.198	127655	1.330
Total		484076	100.000	9596109	100.000

6.7 (4a*R*,8a*S*)-3-(3-methoxyphenyl)-7-phenyl-4a,5,6,8a-tetrahydro-2*H*-chromene (3ag)

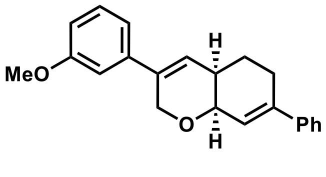

Isolated **3ag** in 61% yield, as white solid (m.p.: 118.6 °C).

^{1}H NMR (400 MHz, $CDCl_3$) δ 7.49 (d, *J* = 7.6 Hz, 2H), 7.38 (t, *J* = 7.5 Hz, 2H), 7.31 (overlapped, 2H), 6.98 (d, *J* = 7.7 Hz, 1H), 6.93 (s, 1H), 6.87 (d, *J* = 7.9 Hz, 1H), 6.26 (overlapped, 2H), 4.62 (q, *J* = 15.6 Hz, 2H), 4.27 (br s, 1H), 3.86 (s, 3H), 2.62 (dt, *J* = 17.5, 4.2 Hz, 1H), 2.49 (m, 1H), 2.37 (m, 1H), 1.97 (m, 1H), 1.83 (td, J = 12.3, 11.8, 5.0 Hz, 1H);

¹³C NMR (100 MHz, $CDCl_3$) δ 159.8, 142.7, 141.4, 139.7, 136.1, 129.5, 128.3 (2C), 127.6, 125.8, 125.6 (2C), 122.8, 117.5, 112.7, 111.0, 69.6, 66.8, 55.3, 34.4, 27.8, 25.2;

HRESIMS (positive ion mode) *m/z* 341.1515 $[M + Na]^+$ (calcd. for $C_{22}H_{22}O_2Na$, 341.1512);

$[\alpha]_D^{23}$ = -92.4 (*c* = 0.11 g/100mL, acetone);

HPLC conditions: Chiralpak IE column (*n*-Hexane:2-Propanol = 90:10, 0.5 ml/min, 254nm); tr (minor) = 24.0 min, tr (major) = 23.0 min; 84% *ee*.

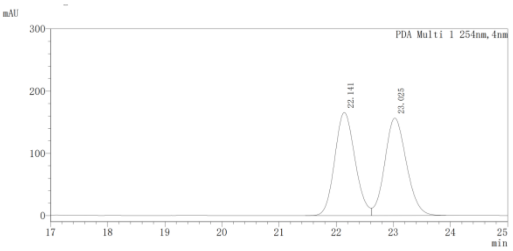

<Peak Table>
PDA Ch1 254nm

No.	Ret.Time(min)	Height	Height%	Area	Area%
1	22.141	165689	51.406	4083833	49.773
2	23.025	156624	48.594	4121069	50.227
Total		322313	100.000	8204902	100.000

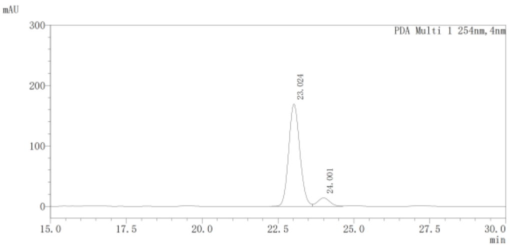

<Peak Table>
PDA Ch1 254nm

No.	Ret.Time(min)	Height	Height%	Area	Area%
1	23.024	169238	92.367	4329375	91.784
2	24.001	13986	7.633	387524	8.216
Total		183224	100.000	4716899	100.000

6.8 (4a*R*,8a*S*)-3-(naphthalen-2-yl)-7-phenyl-4a,5,6,8a-tetrahydro-2*H*-chromene (3ah)

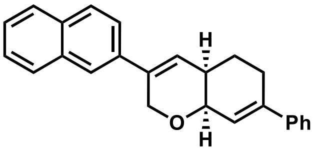

Isolated **3ah** in 72% yield, as white solid (m.p.: 193.9 °C).

¹H NMR (400 MHz, $CDCl_3$) δ 7.83 – 7.81 (overlapped, 3H), 7.69 (br s, 1H), 7.58 (d, *J* = 8.5 Hz, 1H), 7.48 – 7.43 (overlapped, 4H), 7.41 – 7.28 (overlapped, 3H), 6.38 (d, *J* = 5.0 Hz, 1H), 6.26 (d, *J* = 4.8 Hz, 1H), 4.76 (m, 2H), 4.28 (br d, 1H), 2.65 – 2.37 (overlapped, 3H), 1.97 (m, 1H), 1.84 (m, 1H);

¹³C NMR (100 MHz, $CDCl_3$) δ 142.8, 141.4, 135.9, 135.3, 133.4, 132.8, 128.3 (2C), 128.1, 128.1, 127.6 (2C), 126.3, 126.2, 125.9, 125.6 (2C), 123.4, 123.2, 122.8, 69.7, 66.8, 34.5, 27.9, 25.3;

HRESIMS (positive ion mode) *m/z* 361.1557 $[M + Na]^+$ (calcd. for $C_{25}H_{22}ONa$, 361.1563);

$[\alpha]_D^{26}$ = -46.4 (*c* = 0.15 g/100mL, acetone);

HPLC conditions: Chiralpak IC column (*n*-Hexane:2-Propanol = 90:10, 0.5 ml/min, 254nm); tr (minor) = 22.8 min, tr (major) = 17.1 min; 93% *ee*.

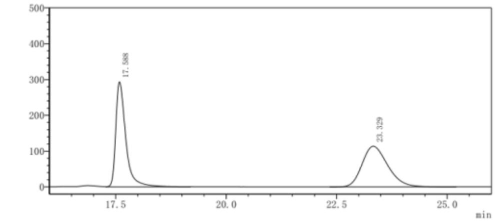

<Peak Table>
检测器A Ch1 254nm

No.	Ret.Time(min)	Height	Height%	Area	Area%
1	17.588	293195	72.099	4662526	50.251
2	23.329	113460	27.901	4615860	49.749
Total		406656	100.000	9278386	100.000

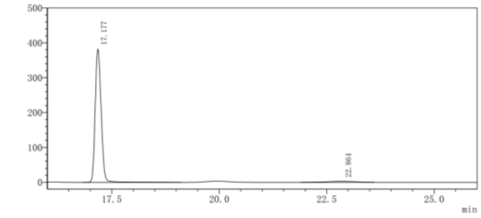

<Peak Table>
检测器A Ch1 254nm

No.	Ret.Time(min)	Height	Height%	Area	Area%
1	17.177	381938	99.341	3727550	96.743
2	22.864	2535	0.659	125491	3.257
Total		384473	100.000	3853041	100.000

6.9 (4a*R*,8a*S*)-3-(prop-1-en-2-yl)-7-phenyl-4a,5,6,8a-tetrahydro-2*H*-chromene (3ai)

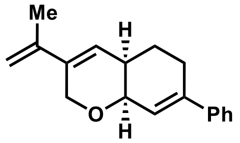

Isolated **3ai** in 68% yield, as white solid (m.p.: 96.3°C).

^{1}H NMR (400 MHz, $CDCl_3$) δ 7.45 (m, 2H), 7.34 (m, 2H), 7.28 (m, 1H), 6.20 (d, J = 5.0, 1H), 5.96 (d, J = 5.0 Hz, 1H), 4.90 (br s, 1H), 4.81 (br s, 1H), 4.45 (m, 2H), 4.13 (t, J = 4.6 Hz, 1H), 2.56 (m, 1H), 2.44 (m, 1H), 2.26 (dt, J = 11.7, 4.0 Hz, 1H), 1.94 (s, 3H), 1.90 (m, 1H), 1.75 (m, 1H);

^{13}C NMR (100 MHz, $CDCl_3$) δ 142.5, 141.4, 140.2, 135.9, 128.3 (2C), 127.5, 125.8, 125.6 (2C), 122.8, 110.2, 69.4, 66.0, 34.3, 27.9, 25.1, 20.4;

HRESIMS (positive ion mode) m/z 275.1405 [M + Na]$^+$ (calcd. for $C_{18}H_{20}ONa$, 275.1406);

[α]$_D^{26}$ = -154.8 (c = 0.09 g/100mL, MeOH);

HPLC conditions: Chiralpak IE column (n-Hexane:2-Propanol = 90:10, 0.5 ml/min, 254nm); tr (minor) = 11.9 min, tr (major) = 12.9 min; 96% *ee*.

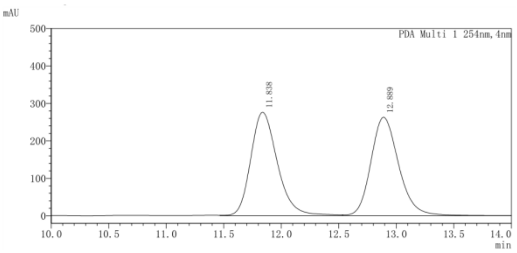

<Peak Table>
PDA Ch1 254nm

No.	Ret.Time(min)	Height	Height%	Area	Area%
1	11.838	276464	51.269	4461908	50.270
2	12.889	262777	48.731	4414016	49.730
Total		539241	100.000	8875924	100.000

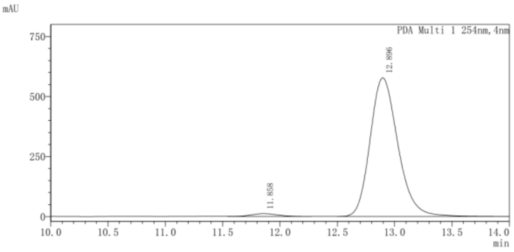

<Peak Table>
PDA Ch1 254nm

No.	Ret.Time(min)	Height	Height%	Area	Area%
1	11.858	11840	2.007	208592	2.089
2	12.896	578049	97.993	9778327	97.911
Total		589889	100.000	9986919	100.000

6.10 (4a*R*,8a*S*)-3-(but-3-en-1-yl)-7-phenyl-4a,5,6,8a-tetrahydro-2*H*-chromene (3aj)

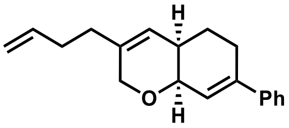

Isolated **3aj** in 77% yield, as colorless oil.

^{1}H NMR (400 MHz, $CDCl_3$) δ 7.45 (m, 2H), 7.35 (m, 2H), 7.28 (m, 1H), 6.18 (d, J = 3.7 Hz, 1H), 5.84 (ddt, J = 16.9, 10.2, 6.5 Hz, 1H), 5.57 (d, J = 3.3 Hz, 1H), 5.06 (dq, J = 17.1, 1.7 Hz, 1H), 4.99 (ddt, J = 10.2, 2.1, 1.2 Hz, 1H), 4.22 – 4.04 (overlapped, 3H), 2.55 (m, 1H), 2.41 (m, 1H), 2.26 – 2.12 (overlapped, 3H), 2.06 (t, J = 3.3 Hz, 2H), 1.84 (m, 1H), 1.69 (m, 1H);

^{13}C NMR (100 MHz, $CDCl_3$) δ 142.4, 141.5, 138.0, 136.5, 128.2 (2C), 127.4, 125.5 (2C), 123.0, 122.7, 114.8, 69.7, 67.7, 33.9, 32.4, 31.8, 27.7, 25.3;

HRESIMS (positive ion mode) m/z 289.1558 [M + Na]$^+$ (calcd. for $C_{19}H_{22}ONa$, 289.1563);

[α]$_D^{23}$ = -44.0 (c = 0.19 g/100mL, acetone);

HPLC conditions: Chiralpak IA column (*n*-Hexane:2-Propanol = 90:10, 0.5 ml/min, 254nm); tr(minor) = 10.4 min, tr (major) = 11.0 min; 93% *ee*.

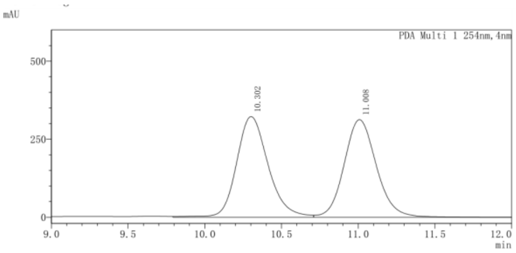

<Peak Table>
PDA Ch1 254nm

No.	Ret.Time(min)	Height	Height%	Area	Area%
1	10.302	323208	50.787	4649036	50.357
2	11.008	313187	49.213	4583153	49.643
Total		636396	100.000	9232189	100.000

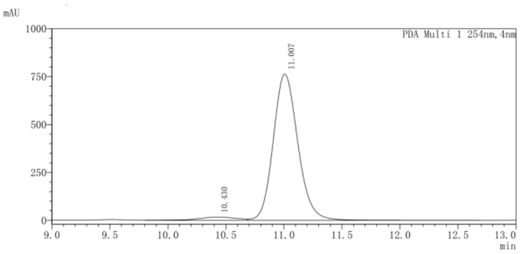

<Peak Table>
PDA Ch1 254nm

No.	Ret.Time(min)	Height	Height%	Area	Area%
1	10.430	16654	2.134	400010	3.423
2	11.007	763601	97.866	11286321	96.577
Total		780255	100.000	11686332	100.000

6.11 (4a*R*,8a*S*)-3-(pent-4-en-1-yl)-7-phenyl-4a,5,6,8a-tetrahydro-2*H*-chromene (3ak)

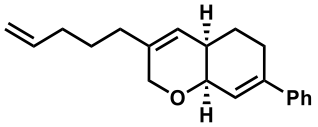

Isolated **3ak** in 76% yield, as colorless oil.

^{1}H NMR (400 MHz, $CDCl_3$) δ 7.45 (t, *J* = 7.9 Hz, 2H), 7.33 (overlapped, 3H), 6.19 (d, *J* = 6.8 Hz, 1H), 5.84 (dq, J = 17.2, 9.2, 8.7 Hz, 1H), 5.56 (m, 1H), 5.02 (dt, J = 18.7, 9.0 Hz, 2H), 4.14 (overlapped, 3H), 2.55 (m, 1H), 2.42 (m, 1H), 2.27 – 2.06 (overlapped, 3H), 1.99 (m, 2H), 1.83 (m, 1H), 1.74 – 1.49 (overlapped, 3H);

^{13}C NMR (100 MHz, $CDCl_3$) δ 142.5, 141.6, 138.5, 137.0, 128.3 (2C), 127.5, 125.6 (2C), 123.1, 122.6, 114.8, 69.8, 67.7, 33.9, 33.3, 32.5, 27.7, 26.9, 25.4;

HRESIMS (positive ion mode) *m/z* 303.1715 $[M + Na]^+$ (calcd. for $C_{20}H_{24}ONa$, 303.1719);

$[\alpha]_D^{24}$ = -118.7 (*c* = 0.07 g/100mL, acetone);

HPLC conditions: Chiralpak IC column (*n*-Hexane:2-Propanol = 95:5, 0.5 ml/min, 254nm); tr (minor) = 26.1 min, tr (major) = 14.1 min; 99% *ee*.

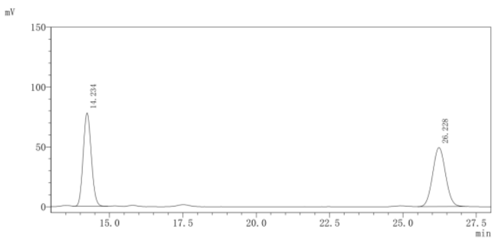

<Peak Table>
检测器A Ch1 254nm

No.	Ret.Time(min)	Height	Height%	Area	Area%
1	14.234	77946	61.219	1496421	49.645
2	26.228	49377	38.781	1517838	50.355
总计		127323	100.000	3014259	100.000

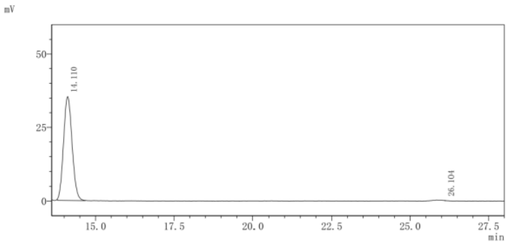

<Peak Table>
检测器A Ch1 254nm

No.	Ret.Time(min)	Height	Height%	Area	Area%
1	14.110	35235	99.742	665798	99.980
2	26.104	91	0.258	135	0.020
总计		35327	100.000	665933	100.000

6.12 (4a*R*,8a*S*)-3-methyl-7-phenyl-4a,5,6,8a-tetrahydro-2*H*-chromene (3al)

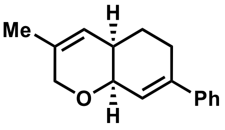

Isolated **3al** in 72% yield, as white solid (m.p.: 37.9 °C).

^{1}H NMR (400 MHz, $CDCl_3$) δ 7.46 (m, 2H), 7.35 (m, 2H), 7.28 (m, 1H), 6.18 (d, J = 2.9 Hz, 1H), 5.56 (br s, 1H), 4.18 – 4.01 (overlapped, 3H), 2.55 (dt, J = 17.3, 4.2 Hz, 1H), 2.42 (m, 1H), 2.12 (m, 1H), 1.83 (m, 1H), 1.69 (m, 1H), 1.67 (s, 3H);

^{13}C NMR (100 MHz, $CDCl_3$) δ 142.3, 141.5, 133.2, 128.2 (2C), 127.4, 125.5 (2C), 123.0, 122.9, 69.5, 68.7, 34.0, 27.8, 25.2, 18.7;

HRESIMS (positive ion mode) m/z 249.1249 [M + Na]$^+$ (calcd. for $C_{16}H_{18}ONa$, 249.1250);

$[\alpha]_D^{26}$ = -92.4 (c = 0.14 g/100mL, MeOH);

HPLC conditions: Chiralpak IE column (n-Hexane:2-Propanol = 90:10, 0.5 ml/min, 254nm); tr (minor) = 11.2 min, tr (major) = 13.2 min; 97% *ee*.

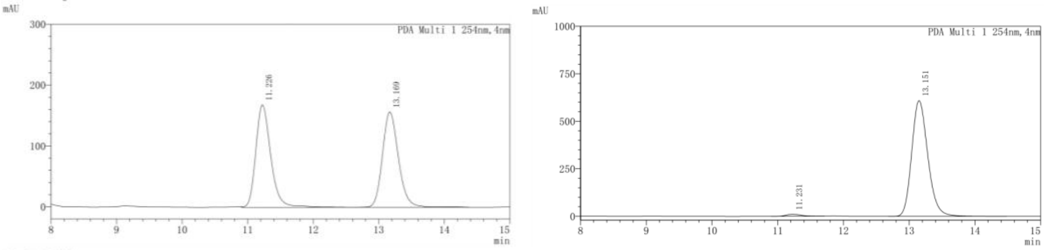

<Peak Table>
PDA Ch1 254nm

No.	Ret.Time(min)	Height	Height%	Area	Area%
1	11.226	168321	51.802	2689237	50.196
2	13.169	156609	48.198	2668216	49.804
Total		324930	100.000	5357452	100.000

<Peak Table>
PDA Ch1 254nm

No.	Ret.Time(min)	Height	Height%	Area	Area%
1	11.231	10907	1.759	165409	1.566
2	13.151	609331	98.241	10397171	98.434
Total		620239	100.000	10562580	100.000

6.13 (4a*R*,8a*S*)-3-ethyl-7-phenyl-4a,5,6,8a-tetrahydro-2*H*-chromene (3am)

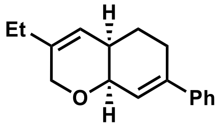

Isolated **3am** in 90% yield, as colorless oil.

^{1}H NMR (400 MHz, $CDCl_3$) δ 7.46 (d, J = 7.7 Hz, 2H), 7.35 (t, J = 7.5 Hz, 2H), 7.30 (m, 1H), 6.20 (d, J = 4.7 Hz, 1H), 5.55 (d, J = 4.6 Hz, 1H), 4.20 – 4.05 (overlapped, 3H), 2.55 (m, 1H), 2.44 (m, 1H), 2.15 (m, 1H), 1.99 (q, J = 7.8 Hz, 2H), 1.85 (m, 1H), 1.69 (qd, J = 12.0, 4.8 Hz, 1H), 1.07 (t, J = 7.6 Hz, 3H);

^{13}C NMR (100 MHz, $CDCl_3$) δ 142.4, 141.6, 138.7, 128.3 (2C), 127.5, 125.6 (2C), 123.1, 121.1, 69.8, 67.9, 34.0, 27.9, 25.7, 25.4, 12.1;

HRESIMS (positive ion mode) m/z 263.1404 [M + Na]$^+$ (calcd. for $C_{17}H_{20}ONa$, 263.1406);

$[\alpha]_D^{25}$ = -86.4 (c = 0.09 g/100mL, acetone);

HPLC conditions: Chiralpak IE column (*n*-Hexane:2-Propanol = 90:10, 0.5 ml/min, 254nm); tr (minor) = 10.6 min, tr (major) = 11.3 min; 92% *ee*.

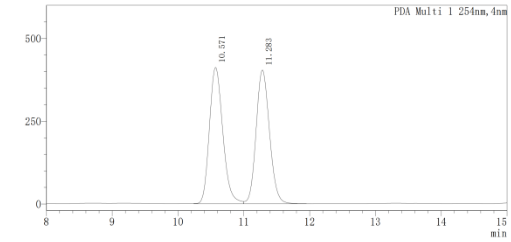

<Peak Table>

PDA Ch1 254nm

No.	Ret.Time(min)	Height	Height%	Area	Area%
1	10.571	412710	50.505	5864182	50.524
2	11.283	404455	49.495	5742450	49.476
Total		817164	100.000	11606632	100.000

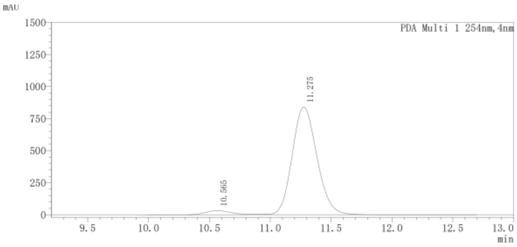

<Peak Table>

PDA Ch1 254nm

No.	Ret.Time(min)	Height	Height%	Area	Area%
1	10.565	32766	3.750	526371	4.233
2	11.275	841110	96.250	11907959	95.767
Total		873876	100.000	12434331	100.000

6.14 (4a*R*,8a*S*)-3-propyl-7-phenyl-4a,5,6,8a-tetrahydro-2*H*-chromene (3an)

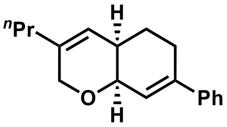

Isolated **3an** in 82% yield, as colorless oil.

^{1}H NMR (400 MHz, $CDCl_3$) δ 7.49 – 7.43 (m, 2H), 7.38 – 7.32 (m, 2H), 7.32 – 7.27 (m, 1H), 6.18 (d, J = 3.5 Hz, 1H), 5.55 (d, J = 4.9 Hz, 1H), 4.18 – 4.06 (overlapped, 3H), 2.60 – 2.51 (m, 1H), 2.42 (m, 1H), 2.16 (m, 1H), 1.95 (d, J = 7.6 Hz, 2H), 1.84 (m, 1H), 1.70 (m, 1H), 1.47 (m, 2H), 0.94 (t, J = 7.3 Hz, 3H);

^{13}C NMR (100 MHz, $CDCl_3$) δ 142.5, 141.6, 137.1, 128.3 (2C), 127.4, 125.6 (2C), 123.2, 122.4, 69.8, 67.7, 35.3, 33.9, 27.8, 25.4, 20.8, 13.8;

HRESIMS (positive ion mode) *m/z* 277.1560 $[M + Na]^+$ (calcd. for $C_{18}H_{22}ONa$, 277.1563);

$[\alpha]_D^{23}$ = -54.3 (c = 0.11 g/100mL, acetone);

HPLC conditions: Chiralpak IF column (*n*-Hexane:2-Propanol = 95:5, 0.5 ml/min, 254nm); tr (minor) = 10.8 min, tr (major) = 11.9 min; 97% *ee*.

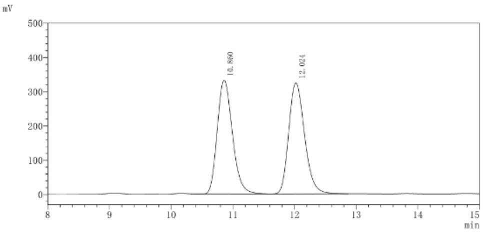

<Peak Table>

检测器A Ch1 254nm

No.	Ret.Time(min)	Height	Height%	Area	Area%
1	10.860	331807	50.555	5672404	49.418
2	12.024	324523	49.445	5805982	50.582
总计		656331	100.000	11478386	100.000

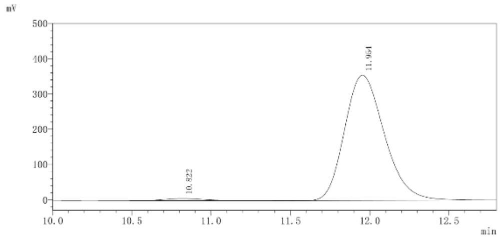

<Peak Table>

检测器A Ch1 254nm

No.	Ret.Time(min)	Height	Height%	Area	Area%
1	10.822	6016	1.670	102125	1.625
2	11.954	354321	98.330	6182430	98.375
总计		360337	100.000	6284555	100.000

6.15 (4a*R*,8a*S*)-3-butyl-7-phenyl-4a,5,6,8a-tetrahydro-2*H*-chromene (3ao)

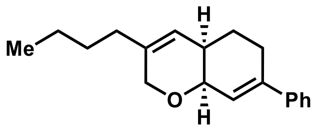

Isolated **3ao** in 91% yield, as white solid (m.p.: 42.0 °C)..

^{1}H NMR (400 MHz, $CDCl_3$) *δ* 7.45 (d, *J* = 7.3 Hz, 2H), 7.35 (t, *J* = 7.4 Hz, 2H), 7.29 (t, *J* = 7.1 Hz, 1H), 6.18 (d, *J* = 3.2 Hz, 1H), 5.55 (d, *J* = 3.8 Hz, 1H), 4.21 – 4.04 (overlapped, 3H), 2.55 (m, 1H), 2.42 (m, 1H), 2.16 (m, 1H), 1.98 (t, *J* = 7.3 Hz, 2H), 1.84 (m, 1H), 1.69 (ddt, *J* = 16.3, 12.9, 5.0 Hz, 2H), 1.44 – 1.35 (overlapped, 4H), 0.93 (t, *J* = 7.1 Hz, 3H);

^{13}C NMR (100 MHz, $CDCl_3$) *δ* 142.5, 141.6, 137.3, 128.3 (2C), 127.4, 125.6 (2C), 123.2, 122.2, 69.8, 67.7, 33.9, 32.9, 29.8, 27.8, 25.4, 22.4, 13.9;

HRESIMS (positive ion mode) *m/z* 291.1720 $[M + Na]^+$ (calcd. for $C_{19}H_{24}ONa$, 291.1719);

$[\alpha]_D^{24}$ = -43.9 (*c* = 0.16 g/100mL, acetone);

HPLC conditions: Chiralpak IE column (*n*-Hexane:2-Propanol = 90:10, 0.5 ml/min, 254nm); tr (minor) = 9.8 min, tr (major) = 10.2 min; 91% *ee*.

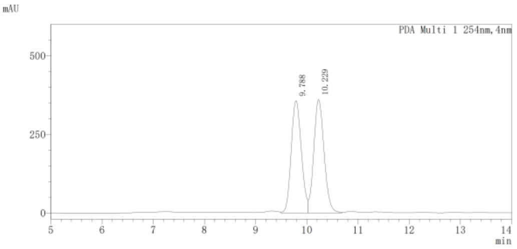

<Peak Table>
PDA Ch1 254nm

No.	Ret.Time(min)	Height	Height%	Area	Area%
1	9.788	356704	49.739	4934338	49.458
2	10.229	360453	50.261	5042443	50.542
Total		717157	100.000	9976781	100.000

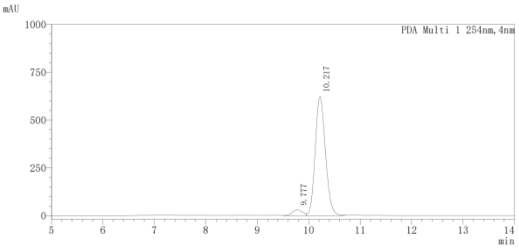

<Peak Table>
PDA Ch1 254nm

No.	Ret.Time(min)	Height	Height%	Area	Area%
1	9.777	30233	4.632	413912	4.577
2	10.217	622431	95.368	8630271	95.423
Total		652664	100.000	9044183	100.000

6.16 (4a*R*,8a*S*)-3-isopentyl-7-phenyl-4a,5,6,8a-tetrahydro-2*H*-chromene (3ap)

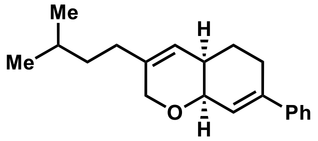

Isolated **3ap** in 64% yield, as colorless oil.

^{1}H NMR (400 MHz, $CDCl_3$) *δ* 7.45 (m, 2H), 7.34 (m, 2H), 7.28 (m, 1H), 6.17 (d, *J* = 3.5 Hz, 1H), 5.54 (d, *J* = 4.6 Hz, 1H), 4.19 – 4.07 (overlapped, 3H), 2.54 (m, 1H), 2.41 (m, 1H), 2.14 (m, 1H), 1.97 (t, *J* = 8.0 Hz, 2H), 1.83 (m, 1H), 1.68 (overlapped, 1H), 1.57 (m, 1H), 1.37 – 1.27 (overlapped, 2H), 0.92 (d, *J* = 6.8 Hz, 3H), 0.91 (d, *J* = 6.8 Hz, 3H);

^{13}C NMR (100 MHz, $CDCl_3$) *δ* 142.5, 141.6, 137.5, 128.3 (2C), 127.4, 125.6 (2C), 123.1, 122.0, 69.8, 67.8, 36.8, 34.0, 31.0, 27.8, 27.7, 25.4, 22.6, 22.5;

HRESIMS (positive ion mode) *m/z* 305.1874 $[M + Na]^+$ (calcd. for $C_{20}H_{26}ONa$, 305.1876);

$[\alpha]_D^{25}$ = -64.5 (*c* = 0.12 g/100mL, acetone);

HPLC conditions: Chiralpak IC column (*n*-Hexane:2-Propanol = 90:10, 0.5 ml/min, 254nm); tr (minor) = 15.3 min, tr (major) = 10.4 min; 95% *ee*.

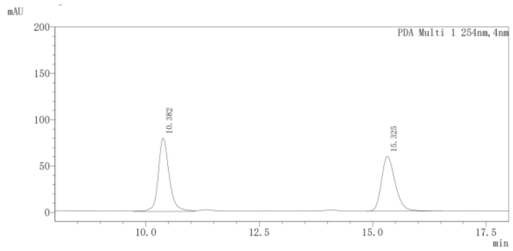

<Peak Table>
PDA Ch1 254nm

No.	Ret.Time(min)	Height	Height%	Area	Area%
1	10.382	79046	57.149	1318483	51.485
2	15.325	59269	42.851	1242410	48.515
Total		138314	100.000	2560893	100.000

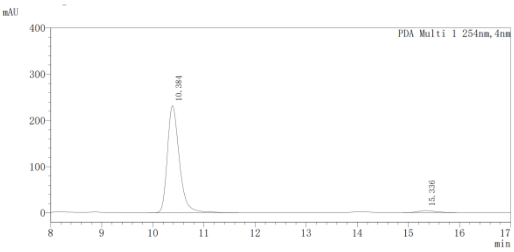

<Peak Table>
PDA Ch1 254nm

No.	Ret.Time(min)	Height	Height%	Area	Area%
1	10.384	230984	98.207	3739033	97.691
2	15.336	4218	1.793	88371	2.309
Total		235203	100.000	3827404	100.000

6.17 (4a*R*,8a*S*)-3-phenethyl-7-phenyl-4a,5,6,8a-tetrahydro-2*H*-chromene (3aq)

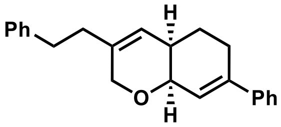

Isolated **3aq** in 78% yield, as white solid (m.p.: 61.0 °C).

^{1}H NMR (400 MHz, $CDCl_3$) δ 7.41 (d, *J* = 7.3 Hz, 2H), 7.31 (t, *J* = 7.4 Hz, 2H), 7.27 – 7.21 (overlapped, 3H), 7.21 – 7.11 (m, 3H), 6.14 (d, *J* = 4.7 Hz, 1H), 5.51 (d, *J* = 4.7 Hz, 1H), 4.17 – 4.04 (overlapped, 3H), 2.73 (h, *J* = 6.0 Hz, 2H), 2.48 (m, 1H), 2.38 (m, 1H), 2.23 (t, *J* = 7.9 Hz, 2H), 2.10 (m, 1H), 1.77 (m, 1H), 1.65 (m, 1H);

^{13}C NMR (100 MHz, $CDCl_3$) δ 142.5, 141.8, 141.5, 136.5, 128.4 (2C), 128.3 (2C), 128.3 (2C), 127.5, 125.9, 125.6 (2C), 123.1, 123.0, 69.8, 67.8, 34.9, 34.3, 34.0, 27.7, 25.3;

HRESIMS (positive ion mode) *m/z* 339.1717 $[M + Na]^+$ (calcd. for $C_{23}H_{24}ONa$, 339.1719);

$[\alpha]_D^{26}$ = -59.4 (*c* = 0.10 g/100mL, MeOH);

HPLC conditions: Chiralpak IC column (*n*-Hexane:2-Propanol = 90:10, 0.5 ml/min, 254nm); tr (minor) = 19.9 min, tr (major) = 13.4 min; 96% *ee*.

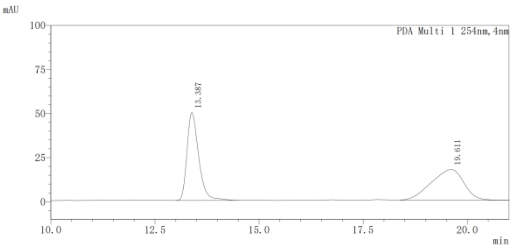

<Peak Table>
PDA Ch1 254nm

No.	Ret.Time(min)	Height	Height%	Area	Area%
1	13.387	49834	74.109	970139	50.457
2	19.611	17410	25.891	952584	49.543
Total		67244	100.000	1922723	100.000

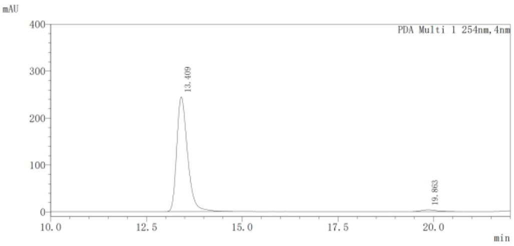

<Peak Table>
PDA Ch1 254nm

No.	Ret.Time(min)	Height	Height%	Area	Area%
1	13.409	244816	98.469	4816978	97.894
2	19.863	3806	1.531	103610	2.106
Total		248621	100.000	4920588	100.000

6.18 (4a*R*,8a*S*)-3-cyclopentyl-7-phenyl-4a,5,6,8a-tetrahydro-2*H*-chromene (3ar)

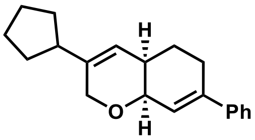

Isolated **3ar** in 51% yield, as white solid (m.p.: 74.6 °C).

^{1}H NMR (400 MHz, $CDCl_3$) δ 7.46 (d, *J* = 7.6 Hz, 2H), 7.35 (t, *J* = 7.5 Hz, 2H), 7.30 (d, *J* = 7.7 Hz, 1H), 6.19 (d, *J* = 4.8 Hz, 1H), 5.57 (d, *J* = 4.7 Hz, 1H), 4.28 – 4.09 (overlapped, 3H), 2.54 (m, 1H), 2.45 (m, 1H), 2.33 (m, 1H), 2.15 (m, 1H), 1.87 – 1.79 (overlapped, 4H), 1.74 – 1.50 (overlapped, 6H);

^{13}C NMR (100 MHz, $CDCl_3$) δ 142.5, 141.6, 140.4, 128.3 (2C), 127.4, 125.6 (2C), 123.2, 120.1, 69.9, 67.4, 43.4, 33.9, 31.0, 30.9, 27.9, 25.5, 25.0, 24.9;

HRESIMS (positive ion mode) *m/z* 303.1715 $[M + Na]^+$ (calcd. for $C_{20}H_{24}ONa$, 303.1719);

$[\alpha]_D^{25}$ = -17.1 (*c* = 0.08 g/100mL, acetone);

HPLC conditions: Chiralpak IC column (*n*-Hexane:2-Propanol = 90:10, 0.5 ml/min, 254nm); tr (minor) = 19.1 min, tr (major) = 11.2 min; 85% *ee*.

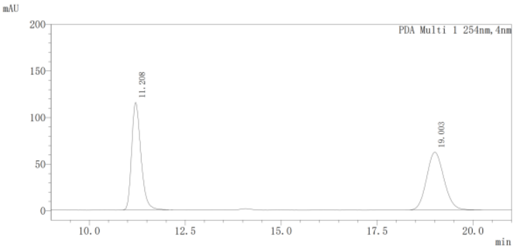

<Peak Table>
PDA Ch1 254nm

No.	Ret.Time(min)	Height	Height%	Area	Area%
1	11.208	115289	64.992	1946579	49.871
2	19.003	62101	35.008	1956624	50.129
Total		177391	100.000	3903203	100.000

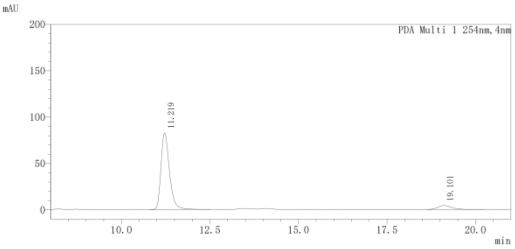

<Peak Table>
PDA Ch1 254nm

No.	Ret.Time(min)	Height	Height%	Area	Area%
1	11.219	82620	95.070	1366012	92.411
2	19.101	4284	4.930	112173	7.589
Total		86905	100.000	1478185	100.000

6.19 (4a*R*,8a*S*)-3-cyclohexyl-7-phenyl-4a,5,6,8a-tetrahydro-2*H*-chromene (3as)

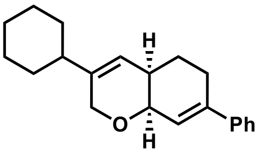

Isolated **3as** in 47% yield, as white solid (m.p.: 67.6 °C).

^{1}H NMR (400 MHz, $CDCl_3$) δ 7.45 (dd, *J* = 7.4, 1.8 Hz, 2H), 7.35 (t, *J* = 7.5 Hz, 2H), 7.29 (m, 1H), 6.17 (d, *J* = 4.5 Hz, 1H), 5.52 (d, *J* = 4.7 Hz, 1H), 4.23 – 4.09 (overlapped, 3H), 2.54 (dt, J = 17.4, 4.3 Hz, 1H), 2.42 (m, 1H), 2.15 (m, 1H), 1.86 – 1.73 (overlapped, 6H), 1.72 – 1.58 (overlapped, 3H), 1.28 – 1.15 (overlapped, 4H);

^{13}C NMR (100 MHz, $CDCl_3$) δ 142.5, 142.3, 141.7, 128.3 (2C), 127.4, 125.6 (2C), 123.2, 120.4, 69.9, 66.9, 41.9, 33.8, 32.1, 31.9, 27.8, 26.7 (2C), 26.3, 25.5;

HRESIMS (positive ion mode) *m/z* 317.1874 $[M + Na]^+$ (calcd. for $C_{21}H_{26}ONa$, 317.1876);

$[\alpha]_D^{24}$ = -85.9 (*c* = 0.09 g/100mL, acetone);

HPLC conditions: Chiralpak IC column (*n*-Hexane:2-Propanol = 90:10, 0.5 ml/min, 254nm); tr (minor) = 19.3 min, tr (major) = 11.0 min; 91% *ee*.

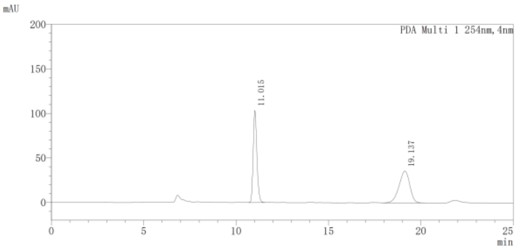

<Peak Table>
PDA Ch1 254nm

No.	Ret.Time(min)	Height	Height%	Area	Area%
1	11.015	103390	74.317	1441745	50.036
2	19.137	35730	25.683	1439659	49.964
Total		139120	100.000	2881404	100.000

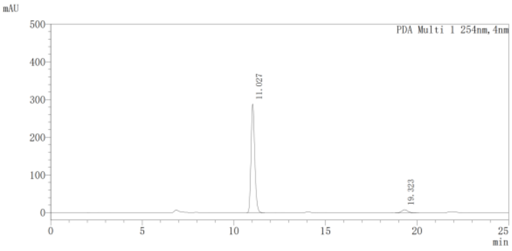

<Peak Table>
PDA Ch1 254nm

No.	Ret.Time(min)	Height	Height%	Area	Area%
1	11.027	288115	97.243	4040479	95.510
2	19.323	8170	2.757	189966	4.490
Total		296285	100.000	4230445	100.000

6.20 (4a*R*,8a*S*)-3-pentyl-7-phenyl-4a,5,6,8a-tetrahydro-2*H*-chromene (3at)

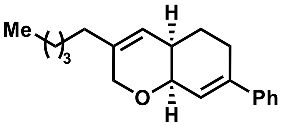

Isolated **3at** in 79% yield, as colorless oil.

^{1}H NMR (400 MHz, $CDCl_3$) δ 7.43 (m, 2H), 7.33 (m, 2H), 7.26 (m, 1H), 6.16 (d, J = 4.1 Hz, 1H), 5.52 (d, J = 4.6 Hz, 1H), 4.19 – 4.03 (overlapped, 3H), 2.52 (dt, J = 17.3, 4.0 Hz, 1H), 2.42 (m, 1H), 2.20 – 2.09 (m, 1H), 1.94 (t, J = 7.6 Hz, 2H), 1.82 (m, 1H), 1.67 (dtd, J = 12.9, 10.8, 5.0 Hz, 1H), 1.42 (m, 2H), 1.33 – 1.25 (overlapped, 4H), 0.89 (t, J = 6.9 Hz, 3H);

^{13}C NMR (100 MHz, $CDCl_3$) δ 142.4, 141.6, 137.3, 128.2 (2C), 127.4, 125.5 (2C), 123.1, 122.1, 69.7, 67.7, 33.9, 33.1, 31.5, 27.7, 27.3, 25.4, 22.5, 14.0;

HRESIMS (positive ion mode) *m/z* 305.1873 $[M + Na]^+$ (calcd. for $C_{20}H_{26}ONa$, 305.1876);

$[\alpha]_D^{23}$ = -47.5 (c = 0.17 g/100mL, acetone);

HPLC conditions: Chiralpak IA column (*n*-Hexane:2-Propanol = 90:10, 0.5 ml/min, 254nm); tr (minor) = 9.9 min, tr (major) = 9.2 min; 93% *ee*.

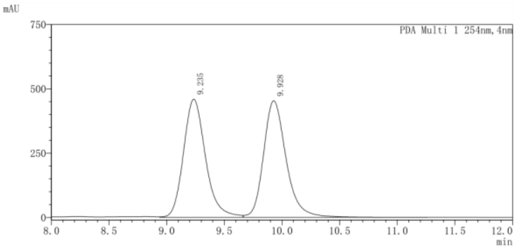

<Peak Table>
PDA Ch1 254nm

No.	Ret.Time(min)	Height	Height%	Area	Area%
1	9.235	459506	50.320	6116096	49.751
2	9.928	453660	49.680	6177278	50.249
Total		913166	100.000	12293374	100.000

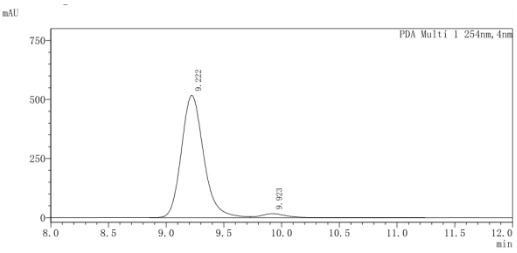

<Peak Table>
PDA Ch1 254nm

No.	Ret.Time(min)	Height	Height%	Area	Area%
1	9.222	517539	96.861	6958810	96.460
2	9.923	16773	3.139	255388	3.540
Total		534312	100.000	7214198	100.000

6.21 (4a*R*,8a*S*)-3-hexyl-7-phenyl-4a,5,6,8a-tetrahydro-2*H*-chromene (3au)

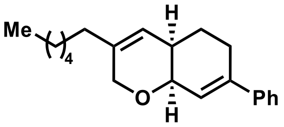

Isolated **3au** in 81% yield, as colorless oil.

^{1}H NMR (400 MHz, $CDCl_3$) δ 7.45 (m, 2H), 7.34 (m, 2H), 7.28 (m, 1H), 6.18 (d, *J* = 4.3 Hz, 1H), 5.54 (d, *J* = 4.6 Hz, 1H), 4.22 – 4.03 (overlapped, 3H), 2.54 (m, 1H), 2.41 (m, 1H), 2.15 (m, 1H), 1.96 (t, *J* = 7.6 Hz, 2H), 1.84 (m, 1H), 1.68 (dtd, *J* = 12.8, 10.8, 5.0 Hz, 1H), 1.47 – 1.48 (overlapped, 2H), 1.33 – 1.24 (overlapped, 6H), 0.91 (t, *J* = 6.9 Hz, 3H);

^{13}C NMR (100 MHz, $CDCl_3$) δ 142.4, 141.6, 137.3, 128.2 (2C), 127.4, 125.5 (2C), 123.1, 122.1, 69.7, 67.7, 33.9, 33.2, 31.7, 28.9, 27.7, 27.6, 25.4, 22.6, 14.1;

HRESIMS (positive ion mode) *m/z* 319.2029 $[M + Na]^+$ (calcd. for $C_{21}H_{28}ONa$, 319.2032);

$[\alpha]_D^{23}$ = -46.4 (*c* = 0.13 g/100mL, acetone);

HPLC conditions: Chiralpak IA column (*n*-Hexane:2-Propanol = 90:10, 0.5 ml/min, 254nm); tr (minor) = 9.7 min, tr (major) = 9.0 min;92% *ee*.

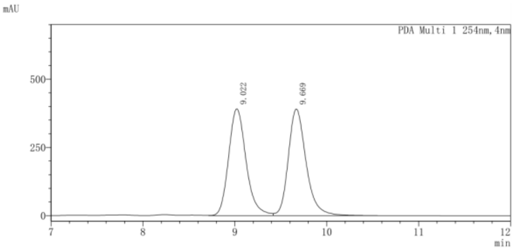

<Peak Table>

PDA Ch1 254nm

No.	Ret.Time(min)	Height	Height%	Area	Area%
1	9.022	391714	50.079	5196011	49.652
2	9.669	390480	49.921	5268886	50.348
Total		782194	100.000	10464897	100.000

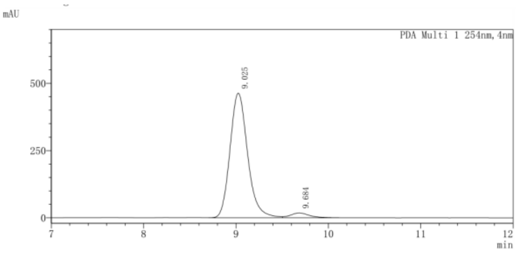

<Peak Table>

PDA Ch1 254nm

No.	Ret.Time(min)	Height	Height%	Area	Area%
1	9.025	463917	96.151	6167124	95.789
2	9.684	18573	3.849	271109	4.211
Total		482490	100.000	6438233	100.000

6.22 (4a*R*,8a*S*)-3-heptyl-7-phenyl-4a,5,6,8a-tetrahydro-2*H*-chromene (3av)

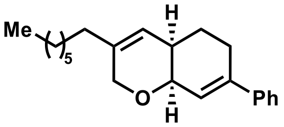

Isolated **3av** in 79% yield, as white solid(m.p.: 34.6 °C).

^{1}H NMR (400 MHz, $CDCl_3$) δ 7.45 (m, 2H), 7.35 (m, 2H), 7.28 (m, 1H), 6.18 (d, *J* = 4.2 Hz, 1H), 5.54 (d, *J* = 4.6 Hz, 1H). 4.20 – 4.06 (overlapped, 3H), 2.55 (dt, *J* = 17.4, 4.3 Hz, 1H), 2.42 (m, 1H), 2.16 (m, 1H), 1.97 (t, *J* = 7.6 Hz, 2H), 1.84 (m, 1H), 1.69 (m, 1H), 1.43 (m, 2H), 1.34 – 1.26 (overlapped, 8H), 0.91 (t, *J* = 6.8 Hz, 3H);

^{13}C NMR (100 MHz, $CDCl_3$) δ 142.4, 141.5, 137.3, 128.2 (2C), 127.4, 125.5 (2C), 123.1, 122.1, 69.7, 67.7, 33.9, 33.2, 31.8, 29.2, 29.1, 27.7, 27.6, 25.4, 22.6, 14.1;

HRESIMS (positive ion mode) *m/z* 333.2189 $[M + Na]^+$ (calcd. for $C_{22}H_{30}ONa$, 333.2189);

$[\alpha]_D^{25}$ = -75.4 (*c* = 0.16 g/100mL, acetone);

HPLC conditions: Chiralpak IA column (*n*-Hexane:2-Propanol = 90:10, 0.5 ml/min, 254nm); tr (minor) = 9.5 min, tr (major) = 8.8 min; 92% *ee*.

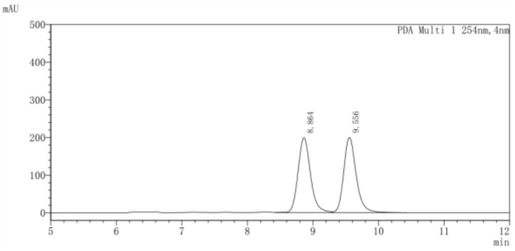

<Peak Table>
PDA Ch1 254nm

No.	Ret.Time(min)	Height	Height%	Area	Area%
1	8.864	199170	49.912	2635882	49.864
2	9.556	199873	50.088	2650226	50.136
Total		399042	100.000	5286108	100.000

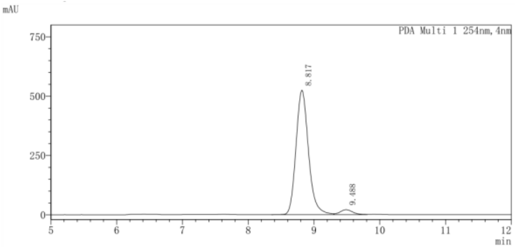

<Peak Table>
PDA Ch1 254nm

No.	Ret.Time(min)	Height	Height%	Area	Area%
1	8.817	524381	96.063	6964432	95.938
2	9.488	21492	3.937	294884	4.062
Total		545872	100.000	7259315	100.000

6.23 (4a*R*,8a*S*)-3-decyl-7-phenyl-4a,5,6,8a-tetrahydro-2*H*-chromene (3aw)

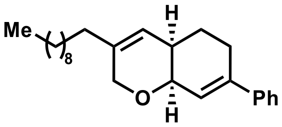

Isolated **3aw** in 86% yield, as white solid (m.p.: 38.0 °C).

^{1}H NMR (400 MHz, $CDCl_3$) δ 7.45 (m, 2H), 7.34 (m, 2H), 7.28 (m, 1H), 6.18 (d, *J* = 4.3 Hz, 1H), 5.54 (d, *J* = 4.6 Hz, 1H), 4.23 – 4.08 (overlapped, 3H), 2.54 (m, 1H), 2.42 (m, 1H), 2.16 (m, 1H), 1.96 (t, *J* = 7.6 Hz, 2H), 1.83 (m, 1H), 1.68 (m, 1H), 1.42 (m, 2H), 1.37 – 1.23 (m, 14H), 0.91 (t, *J* = 6.7 Hz, 3H);

^{13}C NMR (100 MHz, $CDCl_3$) δ 142.4, 141.6, 137.4, 128.3 (2C), 127.4, 125.6 (2C), 123.2, 122.2, 69.8, 67.7, 33.9, 33.2, 31.9, 29.7 (2C), 29.6, 29.5, 29.4, 27.7 (2C), 25.4, 22.7, 14.1;

HRESIMS (positive ion mode) *m/z* 375.2657 $[M + Na]^+$ (calcd. for $C_{25}H_{36}ONa$, 375.2658);

$[\alpha]_D^{23}$ = -83.3 (*c* = 0.12 g/100mL, acetone);

HPLC conditions: Chiralpak IC column (*n*-Hexane:2-Propanol = 90:10, 0.5 ml/min, 254nm); tr (minor) = 15.3 min, tr (major) = 9.7 min; 94% *ee*.

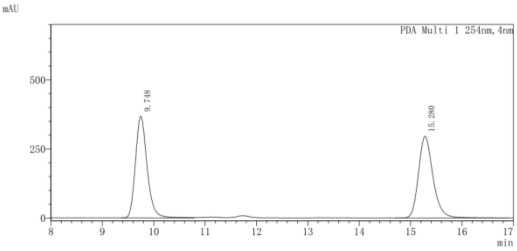

<Peak Table>
PDA Ch1 254nm

No.	Ret.Time(min)	Height	Height%	Area	Area%
1	9.748	367624	55.428	5785654	49.975
2	15.280	295617	44.572	5791520	50.025
Total		663241	100.000	11577175	100.000

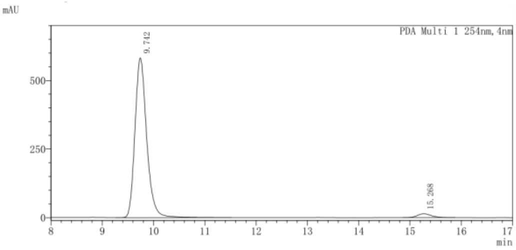

<Peak Table>
PDA Ch1 254nm

No.	Ret.Time(min)	Height	Height%	Area	Area%
1	9.742	581875	97.674	9245393	97.185
2	15.268	13857	2.326	267760	2.815
Total		595732	100.000	9513153	100.000

6.24 (4a*R*,8a*S*)-3-tetradecyl-7-phenyl-4a,5,6,8a-tetrahydro-2*H*-chromene (3ax)

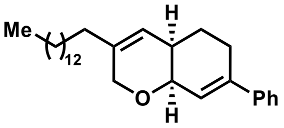

Isolated **3ax** in 83% yield, as white solid (m.p.: 49.1 °C).

^{1}H NMR (400 MHz, $CDCl_3$) δ 7.45 (dd, J = 7.3, 1.8 Hz, 2H), 7.34 (t, J = 7.4 Hz, 2H), 7.28 (m, 1H), 6.18 (d, J = 4.4 Hz, 1H), 5.54 (d, J = 4.7 Hz, 1H), 4.13 (overlapped, 3H), 2.54 (dt, J = 17.3, 4.3 Hz, 1H), 2.42 (m, 1H), 2.16 (m, 1H), 1.96 (t, J = 7.5 Hz, 2H), 1.84 (m, 1H), 1.77 – 1.60 (m, 2H), 1.42 (m, 2H), 1.29 (overlapped, 21H), 0.91 (t, J = 6.7 Hz, 3H);

^{13}C NMR (100 MHz, $CDCl_3$) δ 142.4, 141.6, 137.4, 128.3 (2C), 127.4, 125.6 (2C), 123.2, 122.2, 69.8, 67.7, 33.9, 33.2, 32.0, 29.7 (5C), 29.6, 29.5, 29.4, 29.3, 27.7 (2C), 25.4, 22.7, 14.1;

HRESIMS (positive ion mode) *m/z* 431.3288 $[M + Na]^+$ (calcd. for $C_{29}H_{44}ONa$, 431.3284);

$[\alpha]_D^{24}$ = -81.7 (c = 0.08 g/100mL, acetone);

HPLC conditions: Chiralpak IC column (*n*-Hexane:2-Propanol = 90:10, 0.5 ml/min, 254nm); tr (minor) = 12.2 min, tr (major) = 8.9 min; 95% *ee*.

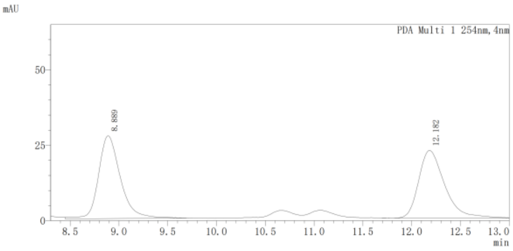

<Peak Table>
PDA Ch1 254nm

No.	Ret.Time(min)	Height	Height%	Area	Area%
1	8.889	27420	55.020	427859	50.862
2	12.182	22417	44.980	413364	49.138
Total		49838	100.000	841223	100.000

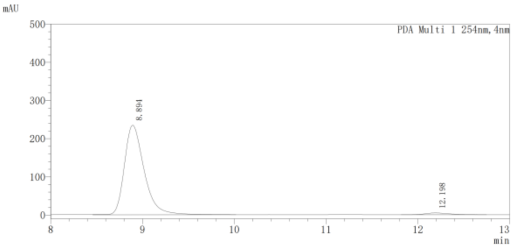

<Peak Table>
PDA Ch1 254nm

No.	Ret.Time(min)	Height	Height%	Area	Area%
1	8.894	234664	98.101	3500538	97.727
2	12.198	4542	1.899	81433	2.273
Total		239206	100.000	3581971	100.000

6.25 (4a*R*,8a*S*)-3-phenyl-7-(4-fluorophenyl)-4a,5,6,8a-tetrahydro-2*H*-chromene (3ba)

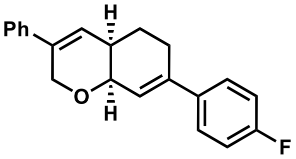

Isolated **3ba** in 87% yield, as white solid (m.p.: 122.0 °C).

^{1}H NMR (400 MHz, $CDCl_3$) δ 7.42 (m, 2H), 7.35 (overlapped, 4H), 7.29 (m, 1H), 7.03 (m, 2H), 6.21 (d, J = 4.8 Hz, 1H), 6.17 (d, J = 4 Hz, 1H), 4.60 (m, 2H), 4.22 (t, J = 4.2 Hz, 1H), 2.54 (m, 1H), 2.44 (m, 1H), 2.33 (m, 1H), 1.94 (m, 1H), 1.78 (m, 1H);[1]

^{13}C NMR (100 MHz, $CDCl_3$) δ 162.4 (d, J = 246.4 Hz), 141.7, 138.1, 137.4 (d, J = 3.3 Hz), 136.1, 128.5 (2C), 127.5, 127.1 (d, J = 8.0 Hz, 2C), 125.4, 124.9 (2C), 122.6 (d, J = 1.4 Hz), 115.1 (d, J = 21.2 Hz, 2C), 69.5, 66.8, 34.2, 27.9, 25.1;

^{19}F NMR (376 MHz, $CDCl_3$) δ -115.0;

HRESIMS (positive ion mode) *m/z* 329.1309 $[M + Na]^+$ (calcd. for $C_{21}H_{19}FONa$, 329.1312);

$[\alpha]_D^{25}$ = -37.4 (c = 0.12 g/100mL, MeOH);

HPLC conditions: Chiralpak ID column (n-Hexane:2-Propanol = 90:10, 0.5 ml/min, 254nm); tr (minor) = 12.9 min, tr (major) = 16.4 min; 98% *ee*.

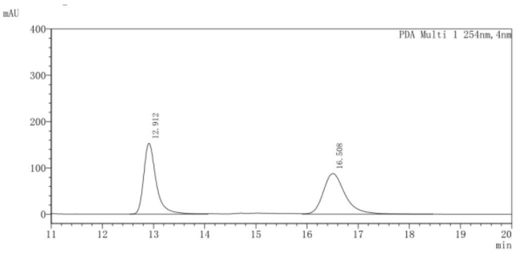

<Peak Table>
PDA Ch1 254nm

No.	Ret.Time(min)	Height	Height%	Area	Area%
1	12.912	153054	63.553	2592891	49.794
2	16.508	87776	36.447	2614354	50.206
Total		240831	100.000	5207245	100.000

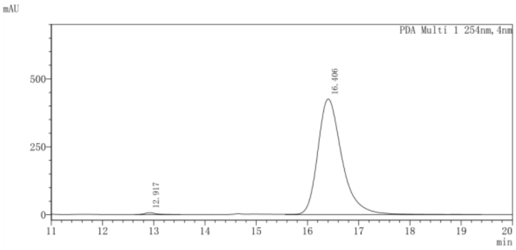

<Peak Table>
PDA Ch1 254nm

No.	Ret.Time(min)	Height	Height%	Area	Area%
1	12.917	7021	1.624	120279	0.853
2	16.406	425270	98.376	13980301	99.147
Total		432292	100.000	14100580	100.000

6.26 (4a*R*,8a*S*)-3-phenyl-7-(4-chlorophenyl)-4a,5,6,8a-tetrahydro-2*H*-chromene (3bb)

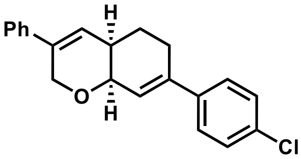

Isolated **3bb** in 76% yield, as white solid (m.p.: 174.0 °C).

^{1}H NMR (400 MHz, $CDCl_3$) δ 7.41 – 7.28 (overlapped, 9H), 6.21 (overlapped, 2H), 4.60 (overlapped, 2H), 4.22 (t, J = 4.6 Hz, 1H), 2.54 (dt, J = 17.2, 4.2 Hz, 1H), 2.41 (m, 1H), 2.33 (m, 1H), 1.94 (m, 1H), 1.77 (dtd, J = 12.9, 11.1, 5.0 Hz, 1H);

^{13}C NMR (100 MHz, $CDCl_3$) δ 141.6, 139.8, 138.1, 136.2, 133.4, 128.6 (2C), 128.5 (2C), 127.6, 126.9 (2C), 125.3, 124.9 (2C), 123.3, 69.5, 66.9, 34.3, 27.8, 25.1;

HRESIMS (positive ion mode) m/z 345.1014 $[M + Na]^+$ (calcd. for $C_{21}H_{19}ClONa$, 345.1017);

$[\alpha]_D^{24}$ = -48.9 (c = 0.11 g/100mL, acetone);

HPLC conditions: Chiralpak IE column (n-Hexane:2-Propanol = 90:10, 0.5 ml/min, 254nm); tr (minor) = 13.1 min, tr (major) = 16.4 min; 99% *ee*.

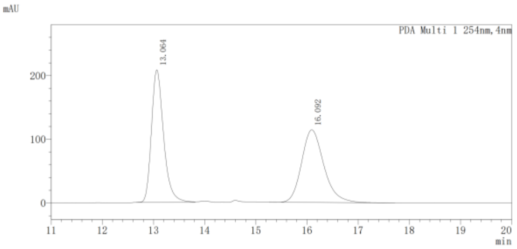

<Peak Table>
PDA Ch1 254nm

No.	Ret.Time(min)	Height	Height%	Area	Area%
1	13.064	207882	64.602	3429991	49.816
2	16.092	113905	35.398	3455297	50.184
Total		321787	100.000	6885288	100.000

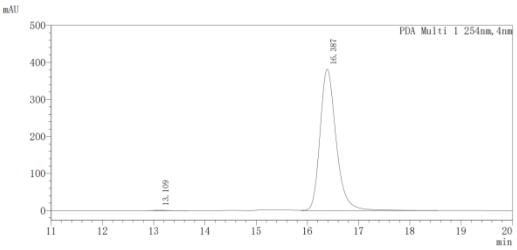

<Peak Table>
PDA Ch1 254nm

No.	Ret.Time(min)	Height	Height%	Area	Area%
1	13.109	1141	0.298	21621	0.258
2	16.387	381777	99.702	8350078	99.742
Total		382918	100.000	8371698	100.000

6.27 (4a*R*,8a*S*)-3-phenyl-7-(*p*-tolyl)-4a,5,6,8a-tetrahydro-2*H*-chromene (3bc)

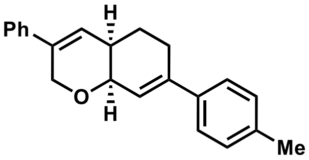

Isolated **3bc** in 91% yield, as white solid (m.p.: 166.0 °C).

^{1}H NMR (400 MHz, $CDCl_3$) δ 7.38 – 7.34 (overlapped, 6H), 7.29 (m, 1H), 7.16 (*d*, J = 7.9 Hz, 2H), 6.22 – 6.20 (overlapped, 2H), 4.62 (m, 2H), 4.24 (d, J = 4.6 Hz, 1H), 2.58 (dt, J = 17.3, 4.3 Hz, 1H), 2.45 (m, 1H), 2.36 (s, 3H), 2.33 (m, 1H), 1.94 (m, 1H), 1.79 (dtd, J = 12.9, 11.0, 5.0 Hz, 1H);

^{13}C NMR (100 MHz, $CDCl_3$) δ 142.5, 138.6, 138.3, 137.3, 136.1, 129.0 (2C), 128.5 (2C), 127.5, 125.6, 125.5 (2C), 125.0 (2C), 121.9, 69.7, 66.8, 34.4, 27.8, 25.3, 21.1;

HRESIMS (positive ion mode) *m/z* 303.1742 [M + H]$^+$ (calcd. for $C_{22}H_{23}O$, 303.1743);

[α]$_D^{25}$ = -38.1 (*c* = 0.09 g/100mL, acetone);

HPLC conditions: Chiralpak ID column (*n*-Hexane:2-Propanol = 90:10, 0.5 ml/min, 254nm); tr (minor) = 12.9 min, tr (major) = 16.3 min; 93% *ee.*

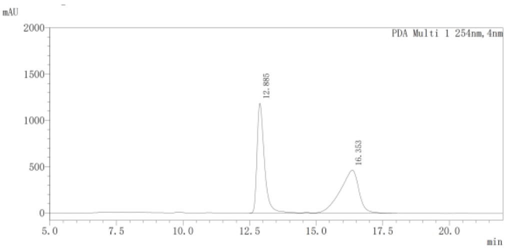

<Peak Table>
PDA Ch1 254nm

No.	Ret.Time(min)	Height	Height%	Area	Area%
1	12.885	1187127	71.857	23085824	50.185
2	16.353	464945	28.143	22915199	49.815
Total		1652072	100.000	46001023	100.000

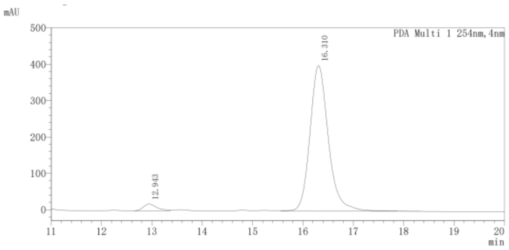

<Peak Table>
PDA Ch1 254nm

No.	Ret.Time(min)	Height	Height%	Area	Area%
1	12.943	18634	4.450	359495	3.408
2	16.310	400068	95.550	10190388	96.592
Total		418702	100.000	10549883	100.000

6.28 (4a*R*,8a*S*)-3-phenyl-7-(4-(trifluoromethoxy)phenyl)-4a,5,6,8a-tetrahydro-2*H*-chromene (3bd)

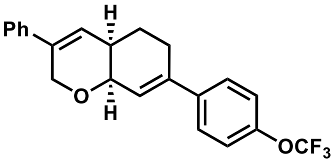

Isolated **3bd** in 73% yield, as white solid (m.p.: 159.0 °C).

^{1}H NMR (400 MHz, $CDCl_3$) δ 7.46 (d, J = 8.7 Hz, 2H), 7.37 – 7.32 (overlapped, 4H), 7.29 (m, 1H), 7.18 (d, J = 8.4 Hz, 2H), 6.21 (overlapped, 2H), 4.61 (m, 2H), 4.23 (t, J = 4.5 Hz, 1H), 2.55 (dt, J = 17.3, 4.3 Hz, 1H), 2.44 (m, 1H), 2.34 (m, 1H), 1.95 (m, 1H), 1.78 (dtd, J = 12.9, 11.0, 5.1 Hz, 1H);

^{13}C NMR (100 MHz, $CDCl_3$) δ 148.6, 141.5, 140.1, 138.1, 136.2, 128.6 (2C), 127.6, 126.9 (2C), 125.3, 124.9 (2C), 123.6, 120.7 (2C), 69.4, 66.9, 34.2, 27.8, 25.1;

^{19}F NMR (376 MHz, $CDCl_3$) δ -57.8 (3F);

HRESIMS (positive ion mode) *m/z* 395.1222 [M + Na]$^+$ (calcd. for $C_{22}H_{19}F_3O_2Na$, 395.1229);

[*α*]$_D^{24}$ = -87.4 (*c* = 0.12 g/100mL, acetone);

HPLC conditions: Chiralpak IE column (*n*-Hexane:2-Propanol = 90:10, 0.5 ml/min, 254nm); tr (minor) = 12.2 min, tr (major) = 12.6 min; 98% *ee*.

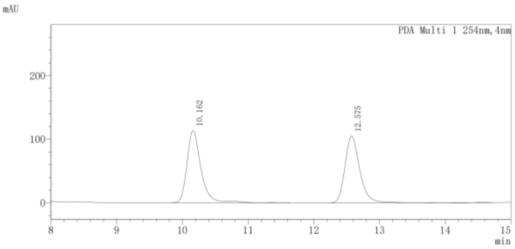

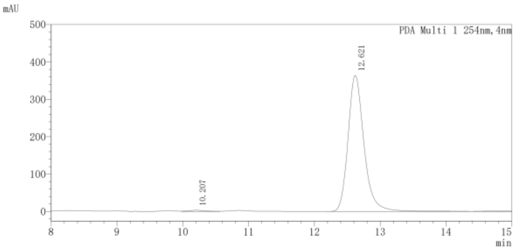

<Peak Table>
PDA Ch1 254nm

No.	Ret.Time(min)	Height	Height%	Area	Area%
1	10.162	113523	51.968	1690950	50.784
2	12.575	104926	48.032	1638729	49.216
Total		218449	100.000	3329679	100.000

<Peak Table>
PDA Ch1 254nm

No.	Ret.Time(min)	Height	Height%	Area	Area%
1	10.207	3249	0.885	56773	0.897
2	12.621	363934	99.115	6274321	99.103
Total		367183	100.000	6331094	100.000

6.2 (4a*R*,8a*S*)-3-phenyl-7-(4-(trifluoromethyl)phenyl)-4a,5,6,8a-tetrahydro-2*H*-chromene (3be)

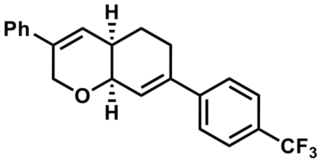

Isolated **3be** in 86% yield, as white solid (m.p.: 168.0 °C).

^{1}H NMR (400 MHz, $CDCl_3$) *δ* 7.67 – 7.54 (overlapped, 4H), 7.40 – 7.35 (overlapped, 4H), 7.31 (m, 1H), 6.32 (d, *J* = 3.8 Hz, 1H), 6.24 (d, *J* = 5.0 Hz, 1H), 4.64 (m, 2H), 4.27 (t, *J* = 4.6 Hz, 1H), 2.60 (dt, *J* = 17.4, 4.3 Hz, 1H), 2.50 (m, 1H), 2.38 (m, 1H), 1.99 (m, 1H), 1.82 (m, 1H);

^{13}C NMR (100 MHz, $CDCl_3$) *δ* 144.9, 141.6, 138.1, 136.3, 128.6 (2C), 127.6, 125.9 (2C), 125.4, 125.3 (q, *J* = 3.8 Hz, 2C), 124.9 (2C), 124.8, 69.4, 66.9, 34.2, 27.8, 25.1;

^{19}F NMR (376 MHz, $CDCl_3$) *δ* -62.5 (3F);

HRESIMS (positive ion mode) *m/z* 357.1460 [M + H]$^+$ (calcd. for $C_{22}H_{20}F_3O$, 357.1461);

[*α*]$_D^{25}$ = -68.8 (*c* = 0.06 g/100mL, MeOH:$CHCl_3$ = 2:1);

HPLC conditions: Chiralpak ID column (*n*-Hexane:2-Propanol = 90:10, 0.5 ml/min, 254nm); tr (minor) = 10.7 min, tr (major) = 13.3 min; 97% *ee*.

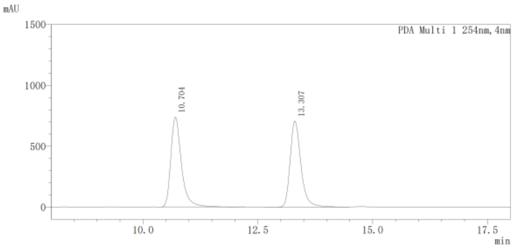

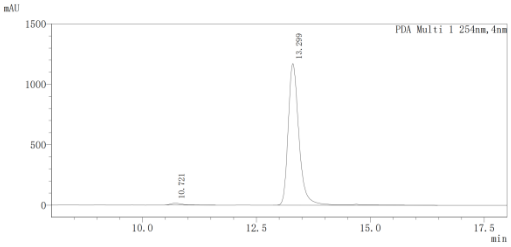

<Peak Table>
PDA Ch1 254nm

No.	Ret.Time(min)	Height	Height%	Area	Area%
1	10.704	738458	51.102	11869522	50.714
2	13.307	706603	48.898	11535081	49.286
Total		1445061	100.000	23404602	100.000

<Peak Table>
PDA Ch1 254nm

No.	Ret.Time(min)	Height	Height%	Area	Area%
1	10.721	15256	1.283	276531	1.399
2	13.299	1173445	98.717	19483765	98.601
Total		1188701	100.000	19760296	100.000

6.30 trimethyl(4-((4a*R*,8a*S*)-3-phenyl-4a,5,6,8a-tetrahydro-2*H*-chromen-7-yl)phenyl)silane (3bf)

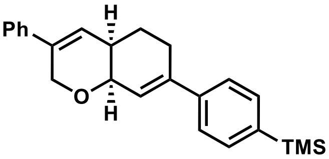

Isolated **3bf** in 88% yield, as white solid (m.p.: 177.0 °C).

^{1}H NMR (400 MHz, $CDCl_3$) *δ* 7.53 (d, *J* = 7.7 Hz, 2H), 7.46 (d, *J* = 7.7 Hz, 2H), 7.36 (overlapped, 4H), 7.30 (m, 1H), 6.26 (d, *J* = 3.8 Hz, 1H), 6.22 (d, *J* = 4.9 Hz, 1H), 4.62 (m, 2H), 4.26 (t, *J* = 4.4 Hz, 1H), 2.59 (m, 1H), 2.49 (m, 1H), 2.36 (m, 1H), 1.96 (m, 1H), 1.81 (m, 1H), 0.30 (s, 9H);

^{13}C NMR (100 MHz, $CDCl_3$) *δ* 142.7, 141.8, 139.8, 138.2, 136.2, 133.4 (2C), 128.6 (2C), 127.5, 125.5, 125.0 (2C), 124.9 (2C), 122.9, 69.7, 66.8, 34.4, 27.7, 25.3, -1.08 (3C);

HRESIMS (positive ion mode) *m/z* 383.1799 [M + Na]$^+$ (calcd. for $C_{24}H_{28}OSiNa$, 383.1802);

$[\alpha]_D^{25}$ = -71.0 (*c* = 0.13 g/100mL, acetone);

HPLC conditions: Chiralpak IC column (*n*-Hexane:2-Propanol = 90:10, 0.5 ml/min, 254nm); tr (minor) = 13.1 min, tr (major) = 21.0 min; 90% *ee*.

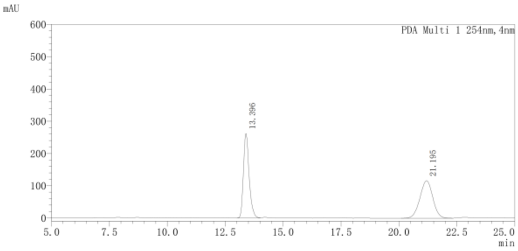

<Peak Table>
PDA Ch1 254nm

No.	Ret.Time(min)	Height	Height%	Area	Area%
1	13.396	262445	69.257	4495059	49.989
2	21.195	116499	30.743	4497058	50.011
Total		378944	100.000	8992118	100.000

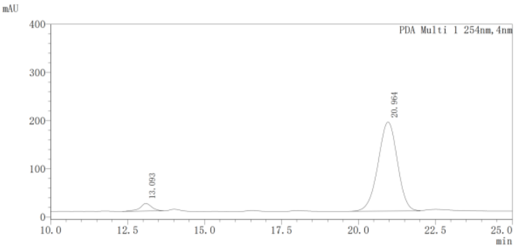

<Peak Table>
PDA Ch1 254nm

No.	Ret.Time(min)	Height	Height%	Area	Area%
1	13.093	15766	7.872	415582	4.883
2	20.964	184518	92.128	8094904	95.117
Total		200284	100.000	8510486	100.000

6.31 (4a*R*,8a*S*)-3-phenyl-7-(3-fluorophenyl)-4a,5,6,8a-tetrahydro-2*H*-chromene (3bg)

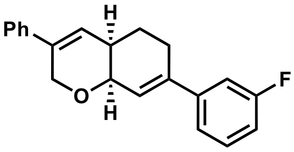

Isolated **3bg** in 74% yield, as white solid (m.p.: 118.3 °C).

^{1}H NMR (400 MHz, $CDCl_3$) *δ* 7.37 – 7.34 (overlapped, 4H), 7.33 – 7.28 (overlapped, 2H), 7.24 (m, 1H), 7.15 (dt, *J* = 10.4, 2.1 Hz, 1H), 6.98 (m, 1H), 6.26 (d, *J* = 4.9 Hz, 1H), 6.21 (d, *J* = 4.7 Hz, 1H), 4.61 (tdd, *J* = 15.7, 14.5, 1.9 Hz, 2H), 4.24 (t, *J* = 4.6 Hz, 1H), 2.55 (dt, *J* = 17.3, 4.2 Hz, 1H), 2.43 (m, 1H), 2.34 (m, 1H), 1.95 (m, 1H), 1.79 (dtd, *J* = 12.9, 11.0, 5.0 Hz, 1H);

^{13}C NMR (100 MHz, $CDCl_3$) *δ* 162.9 (d, *J* = 245.1 Hz), 143.7 (d, *J* = 7.3 Hz), 141.6 (d, *J* = 2.2 Hz), 138.1, 136.2, 129.7 (d, *J* = 8.4 Hz), 128.6 (2C), 127.6, 125.3, 125.0 (2C), 123.8, 121.2 (d, *J* = 2.8 Hz), 114.33 (d, *J* = 21.2 Hz), 112.6 (d, *J* = 22.1 Hz), 69.5, 66.9, 34.3, 27.7, 25.1;

^{19}F NMR (376 MHz, $CDCl_3$) *δ* -113.4;

HRESIMS (positive ion mode) *m/z* 329.1309 [M + Na]$^+$ (calcd. for $C_{21}H_{19}FONa$, 329.1312);

[α]$_D$^{25} = -67.3 (*c* = 0.12 g/100mL, MeOH);

HPLC conditions: Chiralpak IE column (*n*-Hexane:2-Propanol = 90:10, 0.5 ml/min, 254nm); tr (minor) = 12.6 min, tr (major) = 15.9 min; 98% *ee*.

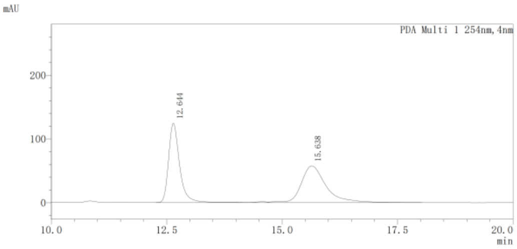

<Peak Table>
PDA Ch1 254nm

No.	Ret.Time(min)	Height	Height%	Area	Area%
1	12.644	124620	68.436	2098337	49.421
2	15.638	57477	31.564	2147474	50.579
Total		182097	100.000	4245811	100.000

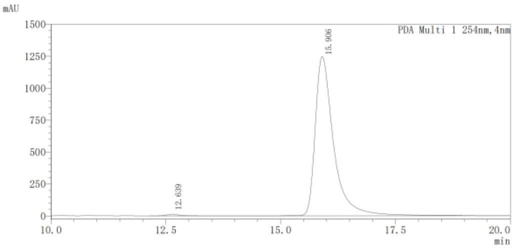

<Peak Table>
PDA Ch1 254nm

No.	Ret.Time(min)	Height	Height%	Area	Area%
1	12.639	13344	1.058	277483	0.788
2	15.906	1248102	98.942	34940922	99.212
Total		1261447	100.000	35218406	100.000

6.32 (4a*R*,8a*S*)-3-phenyl-7-(*m*-tolyl)-4a,5,6,8a-tetrahydro-2*H*-chromene (3bh)

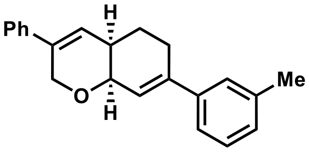

Isolated **3bh** in 93% yield, as white solid (m.p.: 106.3 °C).

^{1}H NMR (400 MHz, $CDCl_3$) δ 7.43 – 7.35 (overlapped, 4H), 7.34 – 7.25 (overlapped, 4H), 7.14 (d, *J* = 6.3 Hz, 1H), 6.25 – 6.23 (overlapped, 2H), 4.64 (m, 2H), 4.26 (t, *J* = 4.6 Hz, 1H), 2.60 (m, 1H), 2.48 (m, 1H), 2.40 (s, 3H), 2.36 (m, 1H), 1.97 (m, 1H), 1.81 (dtd, *J* = 12.9, 10.9, 5.0 Hz, 1H);

^{13}C NMR (100 MHz, $CDCl_3$) δ 142.9, 141.5, 138.2, 137.8, 136.2, 128.5 (2C), 128.3, 128.2, 127.5, 126.5, 125.6, 125.0 (2C), 122.7, 122.6, 69.7, 66.8, 34.4, 27.9, 25.3, 21.5;

HRESIMS (positive ion mode) *m/z* 303.1741 [M + H]$^+$ (calcd. for $C_{22}H_{23}O$, 303.1743);

[α]$_D$^{25} = -112,9 (*c* = 0.10 g/100mL, acetone);

HPLC conditions: Chiralpak IC column (*n*-Hexane:2-Propanol = 90:10, 0.5 ml/min, 254nm); tr (minor) = 11.9 min, tr (major) = 14.7 min; 98% *ee*.

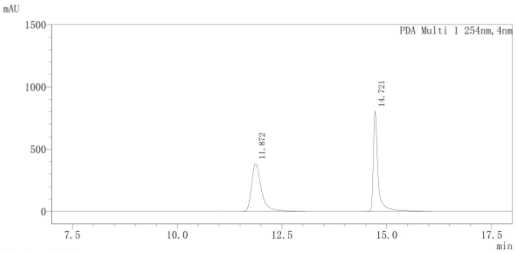

<Peak Table>
PDA Ch1 254nm

No.	Ret.Time(min)	Height	Height%	Area	Area%
1	11.872	383144	32.245	6224744	49.694
2	14.721	805097	67.755	6301444	50.306
Total		1188241	100.000	12526188	100.000

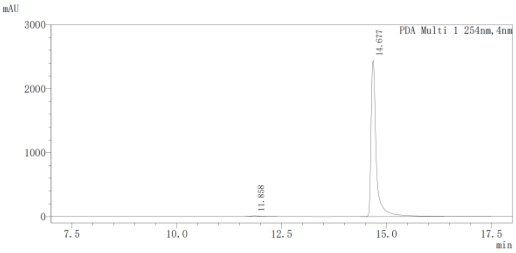

<Peak Table>
PDA Ch1 254nm

No.	Ret.Time(min)	Height	Height%	Area	Area%
1	11.858	11232	0.458	189841	0.881
2	14.677	2440430	99.542	21350844	99.119
Total		2451662	100.000	21540685	100.000

6.33 (4a*R*,8a*S*)-3-phenyl-7-(3-methoxyphenyl)-4a,5,6,8a-tetrahydro-2*H*-chromene (3bi)

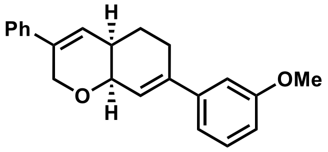

Isolated **3bi** in 88% yield, as white solid (m.p.: 124.0°C).

^{1}H NMR (400 MHz, $CDCl_3$) δ 7.38 – 7.34 (overlapped, 4H), 7.34 – 7.26 (m, 2H), 7.10 – 7.01 (m, 2H), 6.86 (dd, *J* = 8.2, 2.6 Hz, 1H), 6.28 – 6.20 (m, 2H), 4.63 (m, 2H), 4.25 (t, *J* = 4.5 Hz, 1H), 3.84 (s, 3H), 2.59 (m, 1H), 2.46 (m, 1H), 2.35 (dt, *J* = 12.0, 3.8 Hz, 1H), 1.95 (m, 1H), 1.80 (dtd, *J* = 13.0, 11.0, 5.1 Hz, 1H);

^{13}C NMR (100 MHz, $CDCl_3$) δ 159.6, 143.0, 142.7, 138.2, 136.2, 129.3, 128.6 (2C), 127.5, 125.5, 125.0 (2C), 123.0, 118.2, 113.1, 111.4, 69.6, 66.9, 55.3, 34.4, 28.0, 25.2;

HRESIMS (positive ion mode) *m/z* 341.1507 $[M + Na]^+$ (calcd. for $C_{22}H_{22}O_2Na$, 341.1512);

$[\alpha]_D^{25}$ = -28.2 (*c* = 0.12 g/100mL, MeOH);

HPLC conditions: Chiralpak IE column (*n*-Hexane:2-Propanol = 90:10, 0.5 ml/min, 254nm); tr (minor) = 20.8 min, tr (major) = 27.1 min; 98% *ee*.

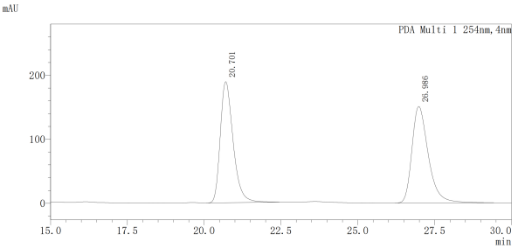

<Peak Table>
PDA Ch1 254nm

No.	Ret.Time(min)	Height	Height%	Area	Area%
1	20.701	189269	55.641	5504668	49.573
2	26.986	150890	44.359	5599530	50.427
Total		340159	100.000	11104198	100.000

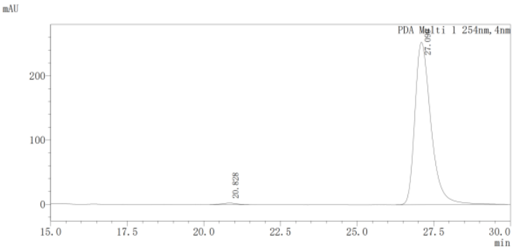

<Peak Table>
PDA Ch1 254nm

No.	Ret.Time(min)	Height	Height%	Area	Area%
1	20.828	2485	0.973	78002	0.838
2	27.099	252844	99.027	9234443	99.162
Total		255330	100.000	9312445	100.000

6.34 (4a*R*,8a*S*)-3-phenyl-7-(2-methoxyphenyl)-4a,5,6,8a-tetrahydro-2*H*-chromene (3bj)

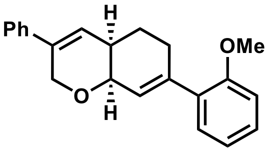

Isolated **3bj** in 57% yield, as white solid (m.p.: 119.0 °C).

^{1}H NMR (400 MHz, $CDCl_3$) δ 7.41 – 7.34 (overlapped, 4H), 7.33 – 7.22 (overlapped, 3H), 6.96 (t, *J* = 7.4 Hz, 1H), 6.91 (d, *J* = 8.2 Hz, 1H), 6.23 (d, *J* = 4.9 Hz, 1H), 5.96 (br s, 1H), 4.63 (m, 2H), 4.23 (t, *J* = 4.7 Hz, 1H), 3.83 (s, 3H), 2.51 (m, 1H), 2.41 (m, 1H), 1.90 (m, 1H), 1.85 – 1.68 (overlapped, 2H);

^{13}C NMR (100 MHz, $CDCl_3$) δ 156.7, 143.6, 138.4, 136.1, 132.4, 129.5, 128.52 (2C), 128.47, 127.5, 125.9, 125.0 (2C), 124.6, 120.6, 110.9, 69.6, 66.7, 55.4, 34.5, 29.2, 25.4;

HRESIMS (positive ion mode) *m/z* 341.1510 $[M + Na]^+$ (calcd. for $C_{22}H_{22}O_2Na$, 341.1512);

$[\alpha]_D^{25}$ = -108.8 (*c* = 0.13 g/100mL, acetone);

HPLC conditions: Chiralpak ID column (*n*-Hexane:2-Propanol = 90:10, 0.5 ml/min, 254nm); tr (minor) = 13.2 min, tr (major) = 14.6 min; 90% *ee*.

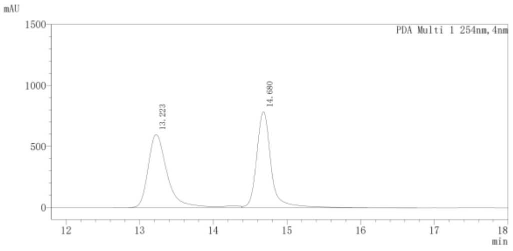

<Peak Table>
PDA Ch1 254nm

No.	Ret. Time(min)	Height	Height%	Area	Area%
1	13.223	599378	43.241	10897587	50.452
2	14.680	786756	56.759	10702391	49.548
Total		1386135	100.000	21599978	100.000

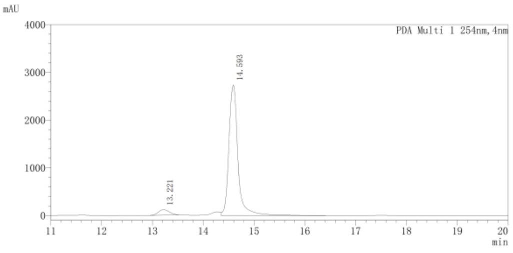

<Peak Table>
PDA Ch1 254nm

No.	Ret. Time(min)	Height	Height%	Area	Area%
1	13.221	113653	3.986	1724827	4.858
2	14.593	2737483	96.014	33779476	95.142
Total		2851137	100.000	35504303	100.000

6.35 (4a*R*,8a*S*)-3-phenyl-7-(naphthalen-1-yl)-4a,5,6,8a-tetrahydro-2*H*-chromene (3bk)

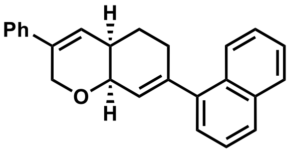

Isolated **3bk** in 52% yield, as white solid (m.p.: 82.1 °C).

^{1}H NMR (400 MHz, $CDCl_3$) δ 7.98 (d, *J* = 7.6 Hz, 1H), 7.87 (d, *J* = 8.0 Hz, 1H), 7.80 (d, *J* = 8.1 Hz, 1H), 7.51 – 7.38 (overlapped, 7H), 7.35 – 7.31 (overlapped, 2H), 6.27 (br s, 1H), 5.98 (br s, 1H), 4.67 (m, 2H), 4.37 (t, *J* = 4.4 Hz, 1H), 2.59 – 2.43 (overlapped, 3H), 2.04 – 1.91 (overlapped, 2H);

^{13}C NMR (100 MHz, $CDCl_3$) δ 143.8, 141.5, 138.3, 136.4, 133.7, 131.0, 128.6 (2C), 128.3, 127.6, 127.3, 126.1, 125.9, 125.7, 125.62, 125.60, 125.4, 125.0 (2C), 124.7, 69.6, 66.5, 34.3, 31.1, 25.5;

HRESIMS (positive ion mode) *m/z* 361.1564 $[M + Na]^+$ (calcd. for $C_{25}H_{22}ONa$, 361.1563);

$[\alpha]_D^{25}$ = -44.9 (*c* = 0.15 g/100mL, MeOH);

HPLC conditions: Chiralpak IAcolumn (*n*-Hexane:2-Propanol = 97:3, 0.5 ml/min, 254nm); tr (minor) = 15.5 min, tr (major) = 19.8 min; 86% *ee*.

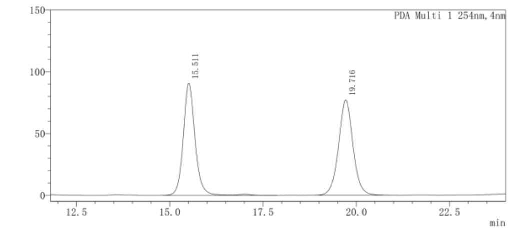

<Peak Table>
PDA Ch1 254nm

No.	Ret. Time(min)	Height	Height%	Area	Area%
1	15.511	90807	54.051	2014670	49.131
2	19.716	77194	45.949	2085978	50.869
Total		168001	100.000	4100648	100.000

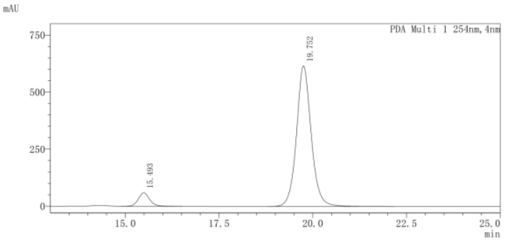

<Peak Table>
PDA Ch1 254nm

No.	Ret. Time(min)	Height	Height%	Area	Area%
1	15.493	59603	8.816	1268701	7.011
2	19.752	616467	91.184	16827297	92.989
Total		676070	100.000	18095998	100.000

6.36 (4a*R*,8a*S*)-3-phenyl-7-(naphthalen-2-yl)-4a,5,6,8a-tetrahydro-2*H*-chromene (3bl)

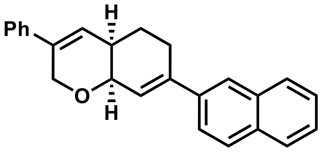

Isolated **3bl** in 71% yield, as white solid (m.p.: 171.0 °C).

^{1}H NMR (400 MHz, $CDCl_3$) δ 7.89 – 7.79 (overlapped, 4H), 7.65 (d, J = 8.7 Hz, 1H), 7.54 – 7.26 (overlapped, 7H), 6.39 (br s, 1H), 6.24 (br s, 1H), 4.63 (q, J = 15.6 Hz, 2H), 4.29 (br s, 1H), 2.73 (m, 1H), 2.60 (m, 1H), 2.39 (m, 1H), 2.00 (m 1H), 1.85 (m, 1H);

^{13}C NMR (100 MHz, $CDCl_3$) δ 142.5, 138.6, 138.2, 136.2, 133.4, 132.9, 128.6 (2C), 128.2, 127.8, 127.6, 127.5, 126.2, 125.9, 125.5, 125.0 (2C), 124.4, 124.0, 123.3, 69.7, 66.9, 34.4, 27.9, 25.3;

HRESIMS (positive ion mode) m/z 361.1559 [M + Na]$^+$ (calcd. for $C_{25}H_{22}ONa$, 361.1563);

[α]$_D^{25}$ = -89.4 (c = 0.10 g/100mL, acetone);

HPLC conditions: Chiralpak IC column (n-Hexane:2-Propanol = 90:10, 0.5 ml/min, 254nm); tr (minor) = 16.7 min, tr (major) = 24.9 min; 96% *ee*.

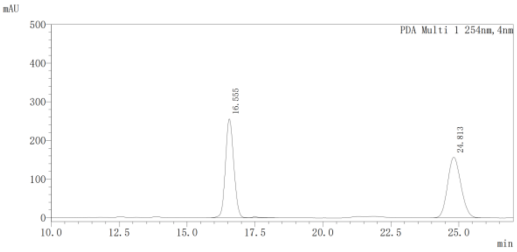

<Peak Table>
PDA Ch1 254nm

No.	Ret.Time(min)	Height	Height%	Area	Area%
1	16.555	256156	61.973	5516793	50.716
2	24.813	157181	38.027	5360947	49.284
Total		413337	100.000	10877740	100.000

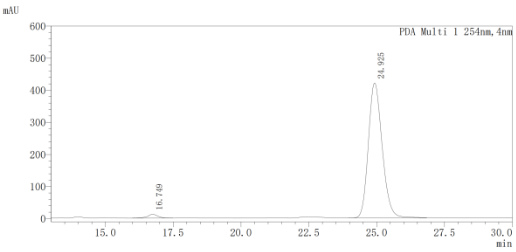

<Peak Table>
PDA Ch1 254nm

No.	Ret.Time(min)	Height	Height%	Area	Area%
1	16.749	12014	2.777	321693	2.110
2	24.925	420571	97.223	14926636	97.890
Total		432584	100.000	15248329	100.000

6.37 (4a*R*,8a*S*)-3-phenyl-7-(benzo[d][1,3]dioxol-5-yl)-4a,5,6,8a-tetrahydro-2*H*-chromene (3bm)

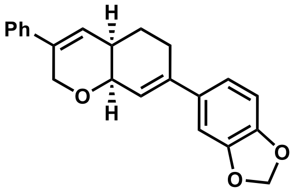

Isolated **3bm** in 94% yield, as white solid (m.p.: 135.0 °C).

^{1}H NMR (400 MHz, $CDCl_3$) δ 7.33 (overlapped, 4H), 7.25 (m, 1H), 6.93 (overlapped, 2H), 6.77 (d, J = 8.1 Hz, 1H), 6.19 (d, J = 4.7 Hz, 1H), 6.11 (d, J = 3.0 Hz, 1H), 5.95 (s, 2H), 4.58 (qt, J = 15.6, 1.9 Hz, 2H), 4.20 (t, J = 4.6 Hz, 1H), 2.51 (dt, J = 17.3, 4.2 Hz, 1H), 2.40 (m, 1H), 2.30 (m, 1H), 1.91 (m, 1H), 1.75 (m, 1H);

^{13}C NMR (100 MHz, $CDCl_3$) δ 147.7, 147.1, 142.1, 138.2, 136.1, 135.8, 128.5 (2C), 127.5, 125.4, 124.9 (2C), 121.7, 119.1, 108.0, 106.1, 101.0, 69.6, 66.8, 34.3, 28.0, 25.2;

HRESIMS (positive ion mode) m/z 355.1304 [M + Na]$^+$ (calcd. for $C_{22}H_{20}O_3Na$, 355.1305);

[α]$_D^{25}$ = -42.3 (c = 0.10 g/100mL, MeOH);

HPLC conditions: Chiralpak IE column (n-Hexane:2-Propanol = 85:15, 0.5 ml/min, 254nm); tr (minor) = 25.6 min, tr (major) = 26.7 min; 91% *ee*.

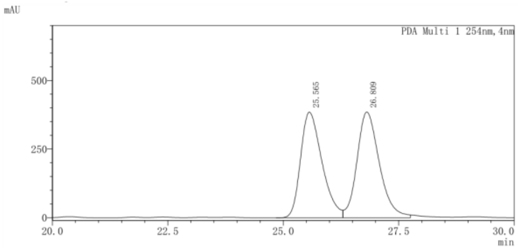

<Peak Table>
PDA Ch1 254nm

No.	Ret.Time(min)	Height	Height%	Area	Area%
1	25.565	385186	49.987	12439782	48.918
2	26.809	385385	50.013	12990091	51.082
Total		770571	100.000	25429873	100.000

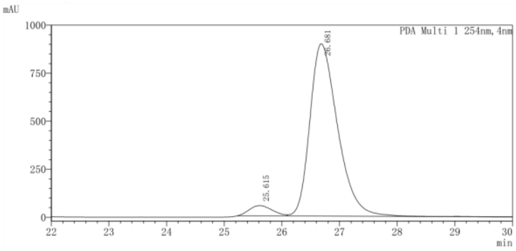

<Peak Table>
PDA Ch1 254nm

No.	Ret.Time(min)	Height	Height%	Area	Area%
1	25.615	53864	5.659	1494373	4.724
2	26.681	898005	94.341	30140812	95.276
Total		951869	100.000	31635186	100.000

6.38 (4a*R*,8a*S*)-7-(benzo[*b*]thiophen-6-yl)-3-phenyl-4a,5,6,8a-tetrahydro-2*H*-chromene (3bn)

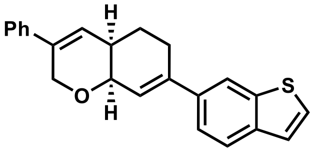

Isolated **3bn** in 76% yield, as white solid (m.p.: 173.4 °C).

^{1}H NMR (400 MHz, $CDCl_3$) δ 7.88 (d, J = 1.7 Hz, 1H), 7.83 (d, J = 8.4 Hz, 1H), 7.47 (dd, J = 8.5, 1.9 Hz, 1H), 7.44 (d, J = 5.5 Hz, 1H), 7.38 – 7.30 (overlapped, 5H), 7.29 (m, 1H), 6.28 (d, J = 3.5 Hz, 1H), 6.22 (d, J = 5.0 Hz, 1H), 4.62 (m, 2H), 4.25 (d, J = 4.7 Hz, 1H), 2.66 (m, 1H), 2.53 (m, 1H), 2.37 (m, 1H), 1.97 (m, 1H), 1.81 (m, 1H);

^{13}C NMR (100 MHz, $CDCl_3$) δ 142.8, 139.8, 138.9, 138.2, 137.9, 136.2, 128.5 (2C), 127.5, 126.8, 125.5 (2C), 125.0, 124.1, 122.8, 122.4, 122.2, 120.5, 69.7, 66.9, 34.4, 28.2, 25.3;

HRESIMS (positive ion mode) m/z 367.1123 $[M + Na]^+$ (calcd. for $C_{23}H_{20}OSNa$, 367.1127);

[α]$_D^{23}$ = -71.1 (c = 0.09 g/100mL, acetone);

HPLC conditions: Chiralpak IC column (n-Hexane:2-Propanol = 90:10, 0.5 ml/min, 254nm); tr (minor) = 19.3 min, tr (major) = 26.4 min; 97% *ee*.

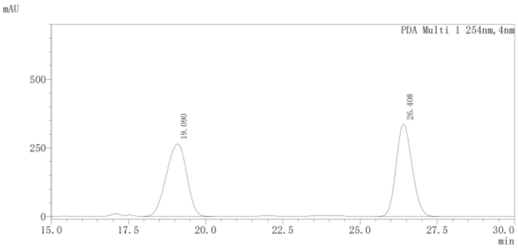

<Peak Table>
PDA Ch1 254nm

No.	Ret.Time(min)	Height	Height%	Area	Area%
1	19.090	264394	43.977	12210772	50.136
2	26.408	336814	56.023	12144437	49.864
Total		601207	100.000	24355209	100.000

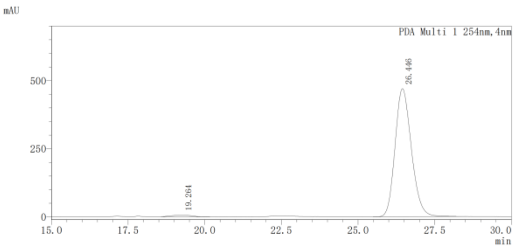

<Peak Table>
PDA Ch1 254nm

No.	Ret.Time(min)	Height	Height%	Area	Area%
1	19.264	6828	1.432	288652	1.629
2	26.446	469960	98.568	17434370	98.371
Total		476787	100.000	17723022	100.000

6.39 (4a*R*,8a*S*)-3-phenyl-7-(thiophen-3-yl)-4a,5,6,8a-tetrahydro-2*H*-chromene (3bo)

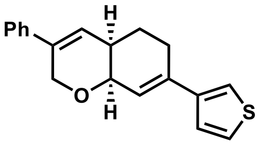

Isolated **3bo** in 66% yield, as white solid (m.p.: 47.2 °C).

^{1}H NMR (400 MHz, $CDCl_3$) δ 7.37 – 7.26 (m, 8H), 6.30 (d, J = 4.9 Hz, 1H), 6.22 (d, J = 5.0 Hz, 1H), 4.62 (q, J = 15.6 Hz, 2H), 4.26 (d, J = 4.6 Hz, 1H), 2.62 (m, 1H), 2.45 (m, 1H), 2.35 (m, 1H), 1.94 (m, 1H), 1.81 (m, 1H);

^{13}C NMR (100 MHz, $CDCl_3$) δ 142.9, 138.3, 137.5, 136.2, 128.7 (2C), 127.6, 125.7, 125.6, 125.2, 125.1 (2C), 121.7, 120.4, 69.8, 66.9, 34.5, 27.7, 25.1;

HRESIMS (negative ion mode) *m/z* 293.1000 [M - H]$^-$ (calcd. for $C_{19}H_{17}OS$, 293.1006);

[α]$_D$^{20} = -43.4 (c = 0.10 g/100mL, acetone);

HPLC conditions: Chiralpak IC column (*n*-Hexane:2-Propanol = 90:10, 0.5 ml/min, 254nm); tr (minor) = 17.7 min, tr (major) = 19.5 min; 83% *ee*.

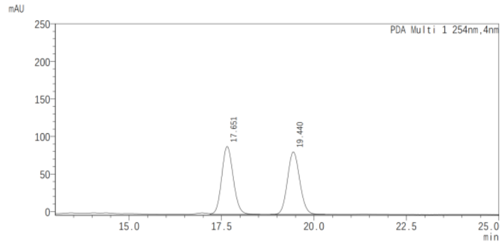

<Peak Table>
PDA Ch1 254nm

No.	Ret.Time(min)	Height	Height%	Area	Area%
1	17.651	90276	52.056	1959799	50.525
2	19.440	83146	47.944	1919048	49.475
Total		173422	100.000	3878847	100.000

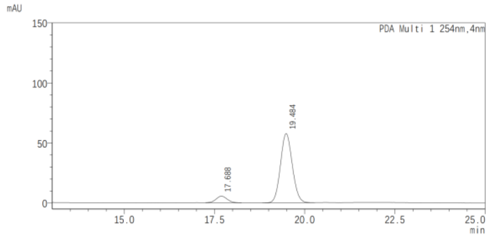

<Peak Table>
PDA Ch1 254nm

No.	Ret.Time(min)	Height	Height%	Area	Area%
1	17.688	5533	8.751	120558	8.378
2	19.484	57693	91.249	1318419	91.622
Total		63226	100.000	1438977	100.000

6.40 (4a*R*,8a*S*)-3-phenyl-7-vinyl-4a,5,6,8a-tetrahydro-2*H*-chromene (3bp)

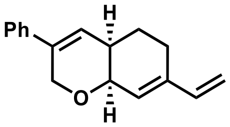

Isolated **3bp** in 76% yield, as white solid (m.p.: 71.0 °C).

^{1}H NMR (400 MHz, $CDCl_3$) δ 7.43 – 7.27 (m, 5H), 6.46 (dd, J = 17.5, 10.8 Hz, 1H), 6.20 (d, J = 5.0 Hz, 1H), 5.89 (d, J = 4.8 Hz, 1H), 5.30 (d, J = 17.5 Hz, 1H), 5.12 (d, J = 10.7 Hz, 1H), 4.59 (m, 2H), 4.17 (t, J = 4.5 Hz, 1H), 2.42 (dt, J = 17.1, 4.2 Hz, 1H), 2.31 (m, 1H), 2.13 (m, 1H), 1.88 (m, 1H), 1.70 (m, 1H);

^{13}C NMR (100 MHz, $CDCl_3$) δ 141.0, 139.2, 138.2, 136.1, 128.5 (2C), 127.5, 126.7, 125.5, 124.9 (2C), 113.5, 69.6, 66.8, 34.7, 24.6, 23.9;

HRESIMS (positive ion mode) *m/z* 261.1246 [M + Na]$^+$ (calcd. for $C_{17}H_{18}ONa$, 261.1250);

[α]$_D$^{26} = -156.0 (c = 0.11 g/100mL, MeOH);

HPLC conditions: Chiralpak IE column (*n*-Hexane:2-Propanol = 90:10, 0.5 ml/min, 254nm); tr (minor) = 11.2 min, tr (major) = 12.0 min; 91% *ee*.

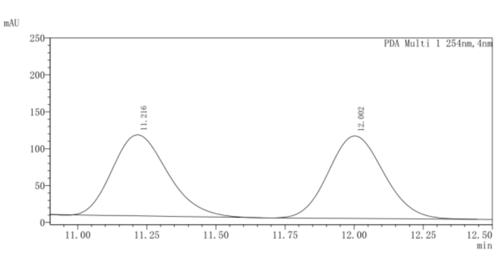

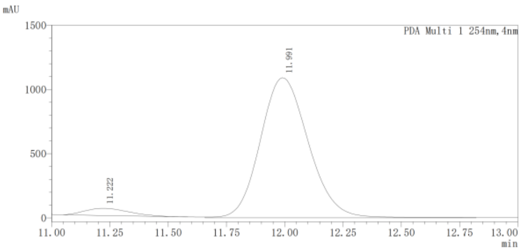

<Peak Table>
PDA Ch1 254nm

No.	Ret.Time(min)	Height	Height%	Area	Area%
1	11.216	109653	49.493	1529564	49.394
2	12.002	111898	50.507	1567066	50.606
Total		221551	100.000	3096630	100.000

<Peak Table>
PDA Ch1 254nm

No.	Ret.Time(min)	Height	Height%	Area	Area%
1	11.222	55611	4.848	726471	4.417
2	11.991	1091427	95.152	15720628	95.583
Total		1147038	100.000	16447099	100.000

6.41 (4a*R*,8a*S*)-3-phenyl-7-(phenylethynyl)-4a,5,6,8a-tetrahydro-2*H*-chromene (3bq)

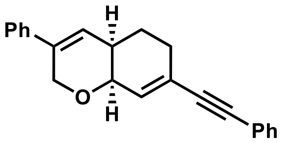

Isolated **3bq** in 55% yield, as white solid (m.p.: 126.0 °C).

^{1}H NMR (400 MHz, $CDCl_3$) δ 7.52 – 7.45 (overlapped, 2H), 7.40 – 7.30 (overlapped, 8H), 6.30 (d, J = 4.8 Hz, 1H), 6.17 (d, J = 4.7 Hz, 1H), 4.66 – 4.52 (m, 2H), 4.19 (t, J = 4.7 Hz, 1H), 2.41 (m, 1H), 2.36 – 2.26 (m, 2H), 1.86 (m, 1H), 1.73 (m, 1H);

^{13}C NMR (100 MHz, $CDCl_3$) δ 138.1, 136.3, 131.9, 131.6 (2C), 128.5 (2C), 128.3 (2C), 128.2, 127.6, 126.5, 125.1, 124.9 (2C), 123.2, 89.9, 89.5, 68.9, 66.6, 33.9, 29.5, 24.7;

HRESIMS (positive ion mode) *m/z* 313.1589 $[M + H]^+$ (calcd. for $C_{23}H_{21}O$, 313.1587);

$[\alpha]_D^{25}$ = -93.1 (c = 0.14 g/100mL, acetone);

HPLC conditions: Chiralpak IE column (*n*-Hexane:2-Propanol = 90:10, 0.5 ml/min, 254nm); tr (minor) = 12.1 min, tr (major) = 14.8 min; 84% *ee*.

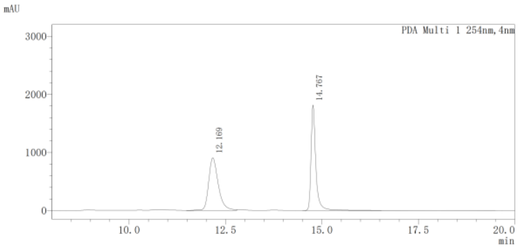

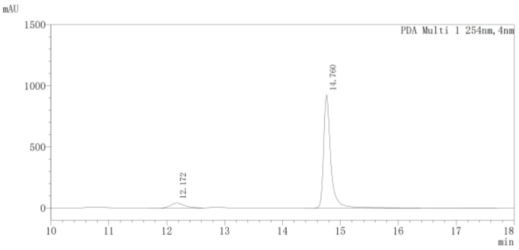

<Peak Table>
PDA Ch1 254nm

No.	Ret.Time(min)	Height	Height%	Area	Area%
1	12.169	910069	33.394	15901137	49.795
2	14.767	1815218	66.606	16031959	50.205
Total		2725287	100.000	31933096	100.000

<Peak Table>
PDA Ch1 254nm

No.	Ret.Time(min)	Height	Height%	Area	Area%
1	12.172	40594	4.202	696891	8.006
2	14.760	925384	95.798	8007894	91.994
Total		965978	100.000	8704785	100.000

6.42 (4a*R*,8a*S*)-3-phenyl-7-butyl-4a,5,6,8a-tetrahydro-2*H*-chromene (3br)

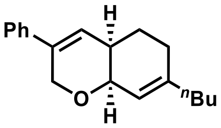

Isolated **3br** in 67% yield, as colorless oil.

^{1}H NMR (400 MHz, $CDCl_3$) δ 7.35 (overlapped, 4H), 7.32 – 7.28 (m, 1H), 6.18 (d, J = 5.1 Hz, 1H), 5.63 (d, J = 4.8 Hz, 1H), 4.57 (m, 2H), 4.03 (t, J = 4.4 Hz, 1H), 2.21 (m, 1H), 2.12 – 2.02 (overlapped, 4H), 1.79 (m, 1H), 1.64 (m, 1H), 1.45 (m, 2H), 1.34 (m, 2H), 0.93 (t, J = 7.2 Hz, 3H);

^{13}C NMR (100 MHz, $CDCl_3$) δ 145.1, 138.3, 135.9, 128.5 (2C), 127.4, 126.0, 124.9 (2C), 119.8, 69.6, 66.8, 37.4, 34.6, 29.7, 28.8, 25.1, 22.5, 14.0;

HRESIMS (positive ion mode) *m/z* 291.1714 $[M + Na]^+$ (calcd. for $C_{19}H_{24}ONa$, 291.1719);

$[\alpha]_D^{25}$ = -66.3 (c = 0.10 g/100mL, acetone);

HPLC conditions: Chiralpak IE column (n-Hexane:2-Propanol = 90:10, 0.5 ml/min, 254nm); tr (minor) = 9.7 min, tr (major) = 11.3 min; 94% *ee*.

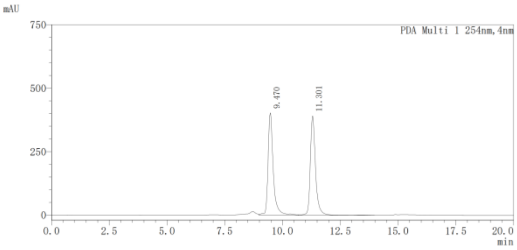

<Peak Table>
PDA Ch1 254nm

No.	Ret.Time(min)	Height	Height%	Area	Area%
1	9.470	402999	50.759	6382318	50.668
2	11.301	390954	49.241	6214078	49.332
Total		793953	100.000	12596396	100.000

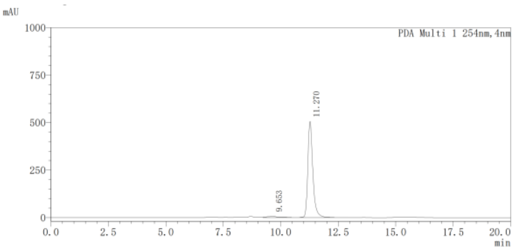

<Peak Table>
PDA Ch1 254nm

No.	Ret.Time(min)	Height	Height%	Area	Area%
1	9.653	7589	1.478	262008	3.281
2	11.270	505892	98.522	7724501	96.719
Total		513481	100.000	7986509	100.000

6.43 (4a*R*,8a*S*)-3-phenyl-4a,5,6,8a-tetrahydro-2*H*-chromene (3bs)

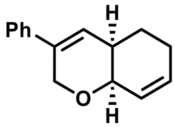

Isolated **3bs** in 94% yield, as white solid (m.p.: 44.4 °C).

^{1}H NMR (400 MHz, $CDCl_3$) δ 7.36 (overlapped, 4H), 7.30 (m, 1H), 6.19 (d, J = 4.6 Hz, 1H), 6.08 (m, 1H), 5.91 (m, 1H), 4.58 (m, 2H), 4.05 (d, J = 4.5 Hz, 1H), 2.28 (m, 1H), 2.20 (m, 1H), 2.08 (m, 1H), 1.79 (m, 1H), 1.64 (m, 1H);

^{13}C NMR (100 MHz, $CDCl_3$) δ 138.3, 135.9, 133.0, 128.5 (2C), 127.5, 126.0, 125.9, 124.9 (2C), 68.9, 66.8, 34.7, 25.3, 24.6;

HRESIMS (positive ion mode) *m/z* 235.1095 $[M + Na]^+$ (calcd. for $C_{15}H_{16}ONa$, 235.1093);

$[\alpha]_D^{25}$ = -103.8 (c = 0.17 g/100mL, acetone);

HPLC conditions: Chiralpak IA column (n-Hexane:2-Propanol = 97:3, 0.5 ml/min, 254nm); tr (minor) = 11.8 min, tr (major) = 13.4min; 93% *ee.*

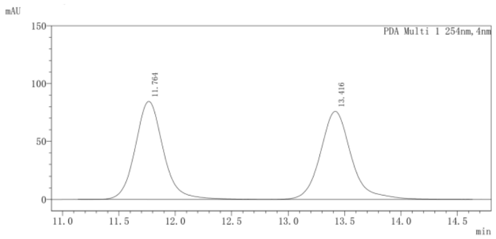

<Peak Table>
PDA Ch1 254nm

No.	Ret.Time(min)	Height	Height%	Area	Area%
1	11.764	84642	52.701	1459732	50.220
2	13.416	75967	47.299	1446963	49.780
Total		160610	100.000	2906695	100.000

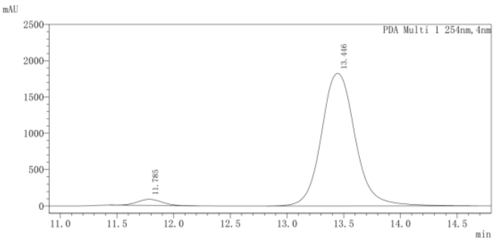

<Peak Table>
PDA Ch1 254nm

No.	Ret.Time(min)	Height	Height%	Area	Area%
1	11.785	84037	4.410	1270022	3.253
2	13.446	1821700	95.590	37777114	96.747
Total		1905736	100.000	39047137	100.000

6.44 (4a*R*,8a*S*)-7-(4-(((1*R*,2*S*,5*R*)-2-isopropyl-5-methylcyclohexyl)oxy)phenyl)-3-phenyl-4a,5,6,8a-tetrahydro-2*H*-chromene (3bt)

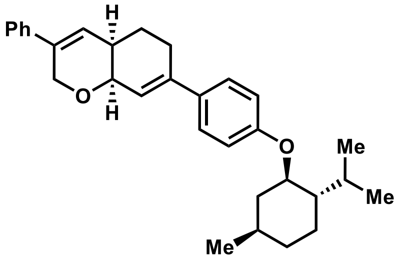

Isolated **3bt** in 58% yield, as white solid (m.p.: 153.7 °C).

^{1}H NMR (400 MHz, $CDCl_3$) δ 7.52 – 7.23 (overlapped, 7H), 6.89 (d, *J* = 8.3 Hz, 2H), 6.22 (d, *J* = 4.9 Hz, 1H), 6.17 (d, *J* = 4.8 Hz, 1H), 4.62 (q, *J* = 15.6 Hz, 2H), 4.25 (t, *J* = 4.5 Hz, 1H), 4.06 (td, *J* = 10.5, 4.2 Hz, 1H), 2.59 (dt, *J* = 17.3, 4.2 Hz, 1H), 2.44 (m, 1H), 2.35 (m, 1H), 2.30 – 2.12 (overlapped, 2H), 1.95 (dq, J = 12.7, 3.9 Hz, 1H), 1.86 – 1.70 (overlapped, 3H), 1.59 – 1.42 (overlapped, 2H), 1.20 – 0.88 (overlapped, 9H), 0.81 (d, J = 7.0 Hz, 3H);

^{13}C NMR (400 MHz, $CDCl_3$) δ 158.1, 142.0, 138.3, 136.1, 133.5, 128.5 (2C), 127.5, 126.7 (2C), 125.6, 124.9 (2C), 120.9, 115.5 (2C), 77.5, 69.8, 66.7, 48.1, 40.4, 34.5, 34.4, 31.5, 27.8, 26.1, 25.3, 23.8, 22.2, 20.8, 16.6;

HRESIMS (positive ion mode) *m/z* 465.2769 $[M + Na]^+$ (calcd. for $C_{31}H_{38}O_2Na$, 465.2764);

$[\alpha]_D^{21}$ = -37.8 (*c* = 0.13 g/100mL, acetone);

6.45 (4a*R*,8a*S*)-3-phenyl-7-(4-(((*S*)-4-(prop-1-en-2-yl)cyclohex-1-en-1-yl)methoxy)phenyl)-4a,5,6, 8a-tetrahydro-2*H*-chromene (3bu)

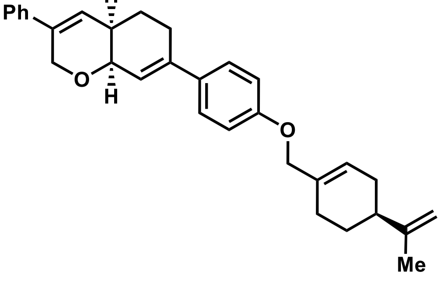

Isolated **3bu** in 44% yield, as white solid (m.p.: 167.0 °C).

^{1}H NMR (400 MHz, $CDCl_3$) δ 7.47 – 7.28 (overlapped, 7H), 6.92 (d, J = 8.3 Hz, 2H), 6.23 (d, J = 4.9 Hz, 1H), 6.19 (d, J = 4.9 Hz, 1H), 5.88 (s, 1H), 4.77 (d, J = 4.9 Hz, 2H), 4.63 (q, J = 15.6 Hz, 2H), 4.43 (s, 2H), 4.25 (t, J = 4.6 Hz, 1H), 2.60 (m, 1H), 2.45 (m, 1H), 2.35 (m, 1H), 2.23 (overlapped, 4H), 2.05 (m, 1H), 1.94 (overlapped, 2H), 1.79 (overlapped, 4H), 1.57 (m, 1H);

^{13}C NMR (100 MHz, $CDCl_3$) δ 158.7, 149.7, 142.0, 138.2, 136.1, 133.8, 133.5, 128.5 (2C), 127.5, 126.6 (2C), 125.6, 125.2, 124.9 (2C), 121.1, 114.5 (2C), 108.8, 72.4, 69.8, 66.8, 41.0, 34.4, 30.5, 27.8, 27.4, 26.4, 25.3, 20.8;

HRESIMS (positive ion mode) *m/z* 461.2454 $[M + Na]^+$ (calcd. for $C_{31}H_{34}O_2Na$, 461.2451);

$[\alpha]_D^{22}$ = -42.2 (c = 0.11 g/100mL, acetone).

6.46 (*S*,*E*)-5-phenyl-2-styryl-3,6-dihydro-2*H*-pyran (3aa')

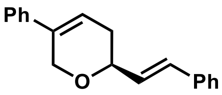

Isolated **3aa'** in 93% yield, as white solid (m.p.: 134.0 °C).

^{1}H NMR (400 MHz, $CDCl_3$) δ 7.45 (overlapped, 2H), 7.41 – 7.25 (overlapped, 8H), 6.82 – 6.67 (m, 1H), 6.36 (dd, J = 16.0, 5.9 Hz, 1H), 6.25 (m, 1H), 4.80 – 4.59 (m, 2H), 4.38 – 4.23 (m, 1H), 2.53 – 2.33 (m, 2H);

^{13}C NMR (100 MHz, $CDCl_3$) δ 13C NMR (101 MHz, CDCl3) δ 138.2, 136.8, 136.0, 130.9, 129.6, 128.6 (4C), 127.7, 127.5, 126.5 (2C), 124.9 (2C), 120.8, 73.8, 67.1, 31.4;

HRESIMS (positive ion mode) *m/z* 262.1350 $[M]^+$ (calcd. for $C_{19}H_{18}O^+$, 262.1352);

$[\alpha]_D^{25}$ = 5.6 (c = 0.18 g/100mL, acetone);

HPLC conditions: Chiralpak IE column (*n*-Hexane:2-Propanol = 90:10, 0.5 ml/min, 254nm); tr (minor) = 12.4 min, tr (major) = 14.3 min; 6% *ee*.

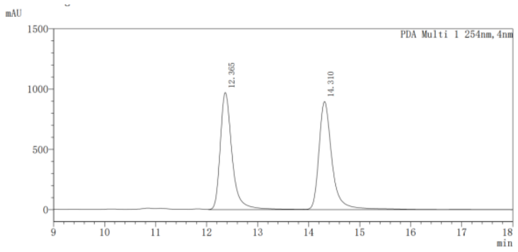

<Peak Table>

PDA Ch1 254nm

No.	Ret.Time(min)	Height	Height%	Area	Area%
1	12.365	971241	51.989	15752225	49.313
2	14.310	896920	48.011	16191095	50.687
Total		1868161	100.000	31943320	100.000

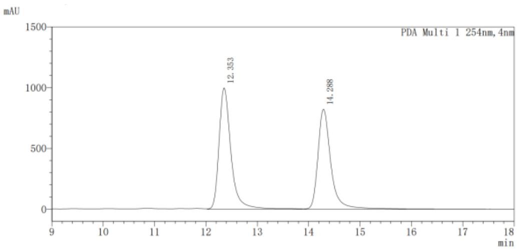

<Peak Table>

PDA Ch1 254nm

No.	Ret.Time(min)	Height	Height%	Area	Area%
1	12.353	998366	54.842	16178634	52.739
2	14.288	822078	45.158	14498400	47.261
Total		1820444	100.000	30677034	100.000

6.47 (4aR,7aR)-3-phenyl-2,4a,5,7a-tetrahydrocyclopenta[b]pyran (S-3e)

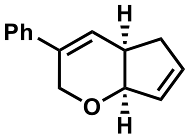

Isolated **S-3e** in 6% yield.

^{1}H NMR (400 MHz, $CDCl_3$) δ 1H NMR (400 MHz, CDCl3) δ 7.37 (d, J = 7.2 Hz, 2H), 7.32 (t, J = 7.2 Hz, 2H), 7.21 (t, J = 7.2 Hz, 1H), 6.09 (d, J = 8.4 Hz, 1H), 5.81-5.83 (m, 1H), 5.54-5.56 (m, 1H), 4.76 (dd, J = 3.2, 7.2 Hz, 1H), 2.77 (d, J = 8.8 Hz, 1H), 2.61-2.69 (m, 2H), 2.14-2.21 (m, 2H)

^{13}C NMR (100 MHz, $CDCl_3$) δ144.2, 135.2, 133.6, 131.1, 128.2, 127.1, 126.5, 125.4, 78.4, 52.2, 39.4, 37.2.

HPLC conditions: Chiralpak IC column (*n*-Hexane:2-Propanol = 98:2, 0.5 ml/min, 254nm); tr (minor) = 13.0 min, tr (major) = 14.1 min; 6% *ee.*

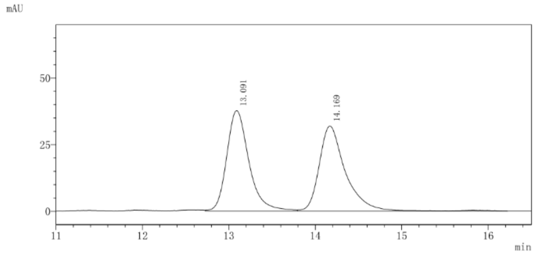

<Peak Table>
PDA Ch1 254nm

No.	Ret.Time(min)	Height	Height%	Area	Area%
1	13.091	37662	54.150	672564	49.979
2	14.169	31890	45.850	673125	50.021
Total		69552	100.000	1345689	100.000

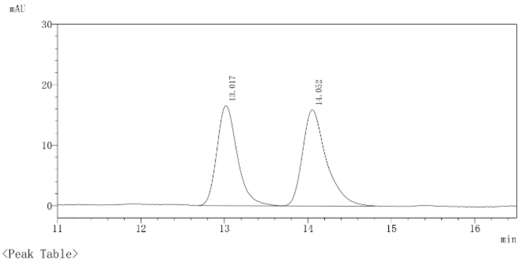

<Peak Table>
PDA Ch1 254nm

No.	Ret.Time(min)	Height	Height%	Area	Area%
1	13.017	16547	51.013	280838	47.138
2	14.052	15890	48.987	314939	52.862
Total		32437	100.000	595778	100.000

(*Z*)-3-Iodoprop-2-en-1-ol

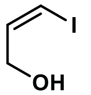

^{1}H NMR (400 MHz, $CDCl_3$) δ 6.48 (dt, *J* = 7.6, 5.6 Hz, 1H), 6.36 (d, *J* = 7.6 Hz, 1H), 4.25 (t, *J* = 5.6 Hz, 2H), 1.71 (br d, 1H).

^{13}C NMR (100 MHz, $CDCl_3$) δ 140.0, 82.7, 65.7. The data are in agreement with those previously reported in the literature[4].

N-Cbz-1,2-dihydropyridine.

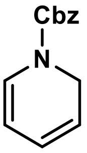

^{1}H NMR (400 MHz, $CDCl_3$) δ 7.36-7.33 (m, 5H), 7.31-7.25 (m, 1H), 6.79-6.68 (m, 1H), 5.84-5.79 (m, 1H), 5.54-5.42 (m, 1H), 5.19(s, 2H), 4.39-4.37(m,2H)

^{13}C NMR (100 MHz, $CDCl_3$) δ 149.4, 136.0, 128.6, 128.3, 128.1, 125.6, 121.9, 119.2, 105.0, 67.8, 43.6. The data are in agreement with those previously reported in the literature[5]

7. Gram-scale synthesis.

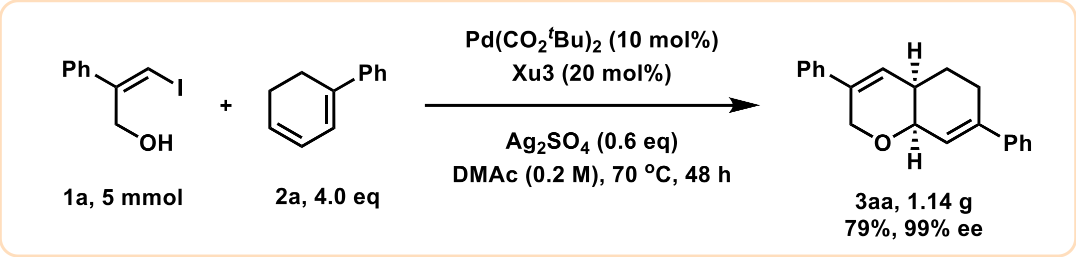

To a sealed tube was added Palladium pivalate (155 mg, 10 mol%, *Bide*) and **Xu3** (684 mg, 20 mol%) in 25 mL dry DMAc and stirred at room temperature for 1 h under argon atmosphere. Then, **1a** (5 mmol), **2a** (20 mmol, 4.0 equiv) and Ag_2SO_4 (935 mg, 0.6 equiv) were added to the tube under argon atmosphere, and stirred at 70 °C for 48 h. After the reaction was complete (monitored by TLC), dilute with saturated salt water and EtOAc, then extracted with EA (twice), dried over anhydrous Na_2SO_4, the solvent was removed under reduced pressure. The crude product was purified by column chromatography (*n*-Hexane/EA, 50:1 to 30:1) to give 3aa as a white solid (1.14 g, 79 % yield, 99% ee).

HPLC conditions: Chiralpak ID column (*n*-Hexane:2-Propanol = 90:10, 1 ml/min, 254nm); tr (minor) = 18.5min, tr (major) = 16.8 min; 99% *ee*.

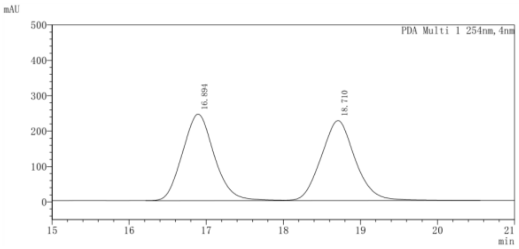

<Peak Table>

PDA Ch1 254nm

No.	Ret.Time(min)	Height	Height%	Area	Area%
1	16.894	244627	51.978	7020694	50.296
2	18.710	226012	48.022	6938149	49.704
Total		470640	100.000	13958843	100.000

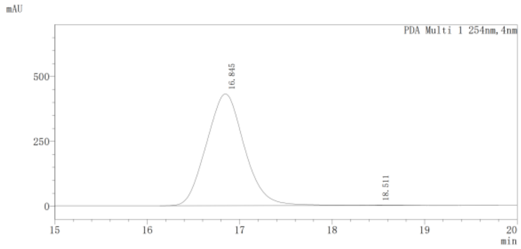

<Peak Table>

PDA Ch1 254nm

No.	Ret.Time(min)	Height	Height%	Area	Area%
1	16.845	430392	99.881	12359172	99.926
2	18.511	514	0.119	9092	0.074
Total		430906	100.000	12368264	100.000

8. Synthetic applications of the products.

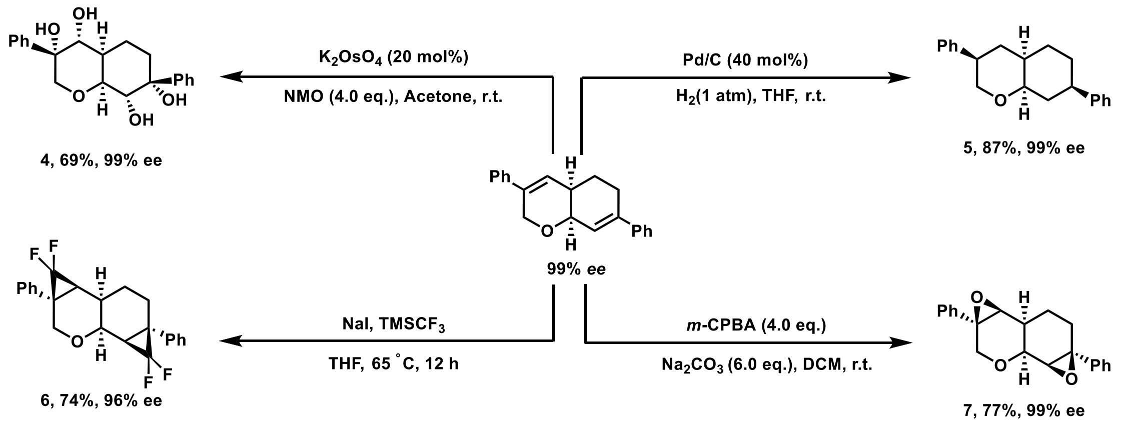

8.1 (3*S*,4*R*,4a*R*,7*R*,8*R*,8a*S*)-3,7-diphenyloctahydro-2*H*-chromene-3,4,7,8-tetraol (4)

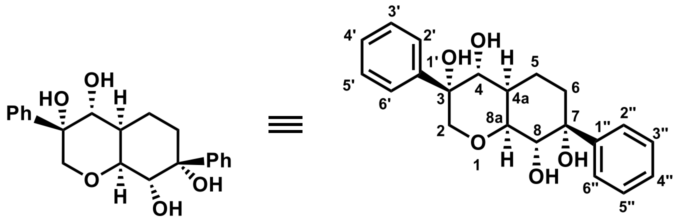

To a solution of **3aa** (99% ee, 0.2 mmol, 57.7 mg) and NMO (4.0 equiv., 93.8 mg, cas: 7529-22-8) in acetone (2 mL) was added $K_2OsO_4·2H_2O$ (20 mol%, 12.4 mg, cas: 10022-66-9) at r.t., then the resulting mixture was stirred for 12 h. Upon completion of the reaction, Na_2SO_3 (sat. aq) was added to the mixture. The mixture was extracted with DCM. The combined organics were washed with H_2O and brine, dried with $MgSO_4$, filtered, and concentrated on a rotary evaporator. The crude product was purified by column chromatography (PE/EA = 1:1) to give **4** (69 % yield, 49.2mg, 99% *ee*) as a white solid (m.p.: 69.0°C).

^{1}H NMR (400 MHz, $CDCl_3$) δ 7.44 – 7.41 (overlapped, 4H, H-2', H-6', H-2'' and H-6''), 7.34 – 7.31 (overlapped, 4H, H-3', H-5', H-3'' and H-5''), 7.27 – 7.21 (overlapped, 2H, H-4' and H-4''), 4.37 (d, J = 10.1 Hz, 1H, H-8), 4.09 (overlapped, 2H, H-4 and H-8a), 3.68 (d, J = 13.0 Hz, 1H, H_a-2), 3.41 (d, J = 13.0 Hz, 1H, H_b-2), 2.31 (m, 1H, H-4a), 1.85 (overlapped, 2H, H_2-5), 1.75 (m, 1H, H_a-6), 1.64 (m, 1H, H_b-6);

^{13}C NMR (100 MHz, $CDCl_3$) δ 145.9 (C-1''), 141.1 (C-1'), 128.5 (2C, C-3''/5''), 128.4 (2C, C-3'/5'), 127.8 (C-4'), 127.2 (C-4''), 125.8 (2C, C-2'/6'), 125.0 (2C, C-2''/6''), 77.2 (C-8a), 76.8 (C-7), 74.8 (C-3), 68.8 (C-2), 68.5 (C-4), 67.9 (C-8), 37.5 (C-4a), 33.4 (C-6), 19.2 (C-5);

HRESIMS (positive ion mode) *m/z* 379.1512 [M + Na]$^+$ (calcd. for $C_{21}H_{24}O_5Na$, 379.1516);

$[\alpha]_D^{22}$ = 29.1 (*c* = 0.09 g/100mL, acetone);

HPLC conditions: Chiralpak IF column (*n*-Hexane:2-Propanol = 50:50, 0.5 ml/min, 254nm); tr (minor) = 9.2 min, tr (major) =19.4 min; 99% *ee*.

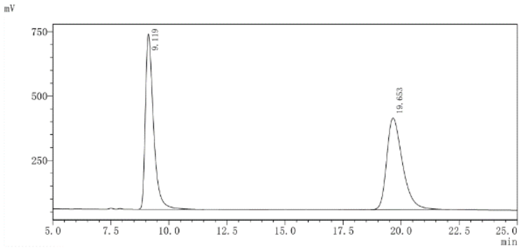

<Peak Table>

检测器A Ch2 210nm

No.	Ret.Time(min)	Height	Height%	Area	Area%
1	9.119	681108	65.668	16567163	49.543
2	19.653	356088	34.332	16872682	50.457
总计		1037197	100.000	33439844	100.000

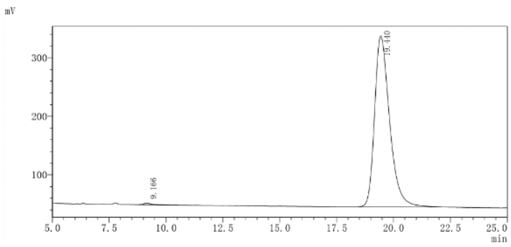

<Peak Table>

检测器A Ch2 210nm

No.	Ret.Time(min)	Height	Height%	Area	Area%
1	9.166	2589	0.879	68786	0.510
2	19.440	292092	99.121	13425206	99.490
总计		294682	100.000	13493992	100.000

8.2 (3*R*,4a*R*,7*R*,8a*R*)-3,7-diphenyloctahydro-2*H*-chromene (5)

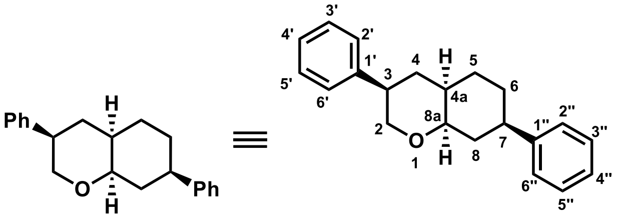

In a 10 mL Schlenk tube, after the solution of Pd/C (40 mol%, 10% on activated carbon, reduced, dry powder, cas: 7440-05-3, *j&k chemical*) and **3aa** (99% *ee*, 0.2 mmol, 57.7mg) in THF (2 mL) was stirred at room temperature for 8 h. The reaction was determined by TLC analysis, after the 3aa was consumed completely. Solvent was removed in a rotary evaporator, purified by flash column chromatography on silica gel (PE/EA= 10:1) to afford the desired product **5** (87% yield, 99% *ee*, 50.9 mg) as white solid (m.p.: 72.8°C).

^{1}H NMR (400 MHz, $CDCl_3$) δ 7.45 – 7.23 (overlapped, 10H, H-2' – H-6"), 4.12 (dt, J = 12.2, 4.8 Hz, 1H, H-8a), 3.78 (ddd, J = 11.7, 4.8, 1.9 Hz, 1H, H_a-2), 3.68 (t, J = 11.5 Hz, 1H, H_b-2), 3.00 (tt, J = 11.8, 4.3 Hz, 1H, H-3), 2.67 (tt, J = 12.1, 3.7 Hz, 1H, H-7), 2.35 – 2.25 (overlapped, 2H, H-4a and H_a-8), 2.14 (q, J = 12.7 Hz, 1H, H_a-4), 1.89 – 1.77 (overlapped, 3H, H_b-8, H_a-5, and H_b-5), 1.77 – 1.64 (overlapped, 3H, H_b-4, H_a-1, and H_b-1);

^{13}C NMR (100 MHz, $CDCl_3$) δ 146.4 (C-1"), 142.6 (C-1'), 128.7 (2C, C-3"/5"), 128.6 (2C, C-3'/5'), 127.4 (2C, C-2'/6'), 126.9 (2C, C-2"/6"), 126.8 (C-4'), 126.4 (C-4"), 74.4 (C-8a), 66.0 (C-2), 43.7 (C-3), 43.3 (C-7), 34.7 (C-4a), 32.0 (C-8), 30.6 (C-5), 30.3 (C-4), 28.2 (C-6);

HRESIMS (positive ion mode) *m/z* 315.1720 $[M + Na]^+$ (calcd. for $C_{21}H_{24}ONa$, 315.1719);

$[\alpha]_D^{22}$ = 35.5 (c = 0.13 g/100mL, acetone);

HPLC conditions: Chiralpak IF column (*n*-Hexane:2-Propanol = 90:10, 0.5 ml/min, 254nm); tr (minor) = 14.1 min, tr (major) = 13.0 min; 99% *ee*.

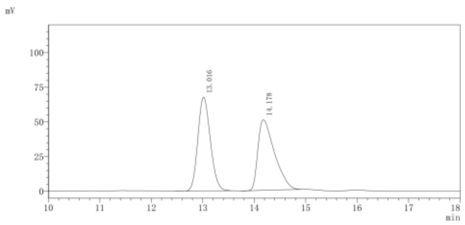

<Peak Table>
検測器A Ch1 254nm

No.	Ret.Time(min)	Height	Height%	Area	Area%
1	13.016	67579	57.082	1142441	50.162
2	14.178	50811	42.918	1135070	49.838
Total		118390	100.000	2277511	100.000

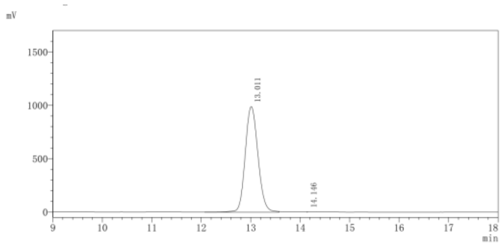

<Peak Table>
検測器A Ch1 254nm

No.	Ret.Time(min)	Height	Height%	Area	Area%
1	13.011	989361	99.695	17686686	99.983
2	14.146	3026	0.305	3025	0.017
Total		992387	100.000	17689712	100.000

8.3 (1a*R*,1b*S*,3a*R*,4a*S*,4b*R*,6a*R*)-1,1,4,4-tetrafluoro-3a,6a-diphenyldecahydro-1*H*-dicycloprop a [*c*,*h*]chromene (6)

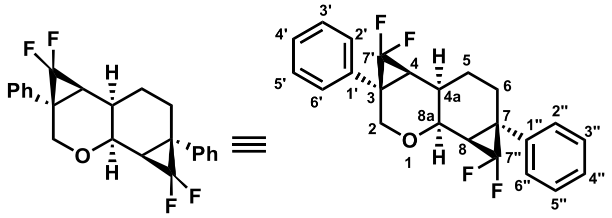

To a dry sealed tube was added material **3aa** (99% *ee*, 0.2 mmol, 57.7mg) and sodium iodide (40 mol%), dried THF (2.0 mL) was added under argon atmosphere, and then $TMSCF_3$ (14.0 eq) was added to the mixture. The reaction mixture was allowed to stir at 65 °C for 12 hours. The reaction was completed by TLC monitoring, then the reaction mixture was concentrated and purified by silica gel chromatography (*n*-Hexane /EA = 20:1) to obtain white solid **6** (74% yield, 57.5 mg, 96% *ee*) (m.p.: 135.7 °C).

^{1}H NMR (400 MHz, $CDCl_3$) δ 7.40 – 7.26 (overlapped, 10H, H-2' – H-6"), 4.25 (d, J = 2.9 Hz, 2H, H-2), 3.87 (s, 1H, H-8a), 2.26 (m, 1H, H_a-6), 2.12 (m, 1H, H_b-6), 2.06 (m, 1H, H-8), 1.99 (m, 1H, H_a-5), 1.95 (m, 1H, H-4a), 1.77 (dd, J = 13.8, 3.9 Hz, 1H, H-4), 1.53 (m, 1H, H_b-5);

^{13}C NMR (100 MHz, $CDCl_3$) δ 140.6 (C-1"), 139.1 (C-1'), 128.9 (2C, C-3'/5' or C-3"/5"), 128.7 (2C, C-3'/5' or C-3"/5"), 128.4 (d, $^3J_{C\text{-}F}$ = 1.9 Hz, 2C, C-2'/6' or C-2"/6"), 128.3 (d, $^3J_{C\text{-}F}$ = 1.9 Hz, 2C, C-2'/6' or C-3"/6"), 127.6 (C-4"), 127.3 (C-4'), 114.1 (m, C-7'), 111.2 (m, C-7"), 68.4 (C-2), 64.9 (d, $^3J_{C\text{-}F}$ = 4.6 Hz, C-8a), 31.8 (t, $^2J_{C\text{-}F}$ = 9.5 Hz, C-7), 30.9 (t, $^2J_{C\text{-}F}$ = 9.3 Hz, C-3), 30.2 (t, $^2J_{C\text{-}F}$ = 10.3 Hz, C-4), 29.8 (t, $^2J_{C\text{-}F}$ = 10.1 Hz, C-8), 26.3 (C-6), 24.1 (C-4a), 24.0 (C-5);

^{19}F NMR (376 MHz, $CDCl_3$) δ -127.3 (dd, J = 153.7, 13.6 Hz, 1F), -129.4 (dd, J = 154.3, 15.9 Hz, 1F), -140.0 (dt, J = 154.0, 4.4 Hz, 1F), -140.8 (dt, J = 154.0, 3.5 Hz, 1F);

HRESIMS (positive ion mode) *m/z* 411.1336 $[M + Na]^+$ (calcd. for $C_{23}H_{20}F_4ONa$, 411.1342);

$[\alpha]_D^{22}$ = -134.1 (c = 0.11 g/100mL, acetone);

HPLC conditions: Chiralpak IA column (*n*-Hexane:2-Propanol = 90:10, 0.5 ml/min, 254nm); tr (minor) = 7.4 min, tr (major) = 8.0 min; 96% *ee*.

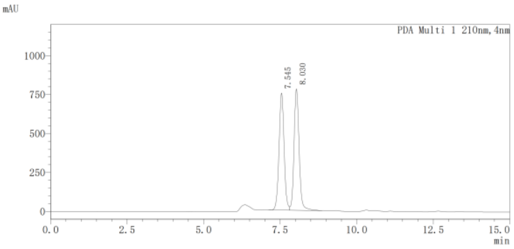

<Peak Table>
PDA Ch1 210nm

No.	Ret.Time(min)	Height	Height%	Area	Area%
1	7.545	751210	49.094	8684020	49.124
2	8.030	778942	50.906	8993888	50.876
Total		1530152	100.000	17677908	100.000

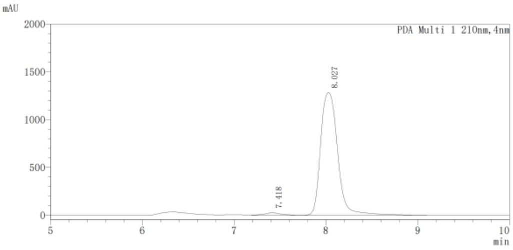

<Peak Table>
PDA Ch1 210nm

No.	Ret.Time(min)	Height	Height%	Area	Area%
1	7.418	25130	1.920	311387	1.851
2	8.027	1283684	98.080	16507685	98.149
Total		1308814	100.000	16819072	100.000

8.4 (1a*S*,1b*S*,3a*R*,4a*S*,4b*S*,6a*S*)-3a,6a-diphenyloctahydro-3*H*-bis(oxireno)[2,3-*c*:2',3'-*h*]chromene (7)

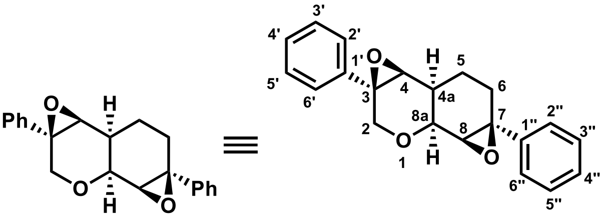

To a solution of **3aa** (99% ee, 0.2 mmol,57.7mg) in DCM (4 mL) was sequentially added an aqueous solution of Na_2CO_3 (127 mg, 6.0 eq) and 3-chloroperoxybenzoic acid (*m*-CPBA, 138 mg, 4.0 eq) at r.t. The reaction mixture was stirred for 2 h and poured into a saturated solution of aqueous sodium bicarbonate. The two layers were separated and the organic layer was washed with a saturated solution of aqueous sodium bicarbonate. This procedure was repeated twice more. The organic layer was dried over $MgSO_4$, filtered, and concentrated on a rotary evaporator. The crude product was purified by column chromatography (*n*-Hexane /EA = 20:1) to give **7** (77 % yield, 49.3mg, 99% *ee*) as a white solid (m.p.: 131.5 °C).

^{1}H NMR (400 MHz, $CDCl_3$) δ 7.43 – 7.27 (overlapped, 10H, H-2' – H-6''), 4.48 (d, J = 13.5 Hz, 1H, H_a-2), 4.19 (dd, J = 4.4, 1.9 Hz, 1H, H-8a), 4.12 (d, J = 13.5 Hz, 1H, H_b-2), 3.18 (s, 1H, H-8), 3.10 (s, 1H, H-4), 2.46 (ddd, J = 15.4, 5.9, 4.7 Hz, 1H, H_a-6), 2.36 (m, 1H, H-4a), 2.15 (ddd, J = 15.7, 10.1, 6.0 Hz, 1H, H_b-6), 1.82 (m, 1H, H_a-5), 1.63 (m, 1H, H_b-5);

^{13}C NMR (100 MHz, $CDCl_3$) δ 141.1 (C-1''), 138.4 (C-1'), 128.6 (2C, C-3''/5''), 128.4 (2C, C-3'/5'), 128.0 (C-4'), 127.6 (C-4''), 125.6 (2C, C-2'/6'), 125.4 (2C, C-2''/6''), 68.1 (C-2), 67.2 (C-8a), 63.8 (C-4), 62.5 (C-8), 61.2 (C-7), 59.5 (C-3), 30.0 (C-4a), 27.0 (C-5), 19.3 (C-6);

HRESIMS (positive ion mode) *m/z* 343.1304 $[M + Na]^+$ (calcd. for $C_{21}H_{20}O_3Na$, 343.1305);

$[\alpha]_D^{22}$ = 10.4 (c = 0.10 g/100mL, acetone);

HPLC conditions: Chiralpak ID column (*n*-Hexane:2-Propanol = 90:10, 0.5 ml/min, 254 nm); tr (minor) = 10.7 min, tr (major) = 14.1 min; 99% *ee*.

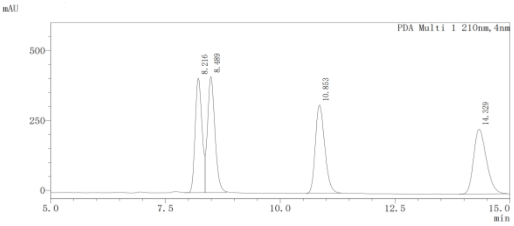

<Peak Table>

PDA Ch1 210nm

No.	Ret.Time(min)	Height	Height%	Area	Area%
1	8.216	409937	29.874	4303436	23.975
2	8.489	415233	30.260	4660837	25.967
Total	10.853	315444	22.988	4496994	25.054
4	14.329	231595	16.878	4488084	25.004
总计		1372209	100.000	17949351	100.000

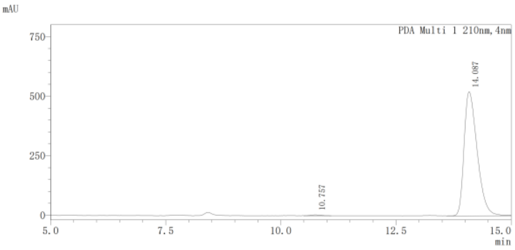

<Peak Table>

PDA Ch1 210nm

No.	Ret.Time(min)	Height	Height%	Area	Area%
1	10.757	3789	0.722	57653	0.556
2	14.087	521186	99.278	10303383	99.444
Total		524975	100.000	10361035	100.000

9. NMR Spectra

^{1}H NMR of Xu3 (400 MHz, $CDCl_3$)

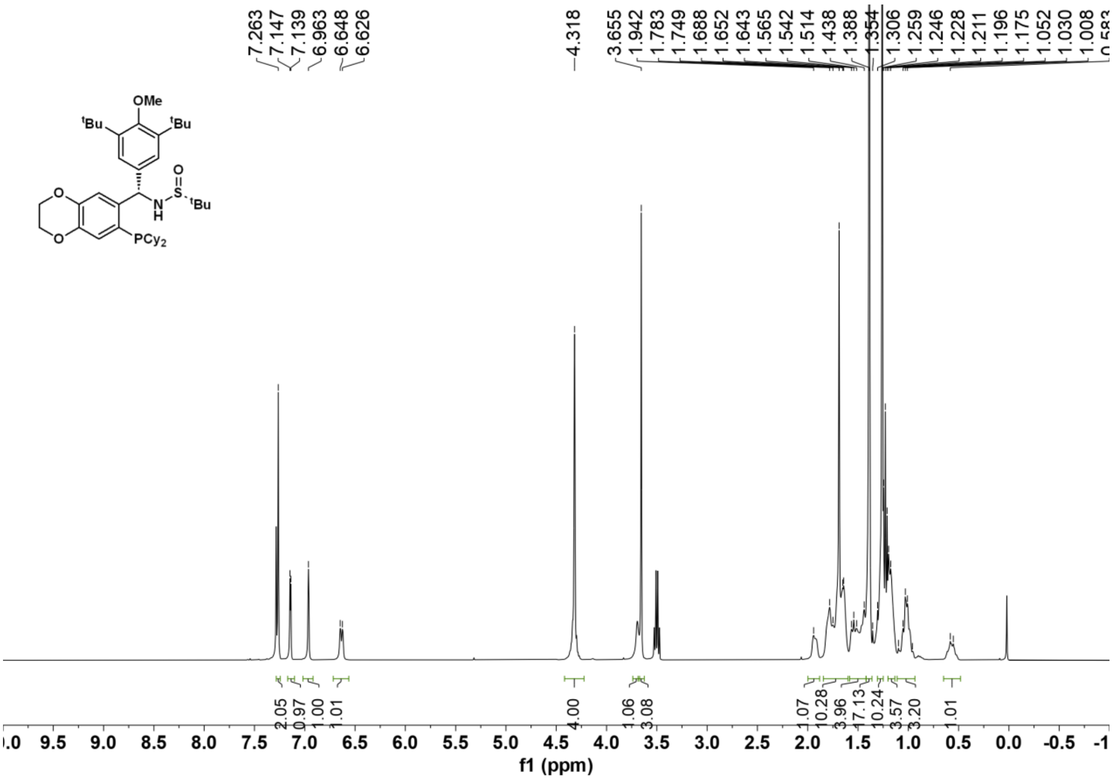

^{13}C NMR of Xu3 (100 MHz, $CDCl_3$)

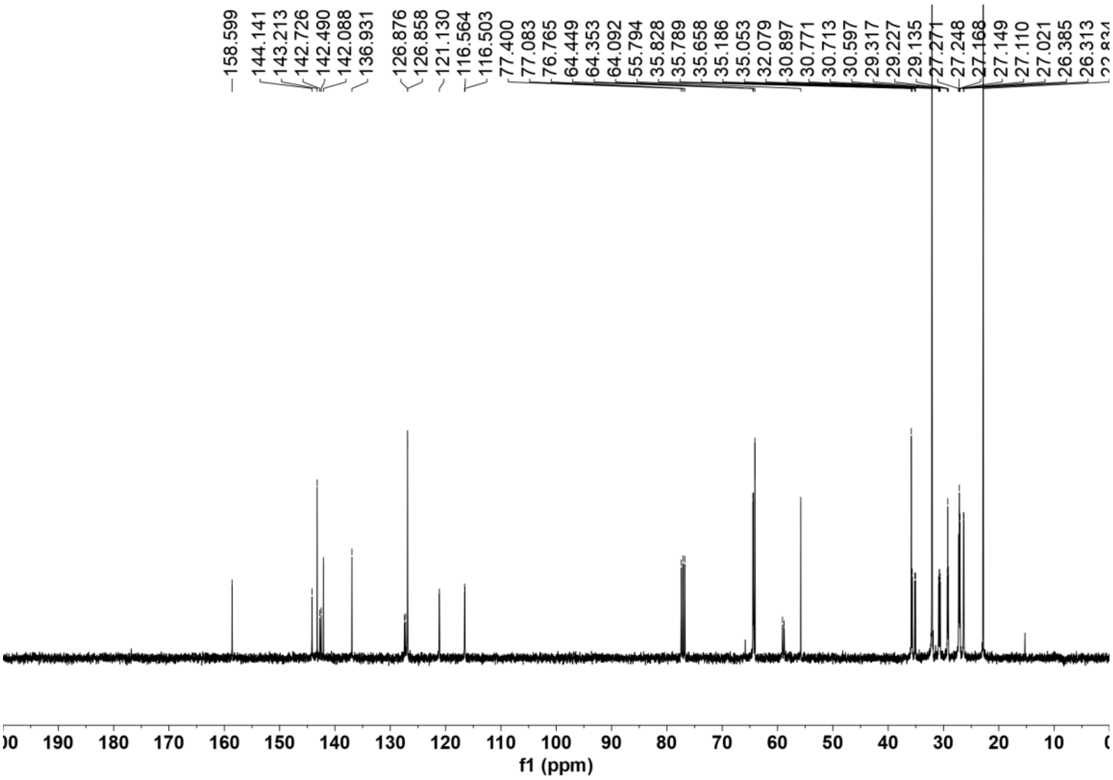

^{31}P NMR of Xu3 (130 MHz, $CDCl_3$)

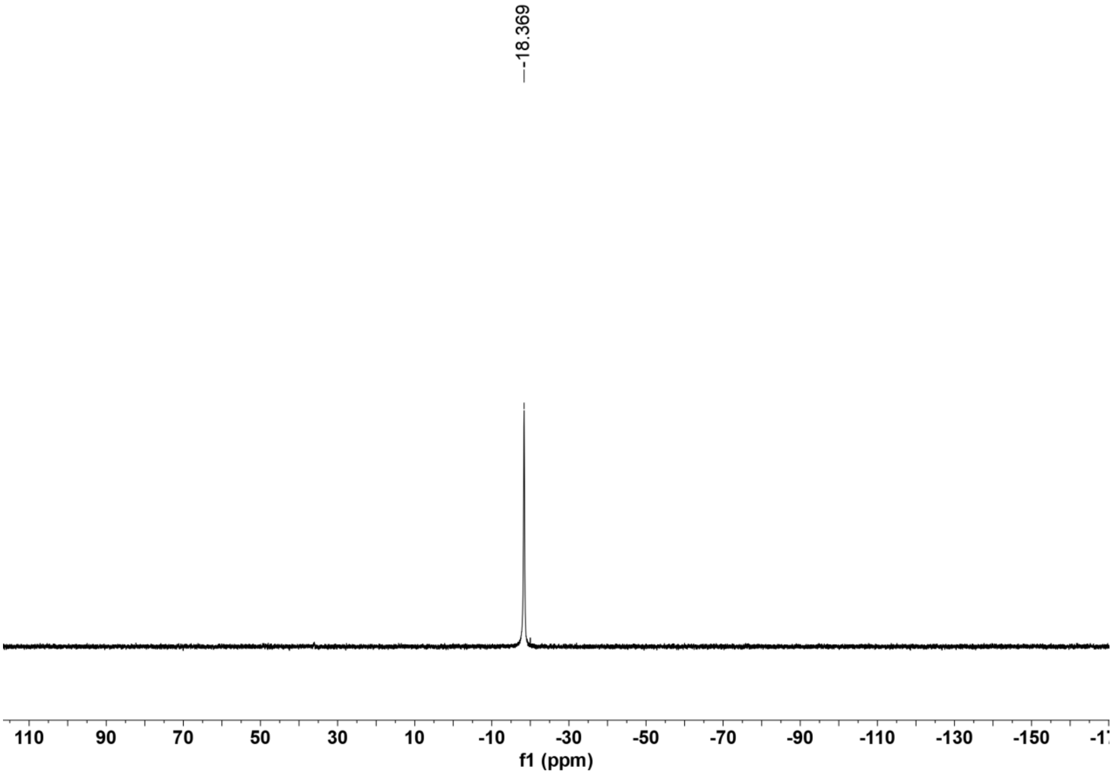

^{1}H NMR of 3aa (400 MHz, $CDCl_3$)

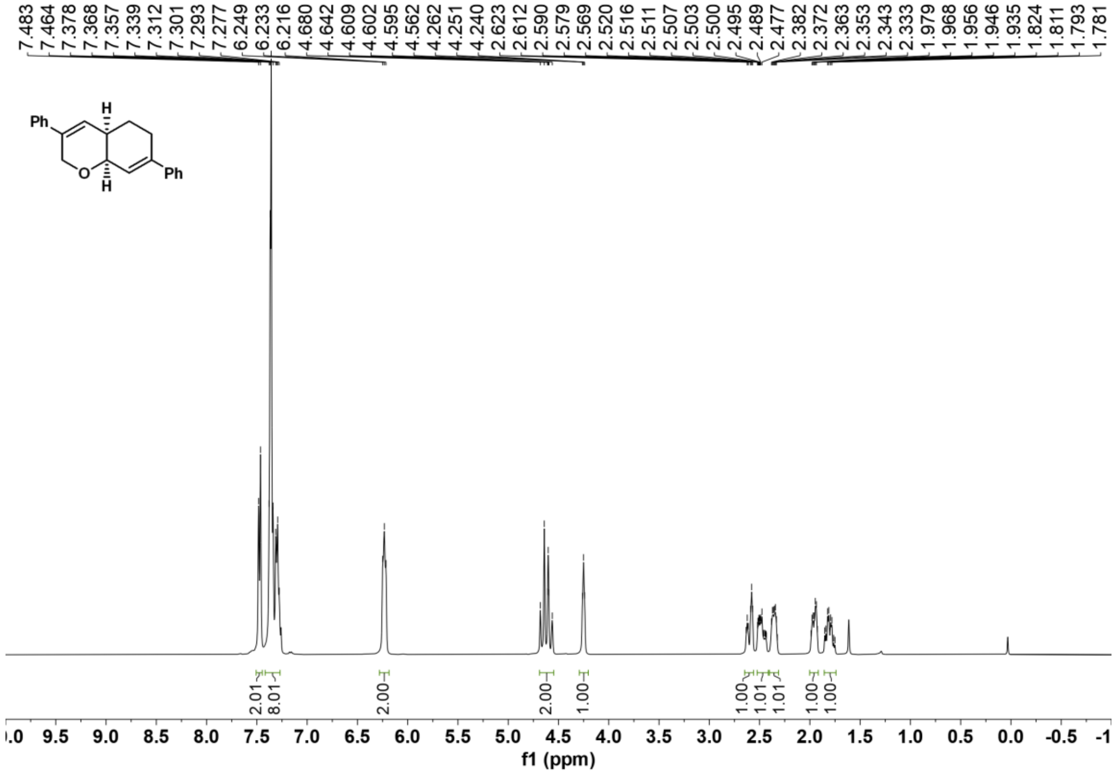

^{13}C NMR of 3aa (100 MHz, $CDCl_3$)

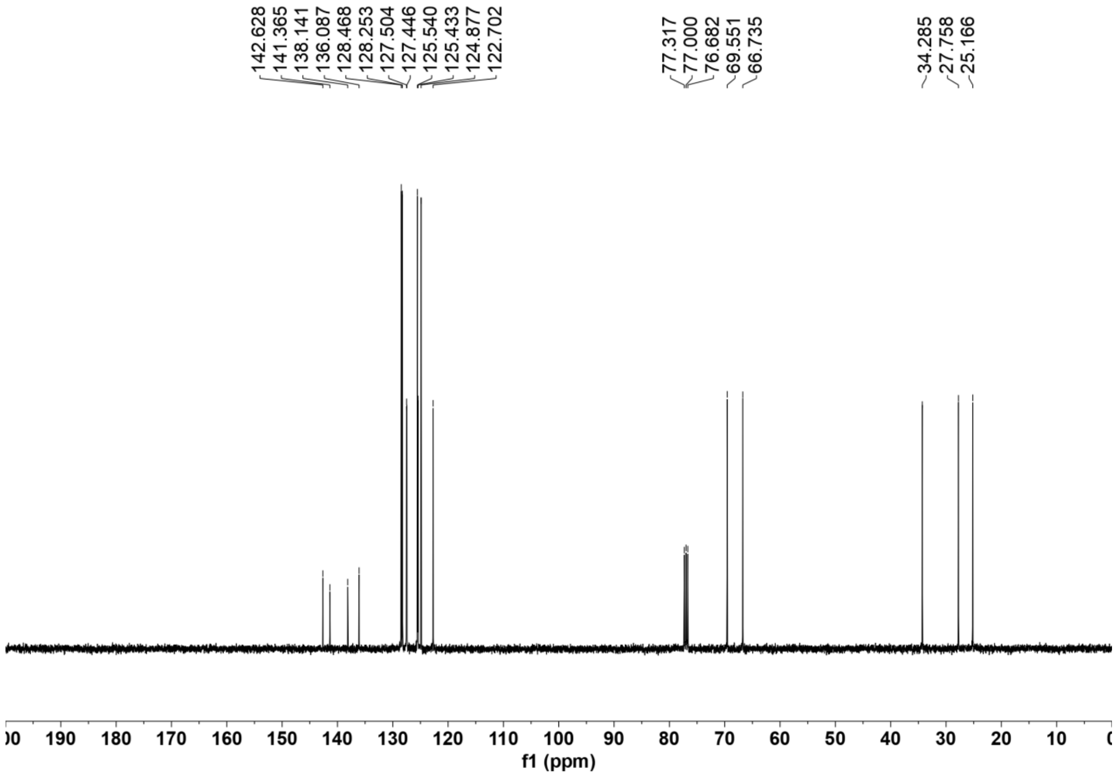

^{1}H NMR of 3ab (400 MHz, $CDCl_3$)

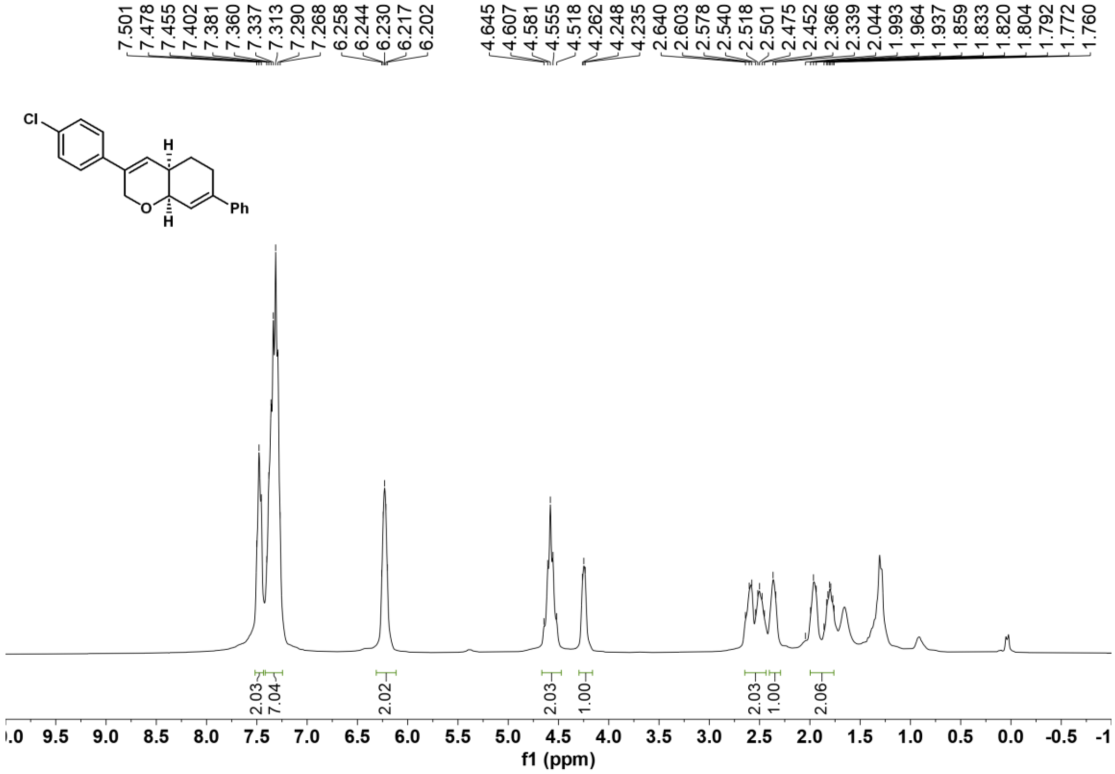

^{13}C NMR of 3ab (100 MHz, $CDCl_3$)

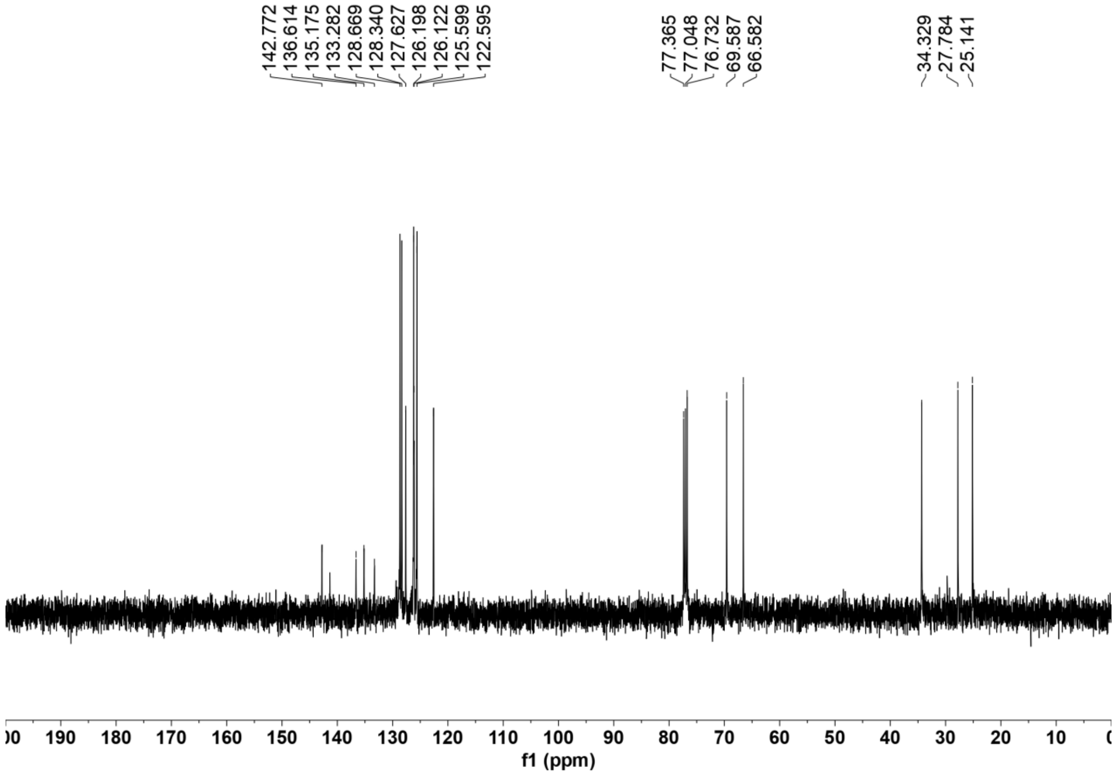

^{1}H NMR of 3ac (400 MHz, $CDCl_3$)

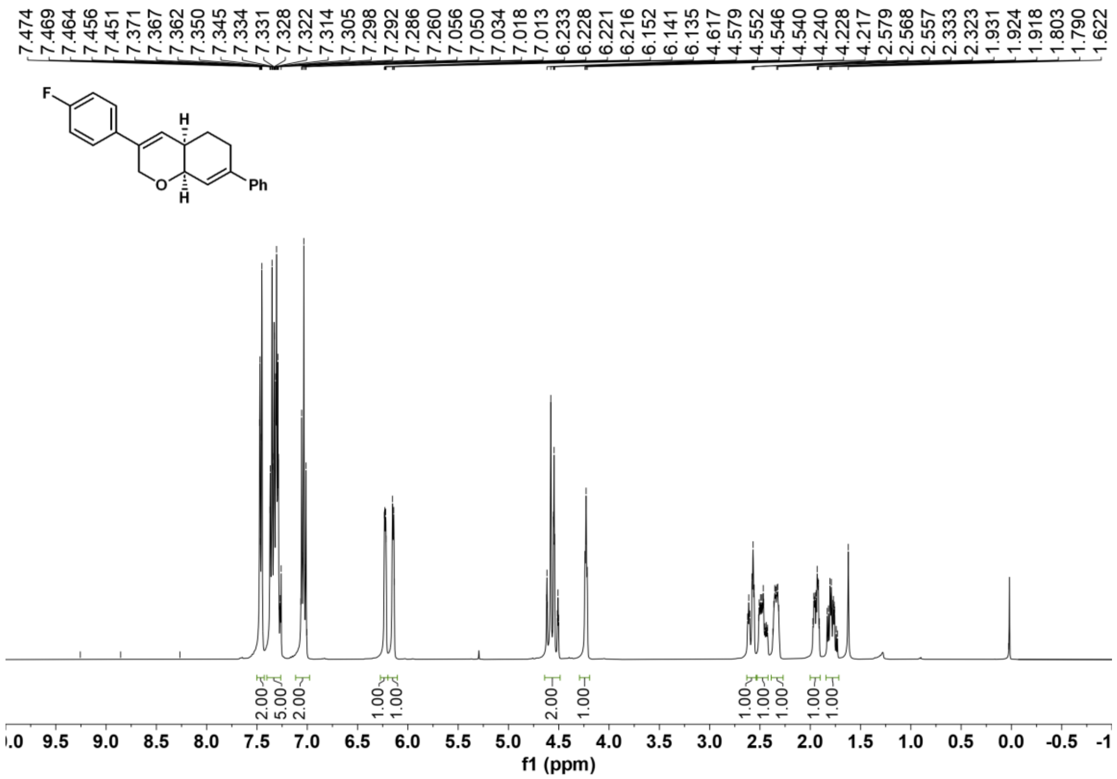

^{13}C NMR of 3ac (100 MHz, $CDCl_3$)

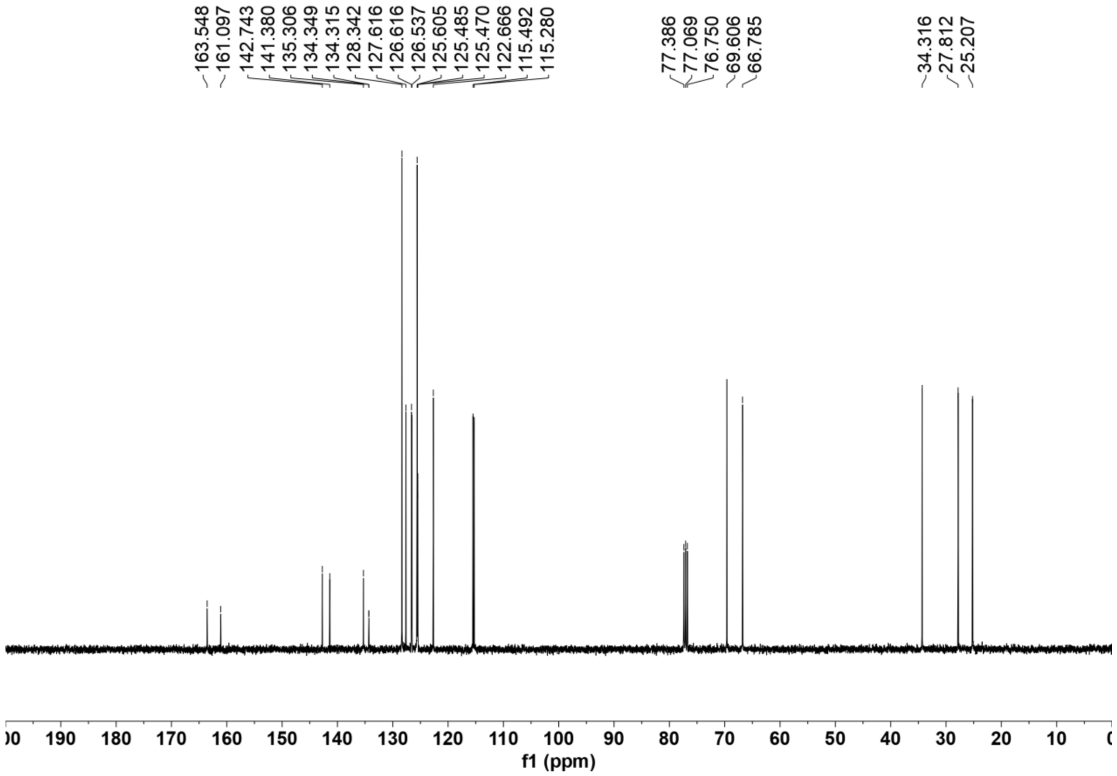

^{19}F NMR of 3ac (376 MHz, $CDCl_3$)

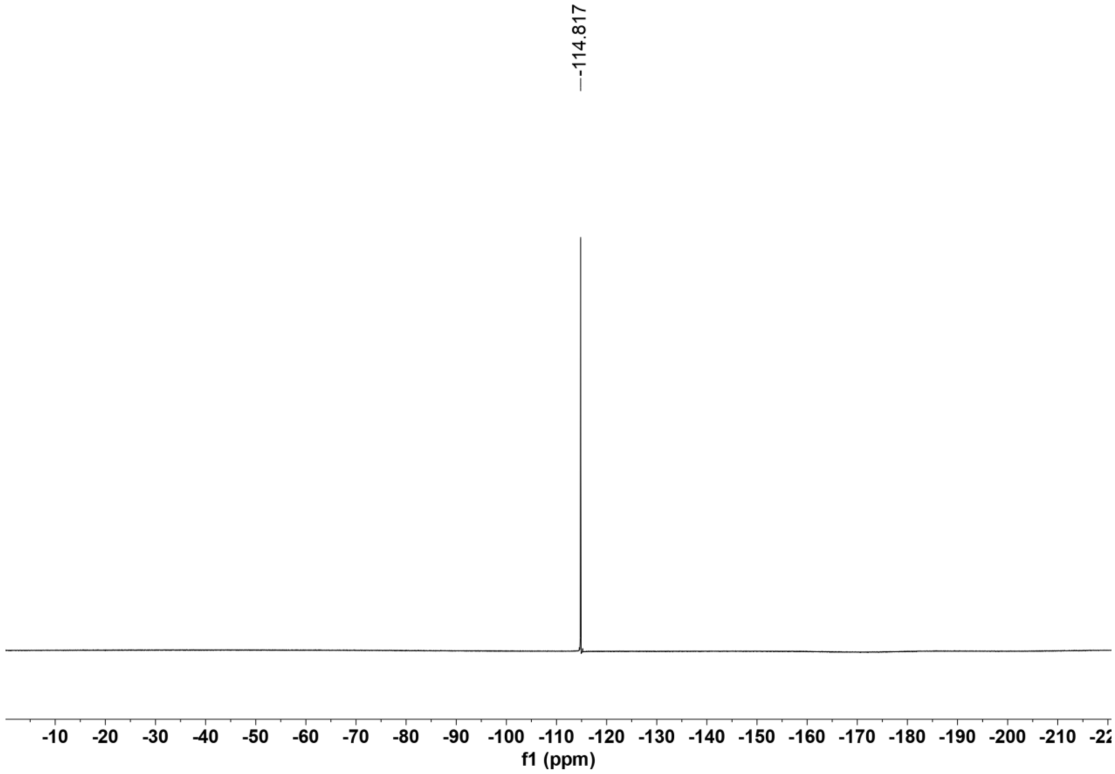

^{1}H NMR of 3ad (400 MHz, $CDCl_3$)

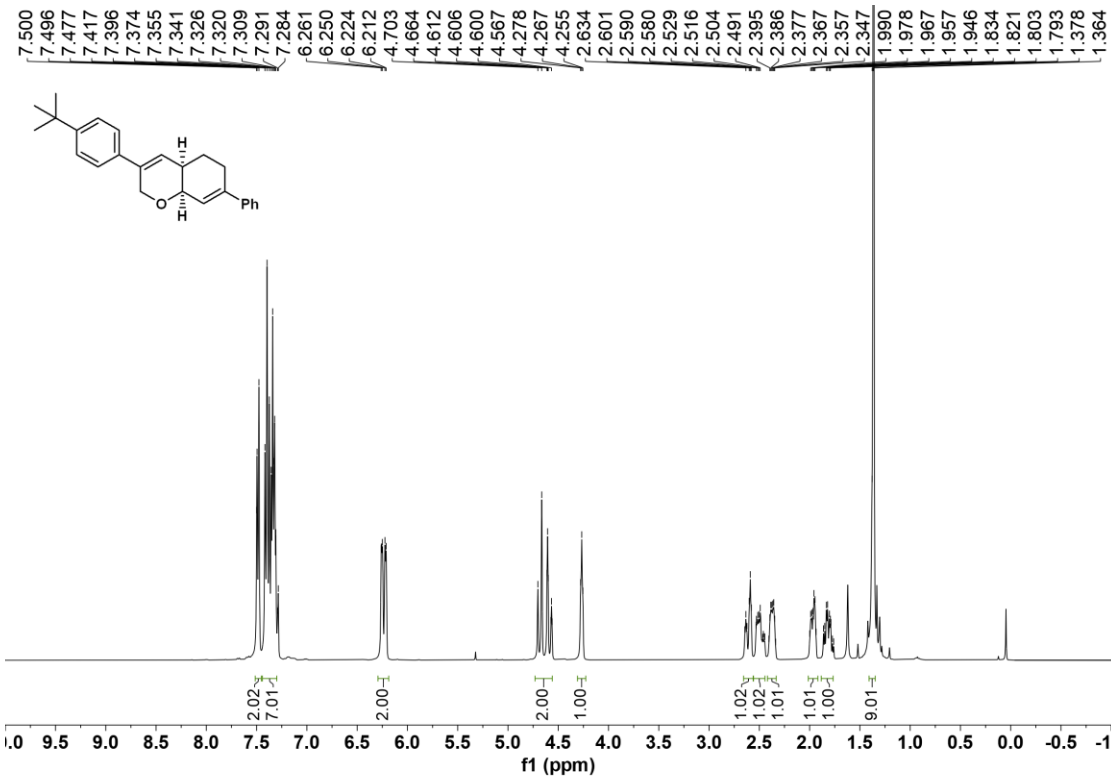

^{13}C NMR of 3ad (100 MHz, $CDCl_3$)

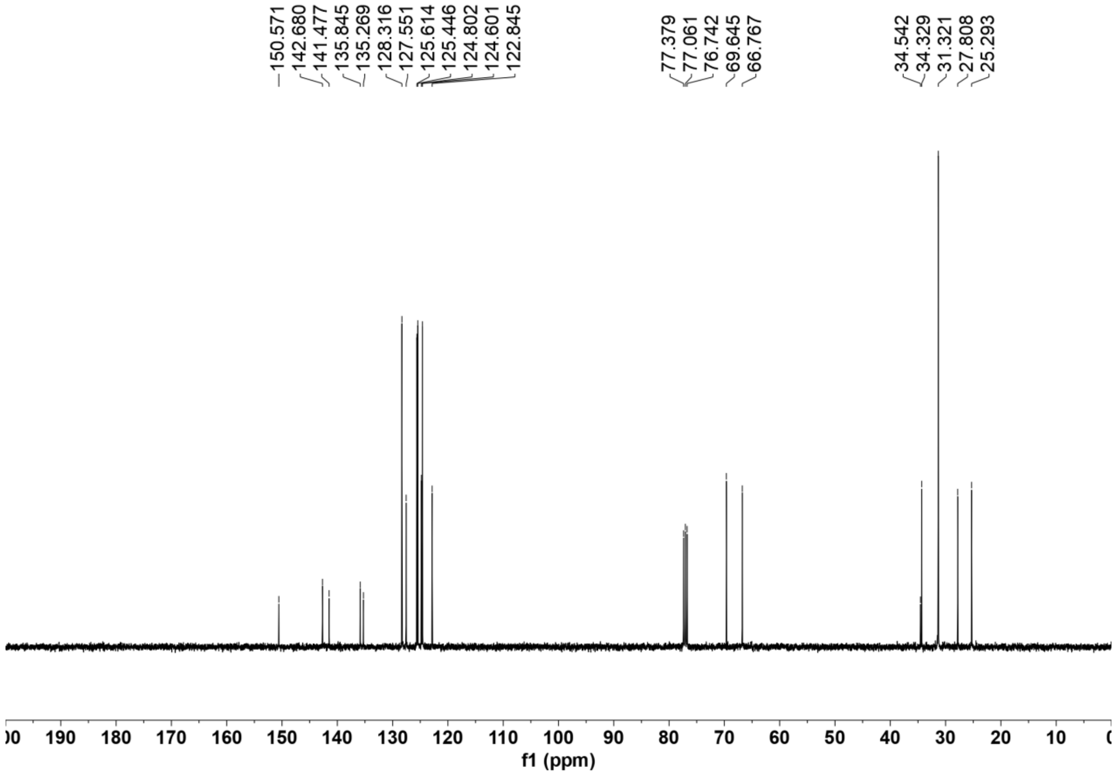

^{1}H NMR of 3ae (400 MHz, $CDCl_3$)

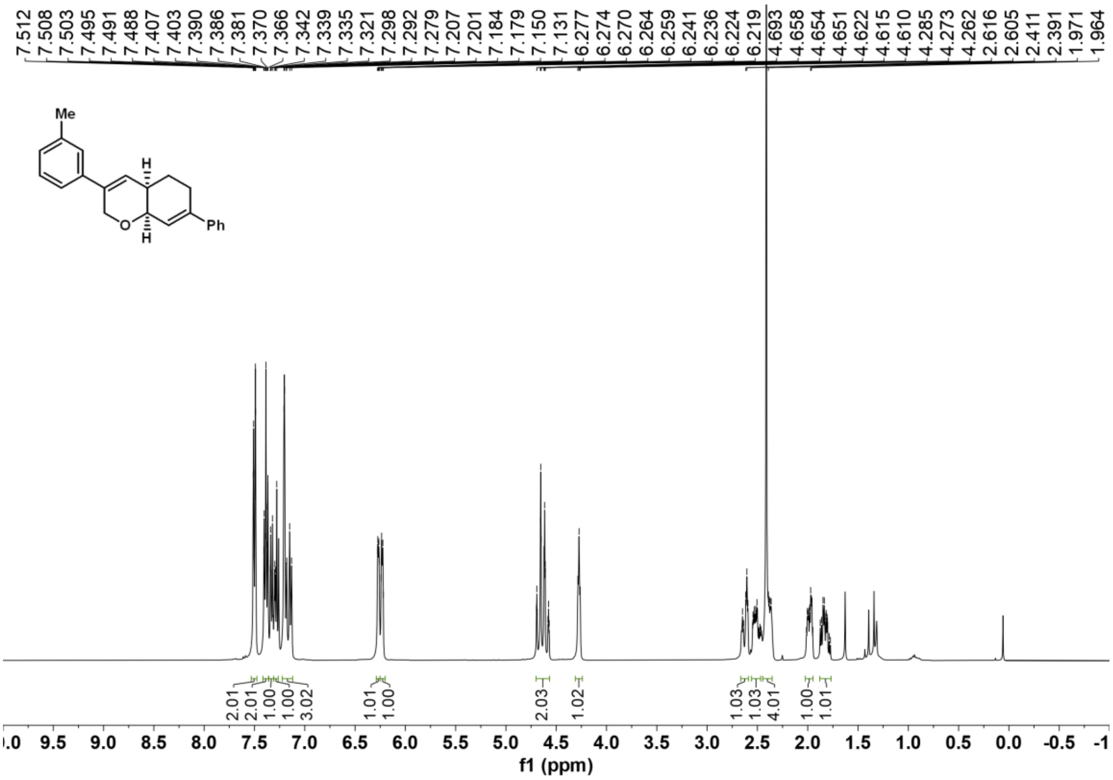

^{13}C NMR of 3ae (100 MHz, $CDCl_3$)

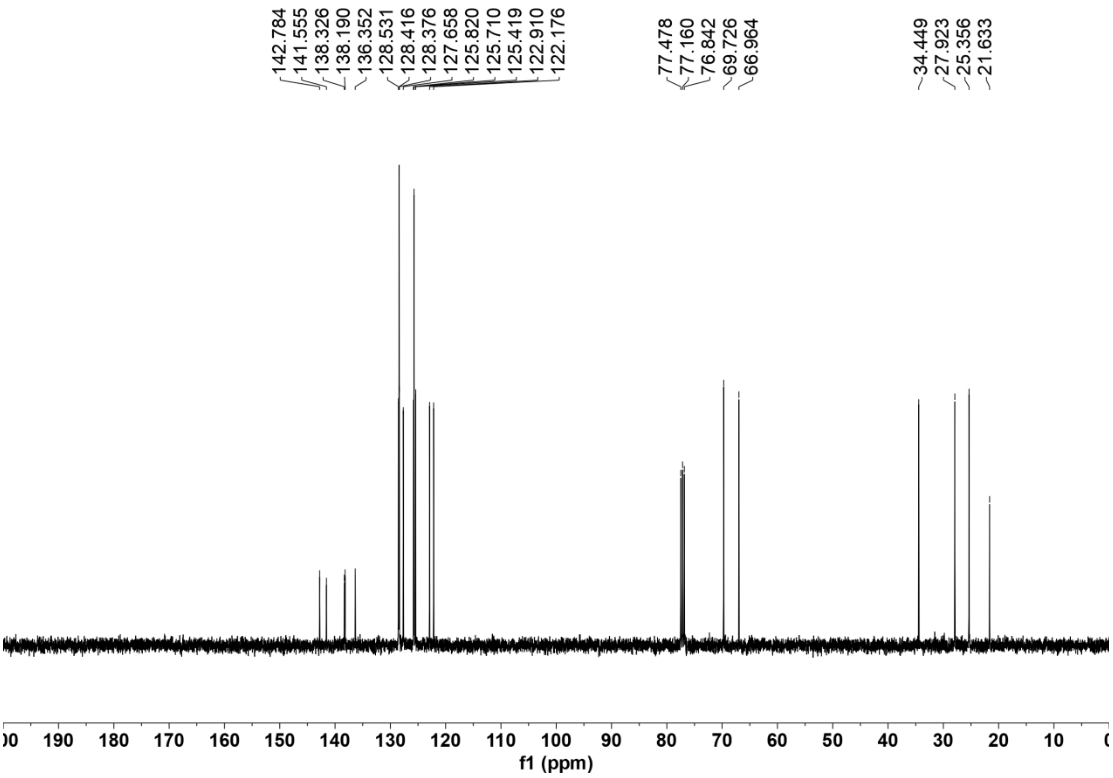

^{1}H NMR of 3af (400 MHz, $CDCl_3$)

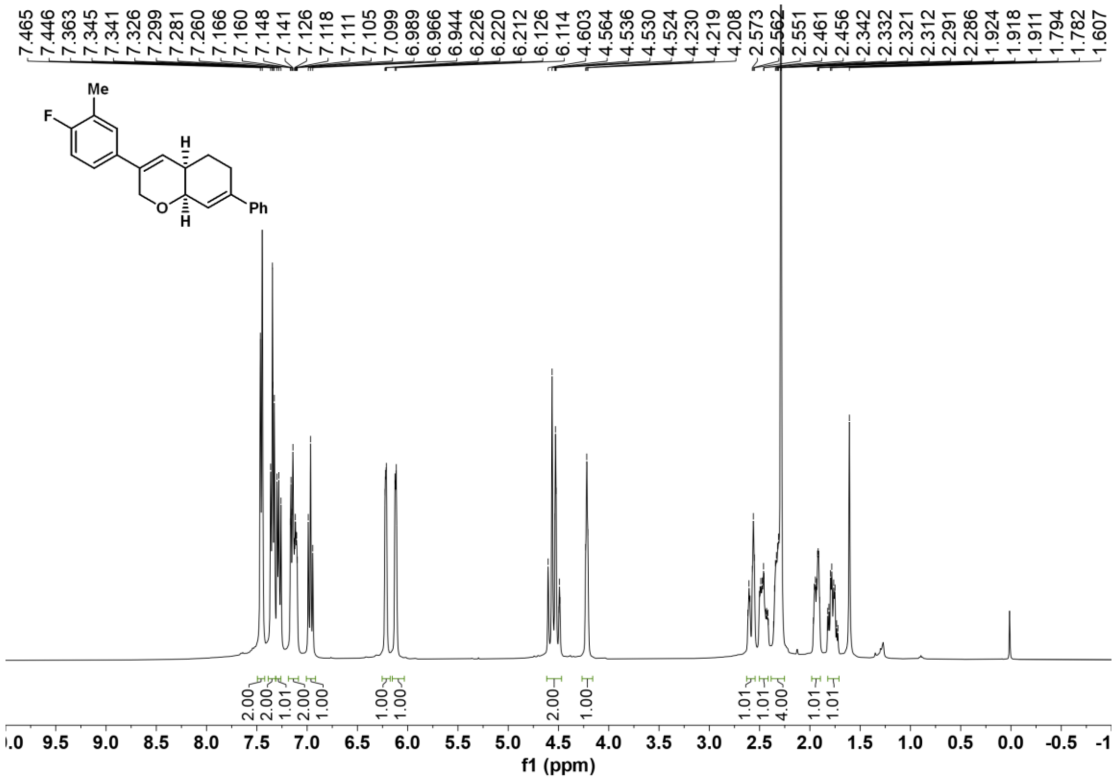

^{13}C NMR of 3af (100 MHz, $CDCl_3$)

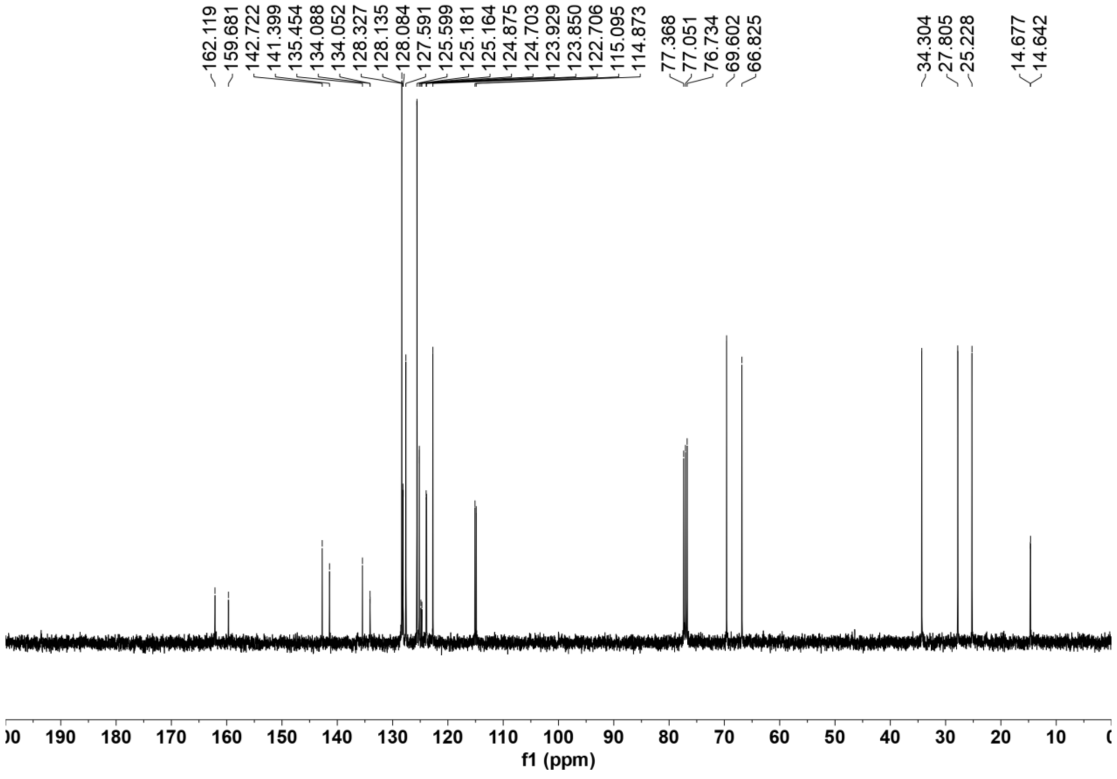

^{19}F NMR of 3af (376 MHz, $CDCl_3$)

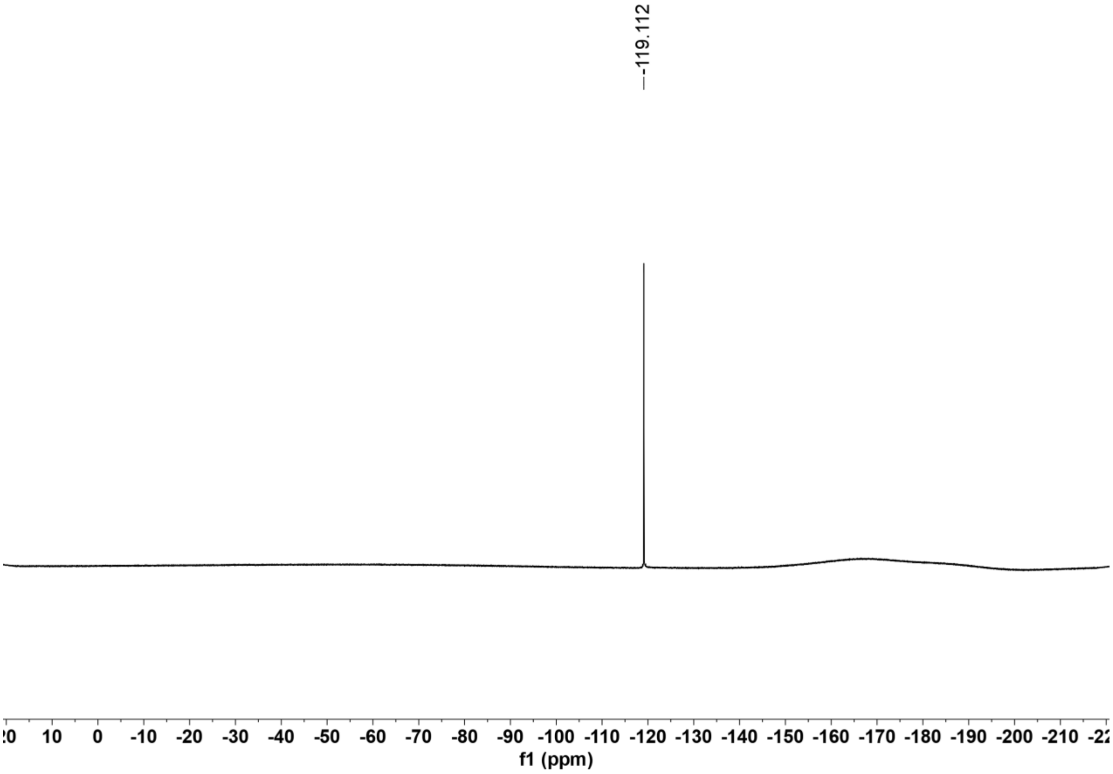

^{1}H NMR of 3ag (400 MHz, $CDCl_3$)

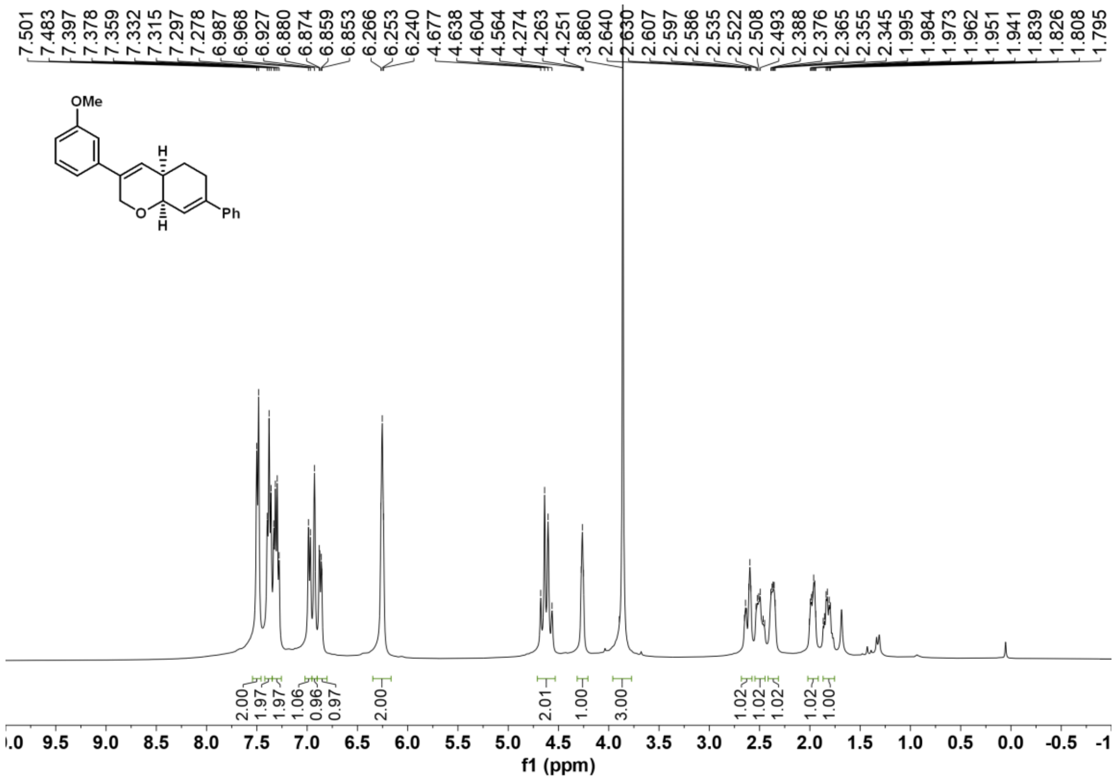

^{13}C NMR of 3ag (100 MHz, $CDCl_3$)

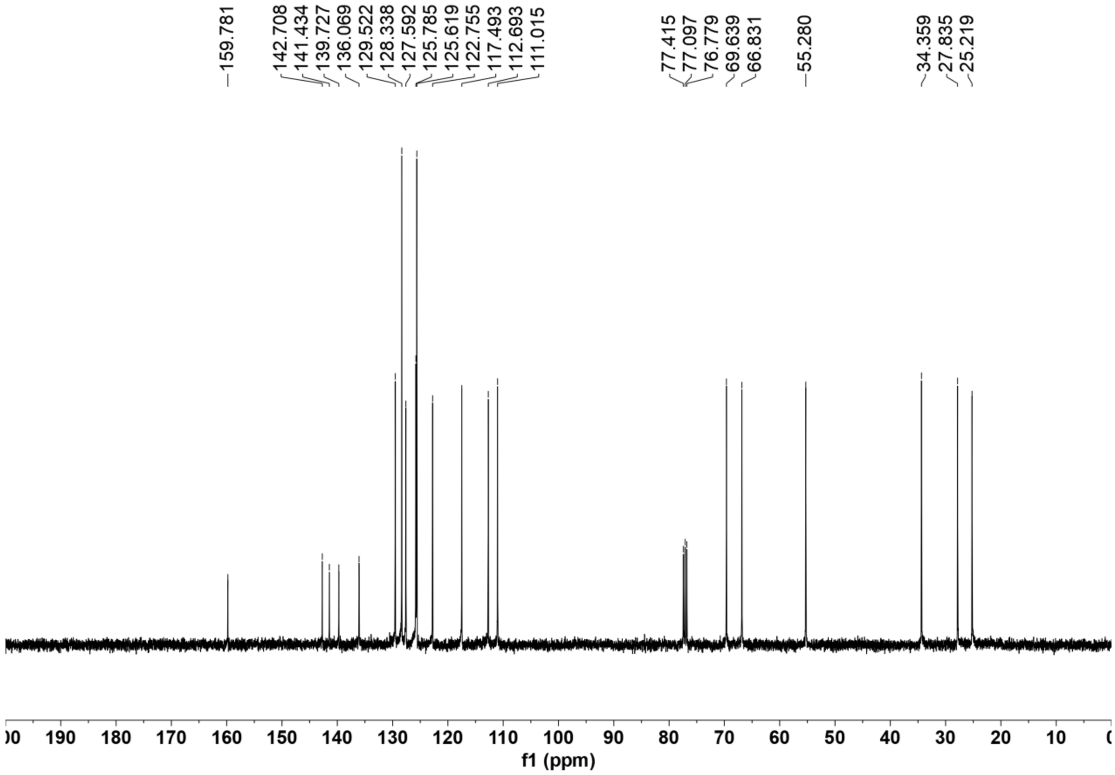

¹H NMR of 3ah (400 MHz, $CDCl_3$)

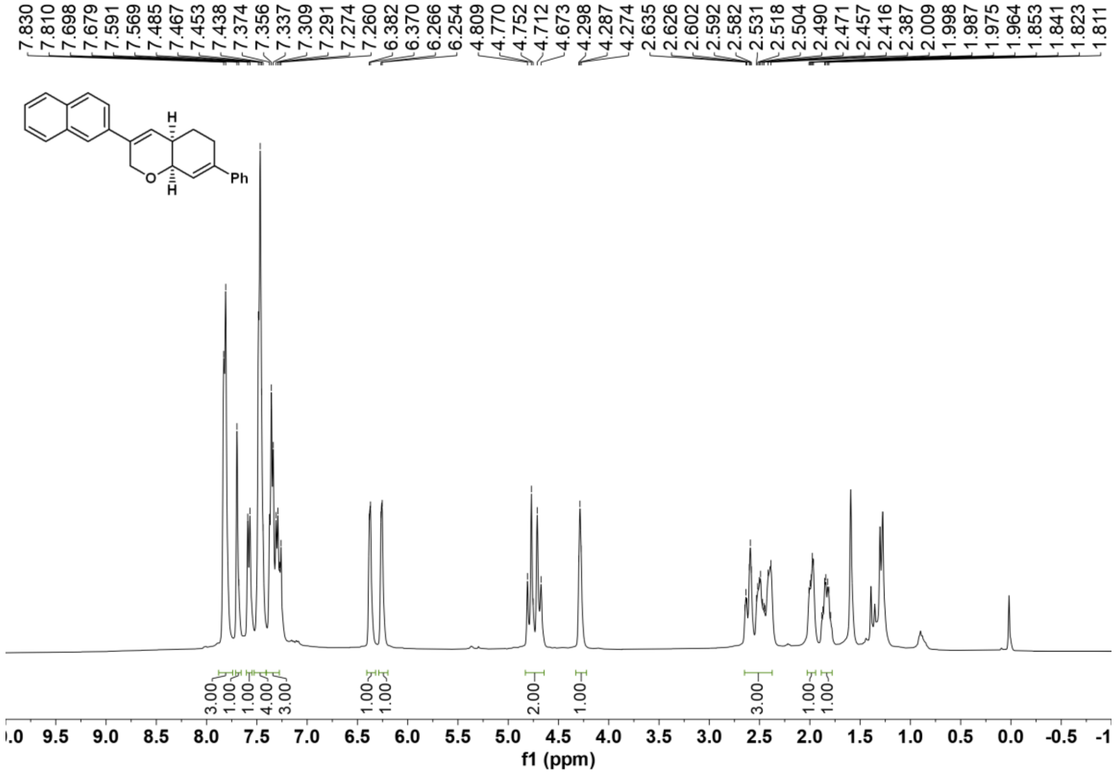

¹³C NMR of 3ah (100 MHz, $CDCl_3$)

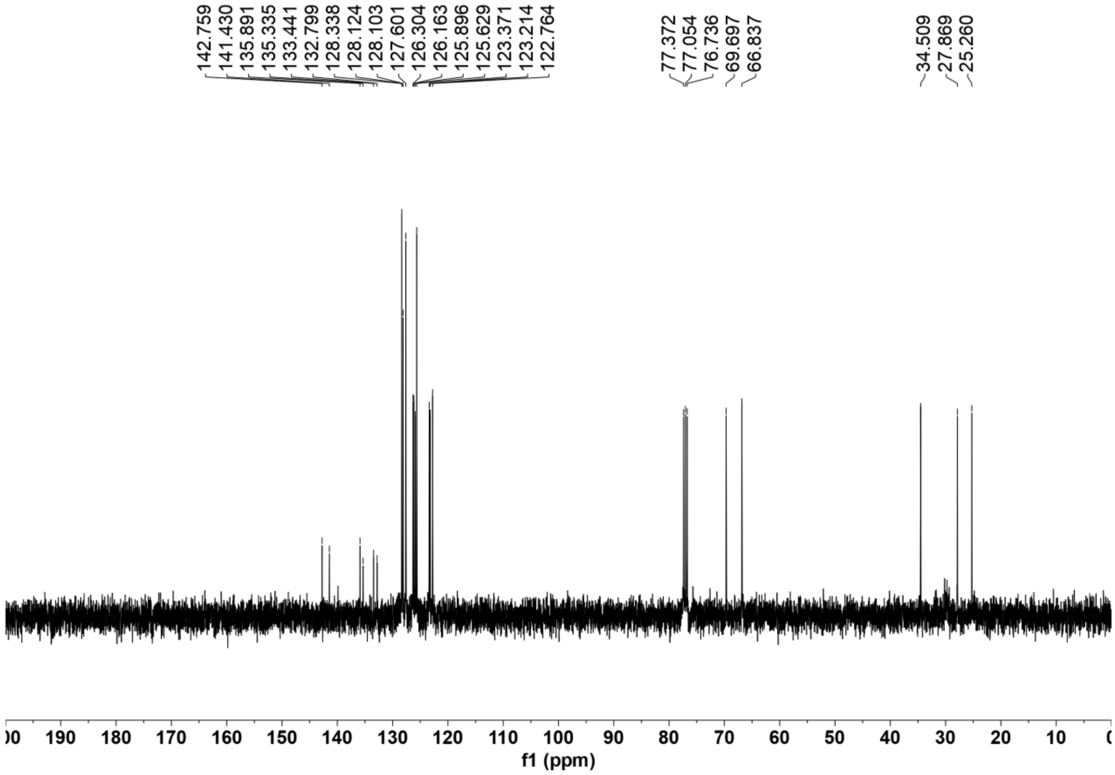

^{1}H NMR of 3ai (400 MHz, $CDCl_3$)

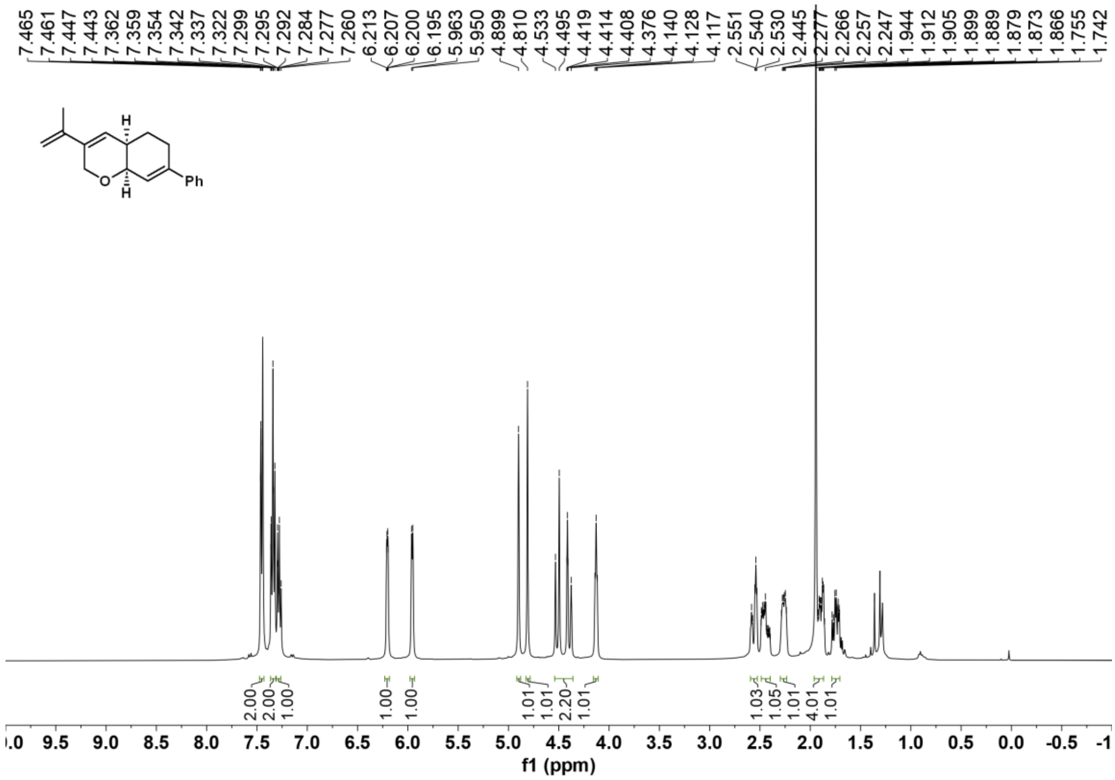

^{13}C NMR of 3ai (100 MHz, $CDCl_3$)

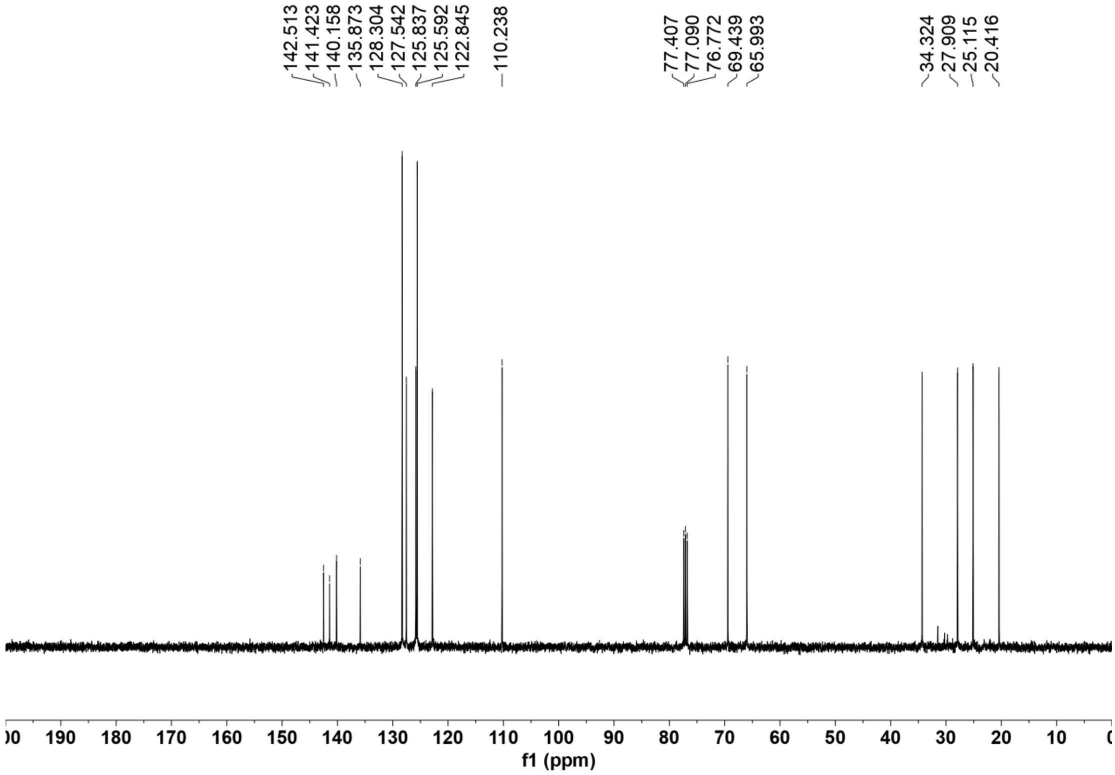

^{1}H NMR of 3aj (400 MHz, $CDCl_3$)

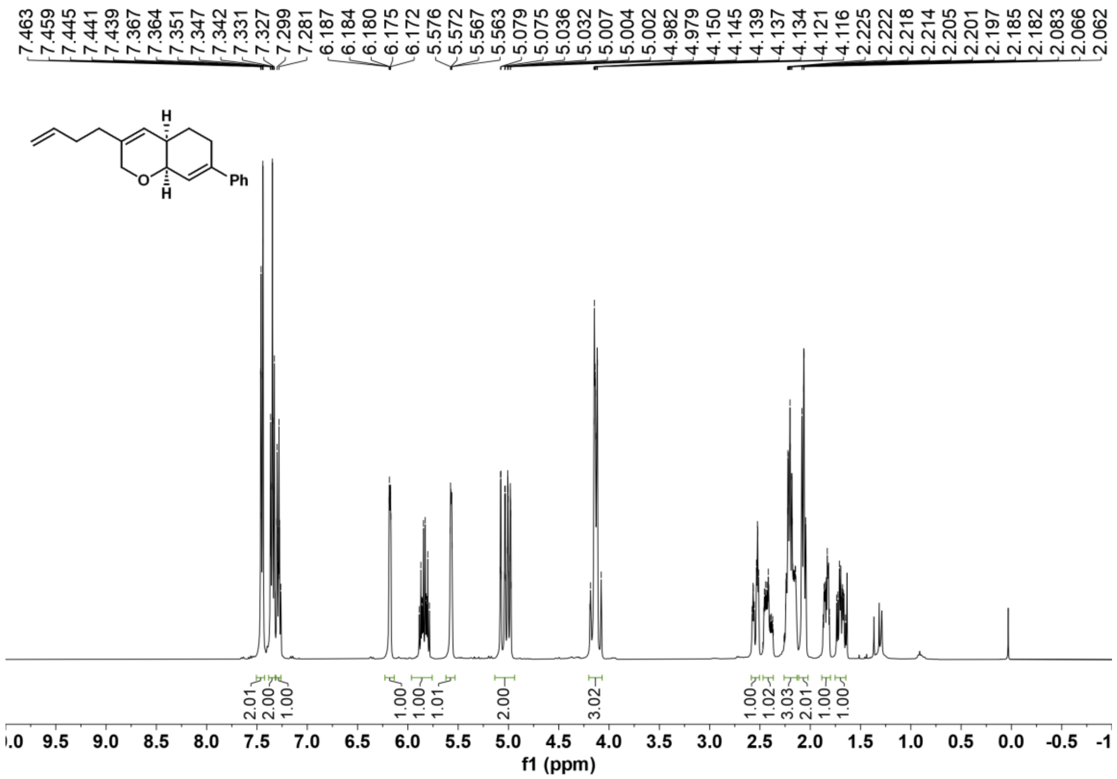

^{13}C NMR of 3aj (100 MHz, $CDCl_3$)

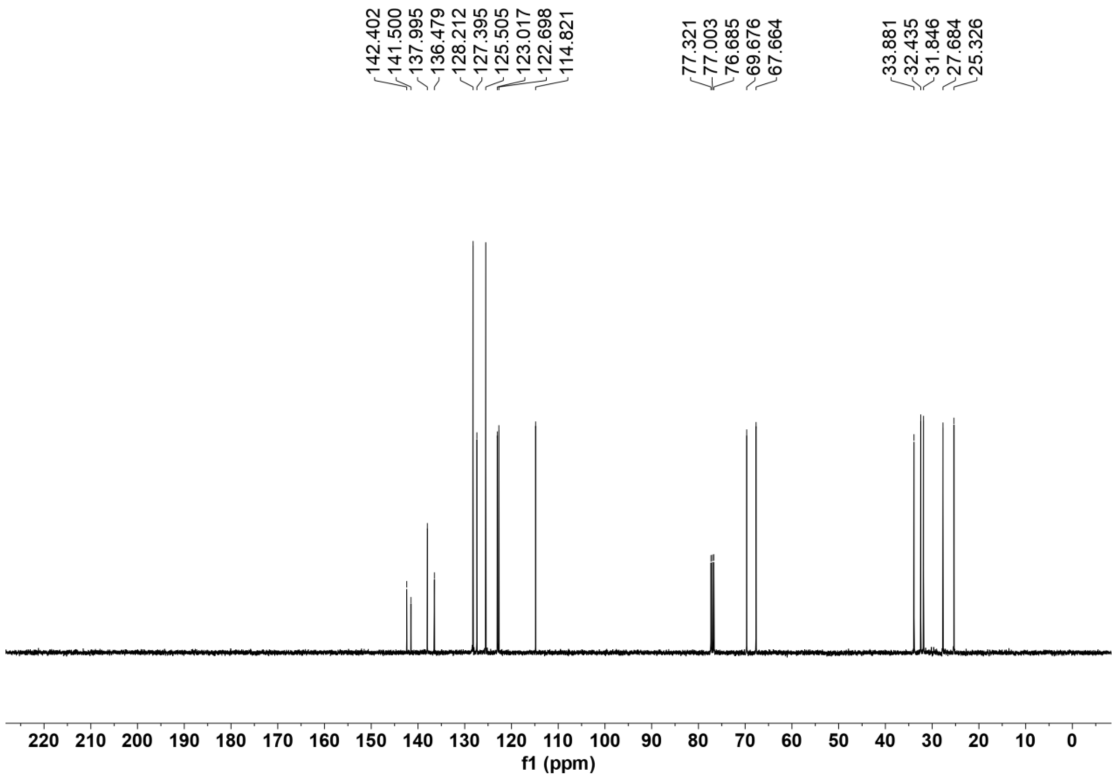

^{1}H NMR of 3ak (400 MHz, $CDCl_3$)

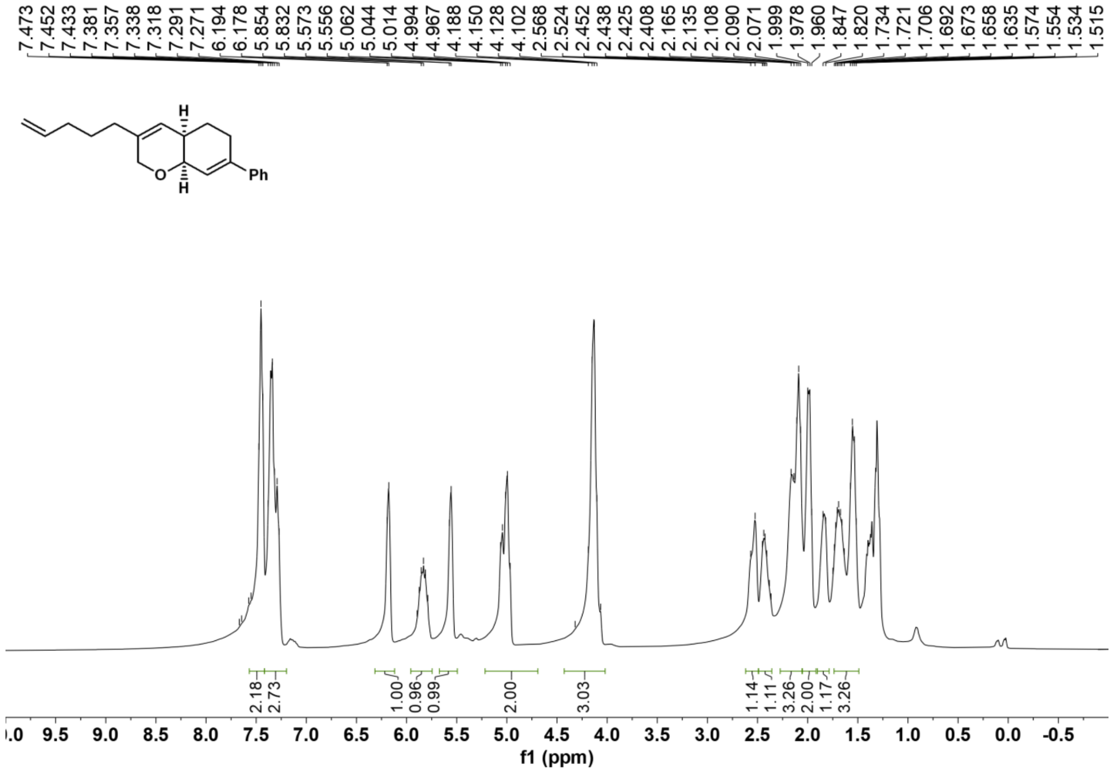

^{13}C NMR of 3ak (100 MHz, $CDCl_3$)

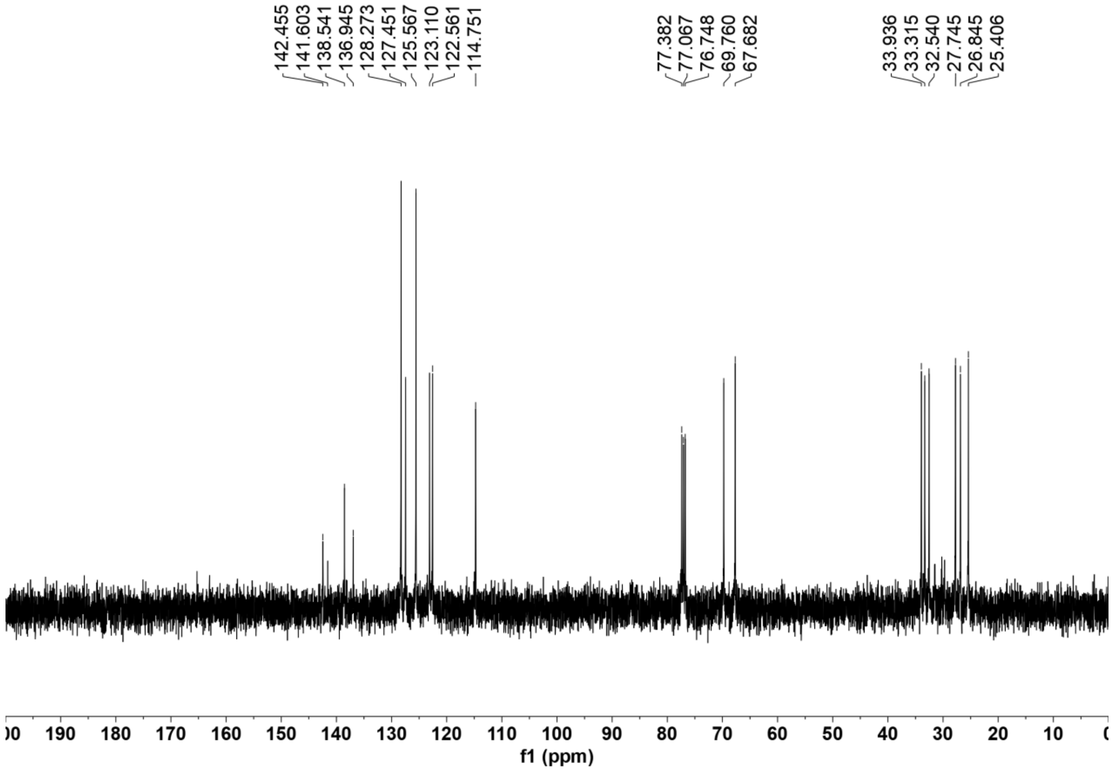

^{1}H **NMR of 3al** (400 MHz, $CDCl_3$)

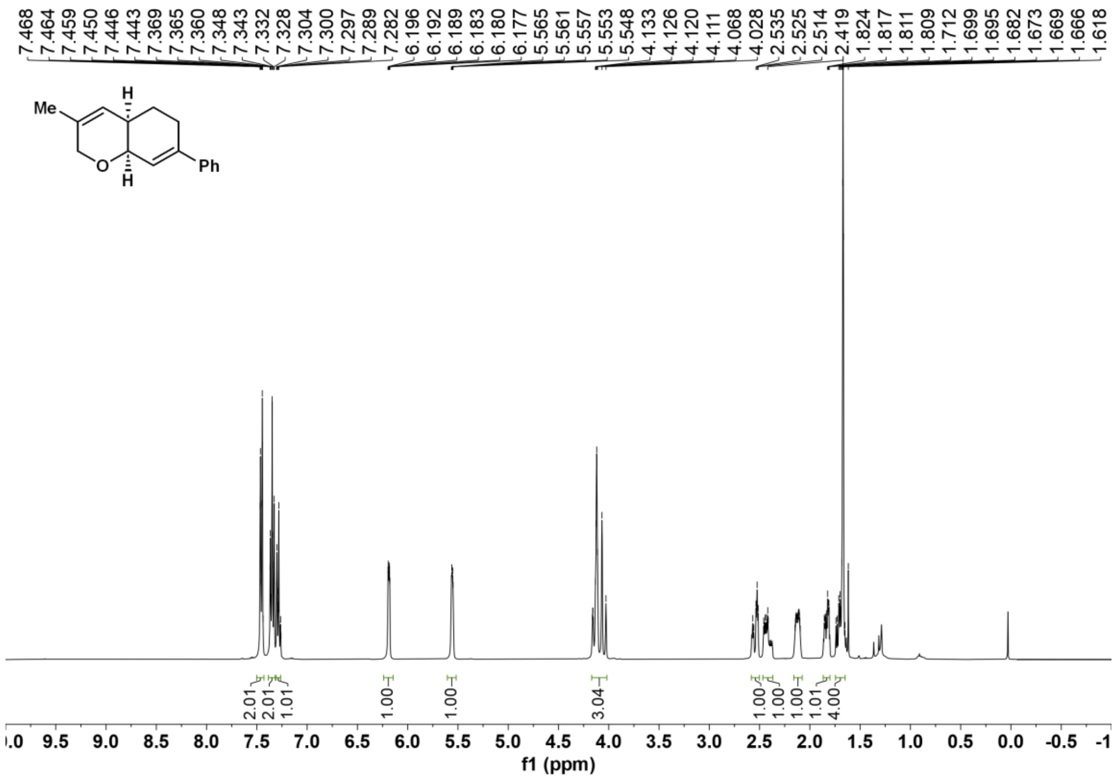

^{13}C **NMR of 3al** (100 MHz, $CDCl_3$)

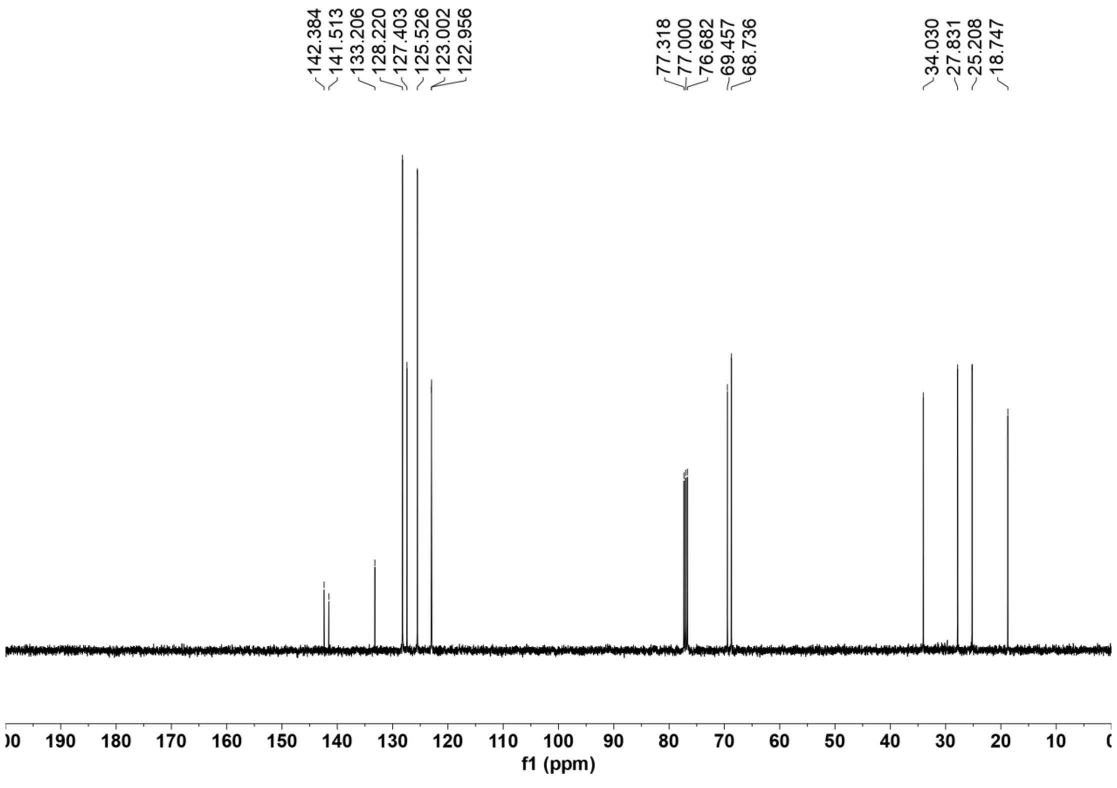

^{1}H NMR of 3am (400 MHz, $CDCl_3$)

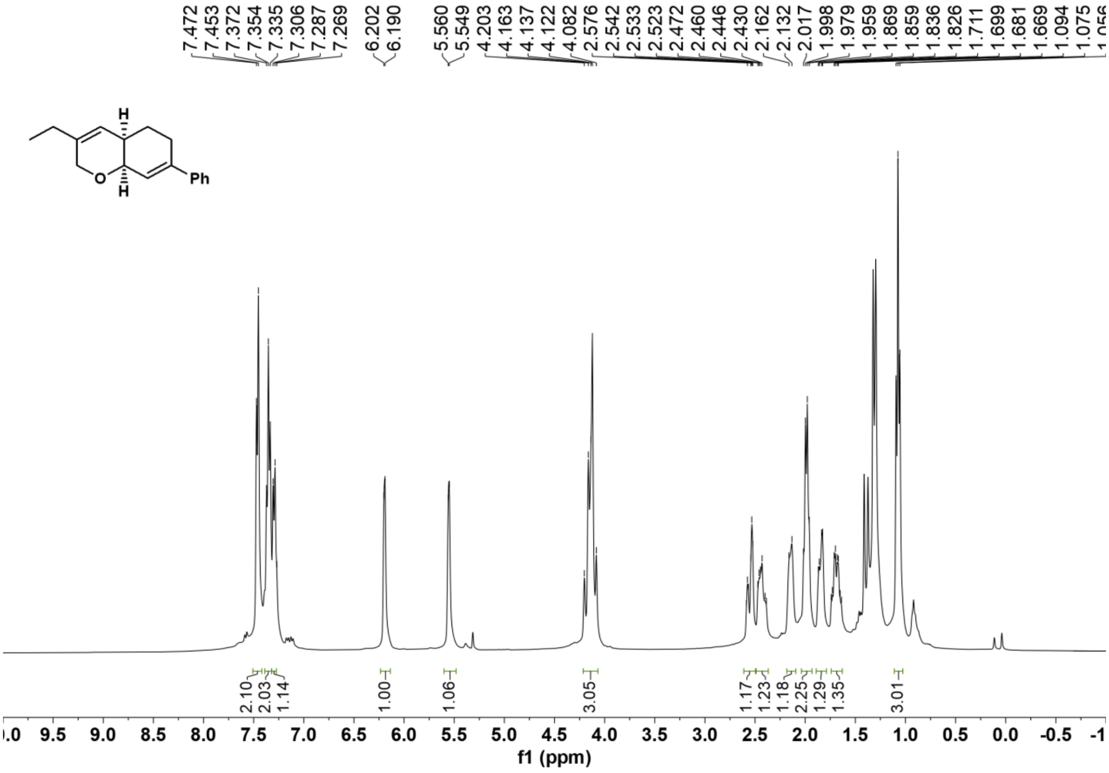

^{13}C NMR of 3am (100 MHz, $CDCl_3$)

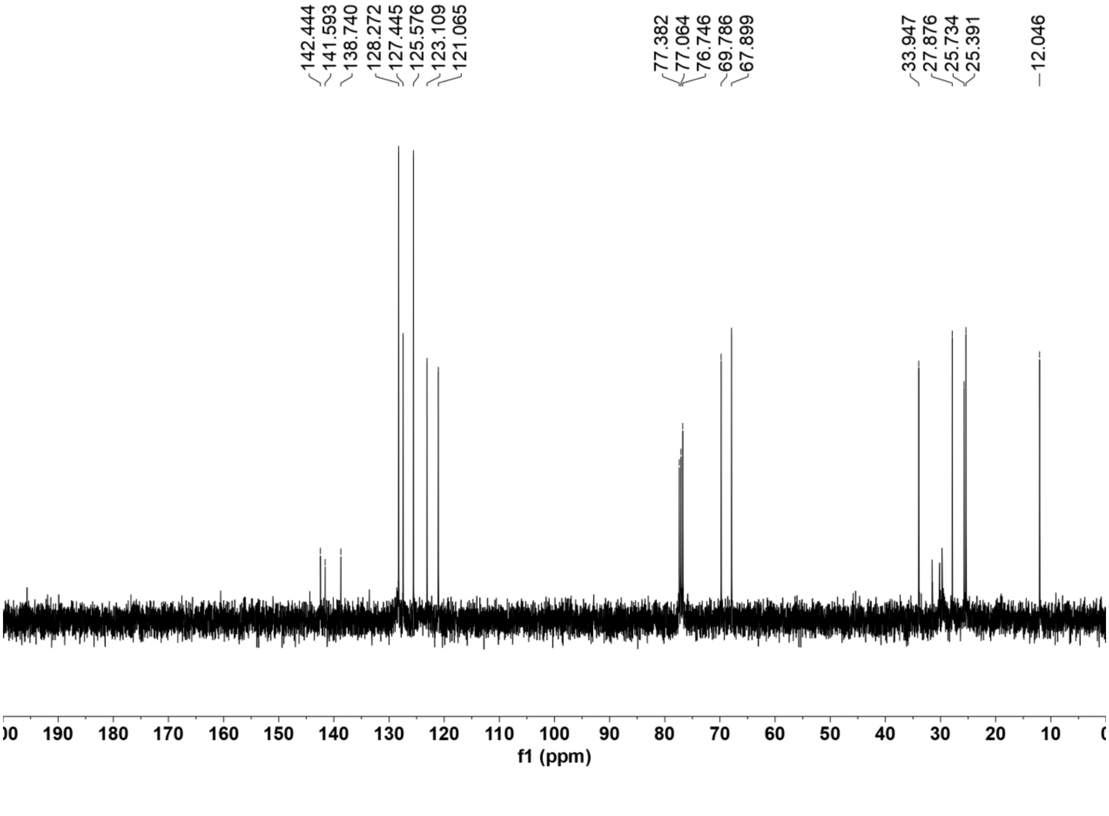

^{1}H NMR of 3an (400 MHz, $CDCl_3$)

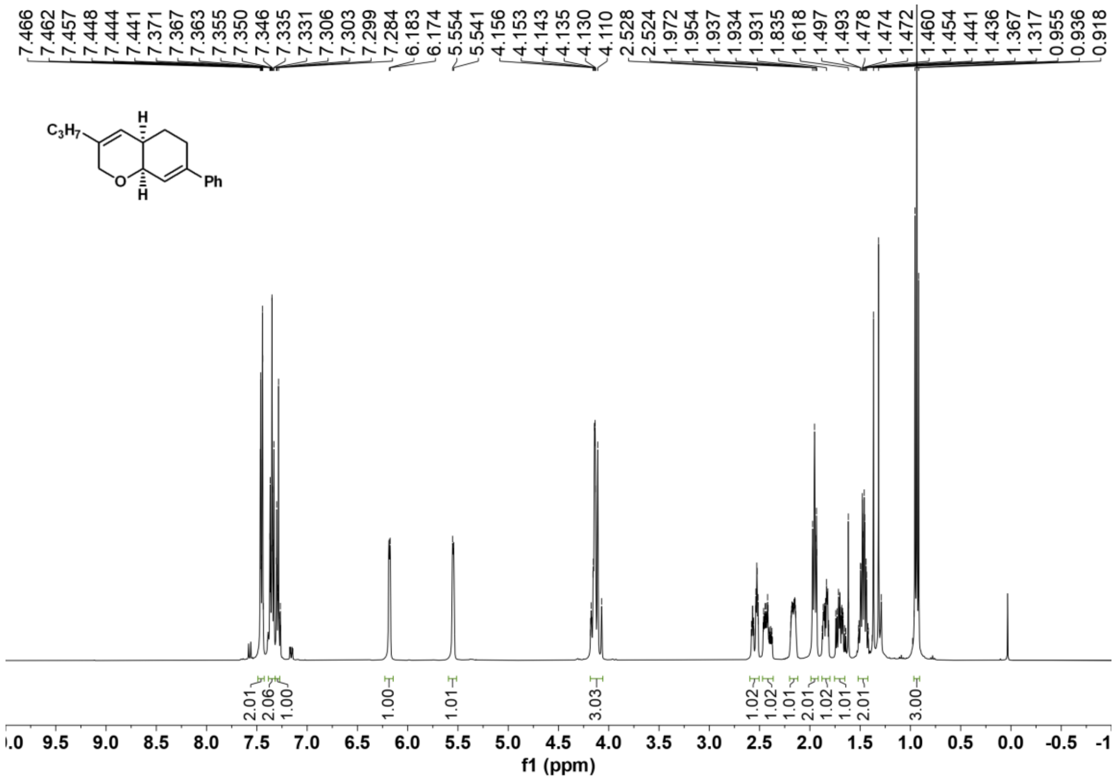

^{13}C NMR of 3an (100 MHz, $CDCl_3$)

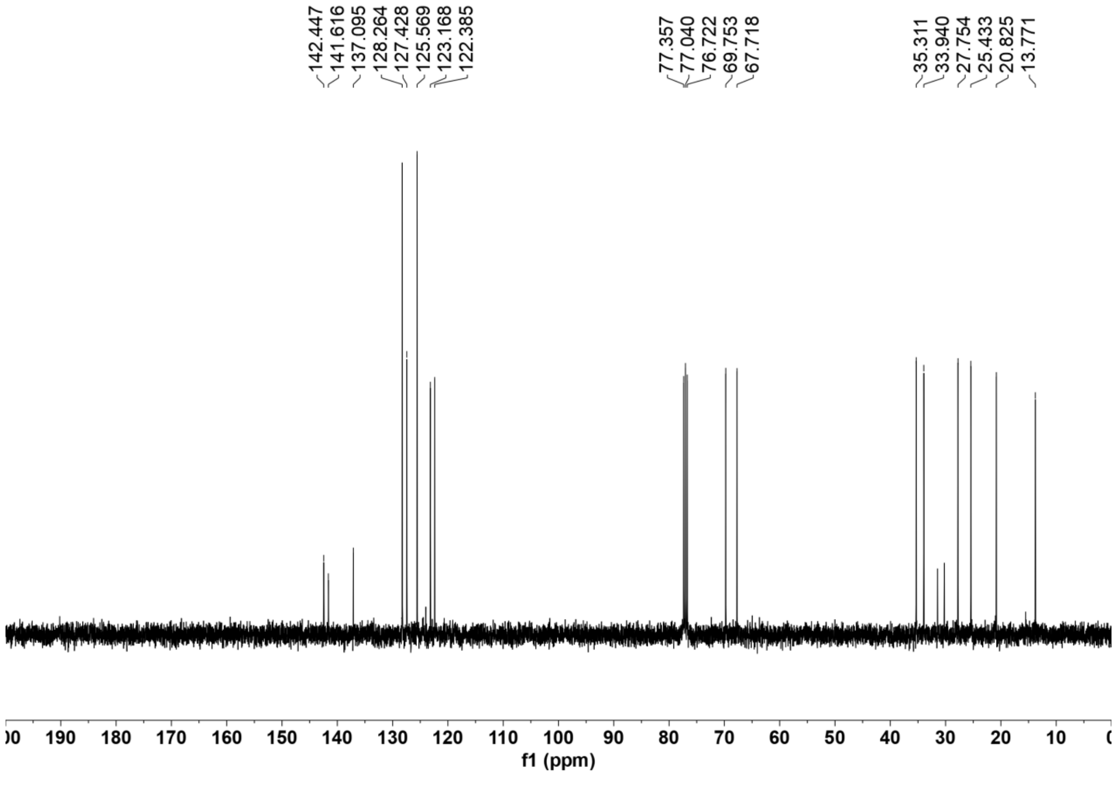

^{1}H NMR of 3ao (400 MHz, $CDCl_3$)

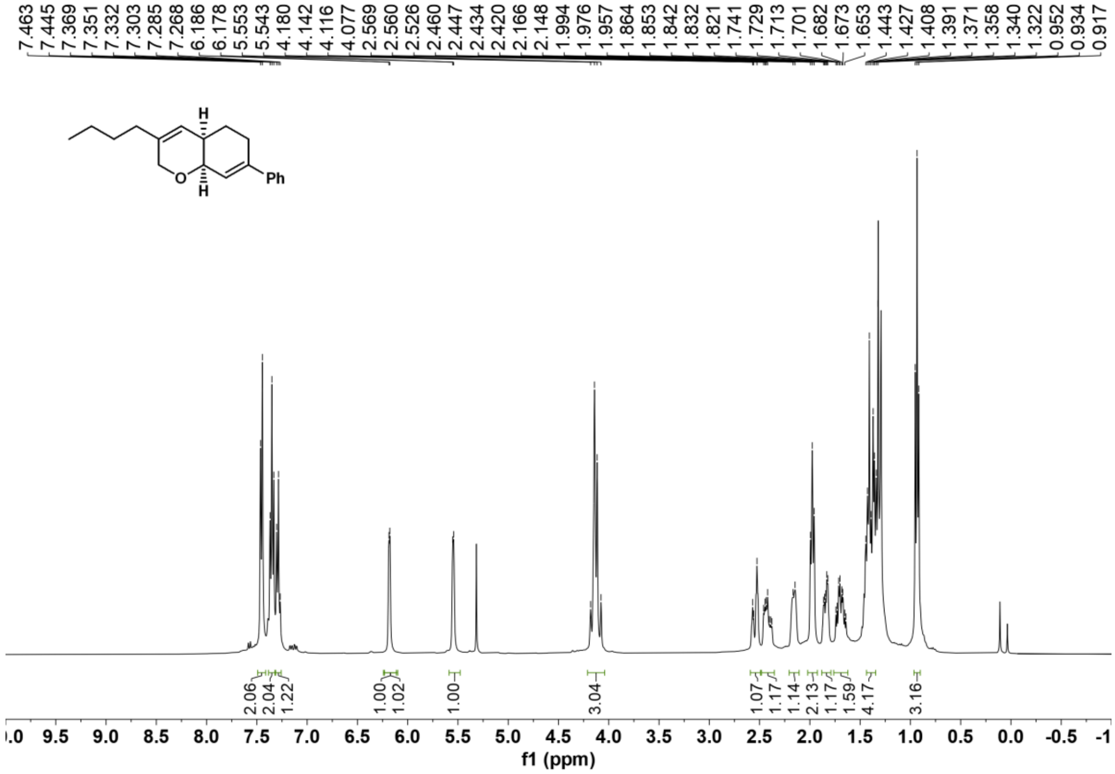

^{13}C NMR of 3ao (100 MHz, $CDCl_3$)

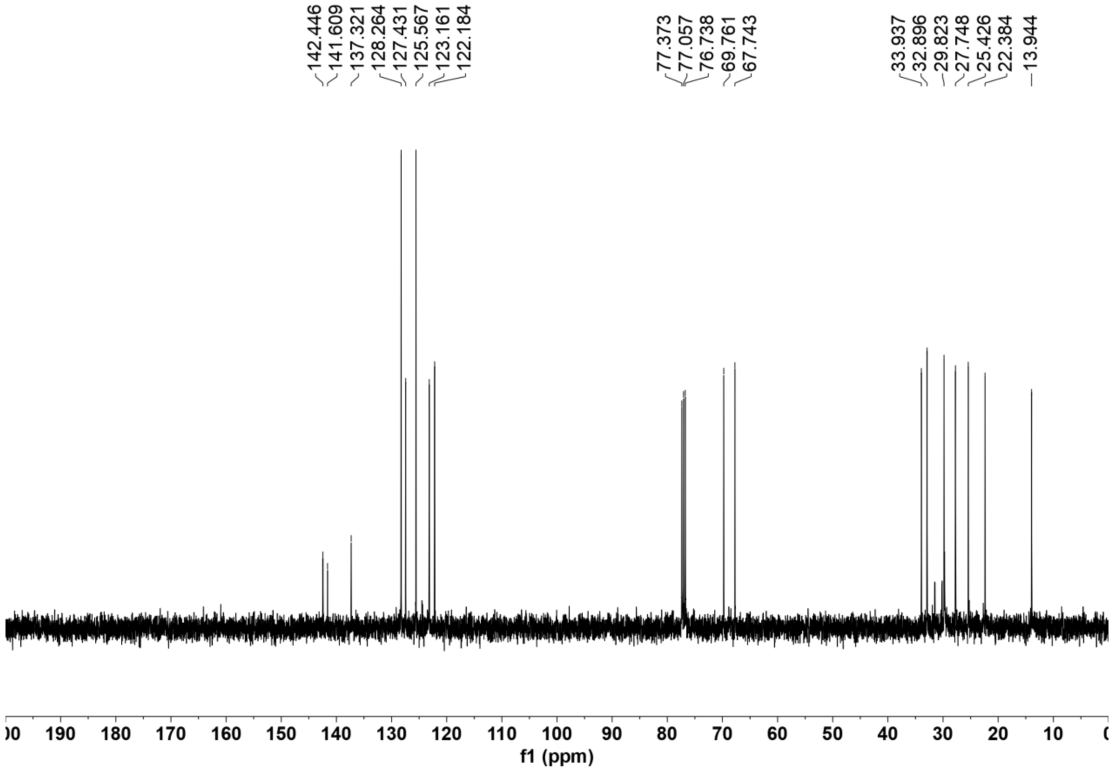

^{1}H NMR of 3ap (400 MHz, $CDCl_3$)

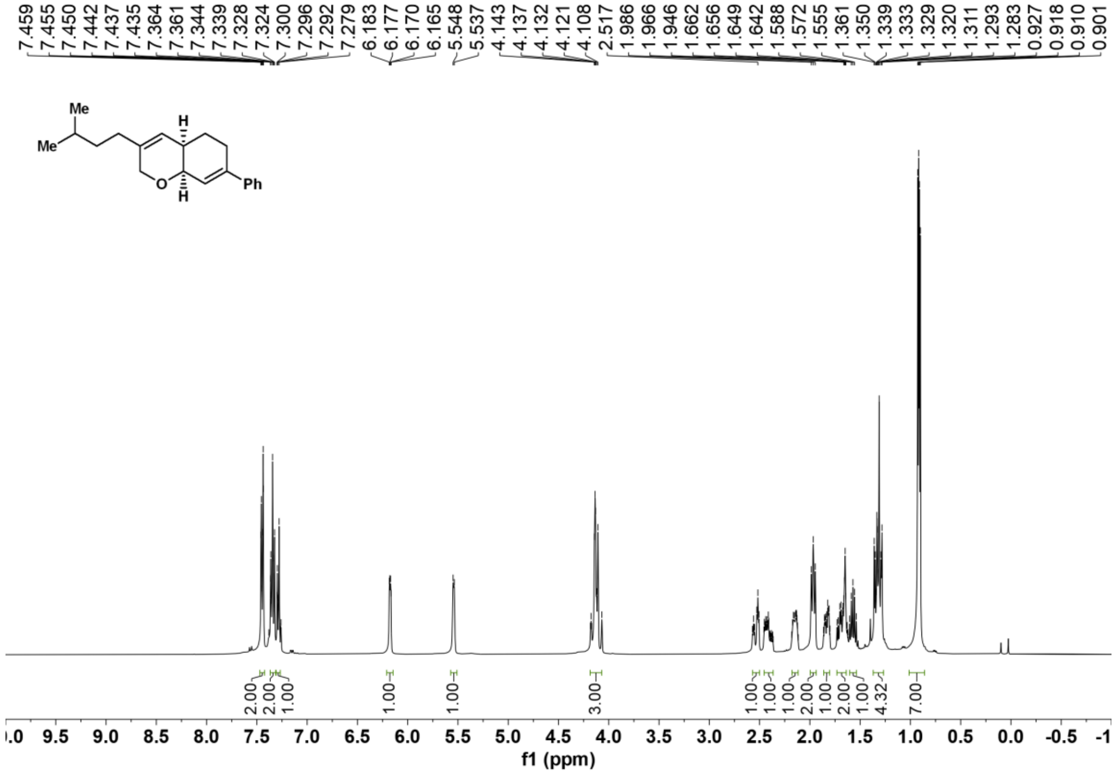

^{13}C NMR of 3ap (100 MHz, $CDCl_3$)

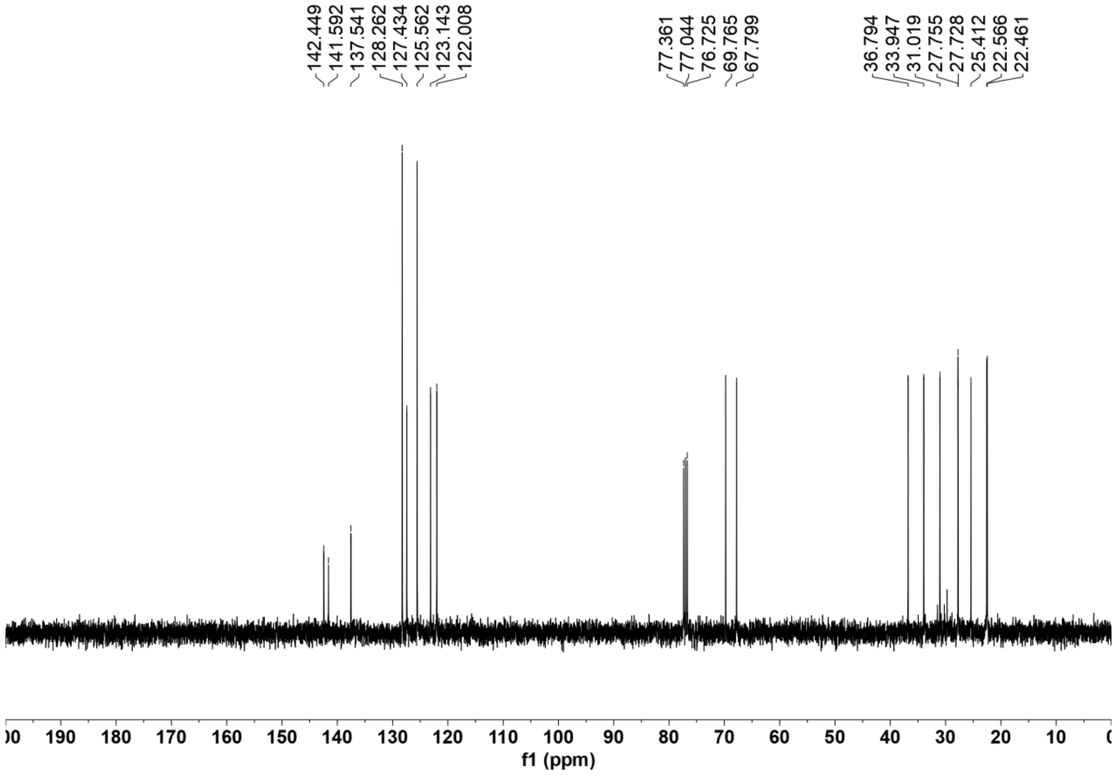

^{1}H NMR of 3aq (400 MHz, $CDCl_3$)

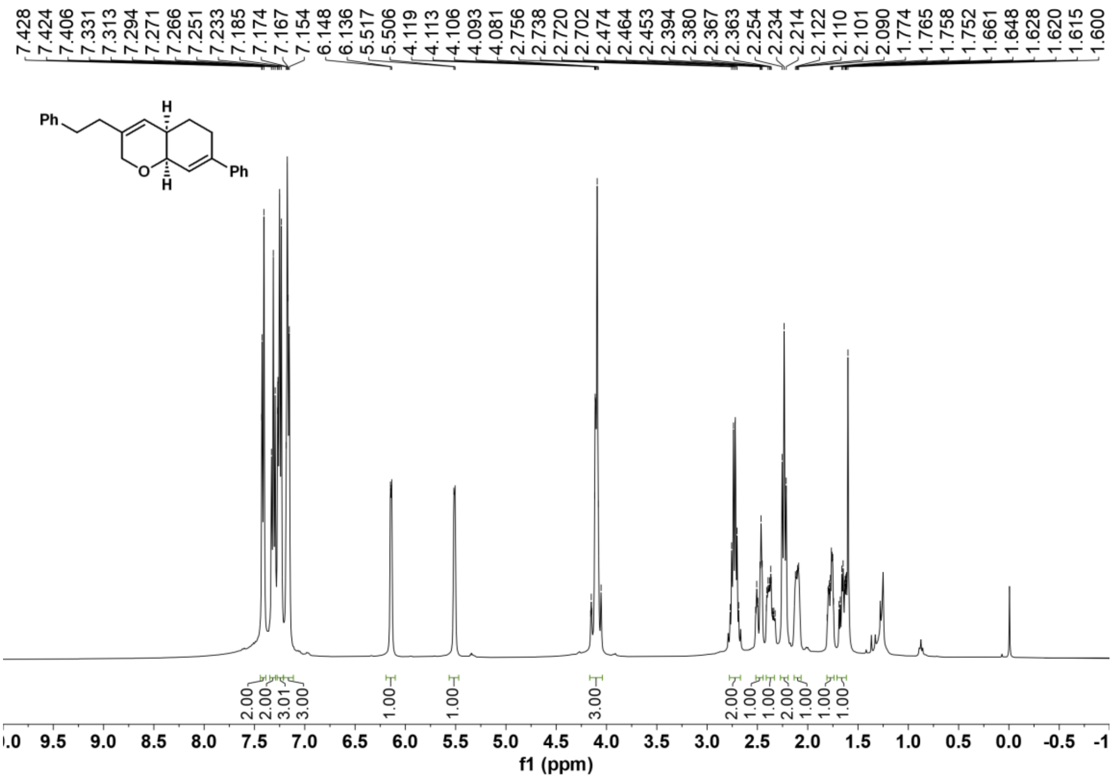

^{13}C NMR of 3aq (100 MHz, $CDCl_3$)

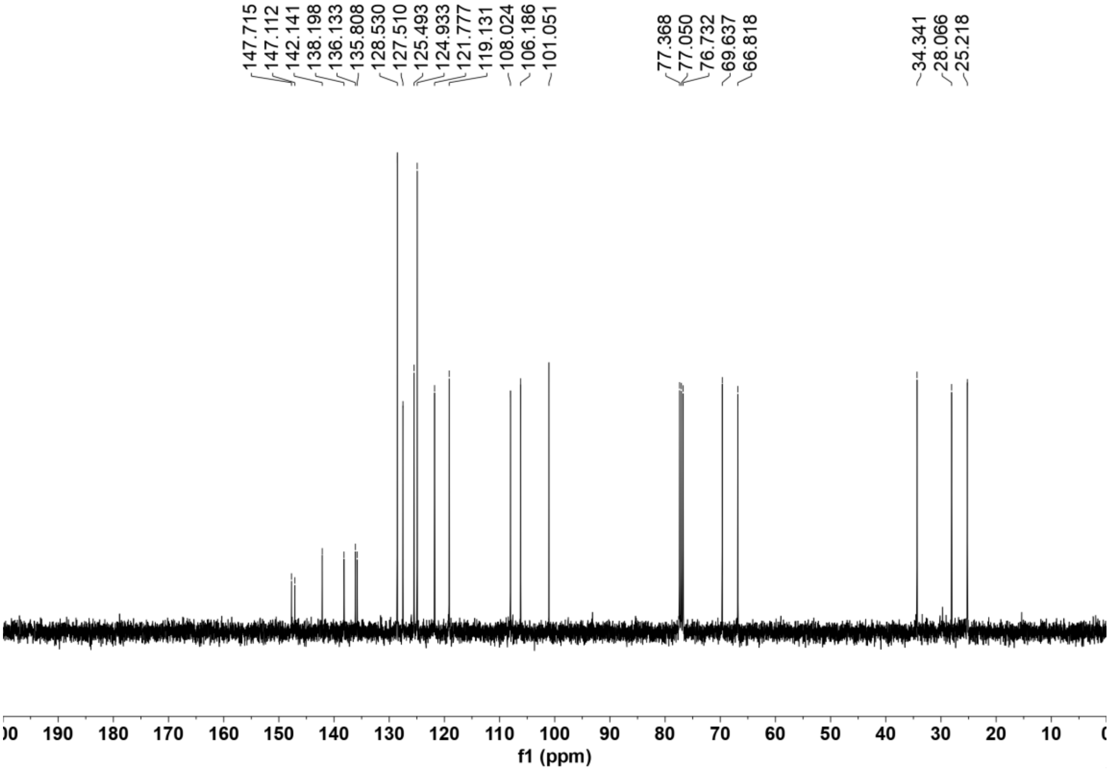

^{1}H NMR of 3ar (400 MHz, $CDCl_3$)

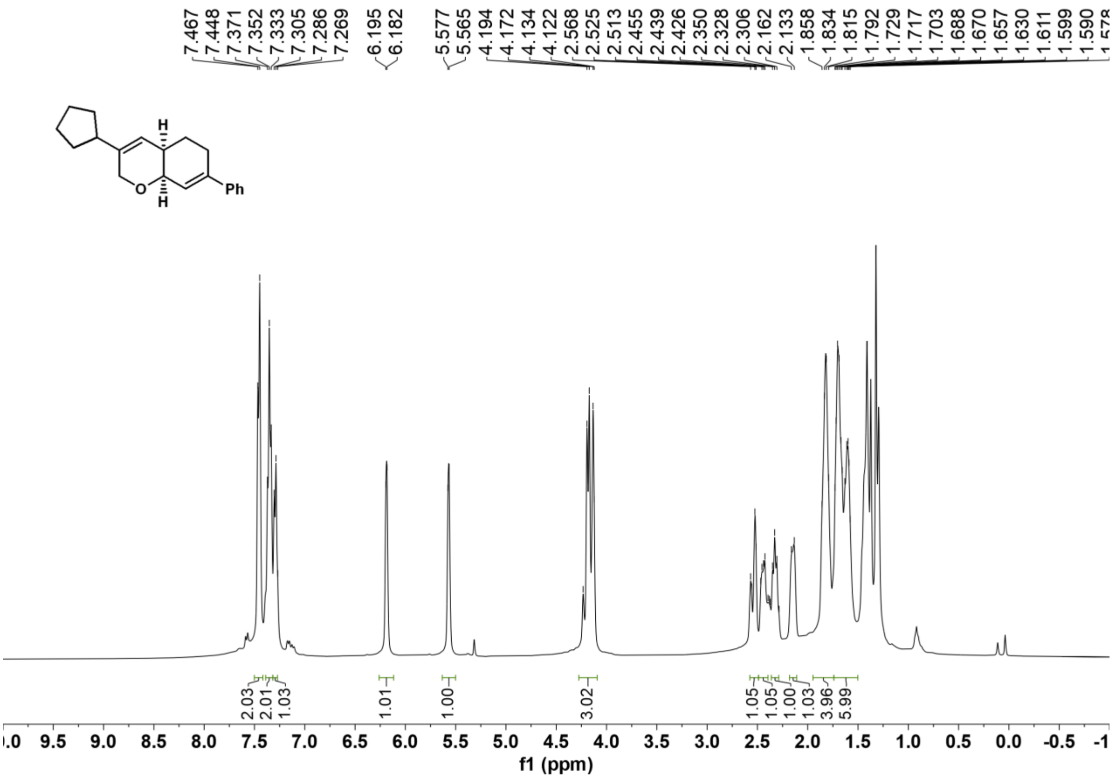

^{13}C NMR of 3ar (100 MHz, $CDCl_3$)

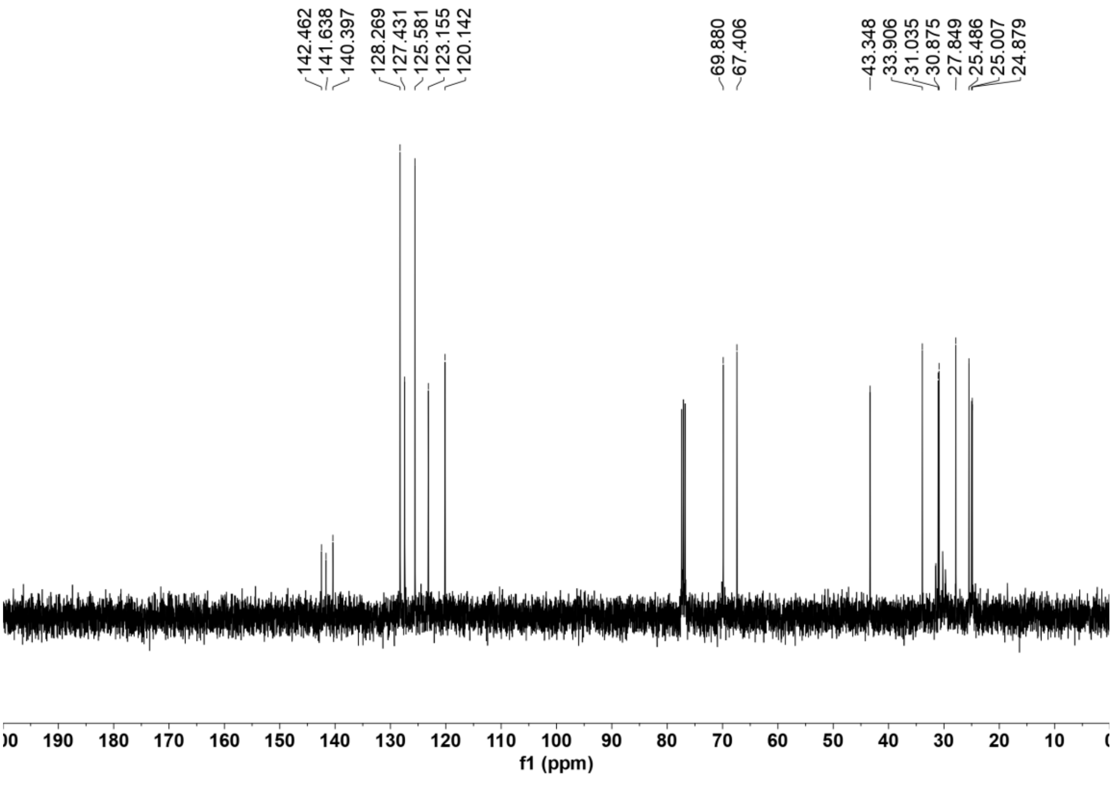

^{1}H NMR of 3as (400 MHz, $CDCl_3$)

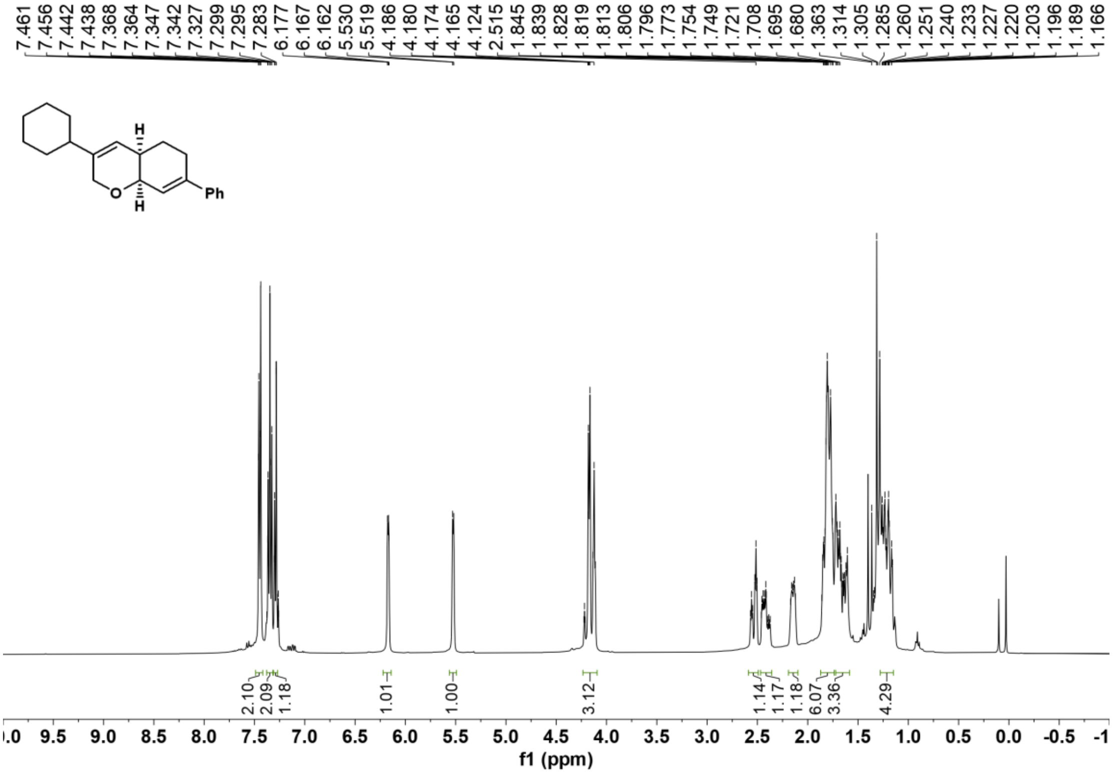

^{13}C NMR of 3as (100 MHz, $CDCl_3$)

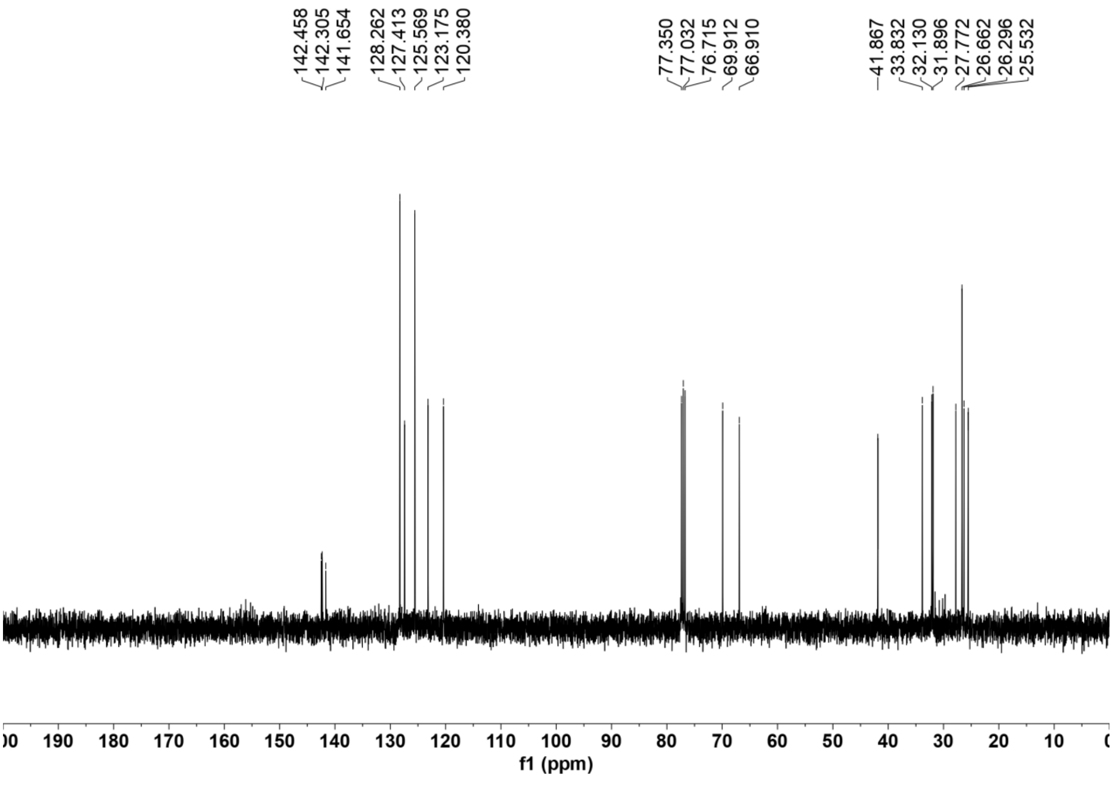

^{1}H NMR of 3at (400 MHz, $CDCl_3$)

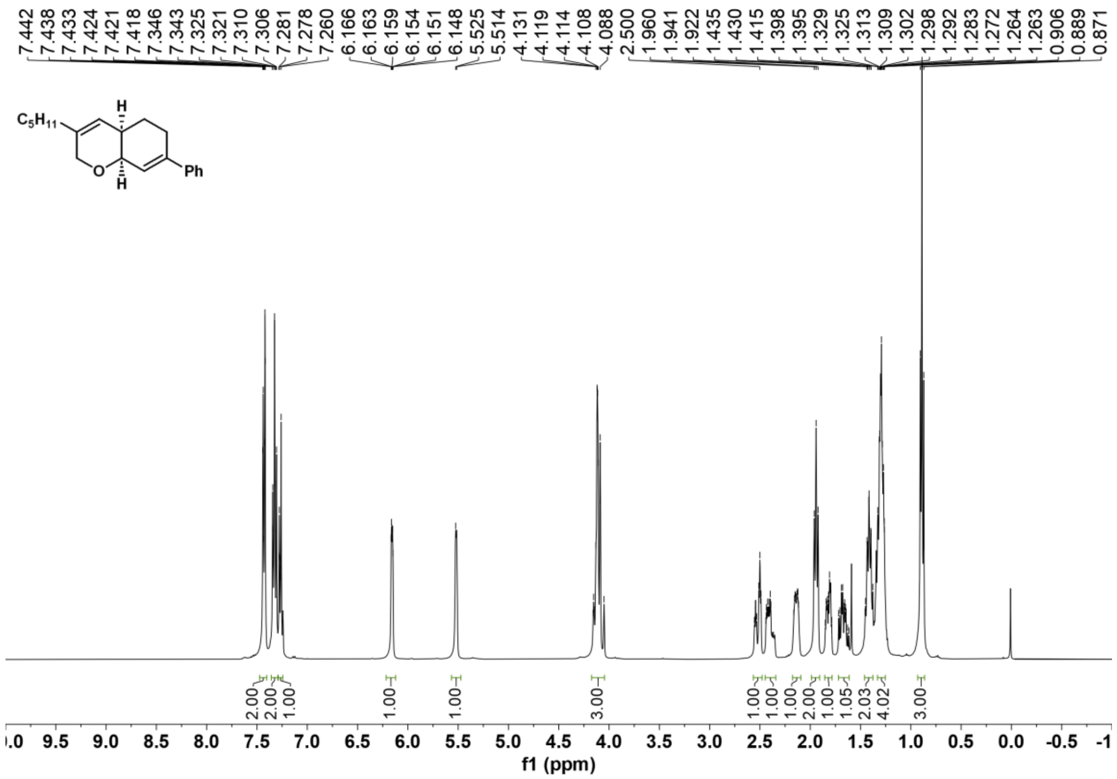

^{13}C NMR of 3at (100 MHz, $CDCl_3$)

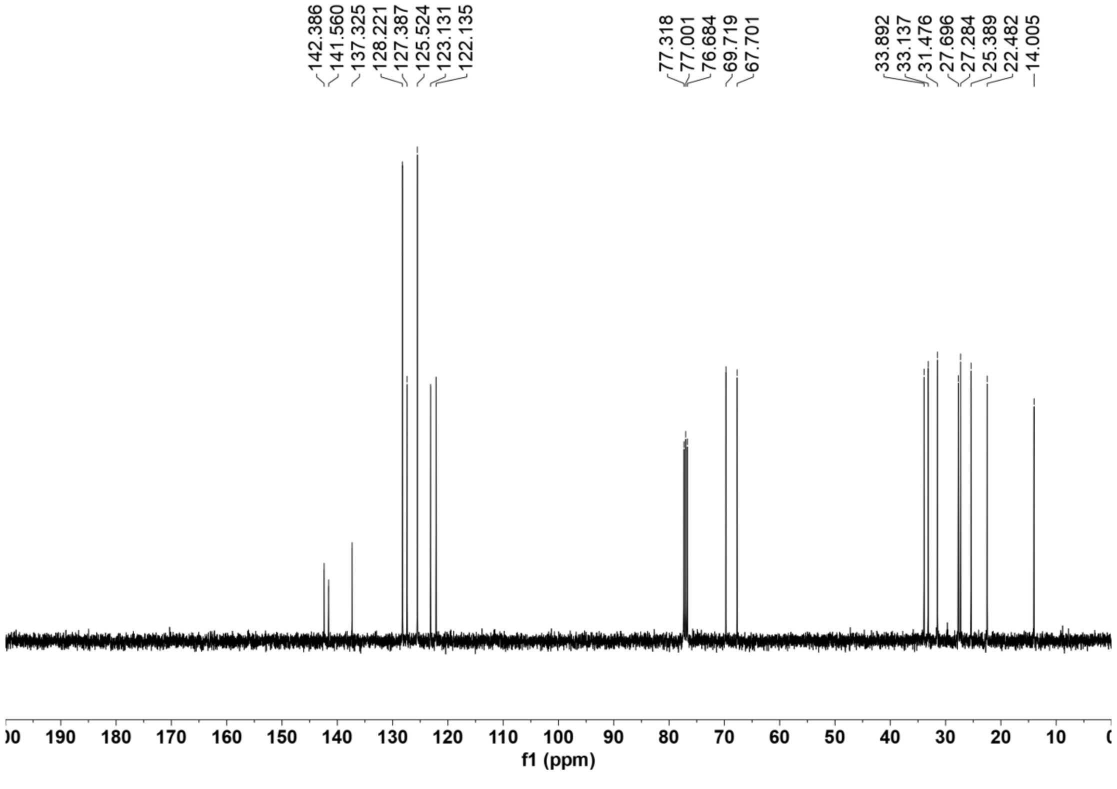

^{1}H NMR of 3au (400 MHz, $CDCl_3$)

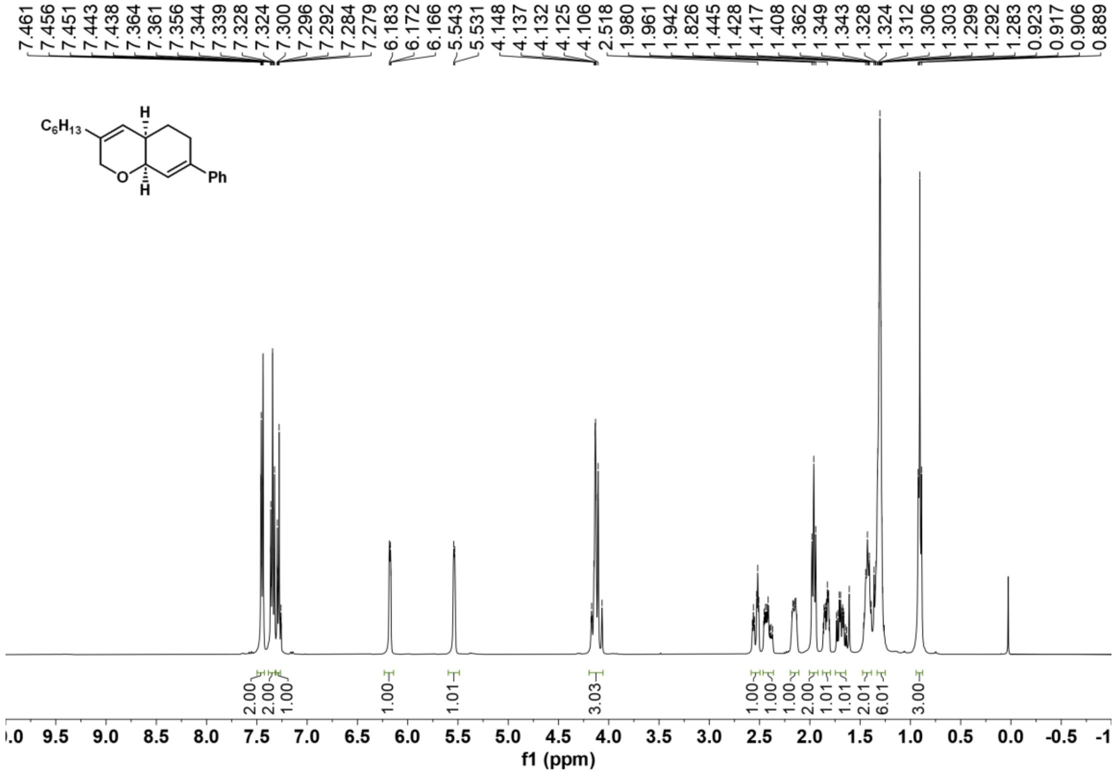

^{13}C NMR of 3au (100 MHz, $CDCl_3$)

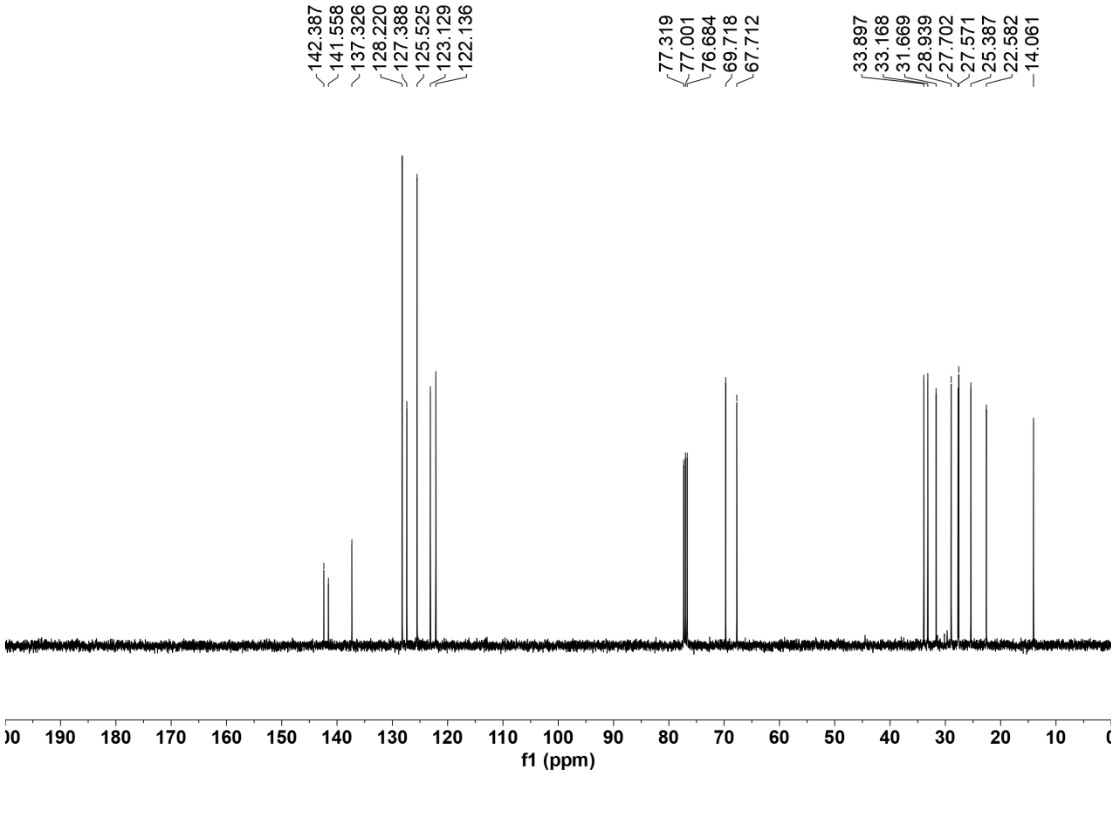

^{1}H NMR of 3av (400 MHz, $CDCl_3$)

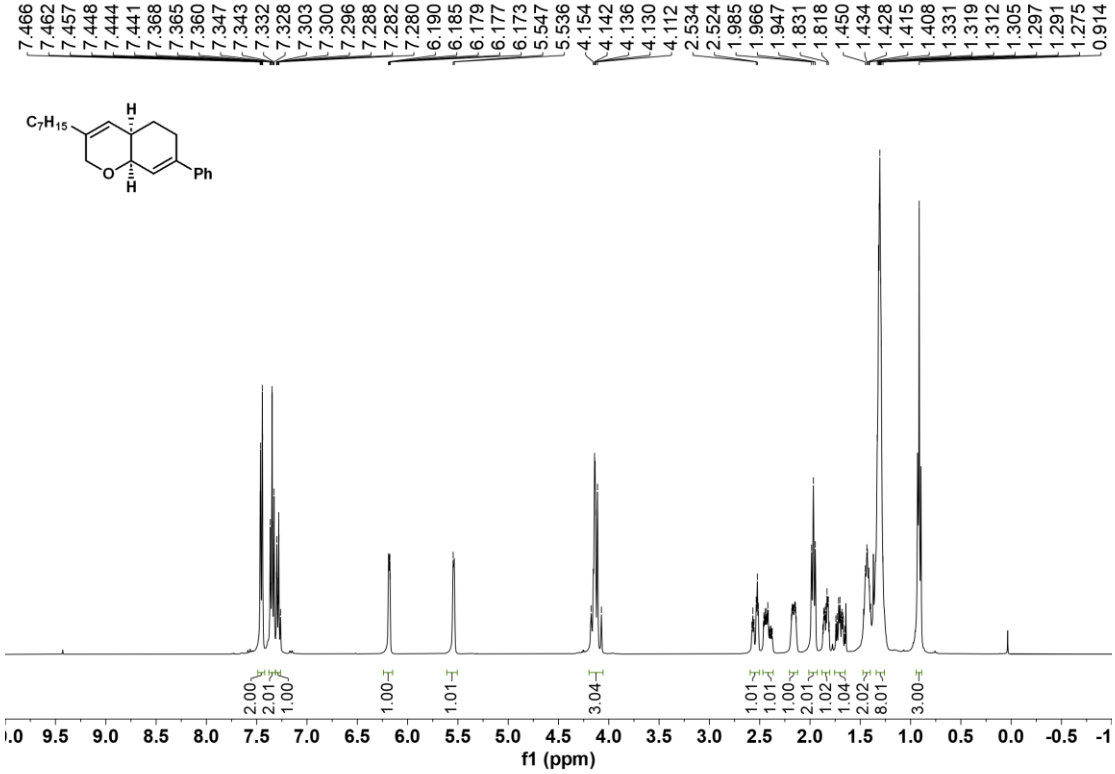

^{13}C NMR of 3av (100 MHz, $CDCl_3$)

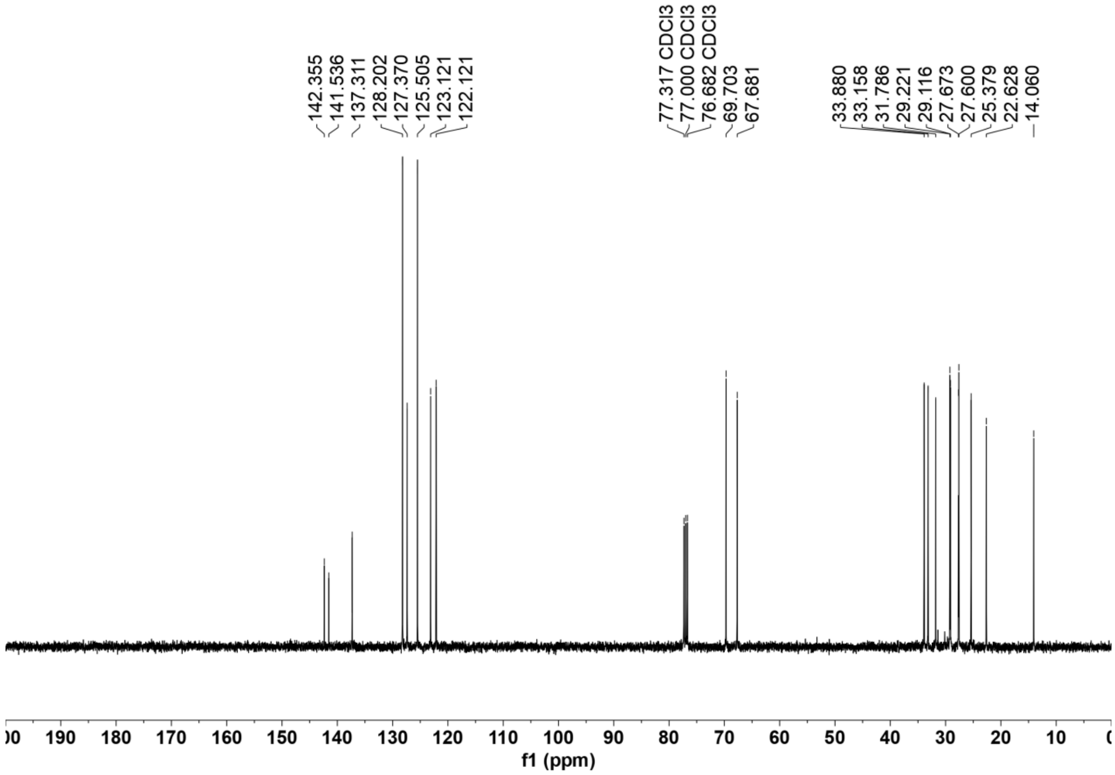

^{1}H NMR of 3aw (400 MHz, $CDCl_3$)

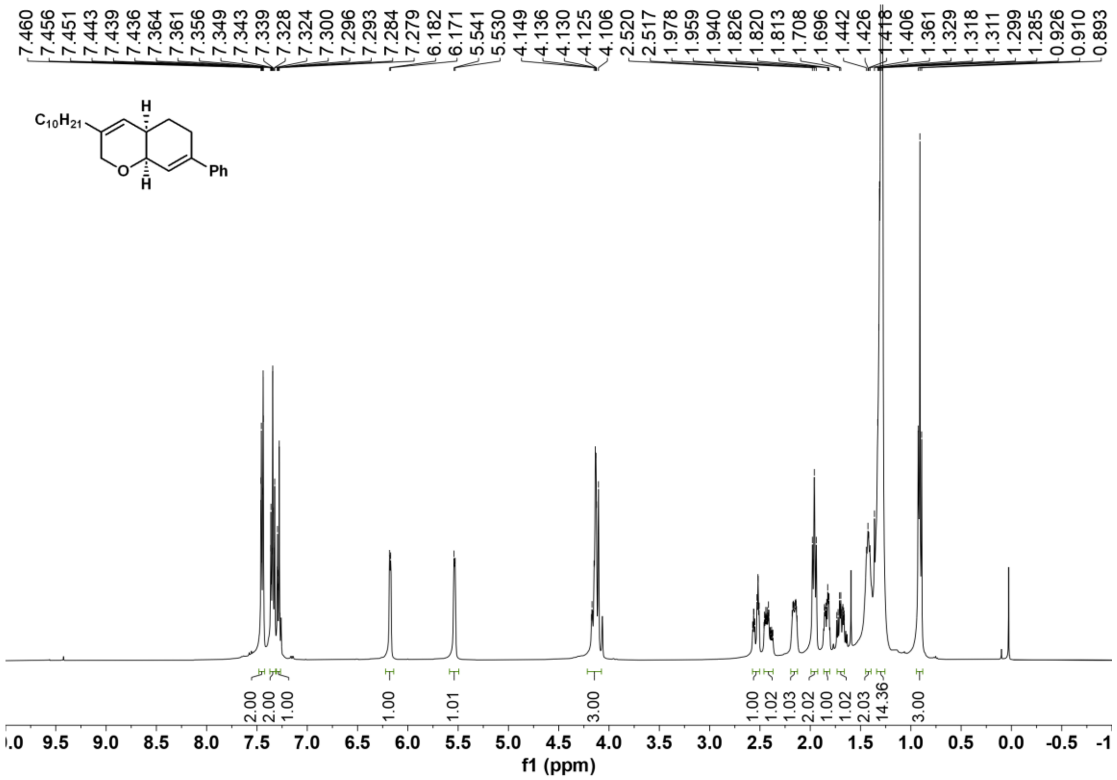

^{13}C NMR of 3aw (100 MHz, $CDCl_3$)

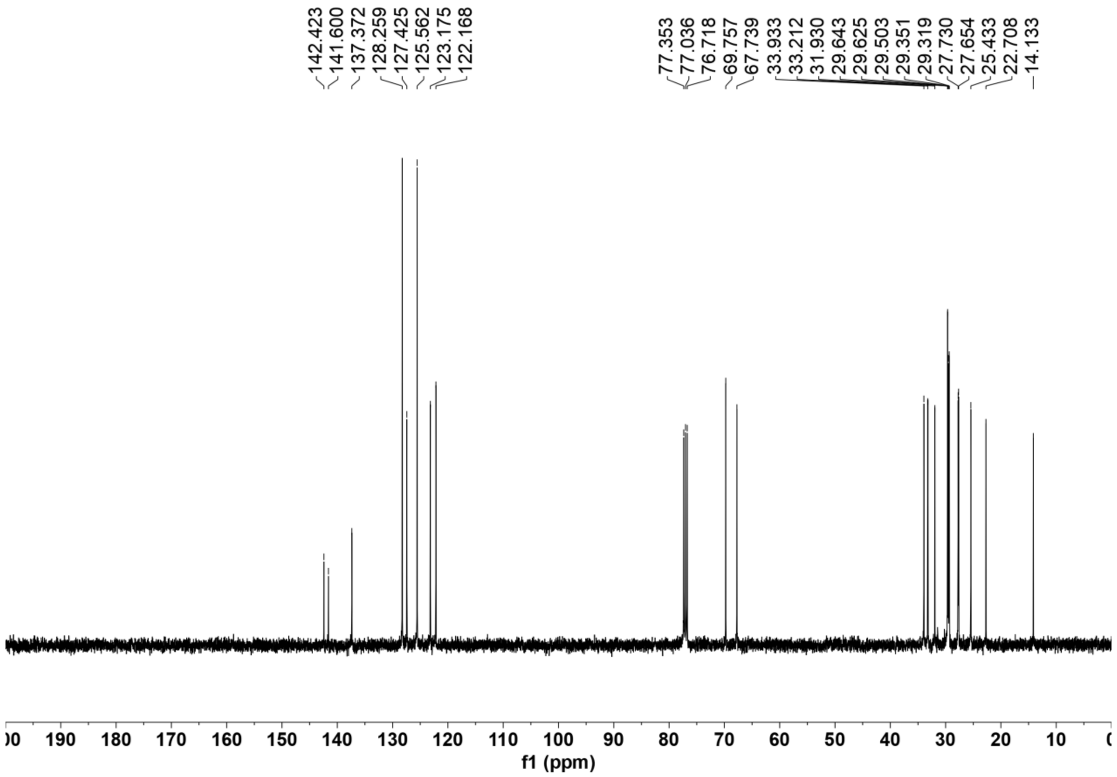

^{1}H NMR of 3ax (400 MHz, $CDCl_3$)

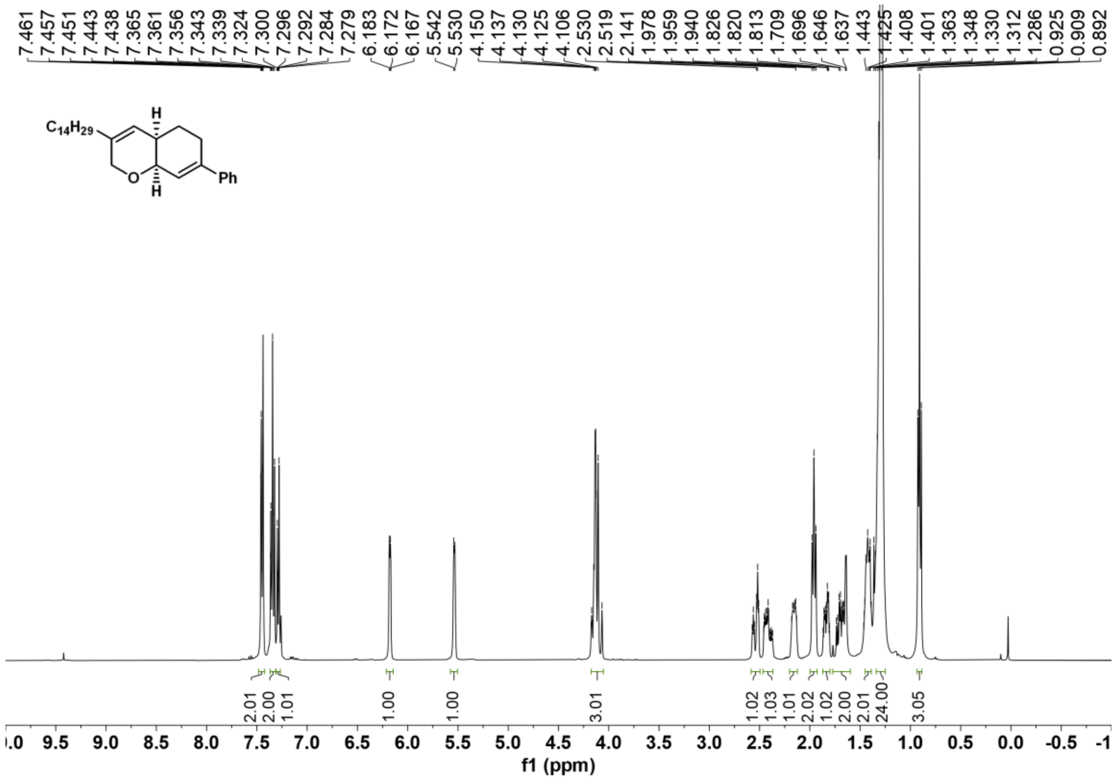

^{13}C NMR of 3ax (100 MHz, $CDCl_3$)

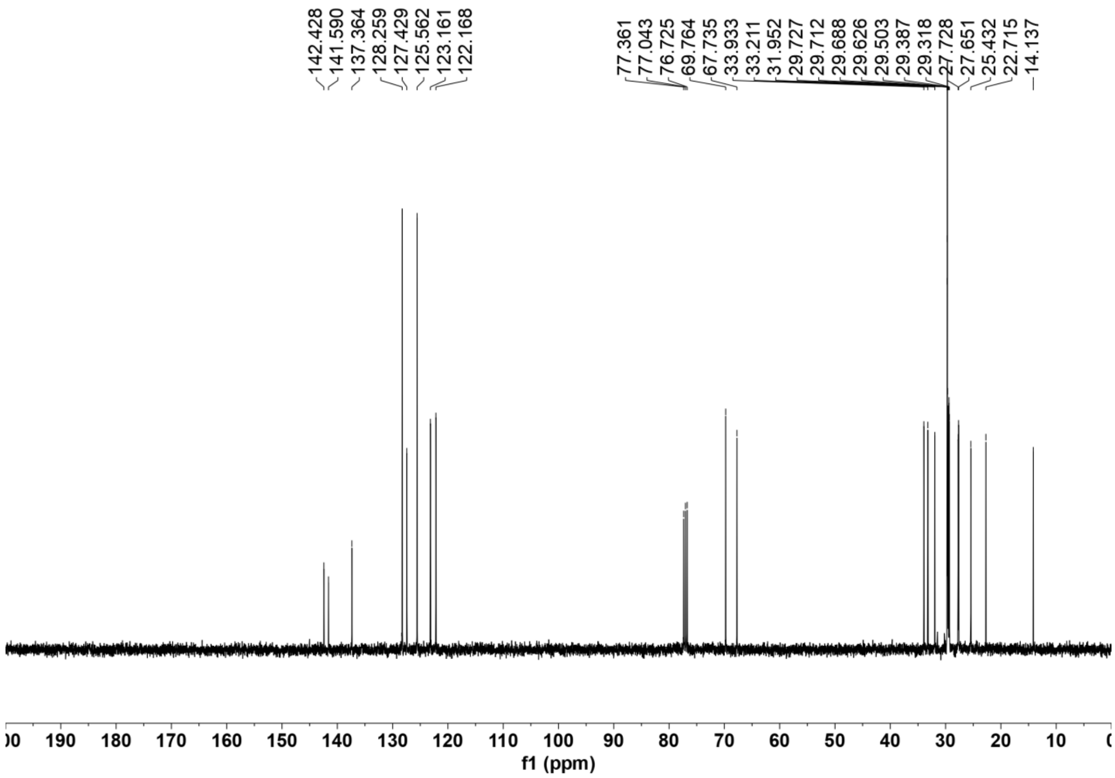

^{1}H NMR of 3ba (400 MHz, $CDCl_3$)

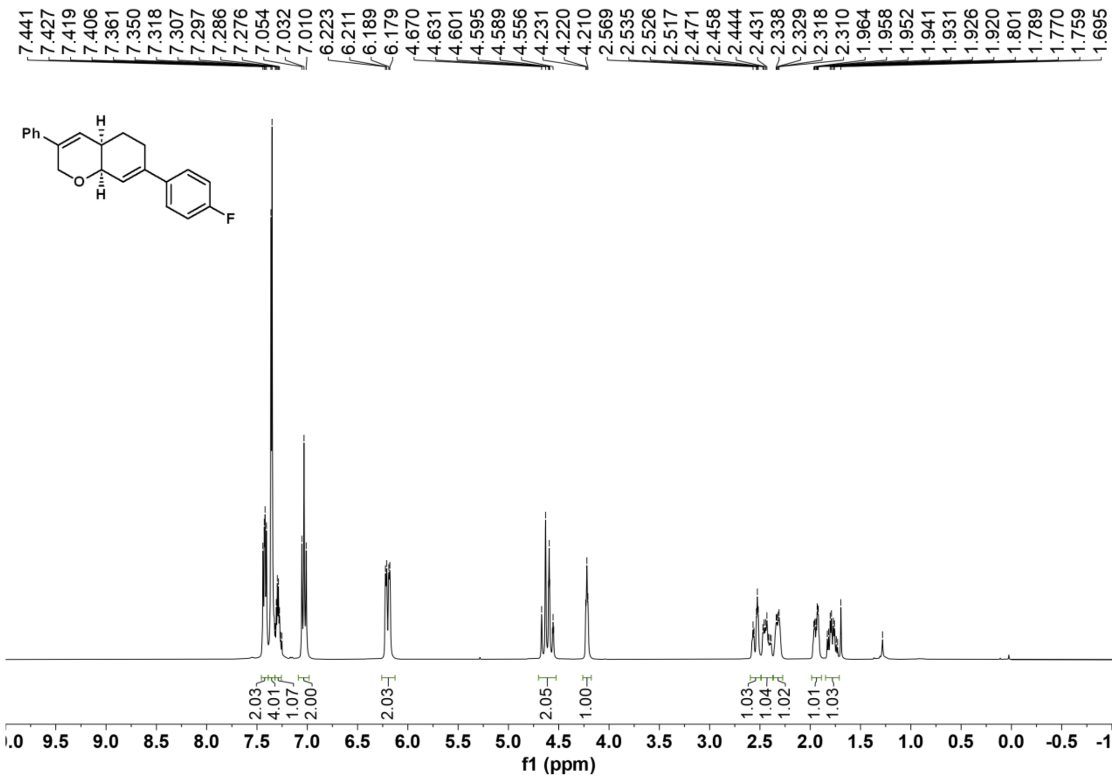

^{13}C NMR of 3ba (100 MHz, $CDCl_3$)

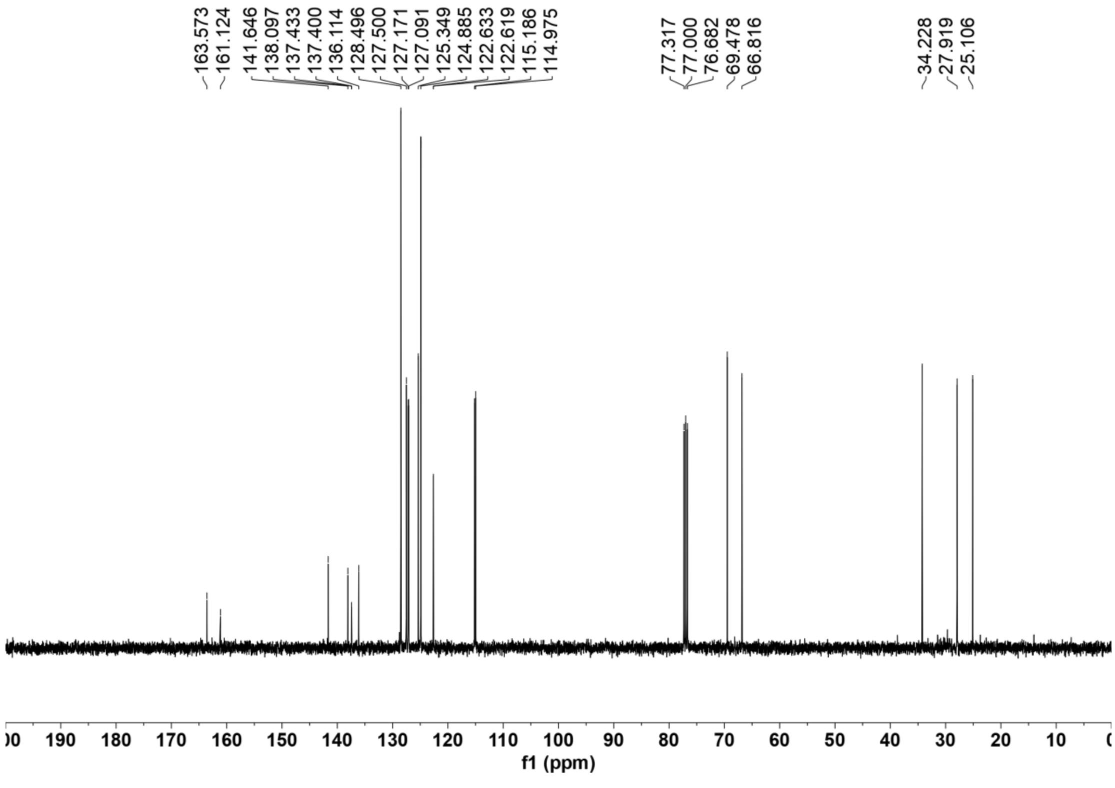

^{19}F NMR of 3ba (376 MHz, $CDCl_3$)

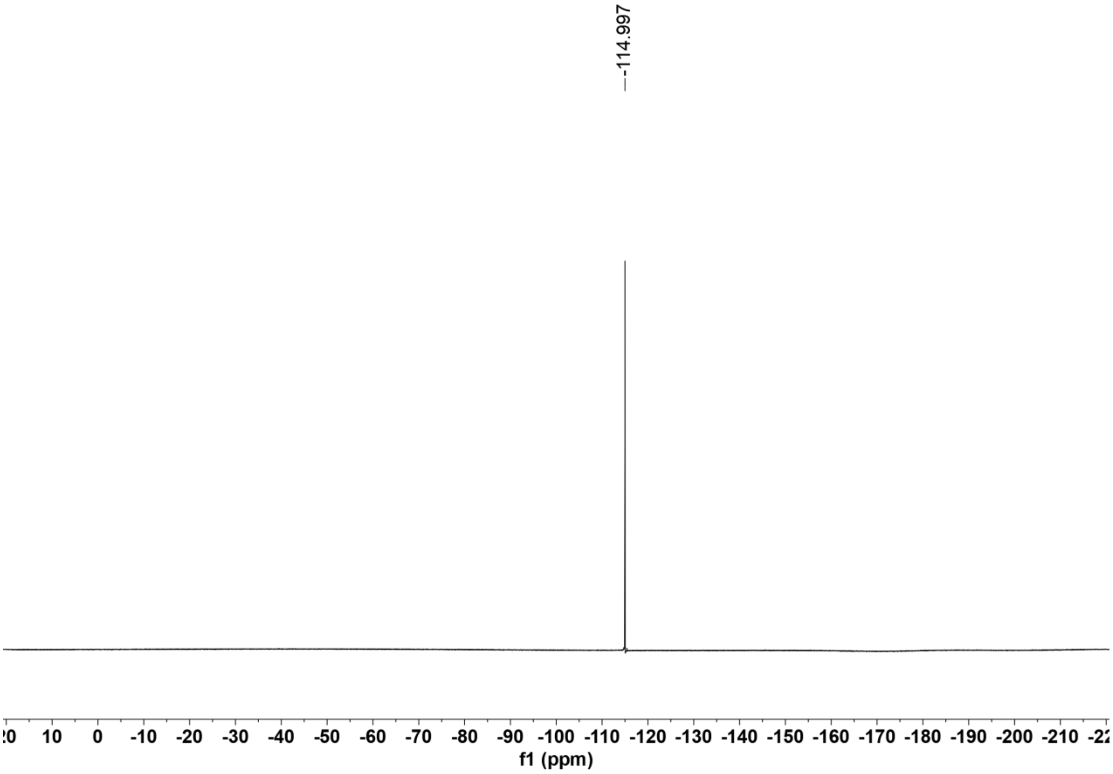

^{1}H NMR of 3bb (400 MHz, $CDCl_3$)

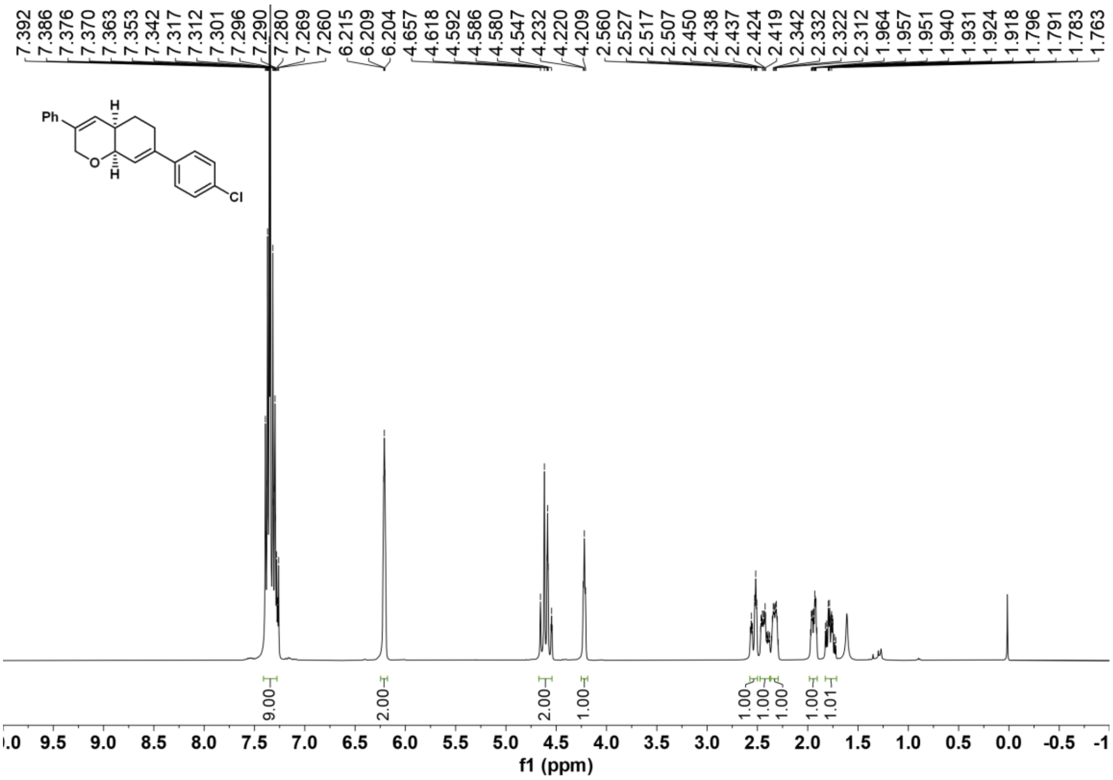

^{13}C NMR of 3bb (100 MHz, $CDCl_3$)

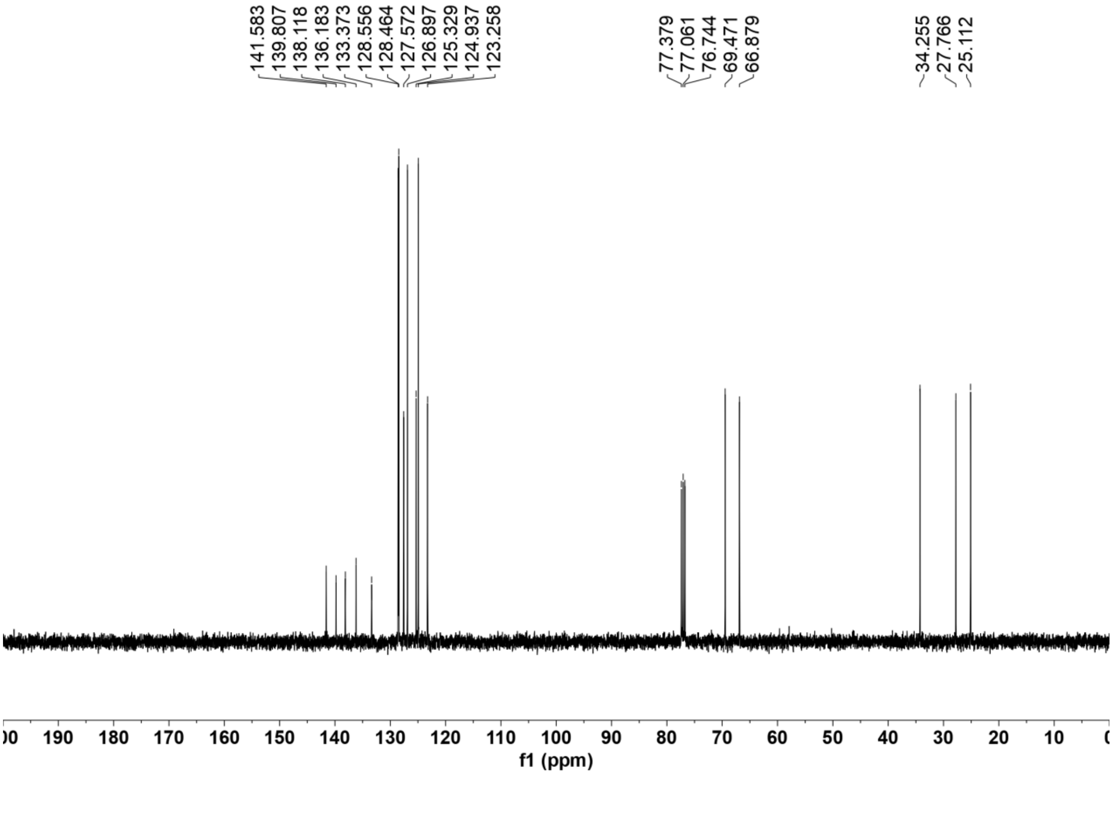

^{1}H NMR of 3bc (400 MHz, $CDCl_3$)

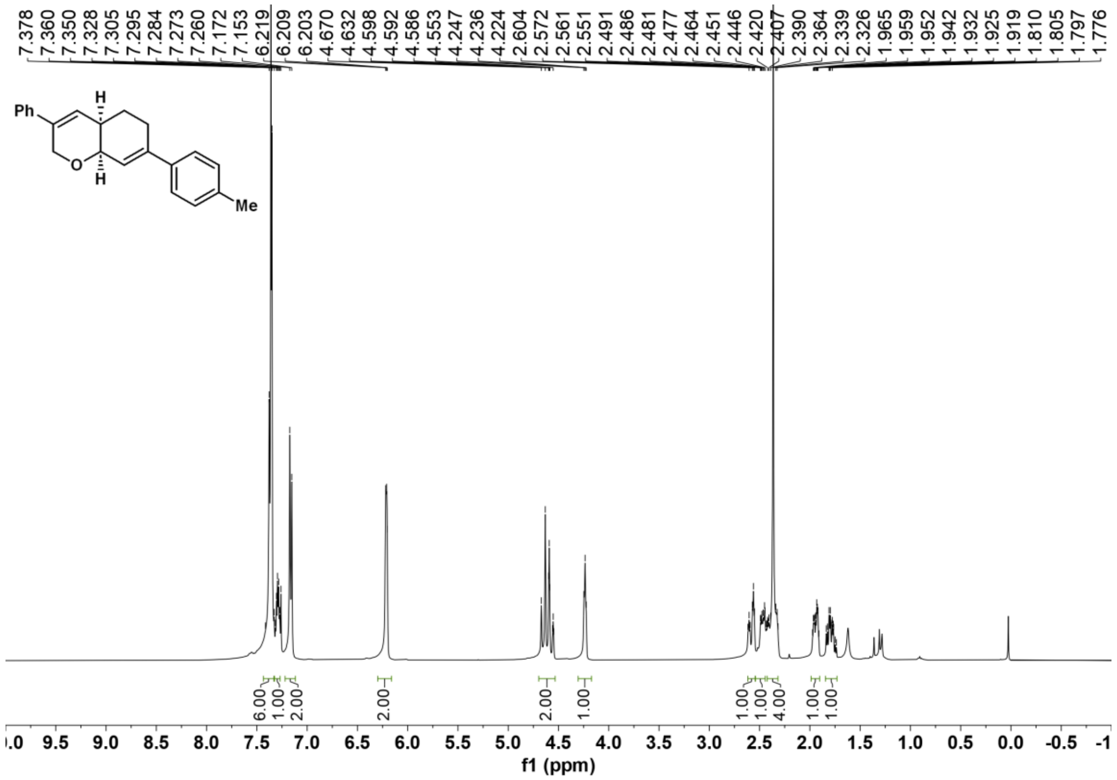

^{13}C NMR of 3bc (100 MHz, $CDCl_3$)

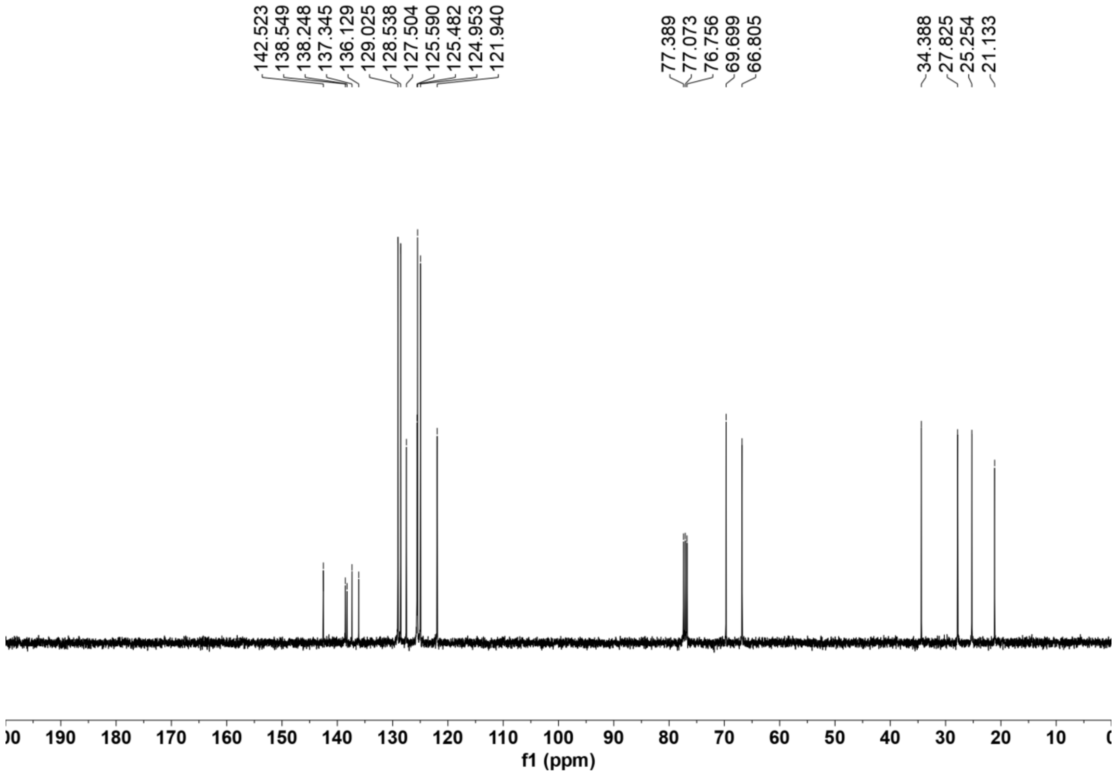

^{1}H NMR of 3bd (400 MHz, $CDCl_3$)

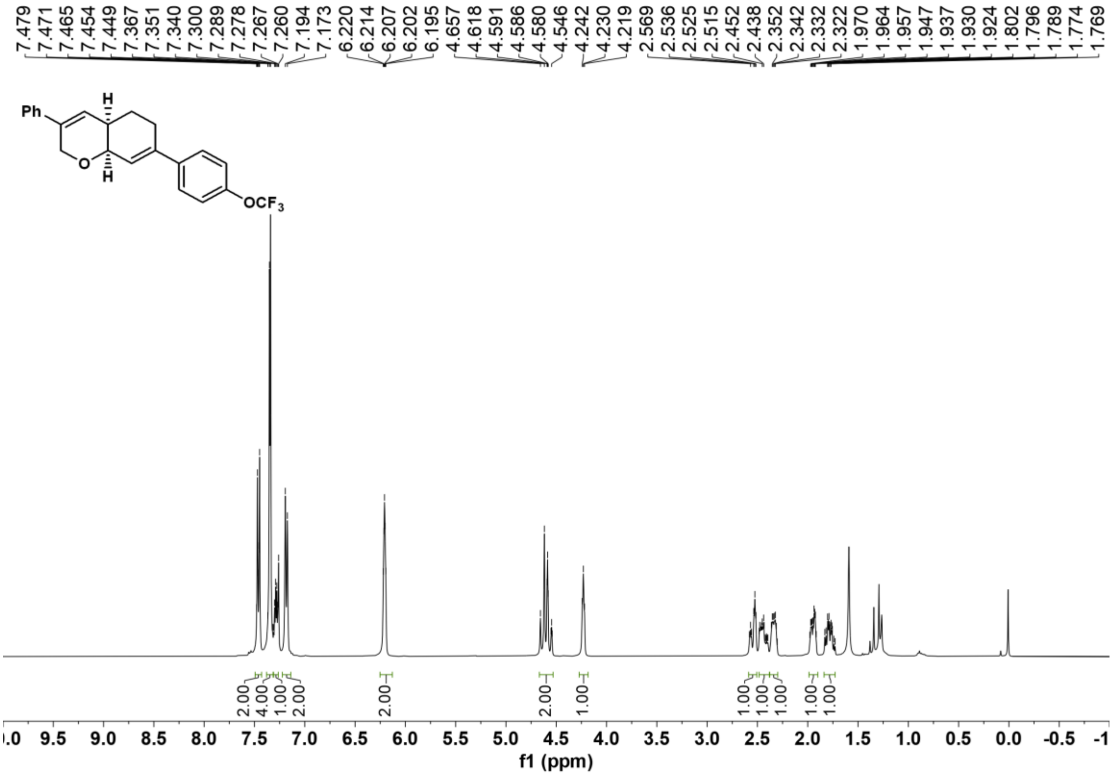

^{13}C NMR of 3bd (100 MHz, $CDCl_3$)

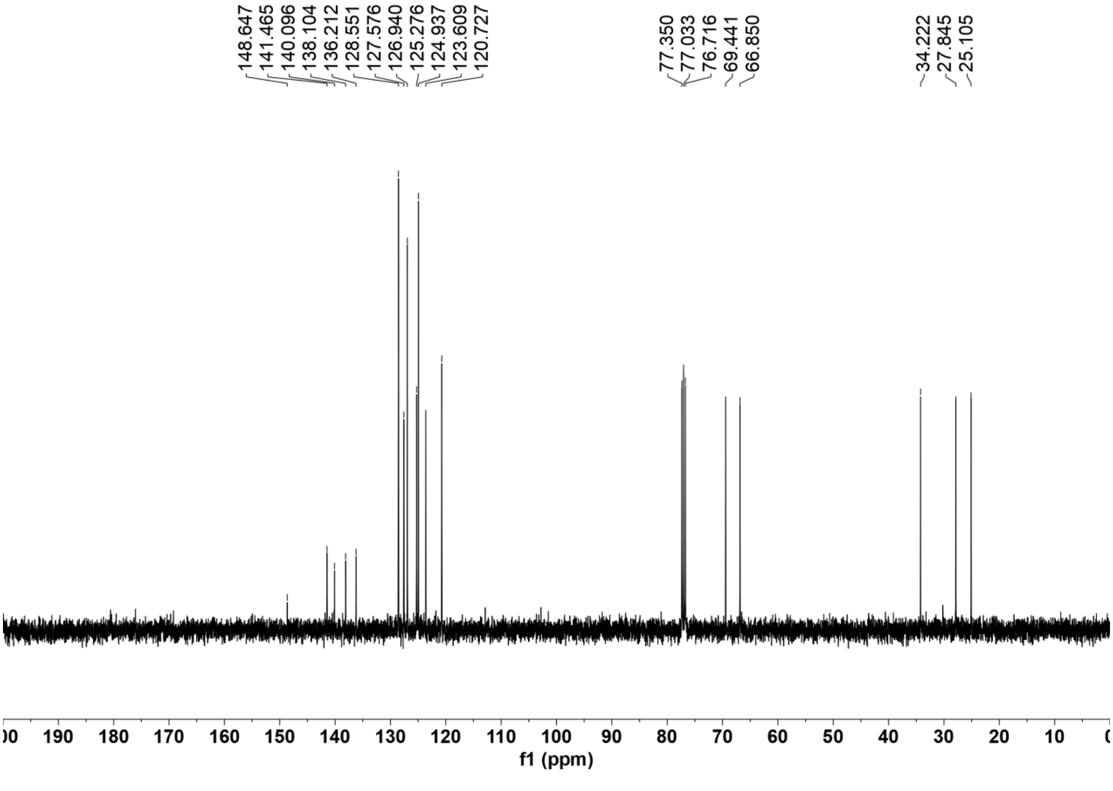

^{19}F NMR of 3bd (376 MHz, $CDCl_3$)

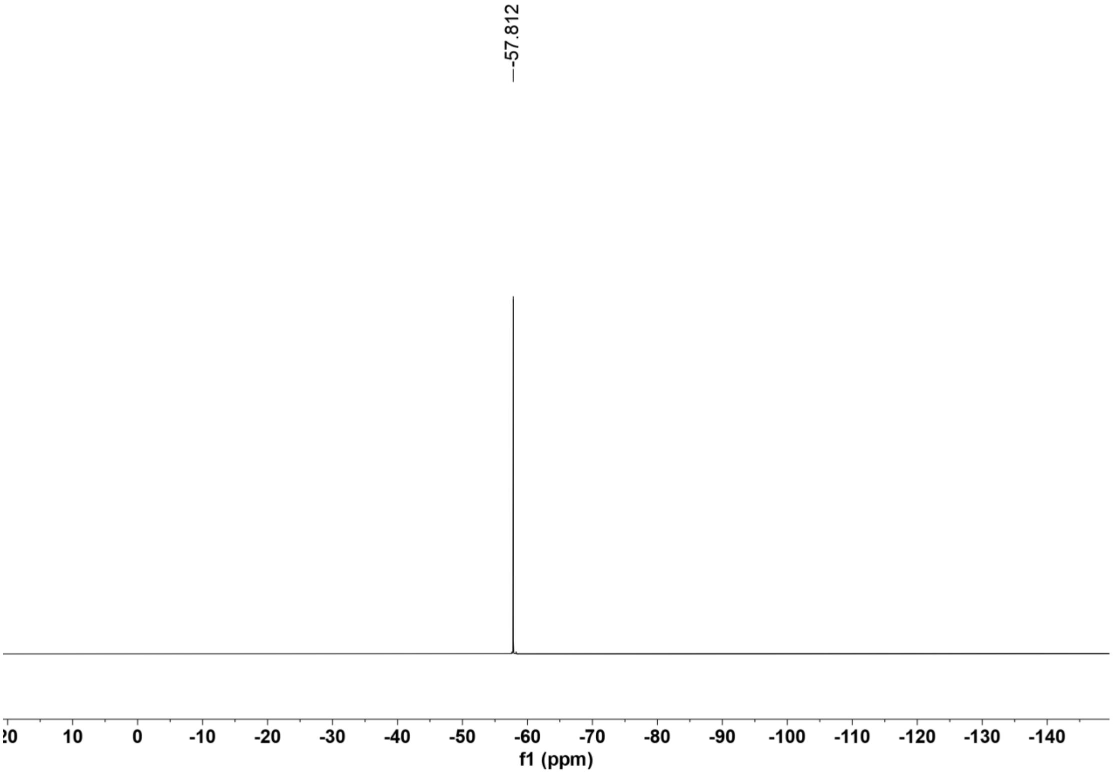

^{1}H NMR of 3be (400 MHz, $CDCl_3$)

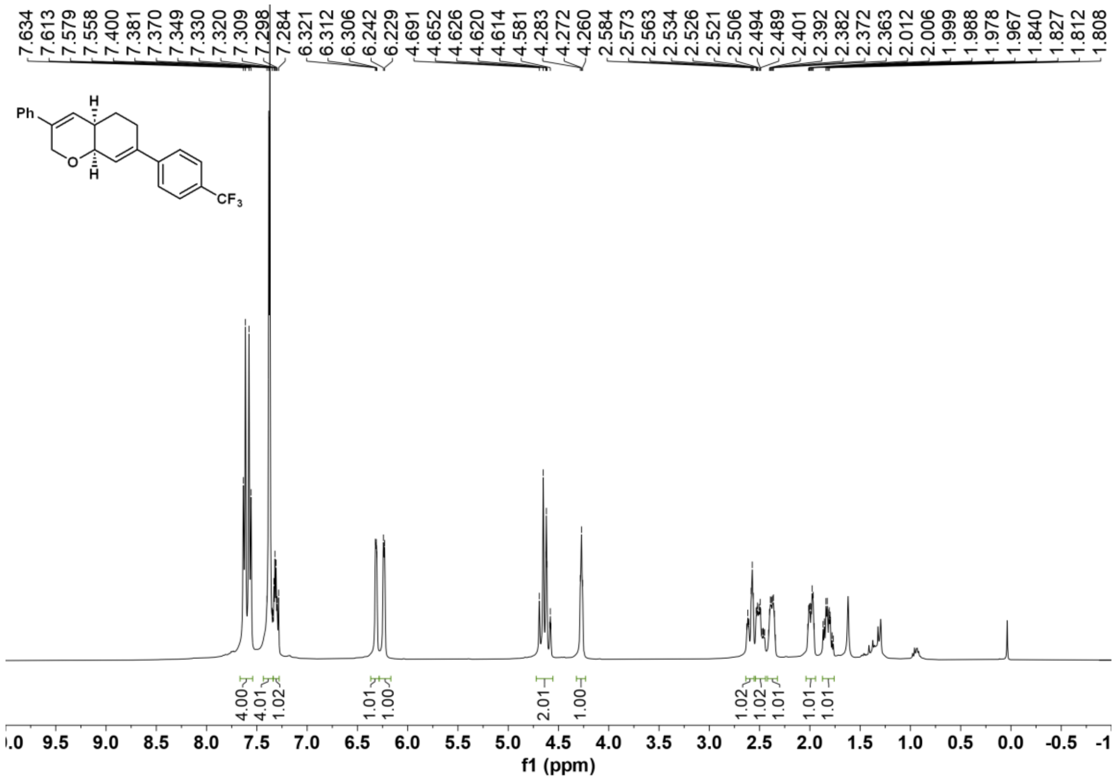

^{13}C NMR of 3be (100 MHz, $CDCl_3$)

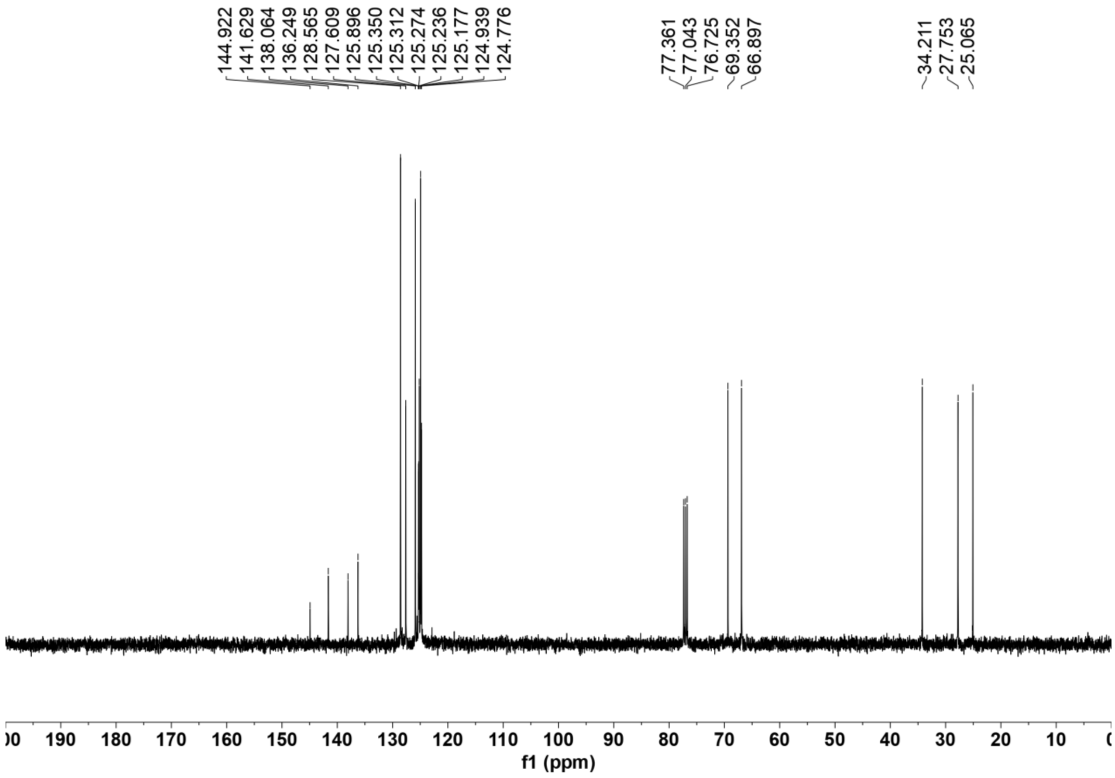

^{19}F NMR of 3be (376 MHz, $CDCl_3$)

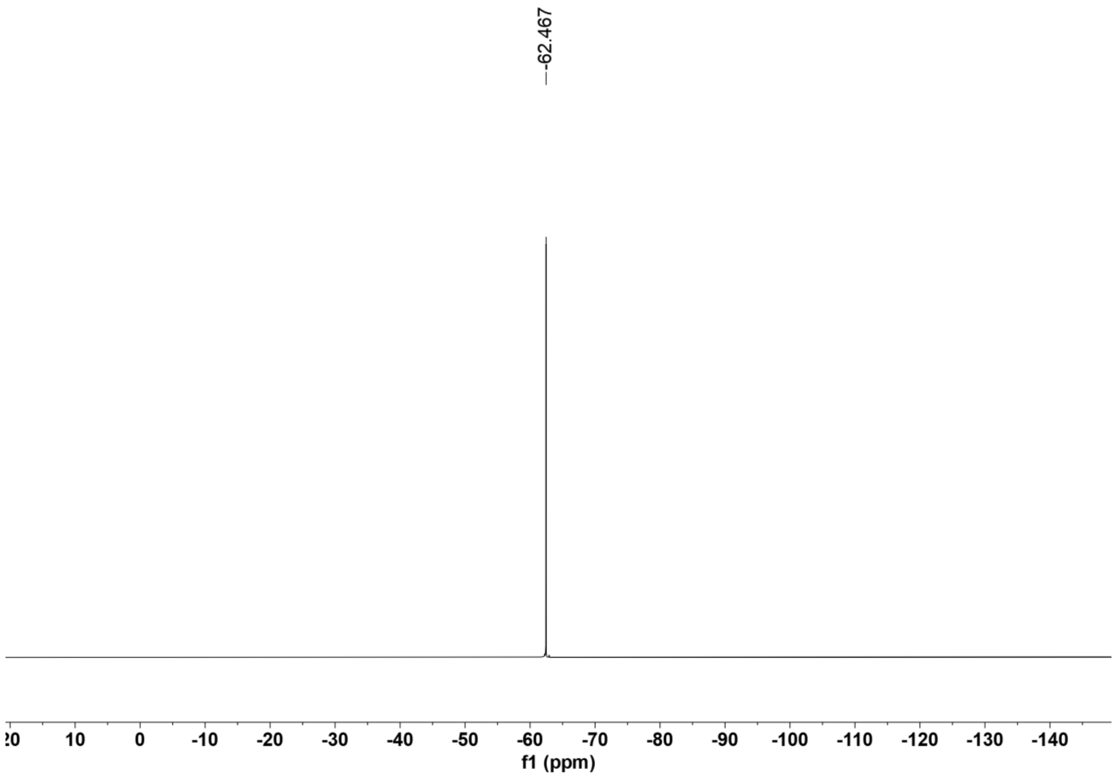

^{1}H NMR of 3bf (400 MHz, $CDCl_3$)

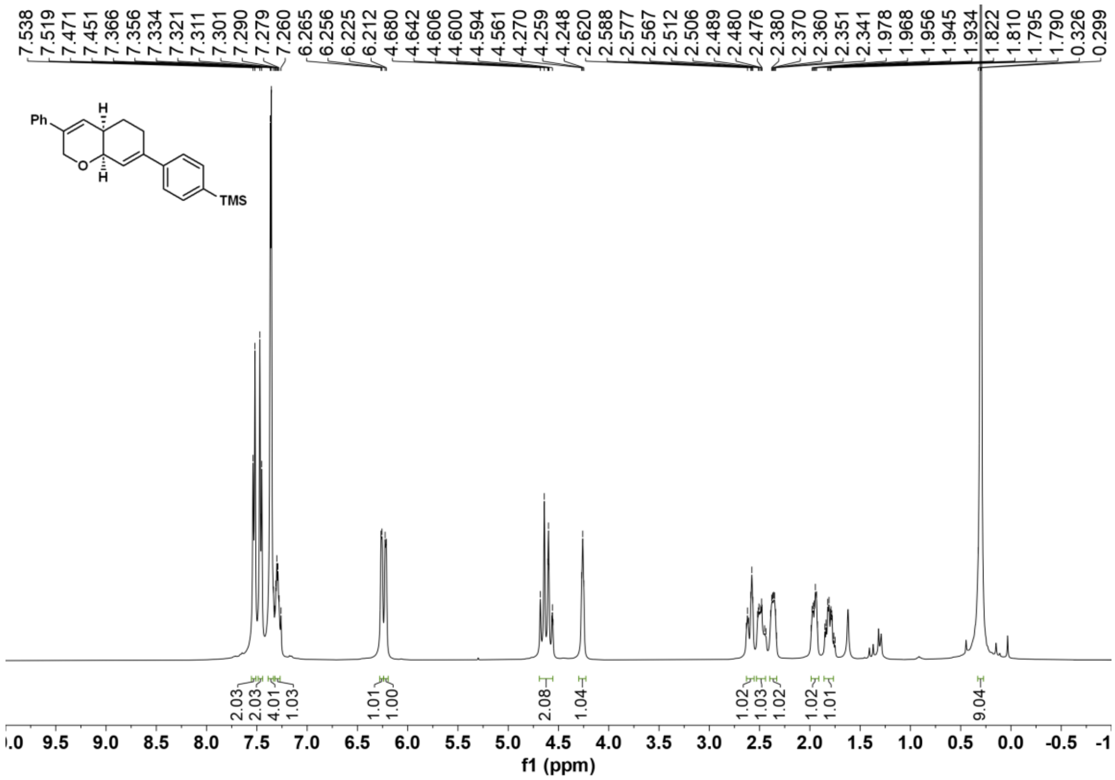

^{13}C NMR of 3bf (100 MHz, $CDCl_3$)

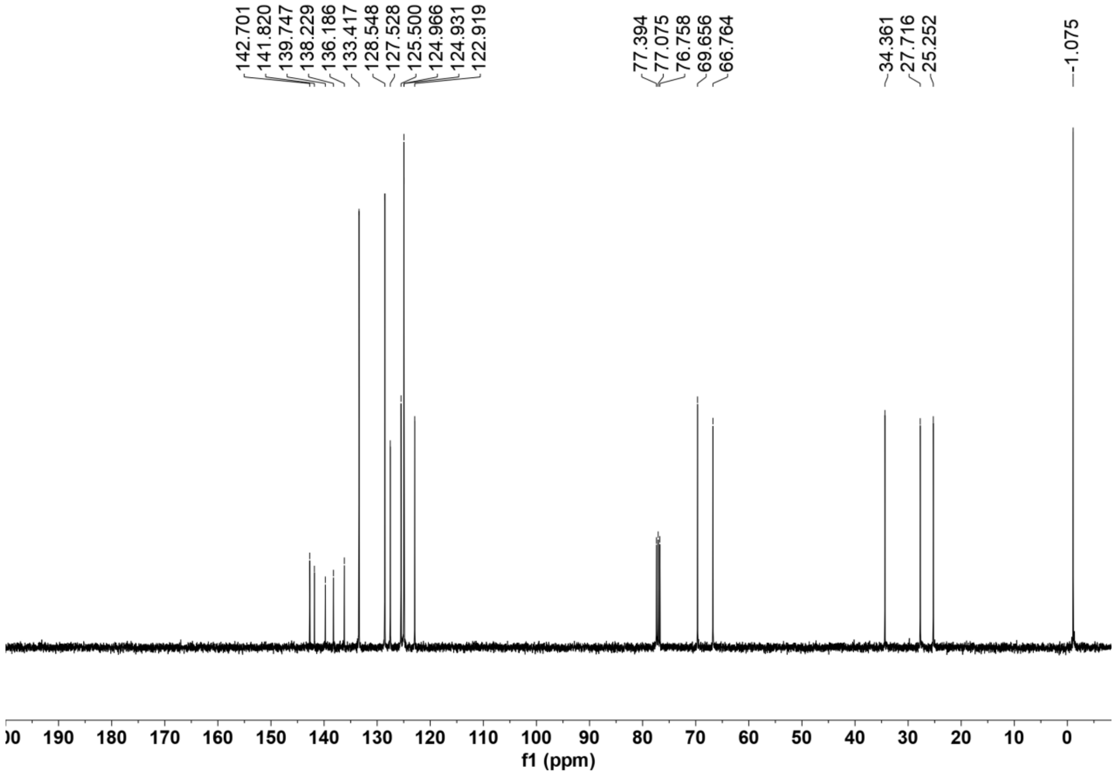

^{1}H NMR of 3bg (400 MHz, $CDCl_3$)

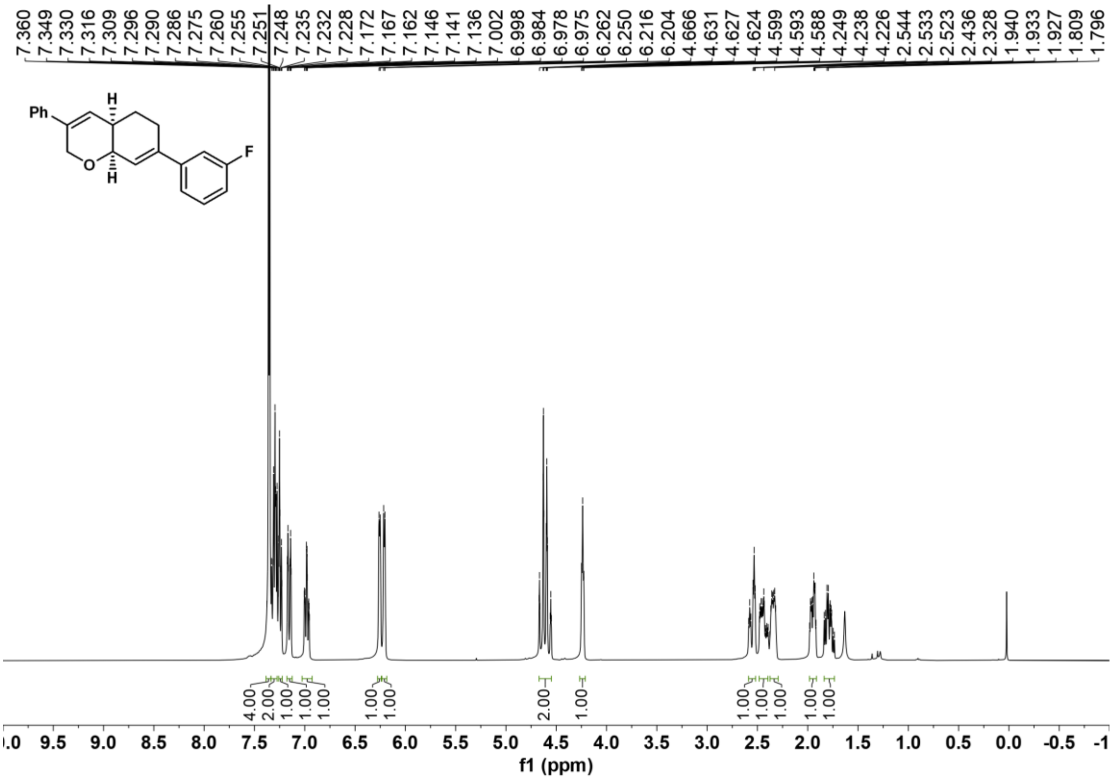

^{13}C NMR of 3bg (100 MHz, $CDCl_3$)

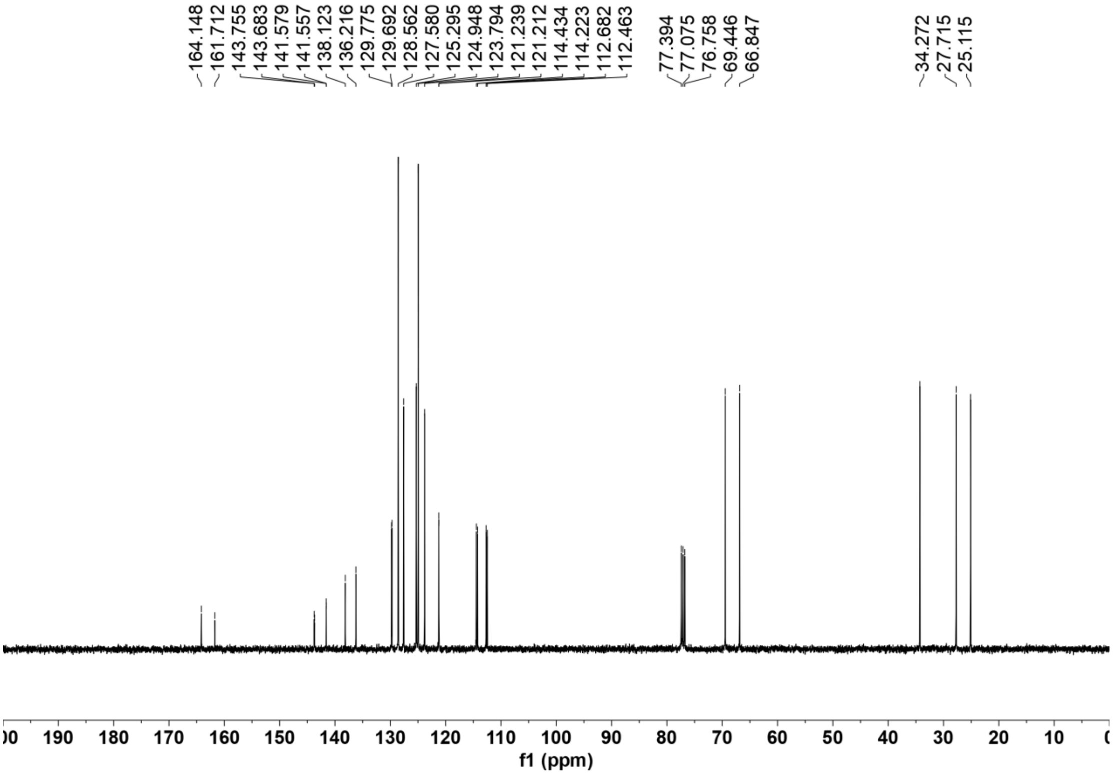

^{19}F NMR of 3bg (376 MHz, $CDCl_3$)

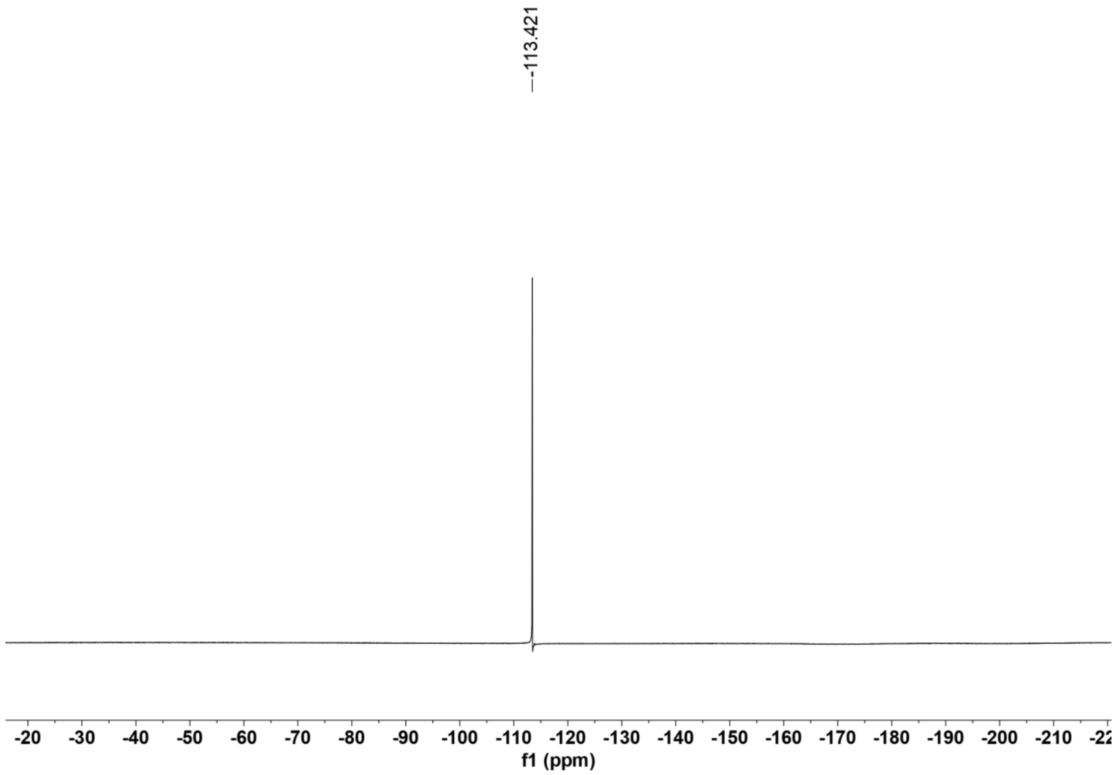

^{1}H NMR of 3bh (400 MHz, $CDCl_3$)

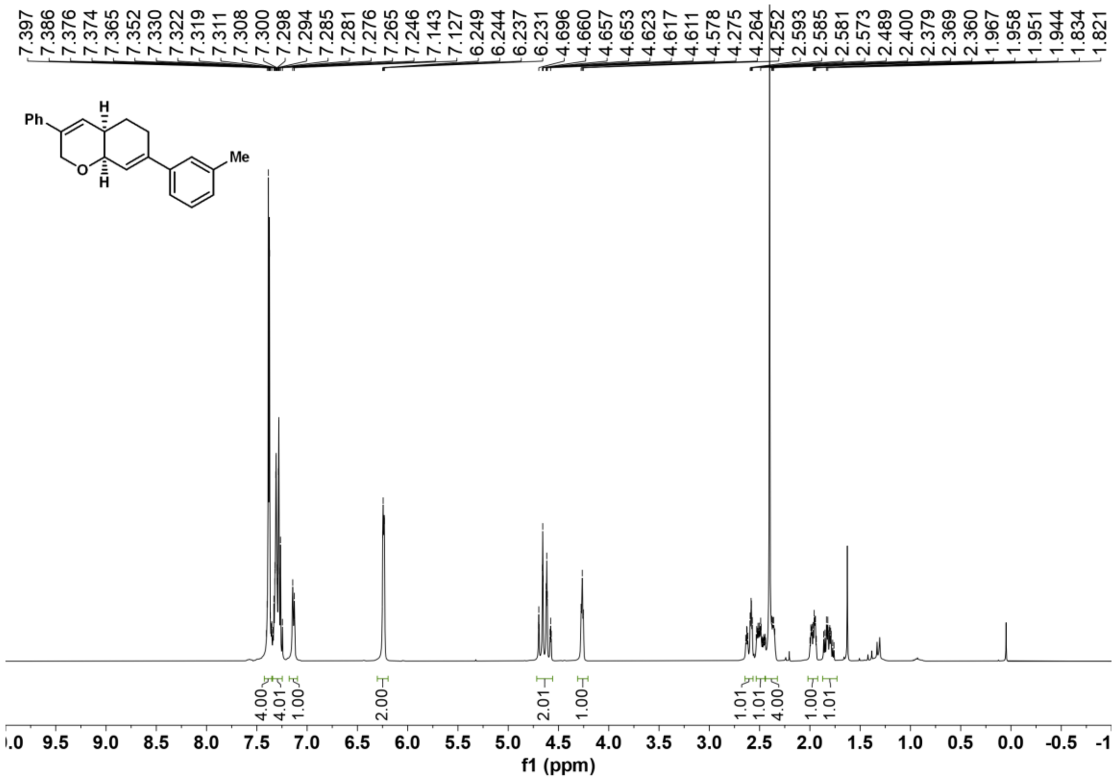

^{13}C NMR of 3bh (100 MHz, $CDCl_3$)

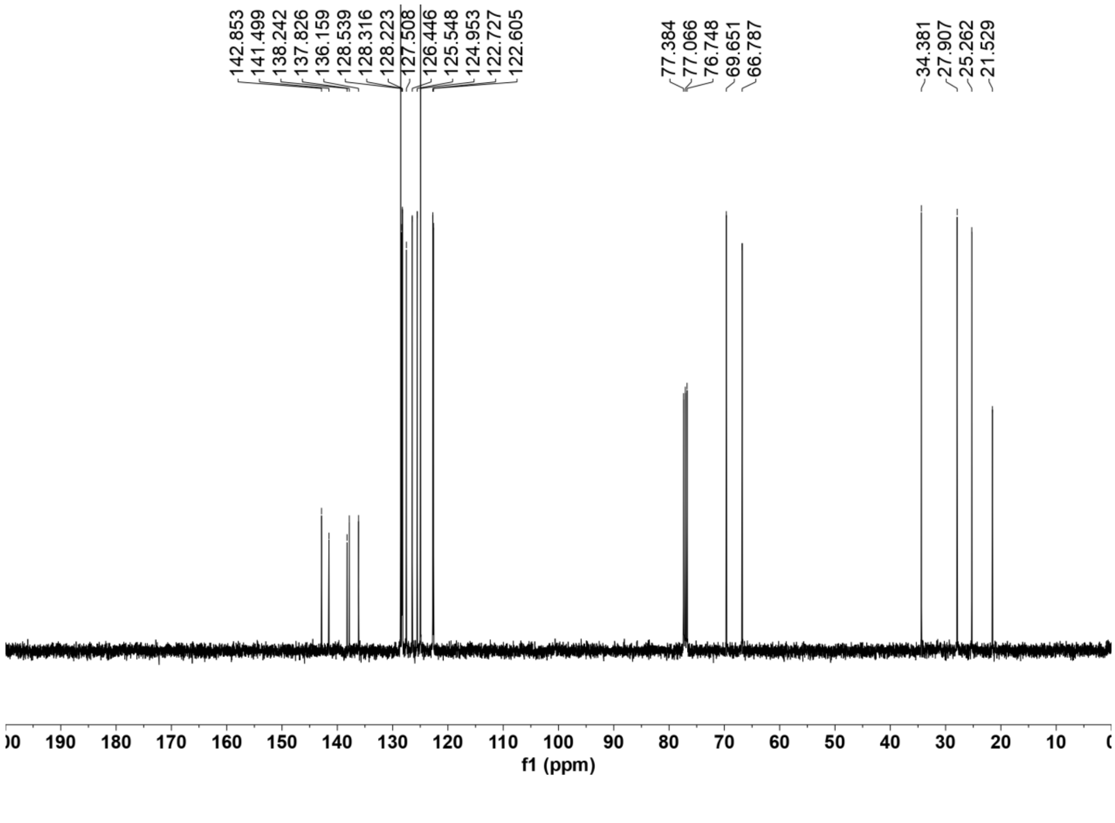

^{1}H NMR of 3bi (400 MHz, $CDCl_3$)

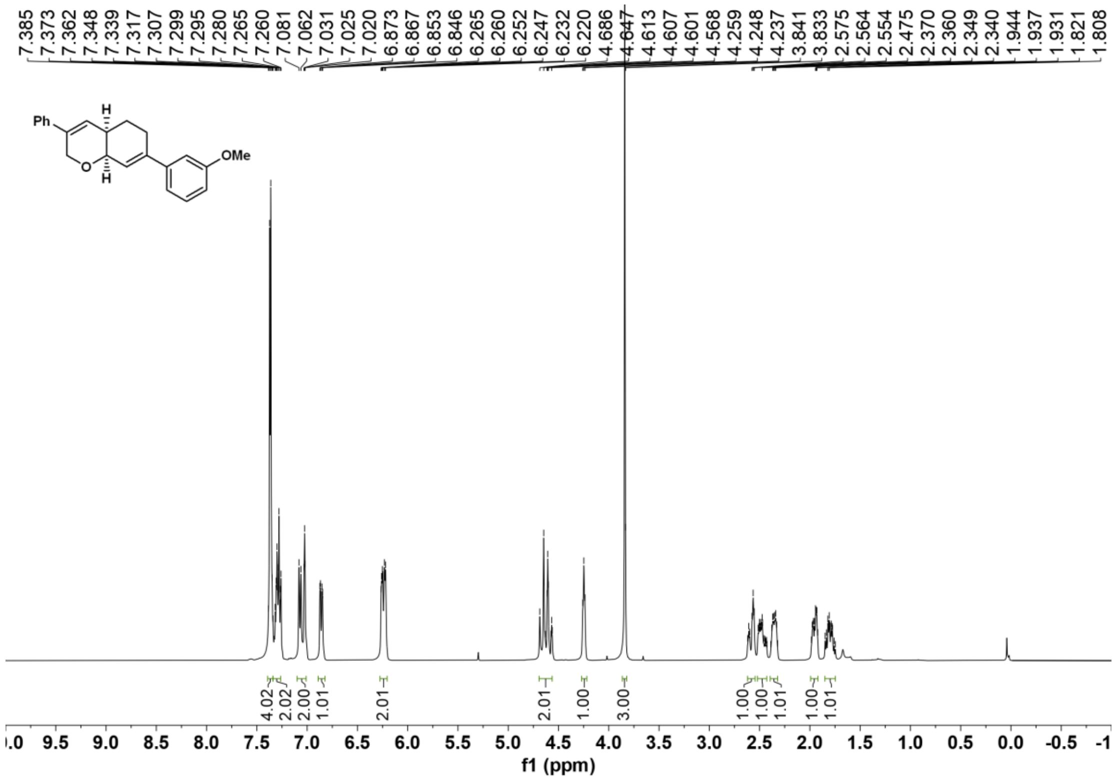

^{13}C NMR of 3bi (100 MHz, $CDCl_3$)

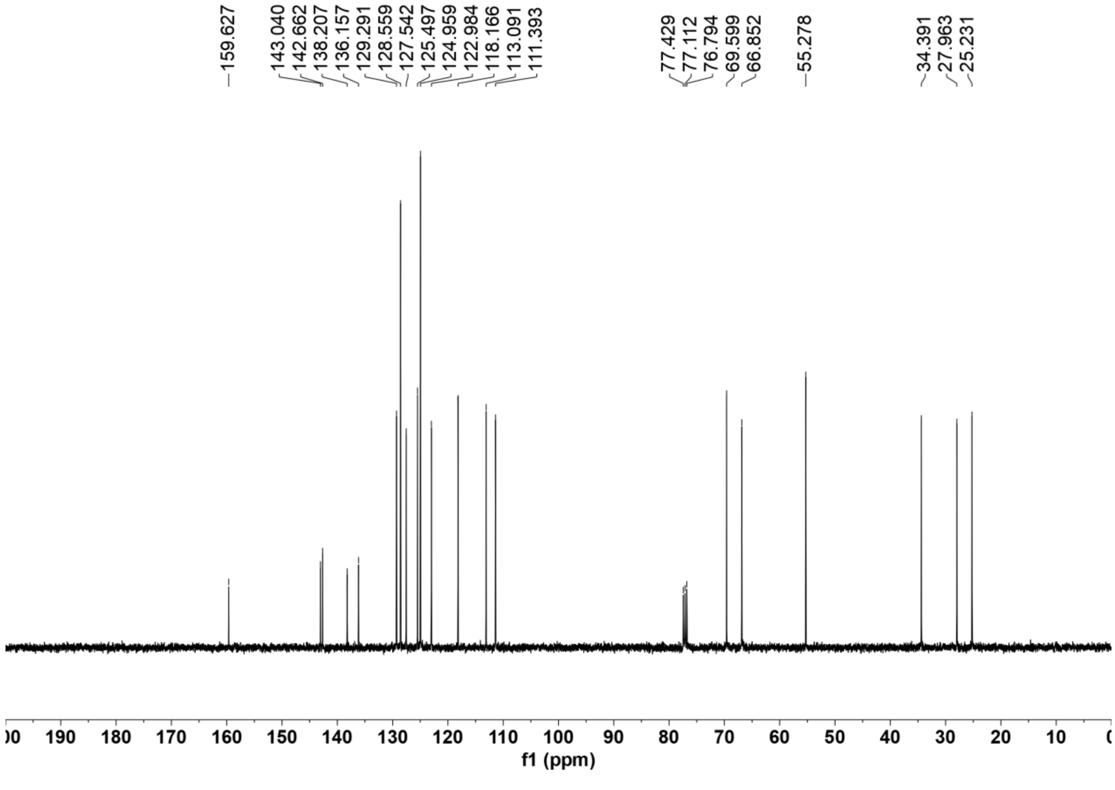

^{1}H NMR of 3bj (400 MHz, $CDCl_3$)

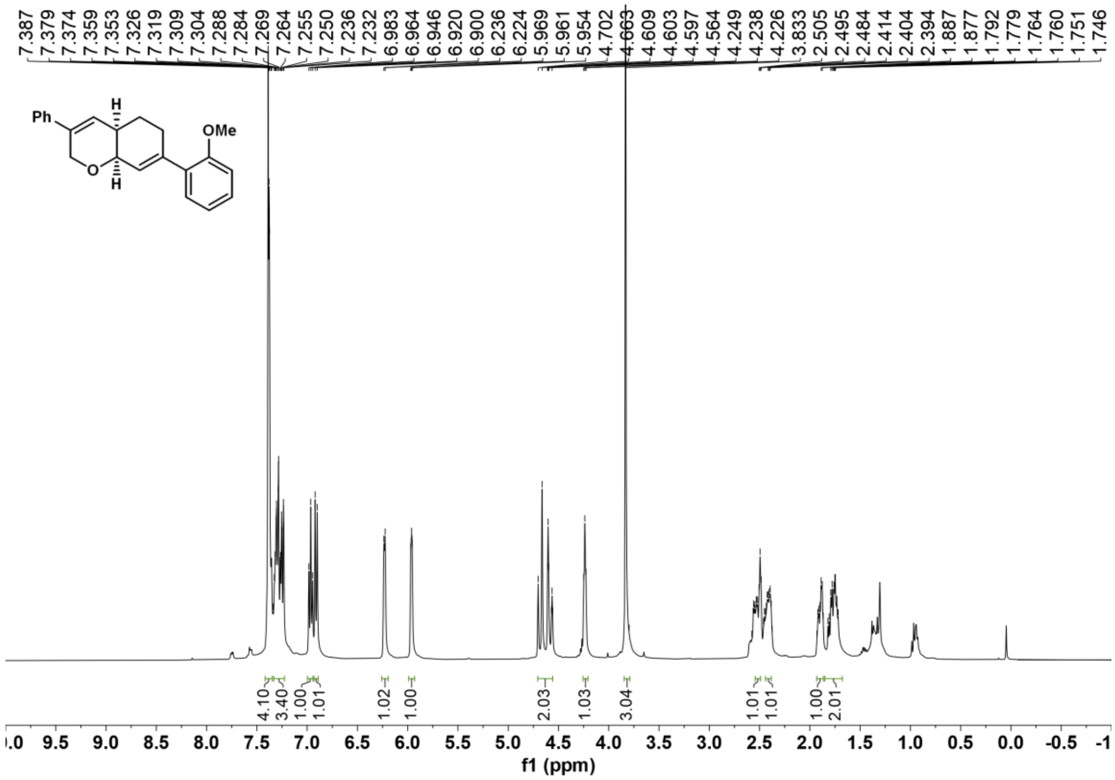

^{13}C NMR of 3bj (100 MHz, $CDCl_3$)

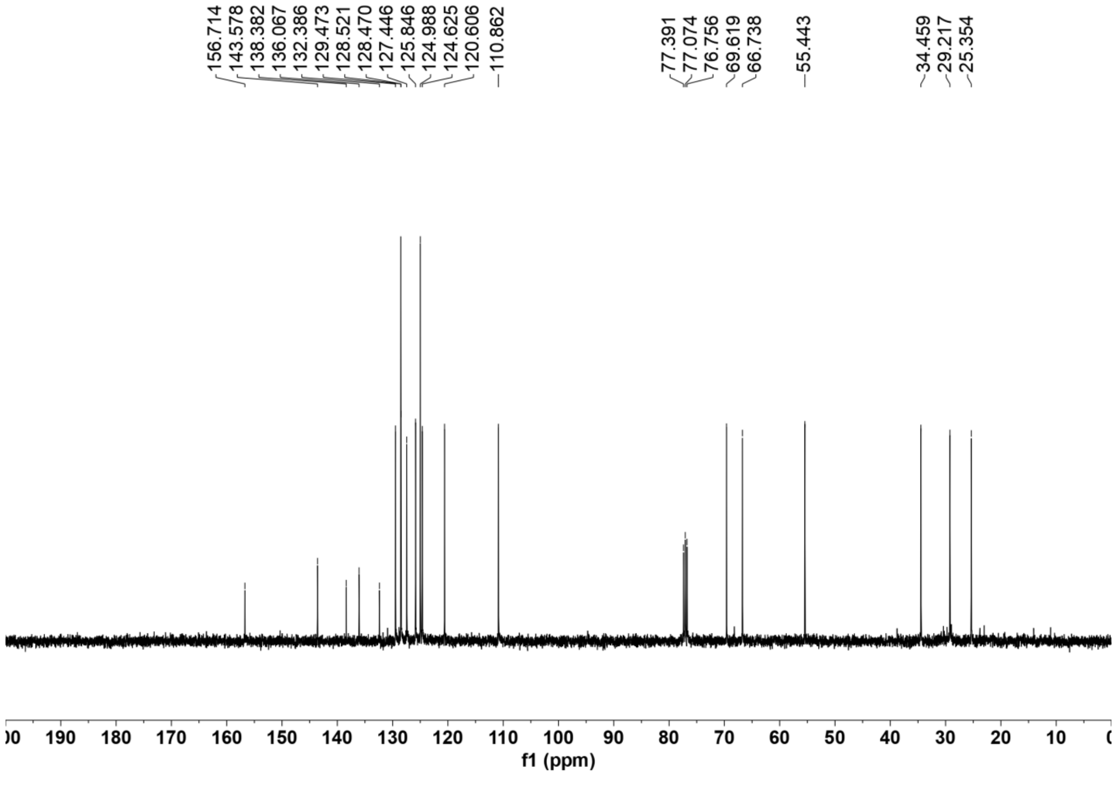

^{1}H NMR of 3bk (400 MHz, $CDCl_3$)

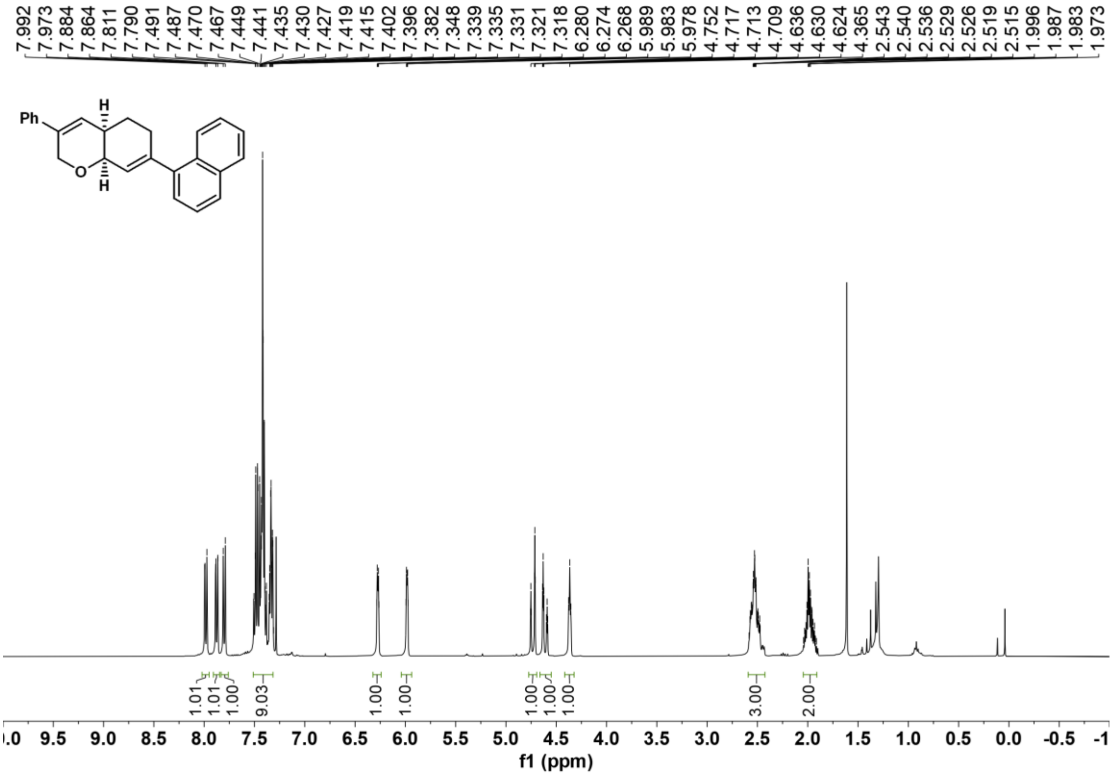

^{13}C NMR of 3bk (100 MHz, $CDCl_3$)

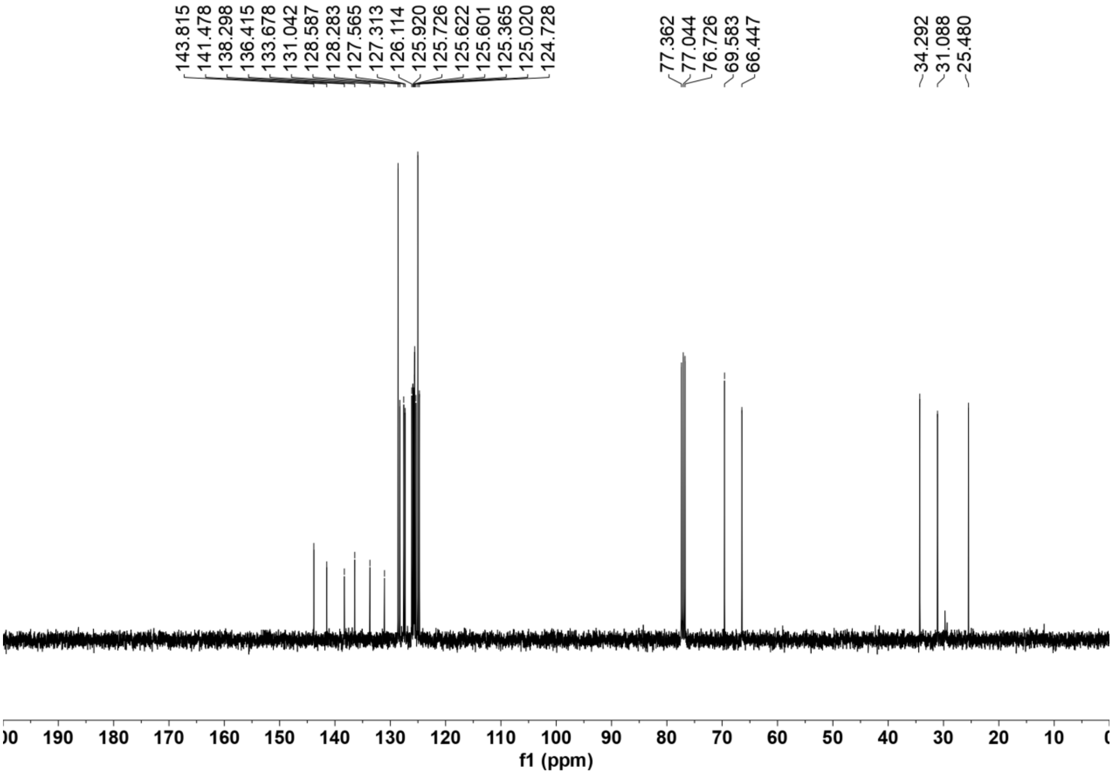

^{1}H NMR of 3bl (400 MHz, $CDCl_3$)

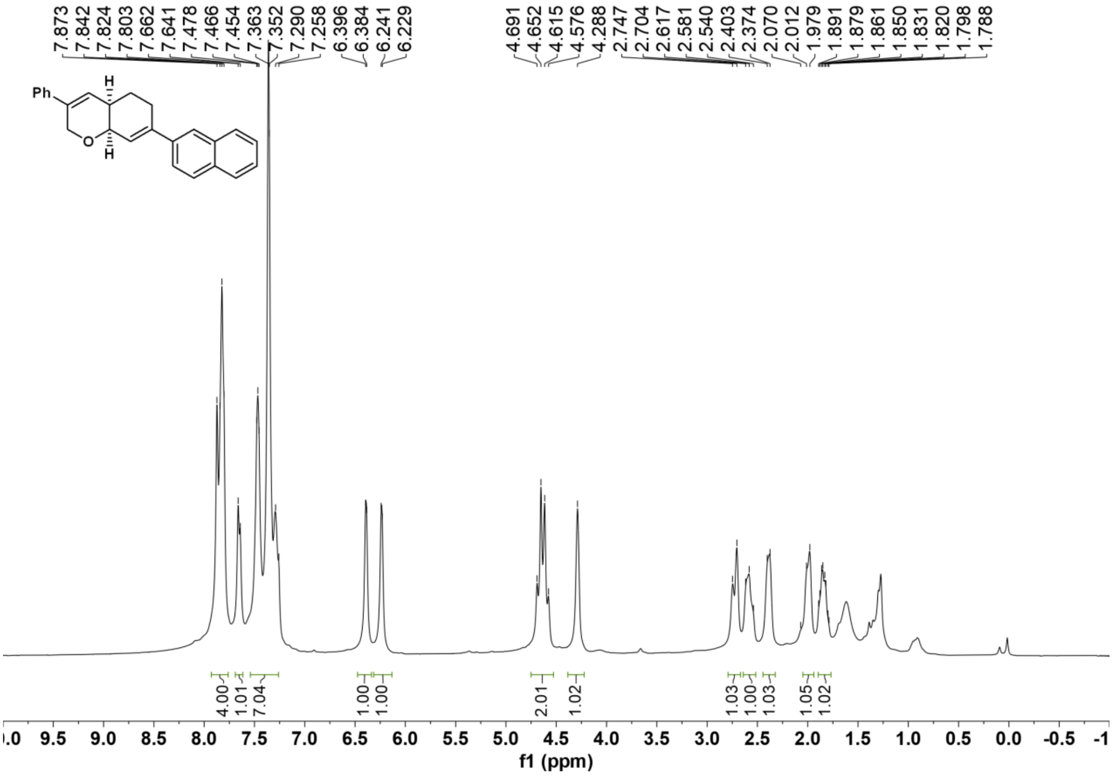

^{13}C NMR of 3bl (100 MHz, $CDCl_3$)

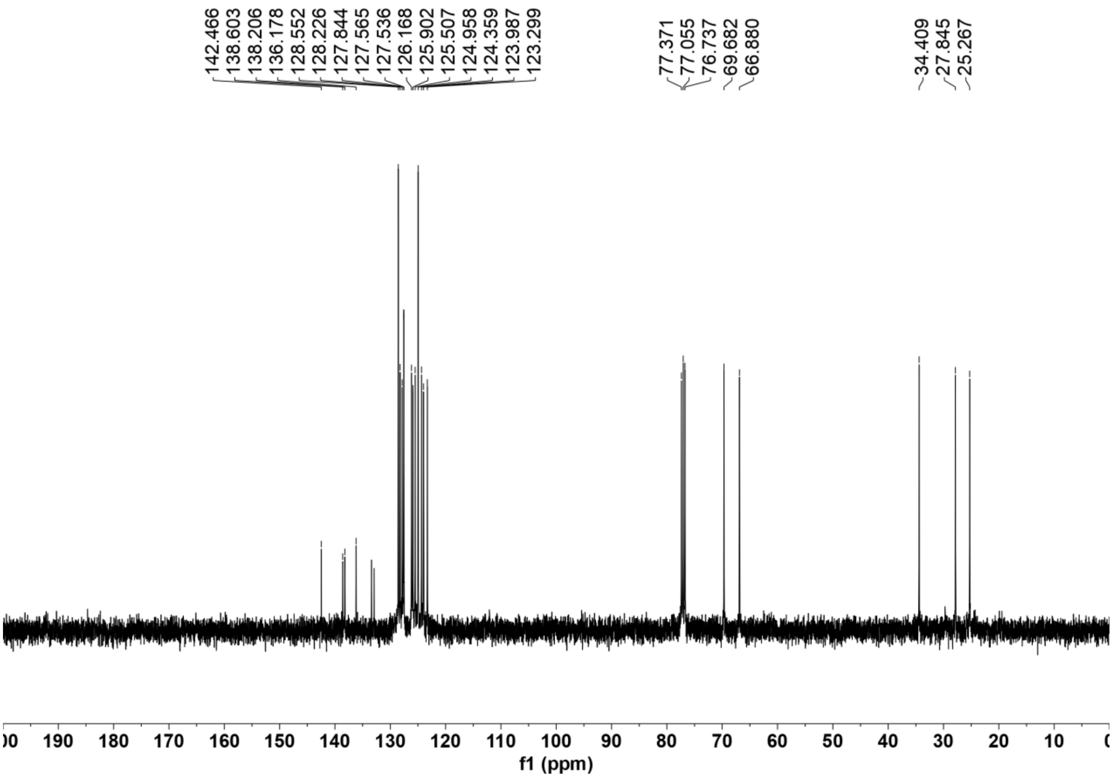

^{1}H NMR of 3bm (400 MHz, $CDCl_3$)

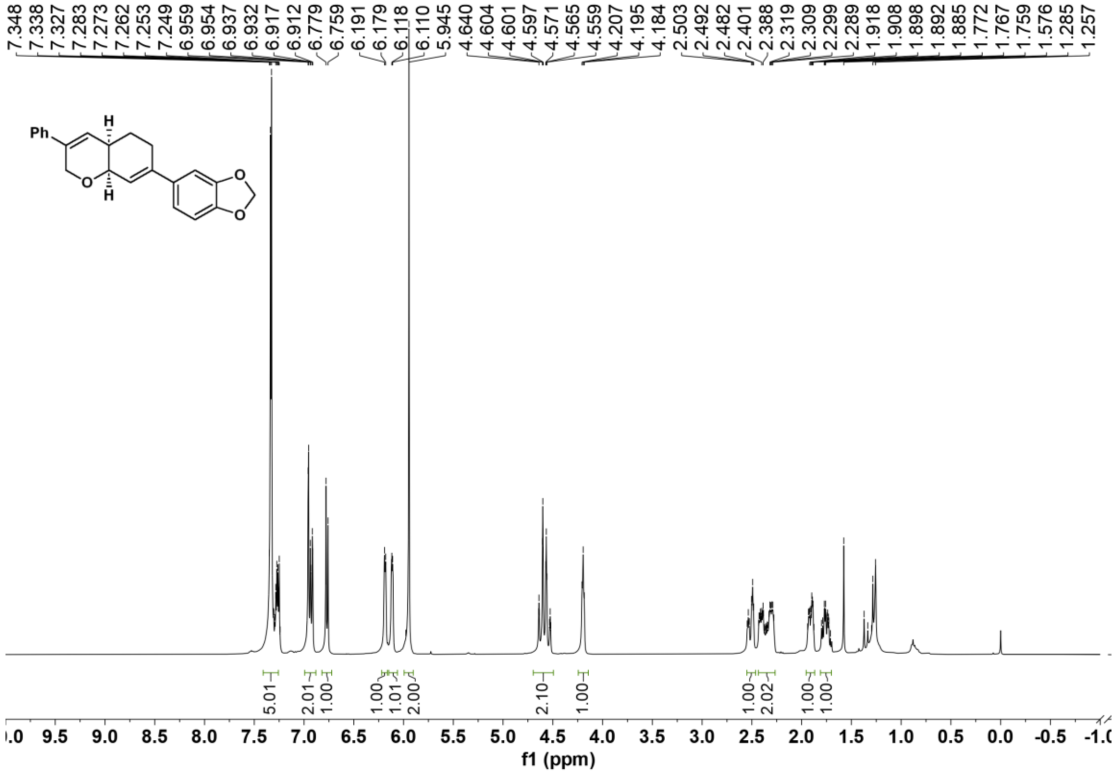

^{13}C NMR of 3bm (100 MHz, $CDCl_3$)

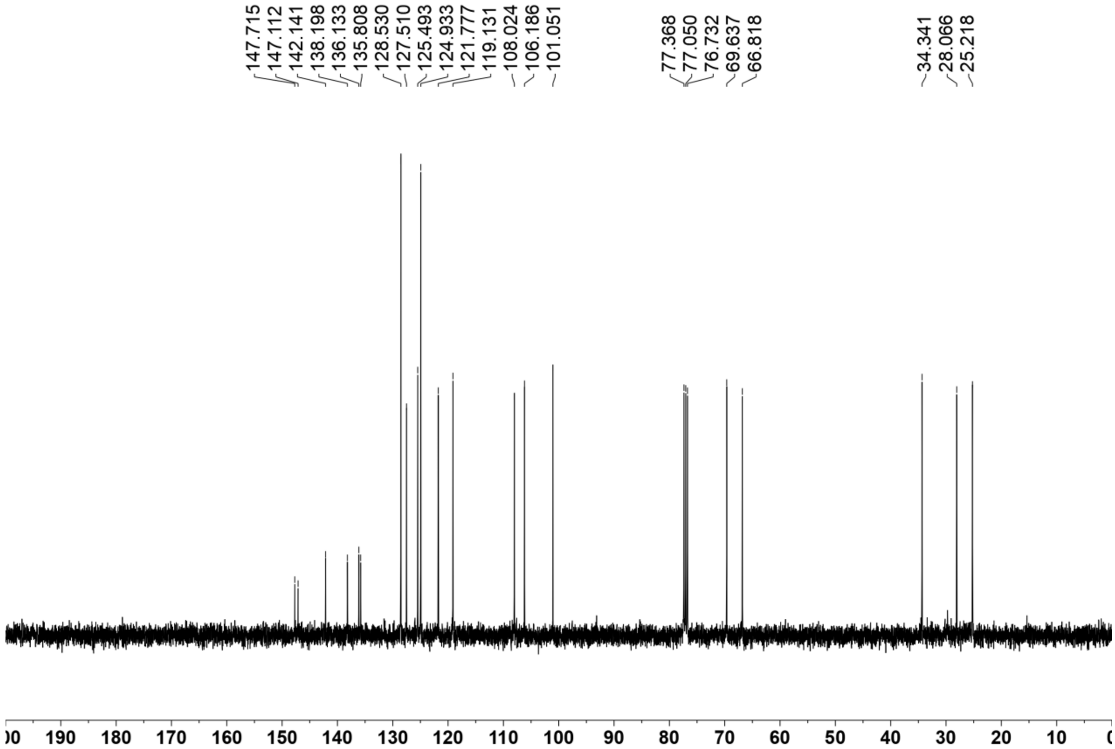

^{1}H NMR of 3bn (400 MHz, $CDCl_3$)

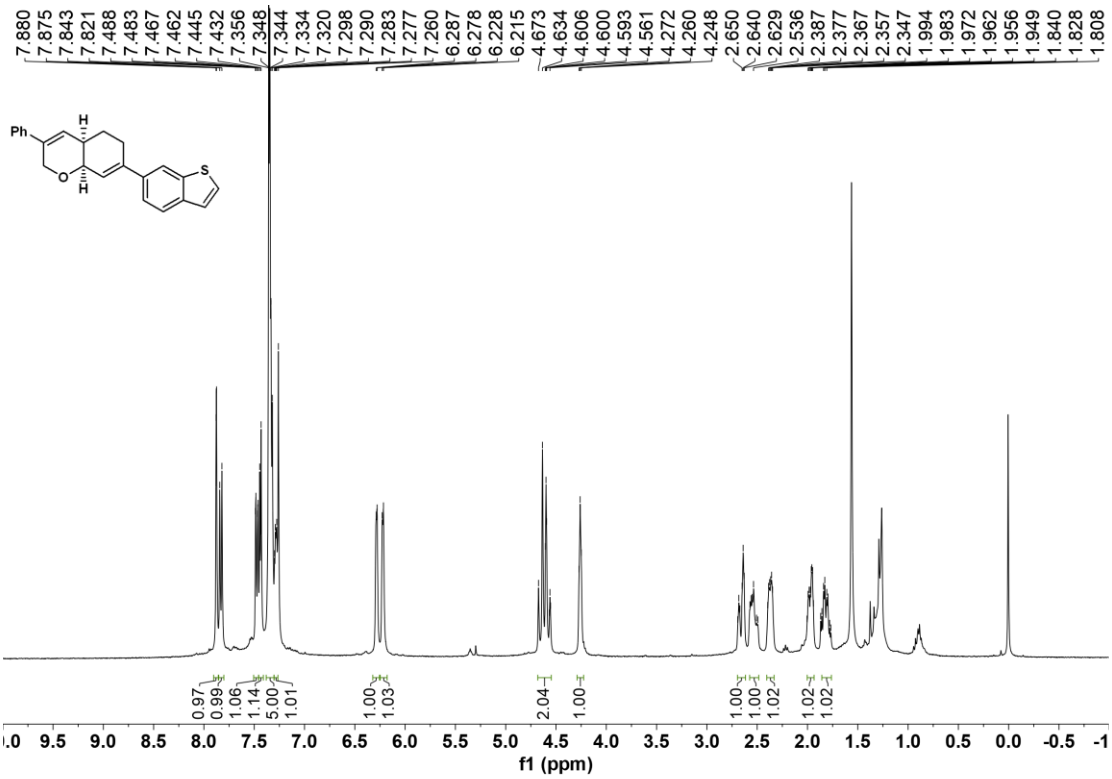

^{13}C NMR of 3bn (100 MHz, $CDCl_3$)

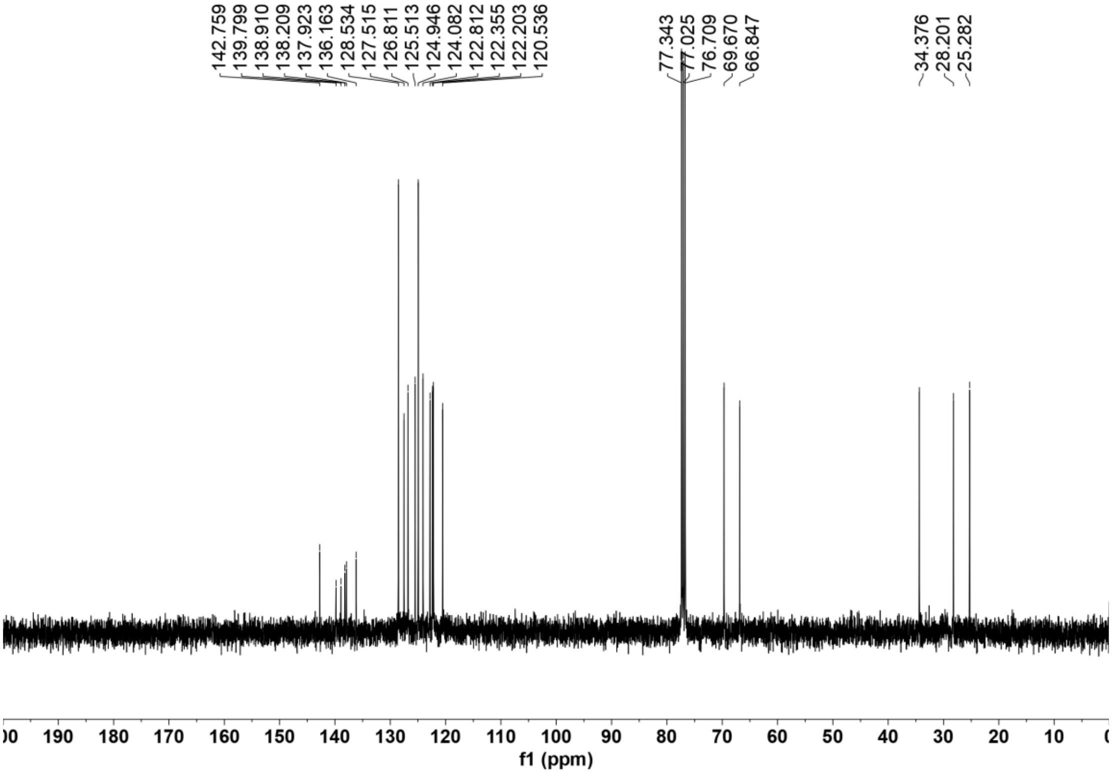

^{1}H NMR of 3bo (400 MHz, $CDCl_3$)

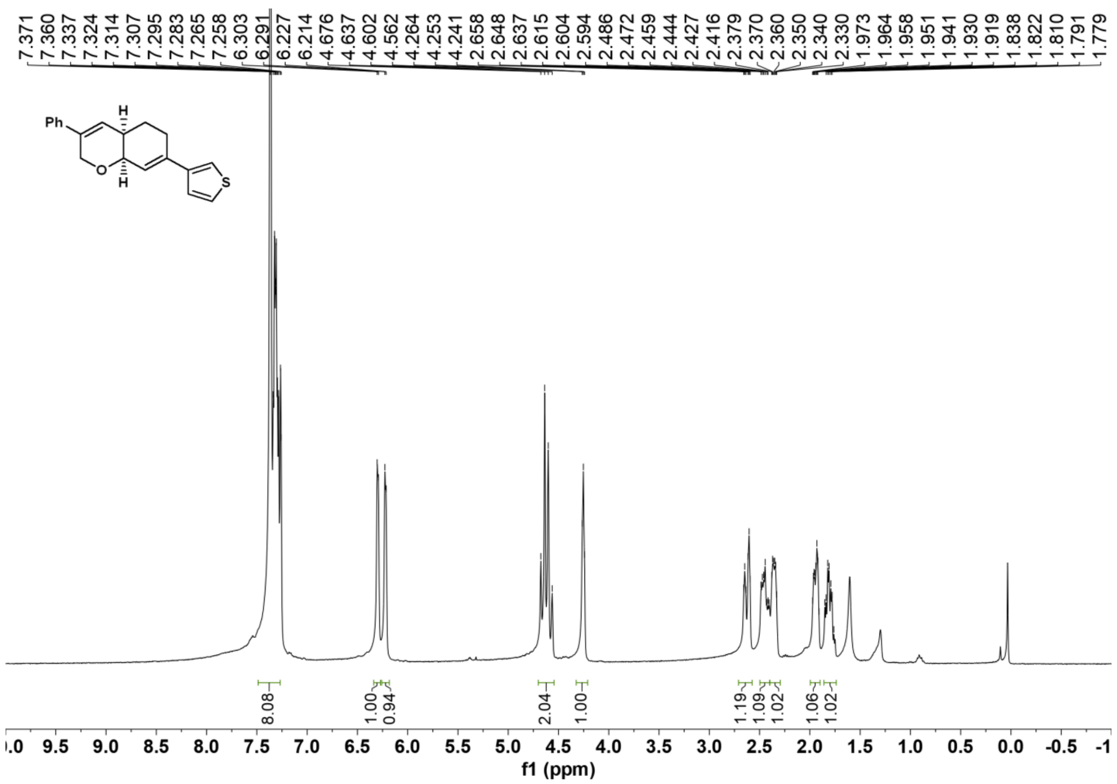

^{13}C NMR of 3bo (100 MHz, $CDCl_3$)

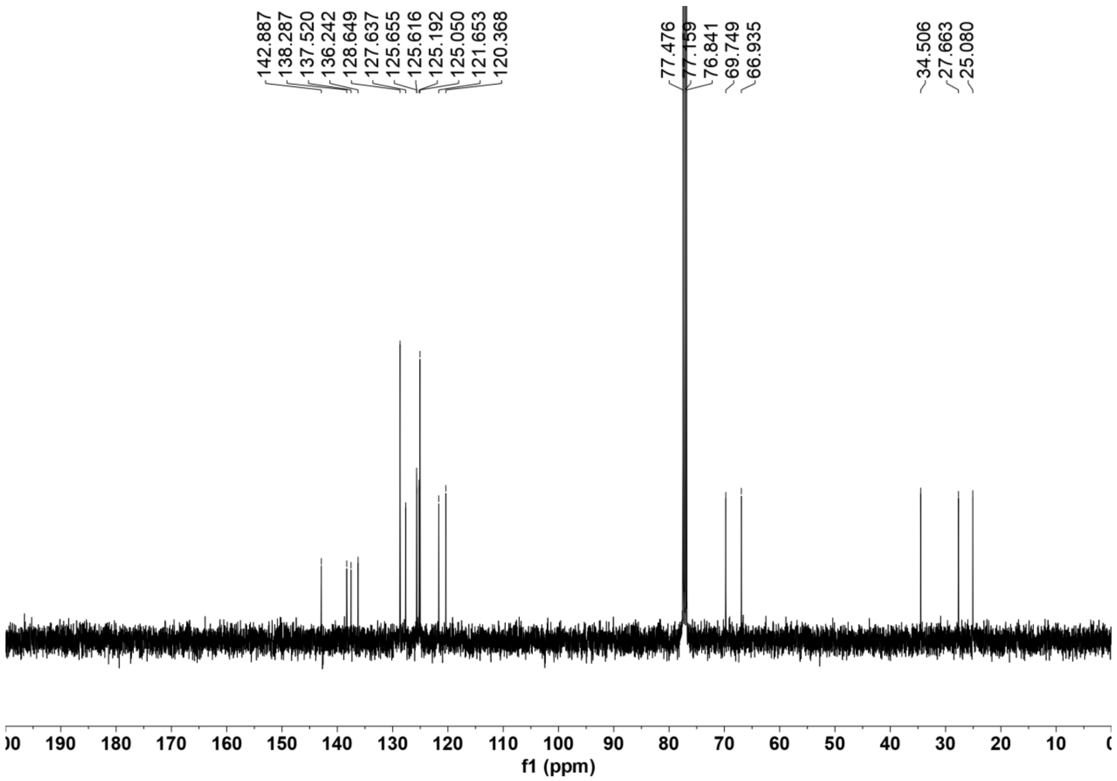

^{1}H NMR of 3bp (400 MHz, $CDCl_3$)

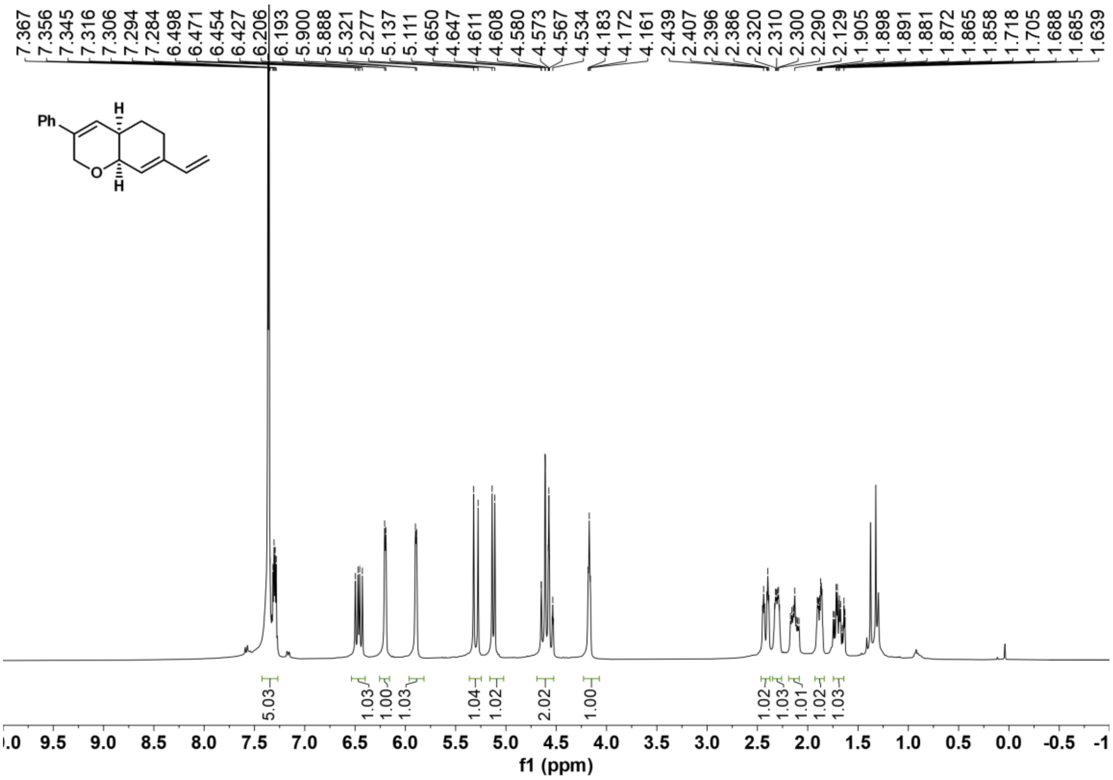

^{13}C NMR of 3bp (100 MHz, $CDCl_3$)

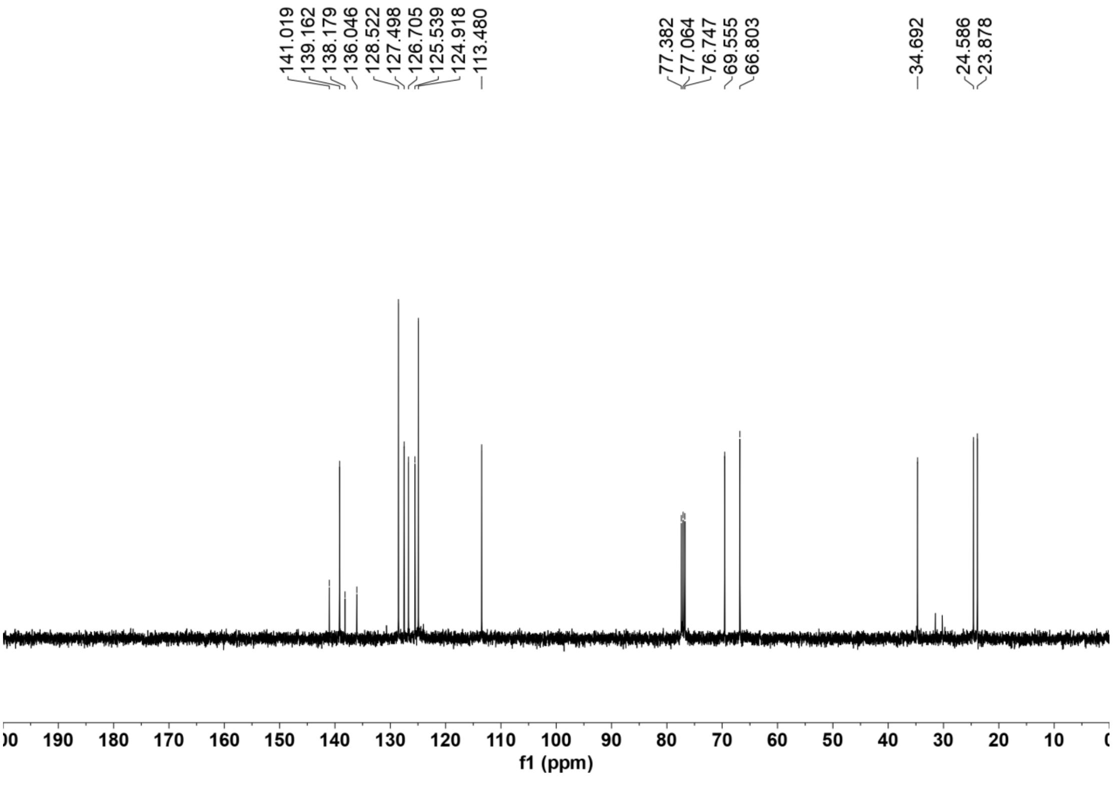

^{1}H NMR of 3bq (400 MHz, $CDCl_3$)

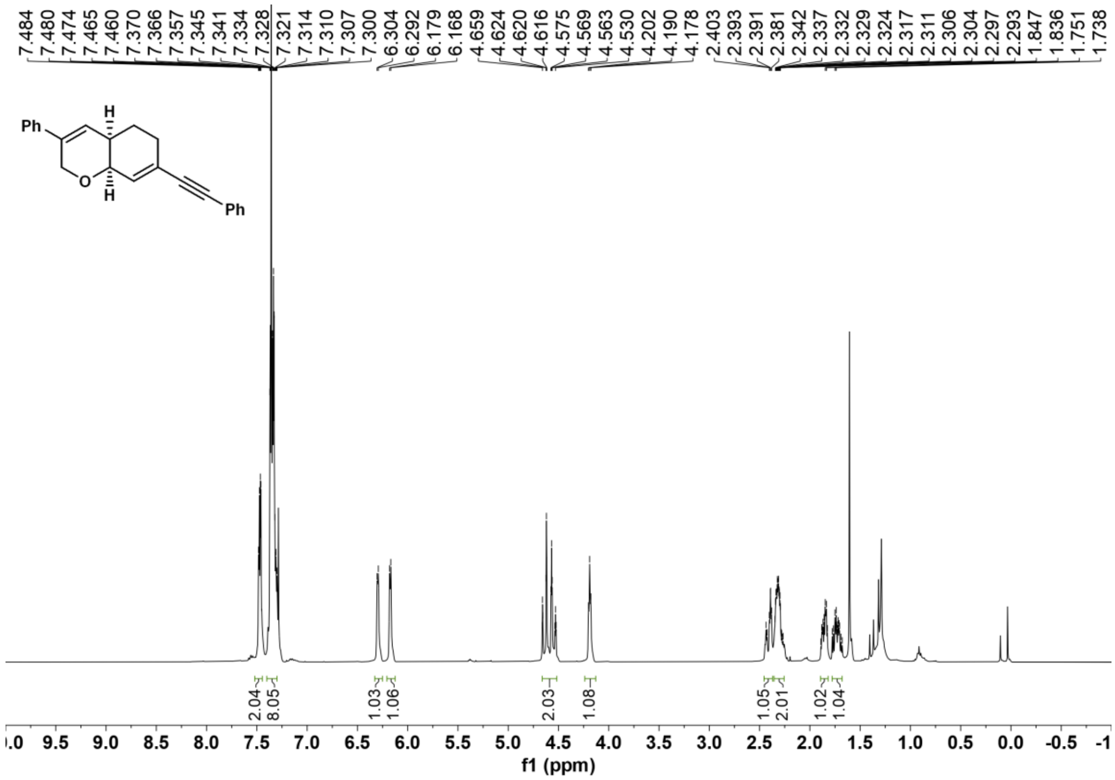

^{13}C NMR of 3bq (100 MHz, $CDCl_3$)

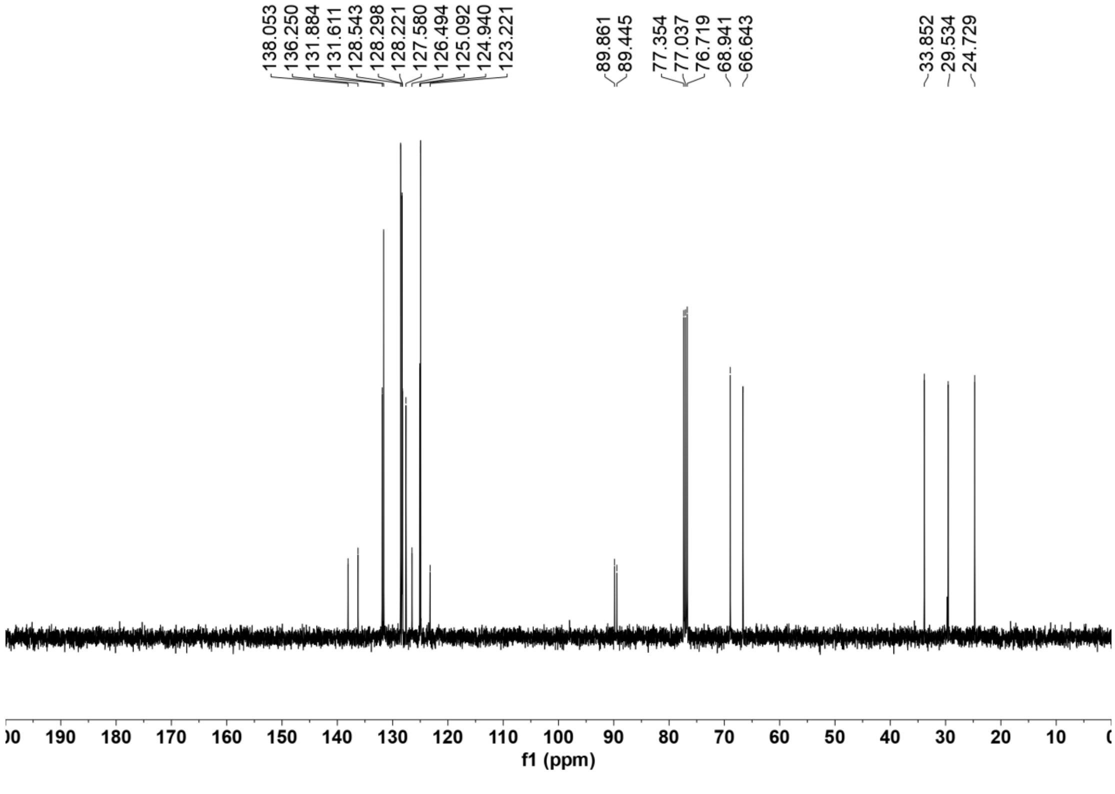

^{1}H NMR of 3br (400 MHz, $CDCl_3$)

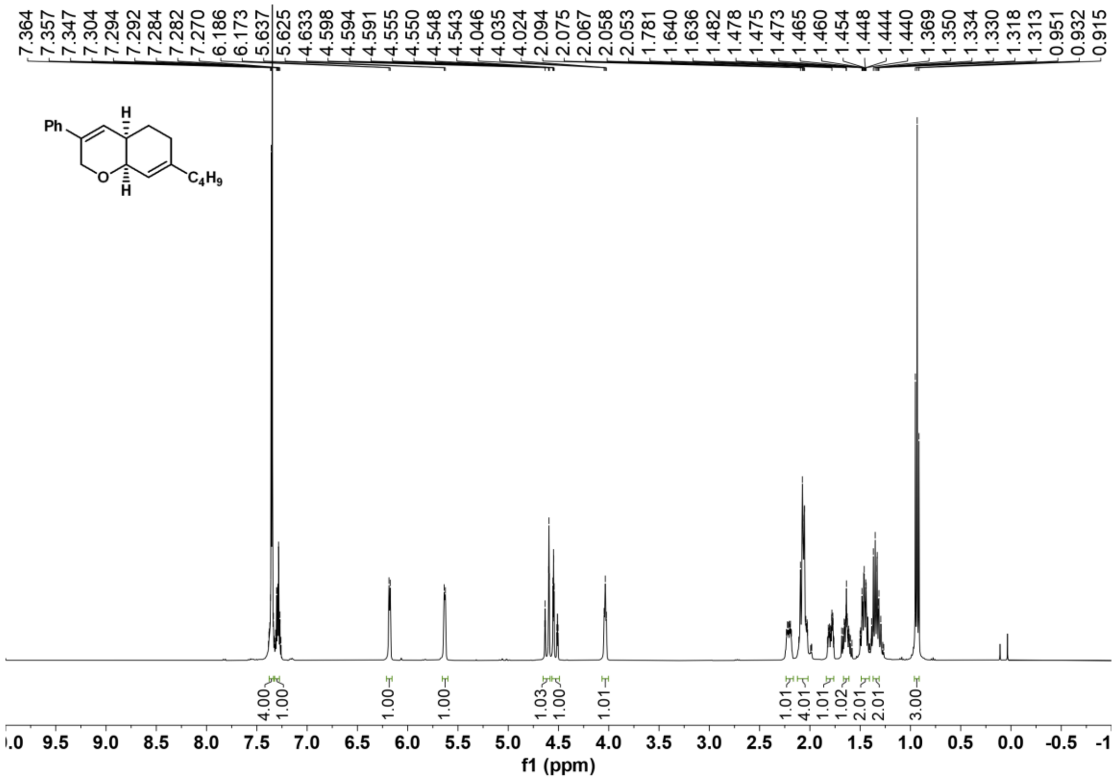

^{13}C NMR of 3br (100 MHz, $CDCl_3$)

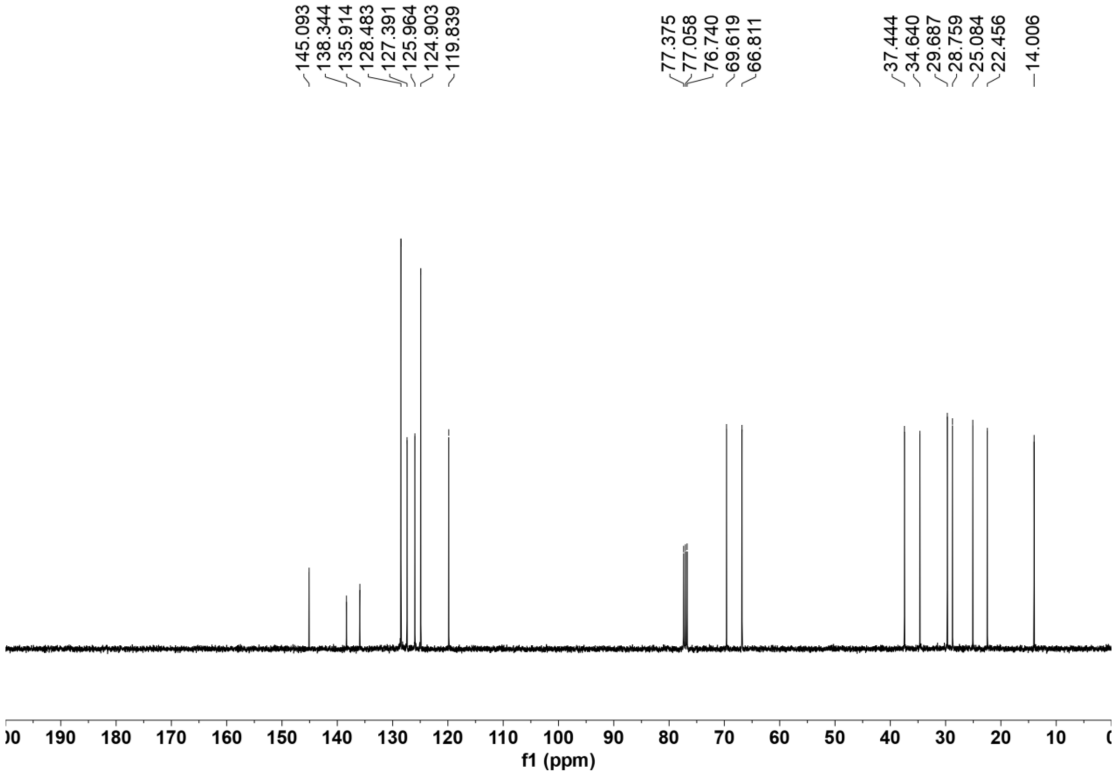

^{1}H NMR of 3bs (400 MHz, $CDCl_3$)

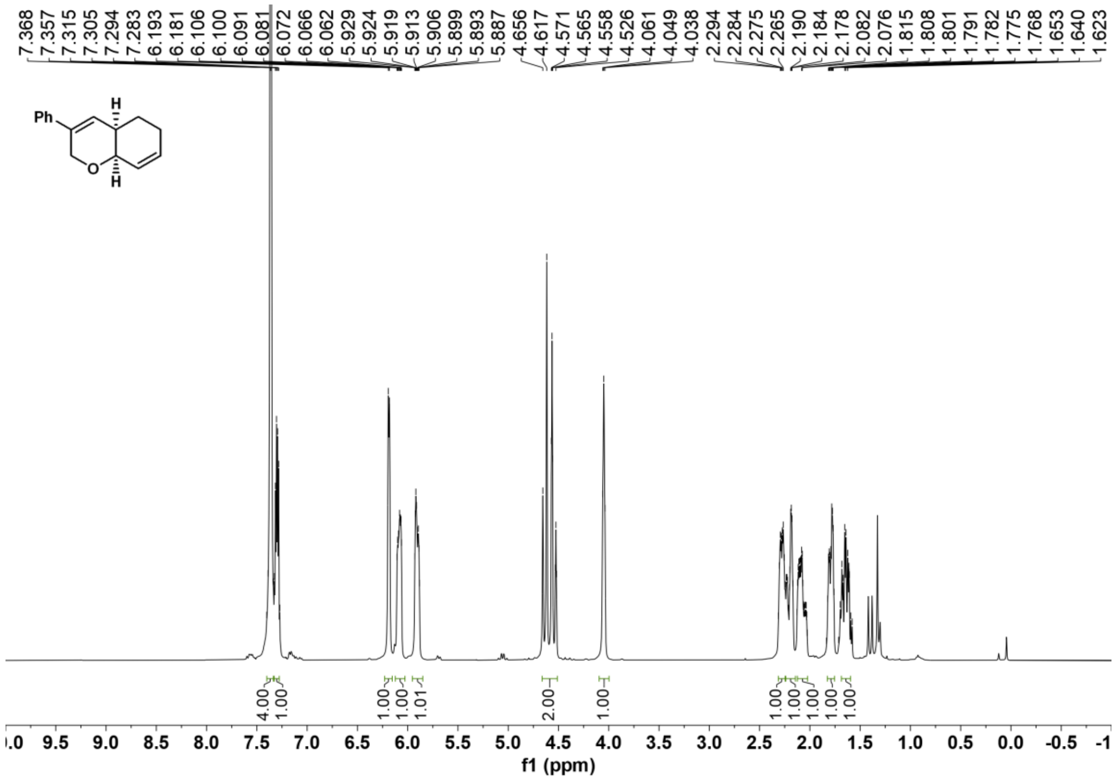

^{13}C NMR of 3bs (100 MHz, $CDCl_3$)

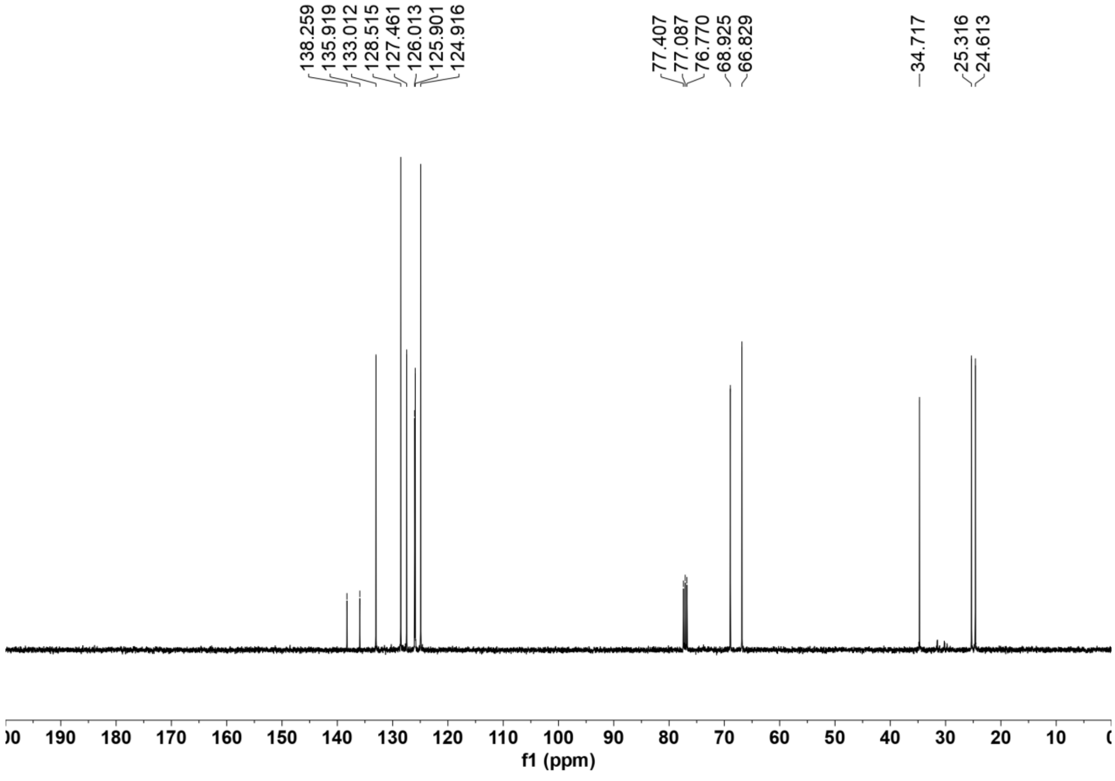

^{1}H NMR of 3bt (400 MHz, $CDCl_3$)

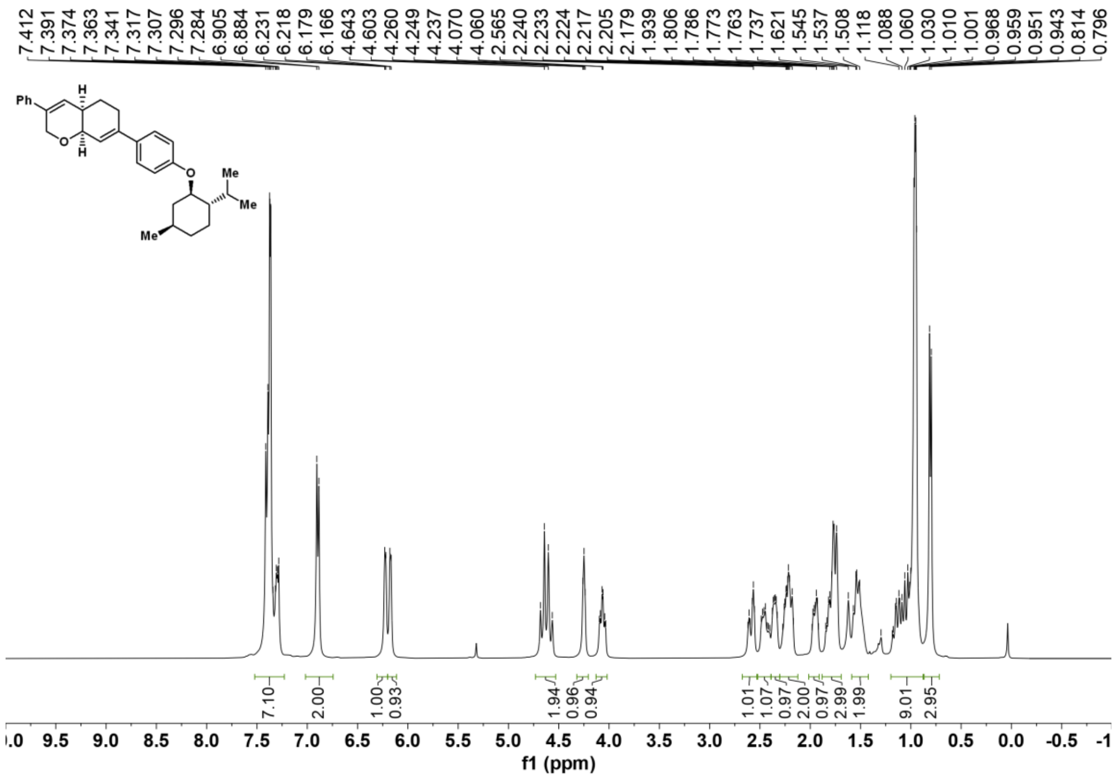

^{13}C NMR of 3bt (100 MHz, $CDCl_3$)

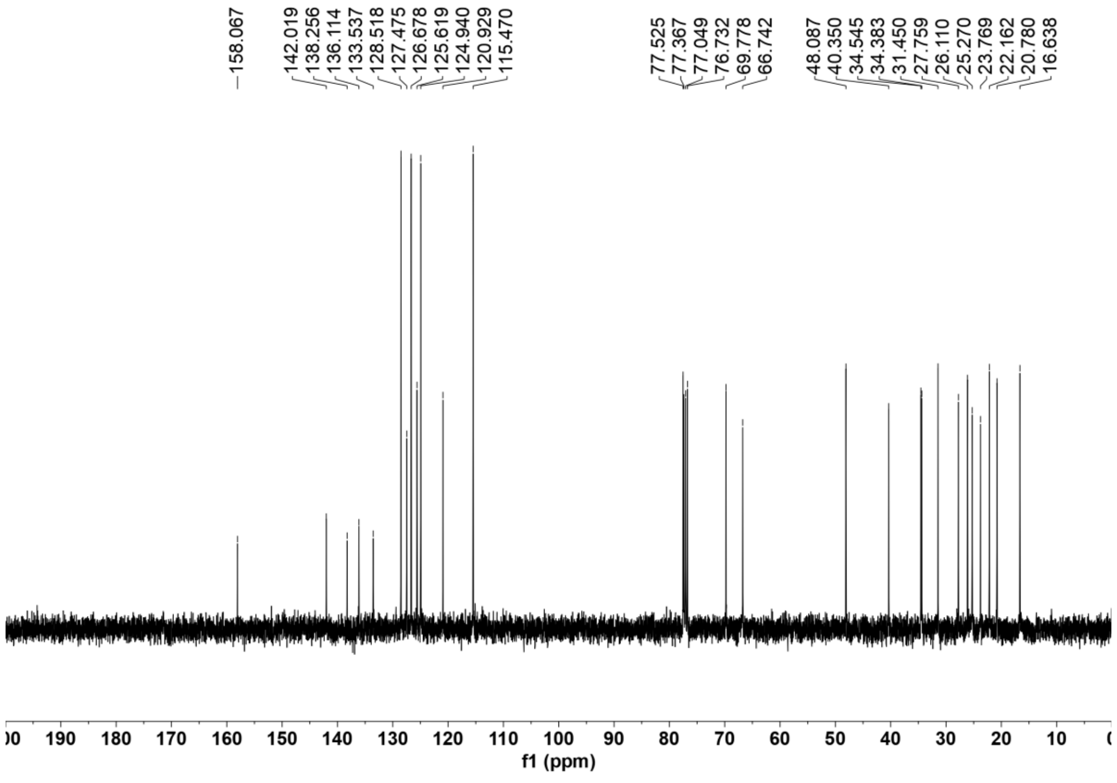

^{1}H NMR of 3bu (400 MHz, $CDCl_3$)

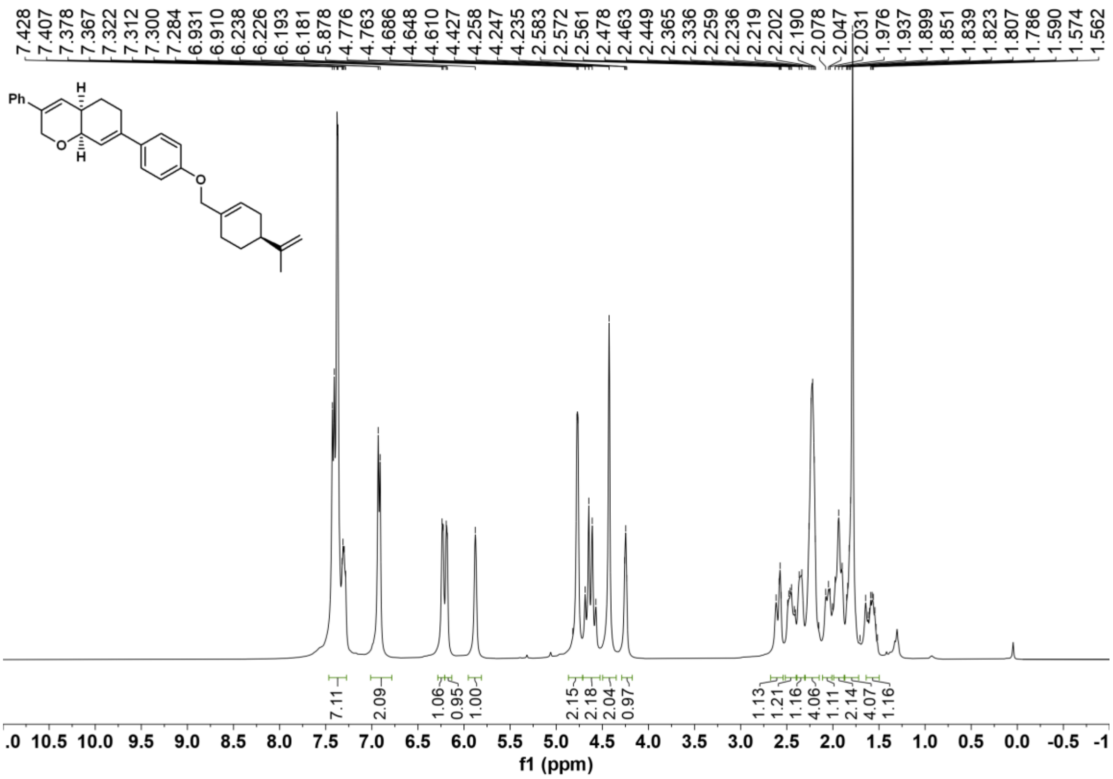

^{13}C NMR of 3bu (100 MHz, $CDCl_3$)

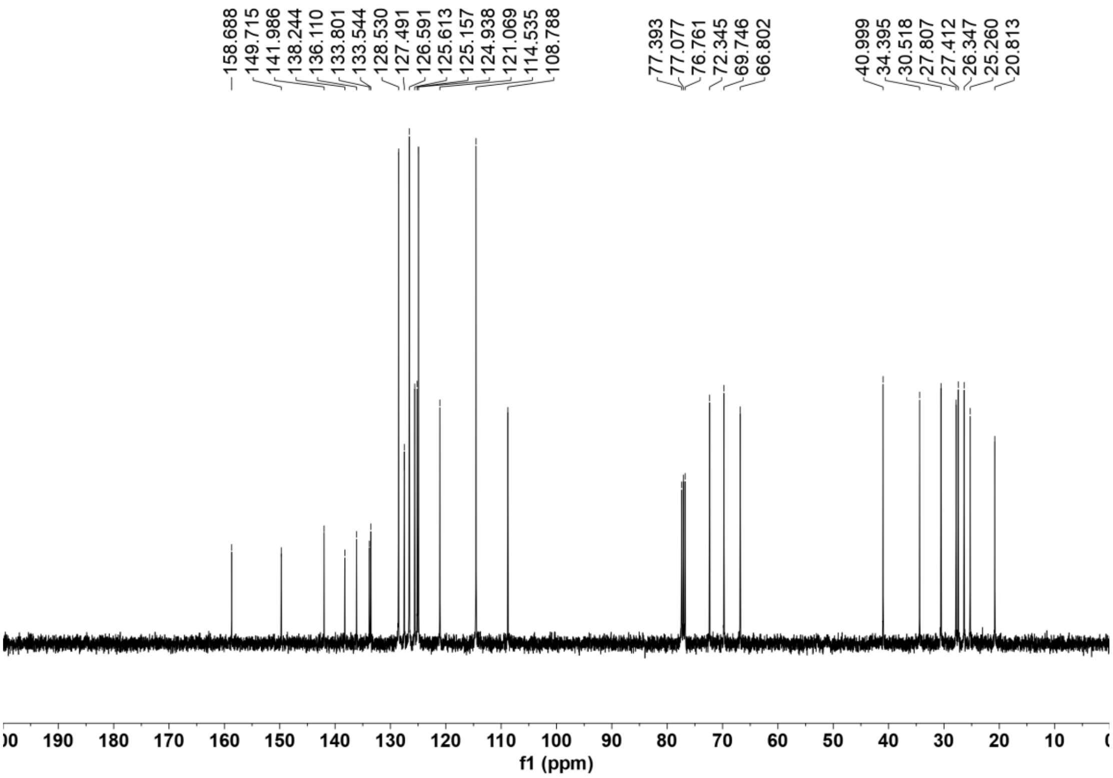

^{1}H NMR of 3aa' (400 MHz, $CDCl_3$)

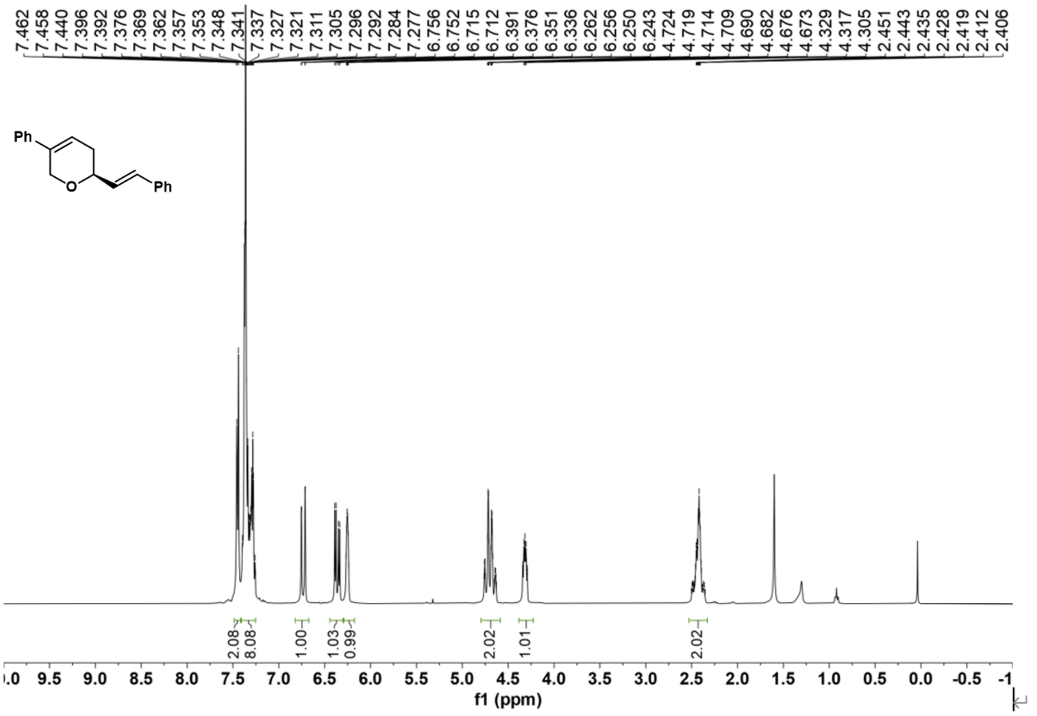

^{13}C NMR of 3aa' (100 MHz, $CDCl_3$)

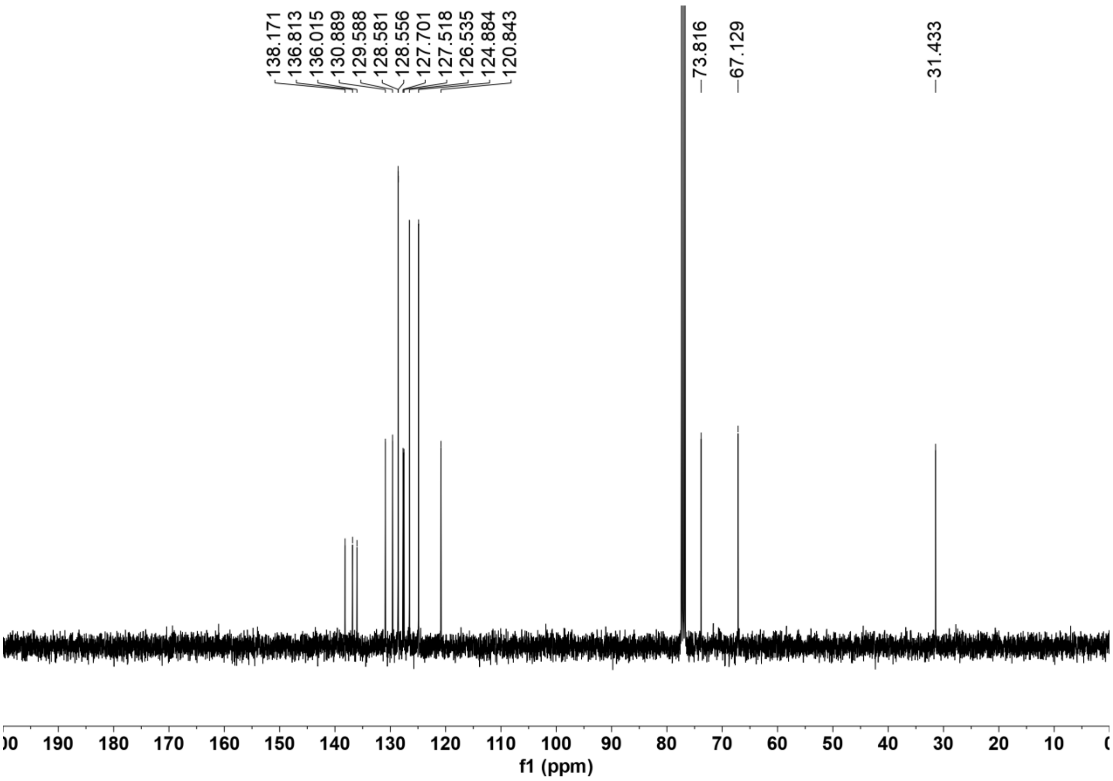

^{1}H NMR of 4 (400 MHz, $CDCl_3$)

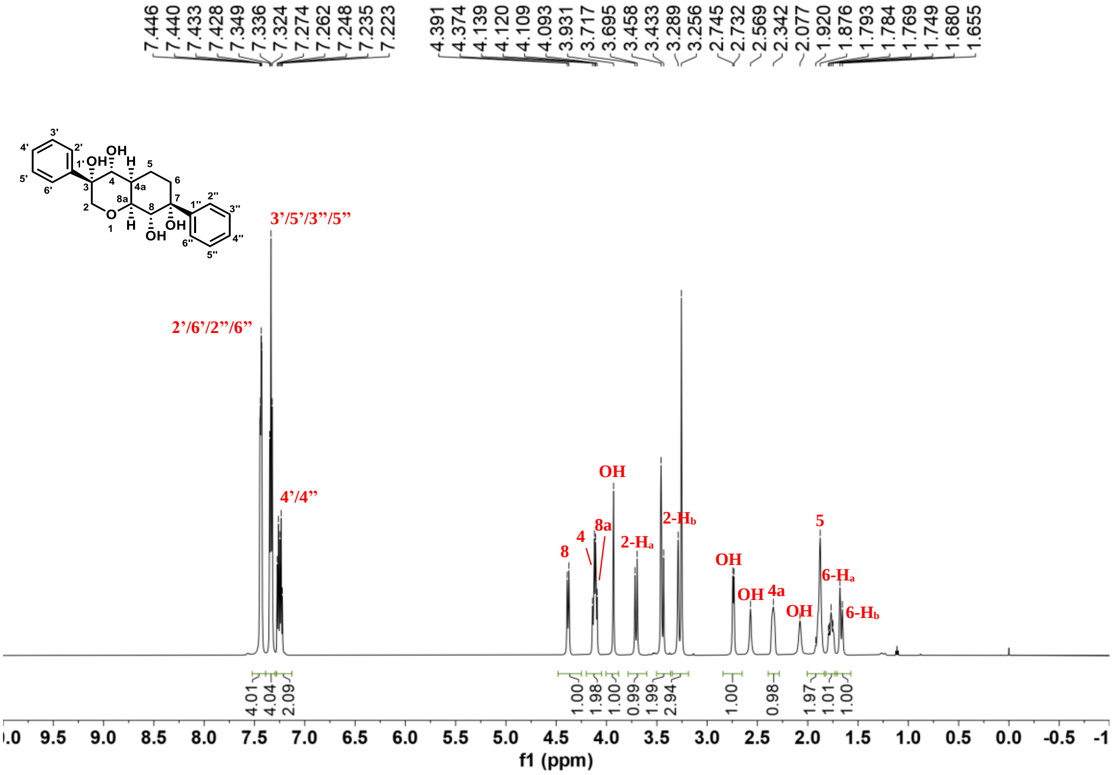

^{13}C NMR of 4 (100 MHz, $CDCl_3$)

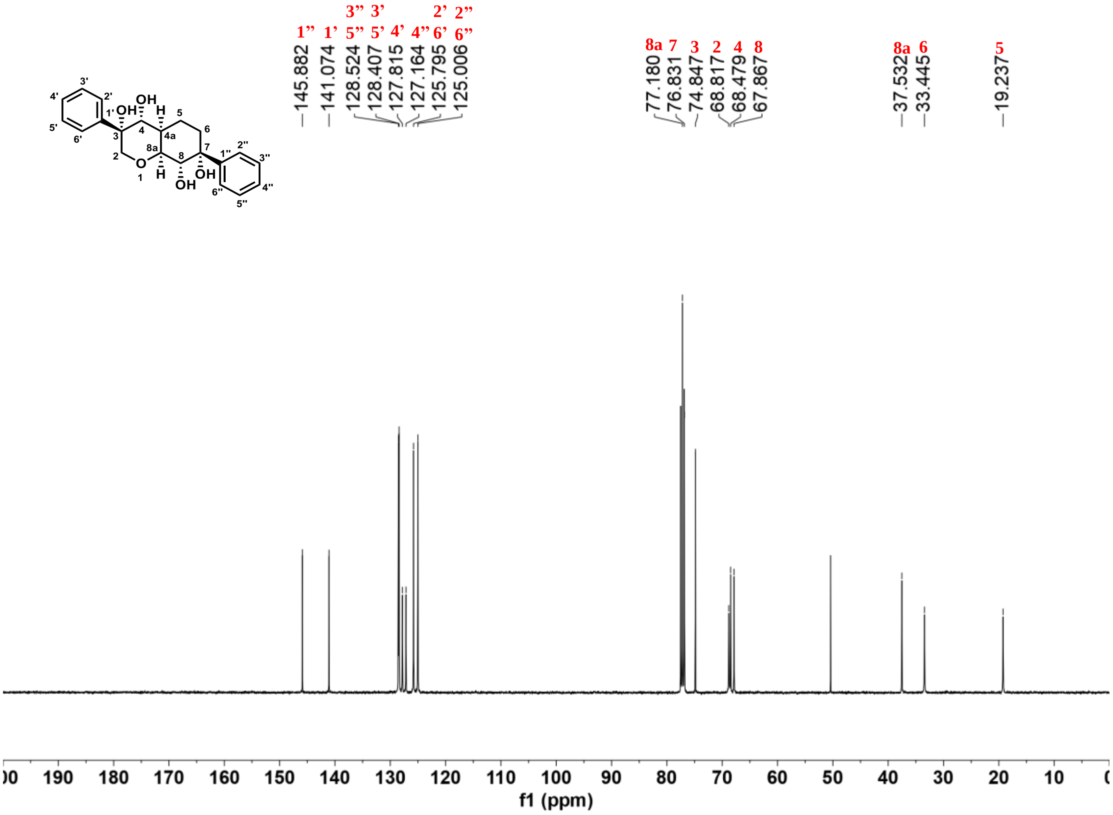

^1H-^1H COSY spectrum of 4 (400 MHz, $CDCl_3$)

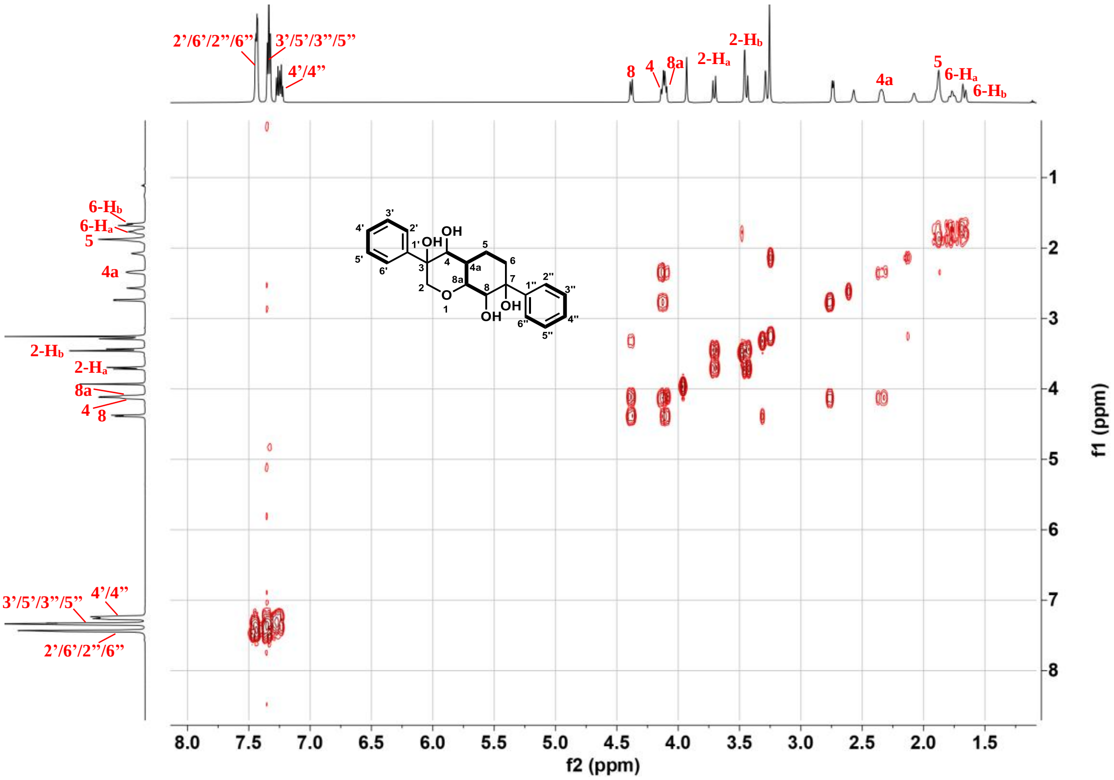

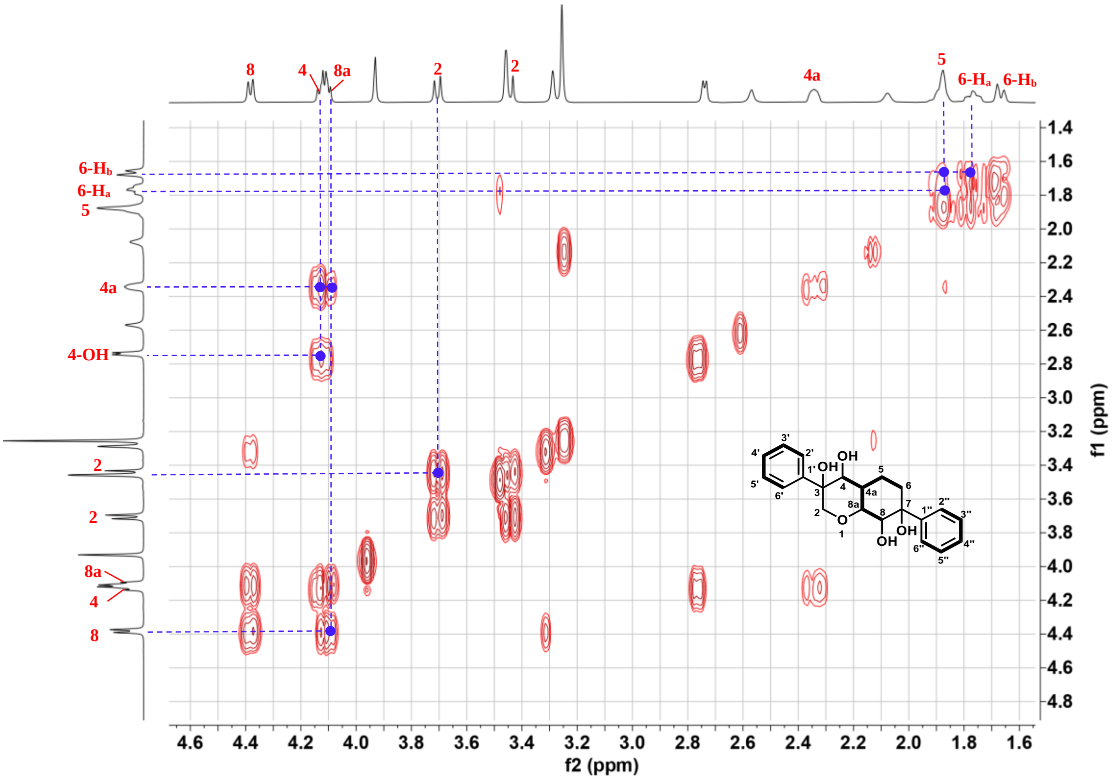

HMQC spectrum of 4 (400/100 MHz, $CDCl_3$)

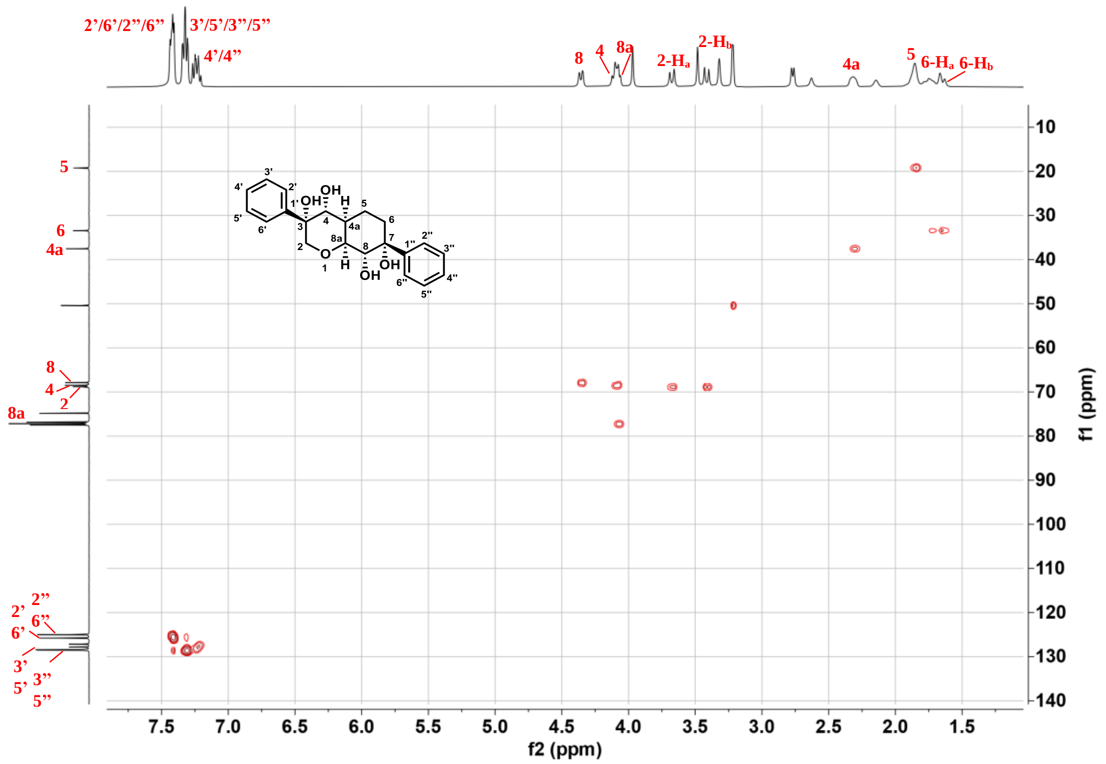

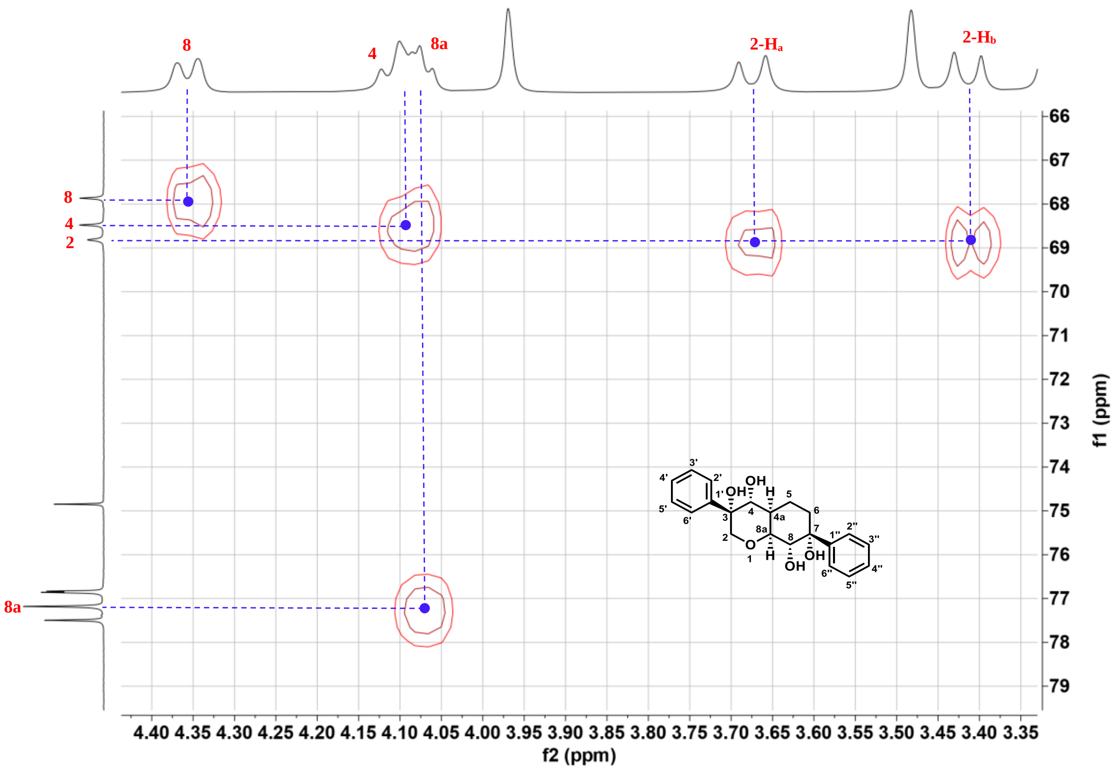

HMBC spectrum of 4 (400/100 MHz, $CDCl_3$)

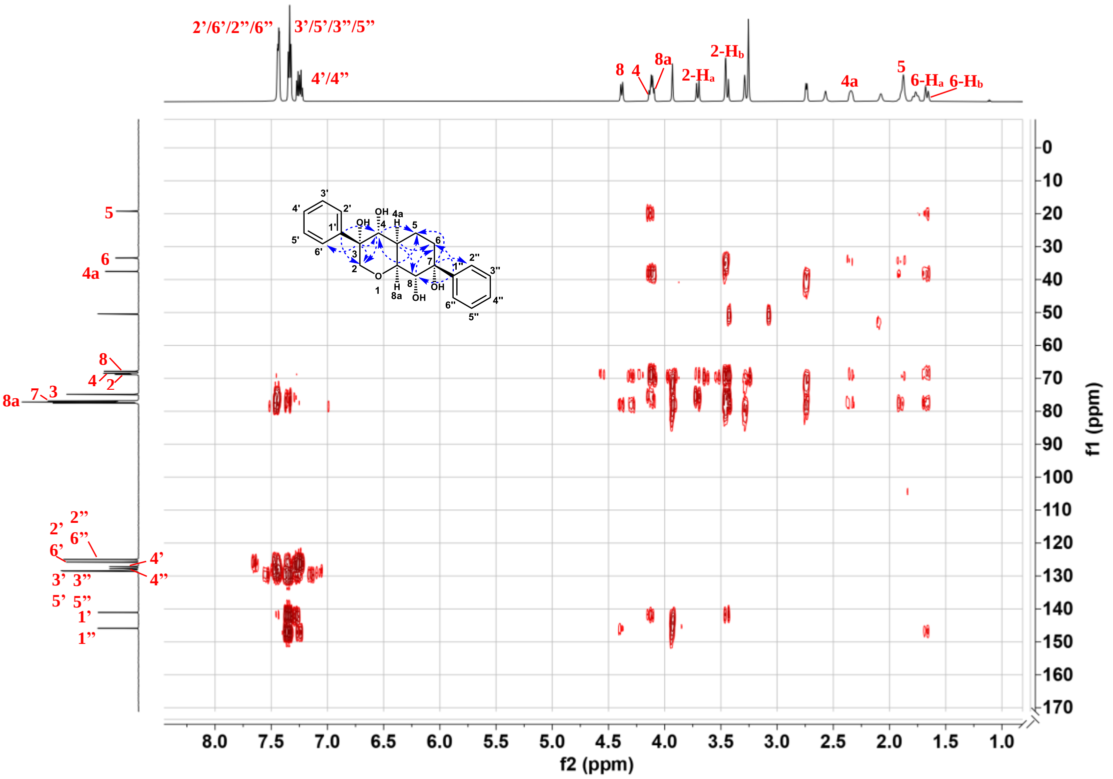

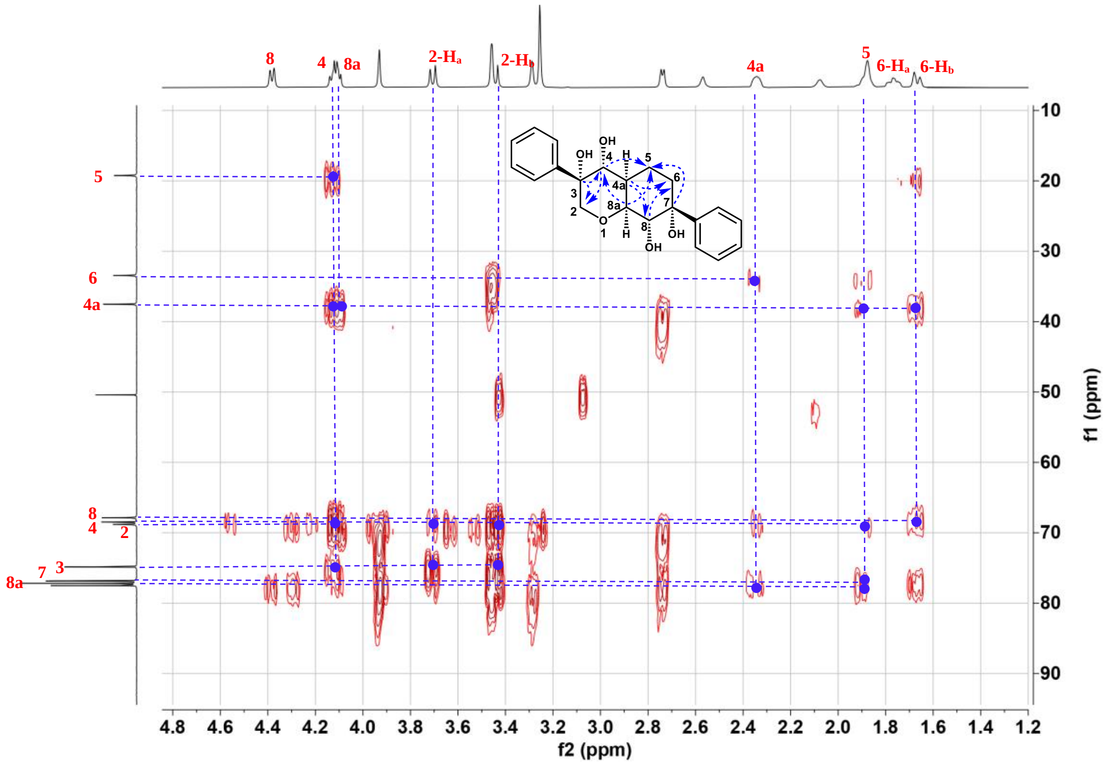

ROESY spectrum of 4 (400 MHz, $CDCl_3$)

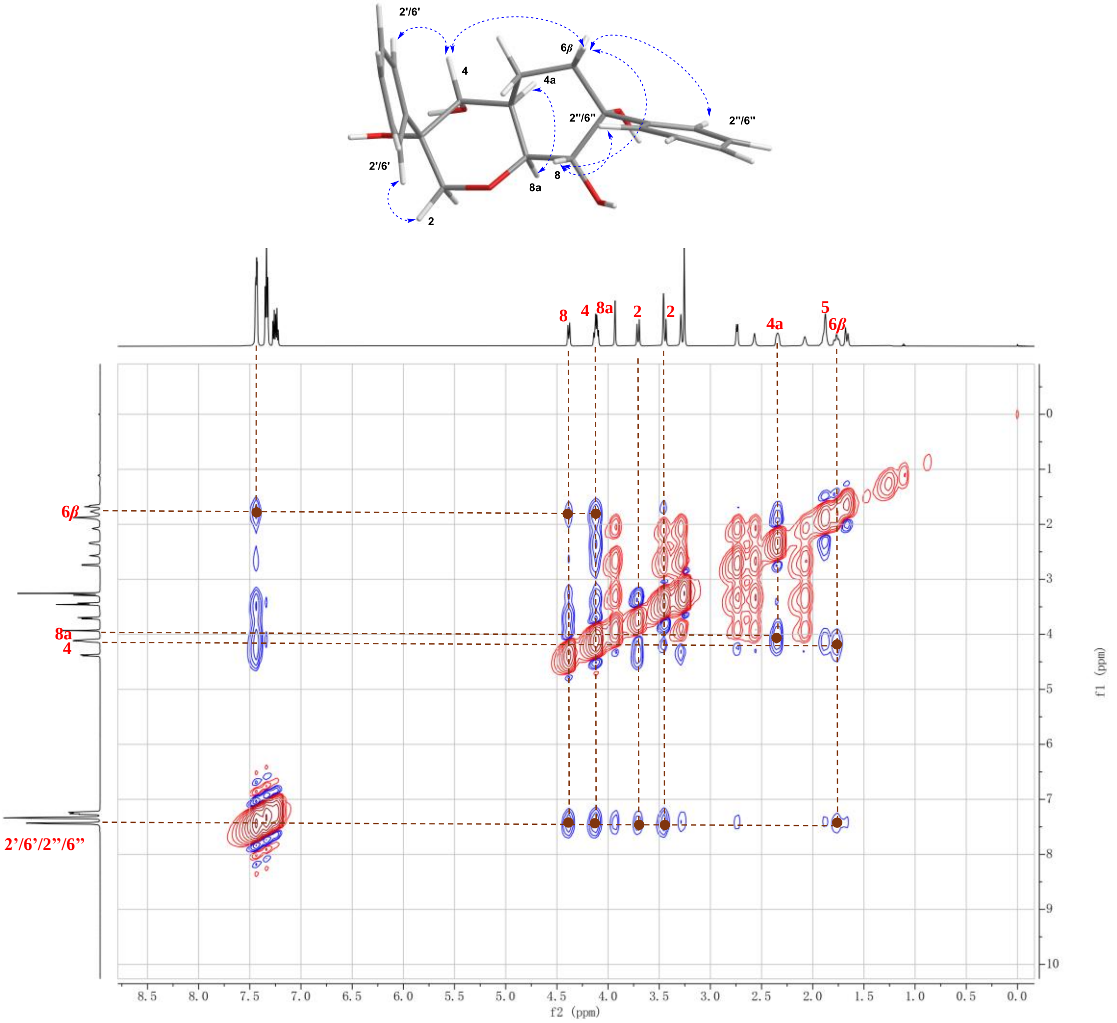

^{1}H NMR of 5 (400 MHz, $CDCl_3$)

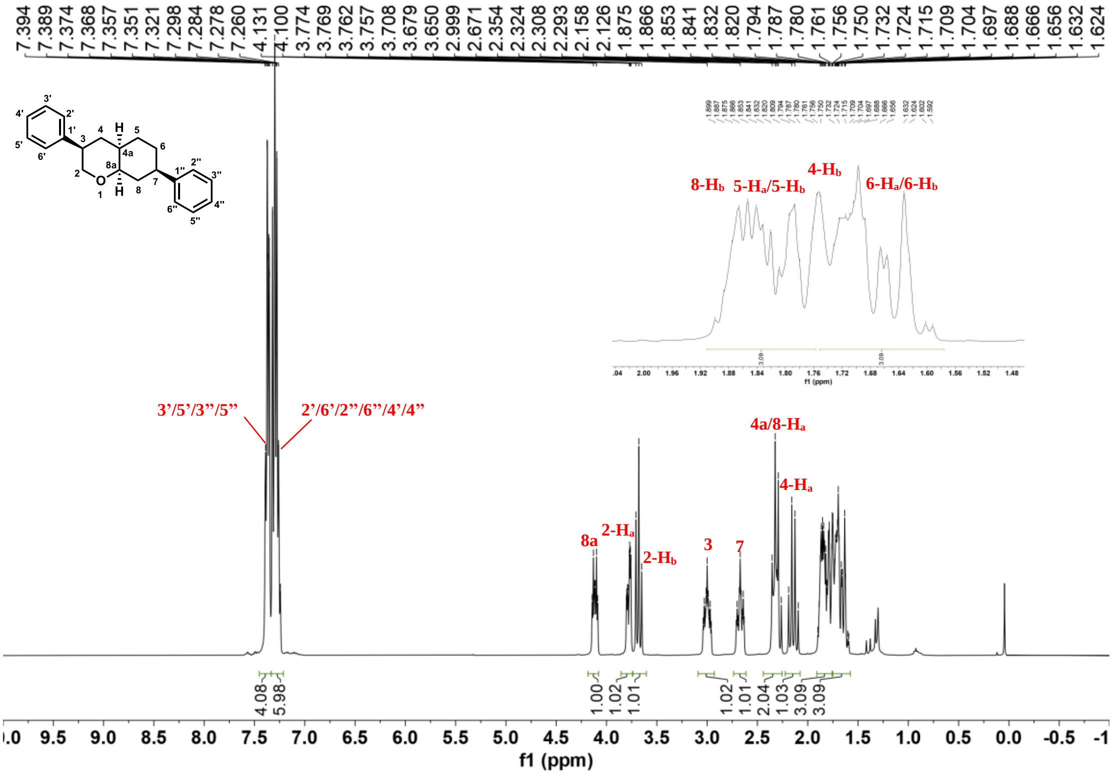

^{13}C NMR of 5 (100 MHz, $CDCl_3$)

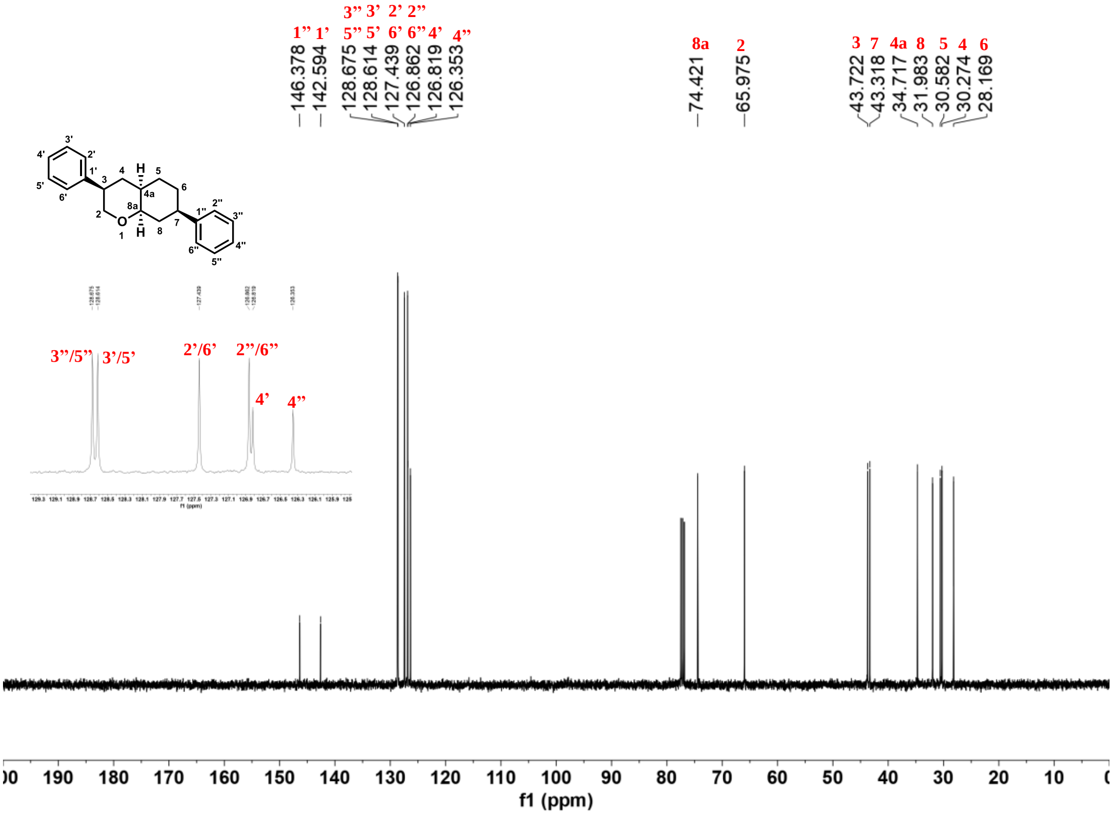

^{1}H-^{1}H COSY spectrum of 5 (400 MHz, $CDCl_3$)

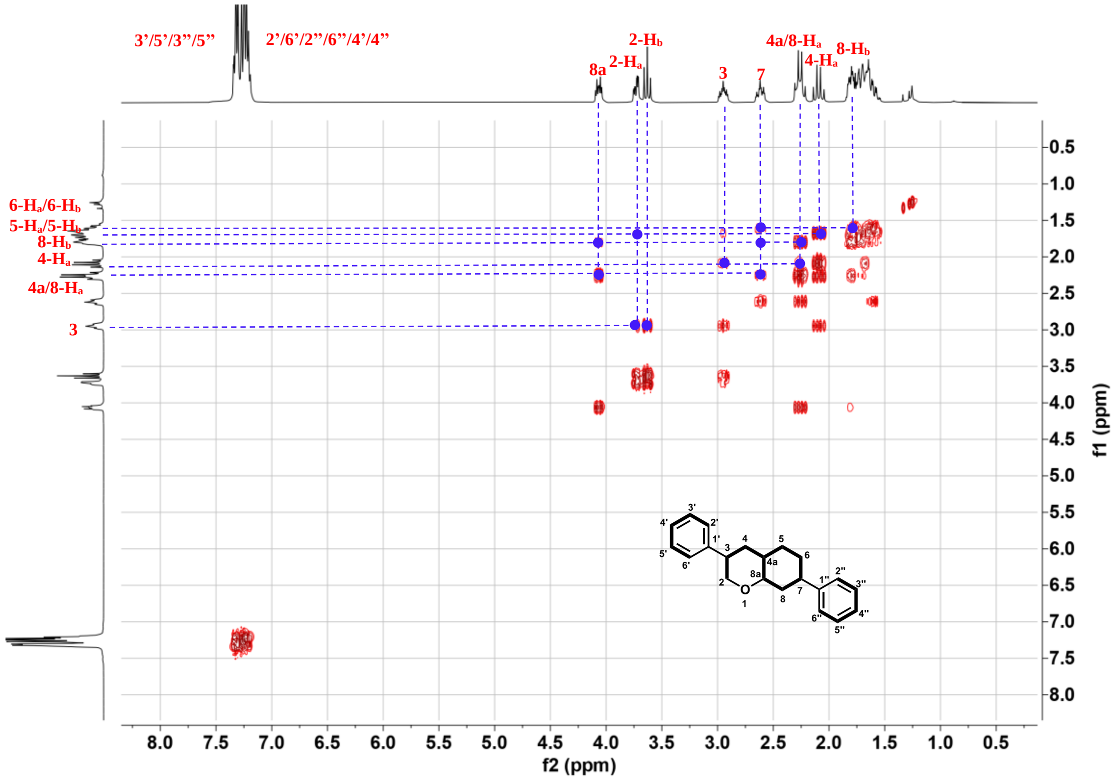

HMQC spectrum of 5 (400/100 MHz, $CDCl_3$)

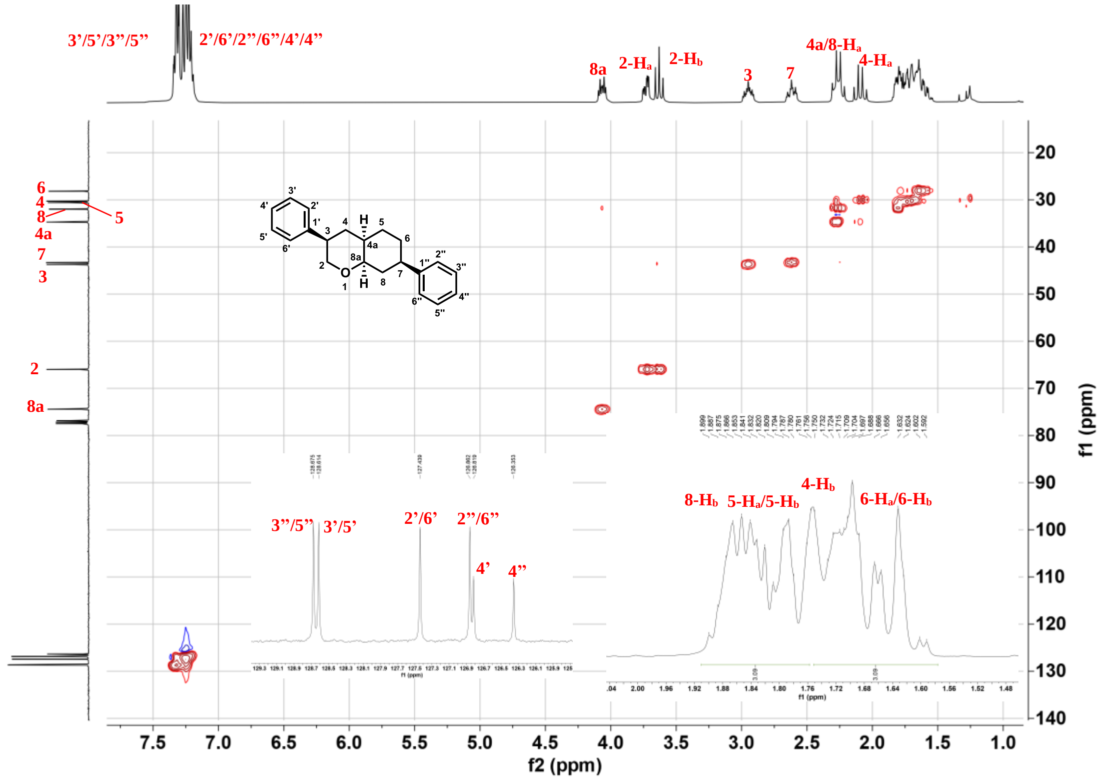

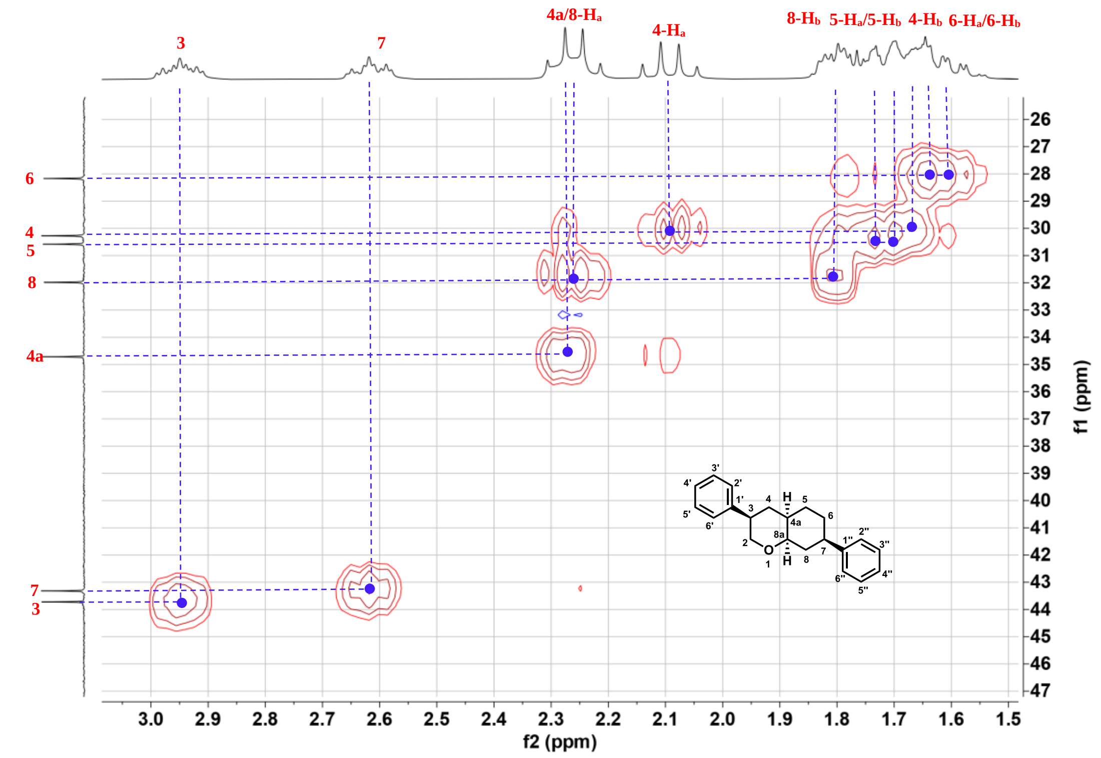

HMBC spectrum of 5 (400/100 MHz, $CDCl_3$)

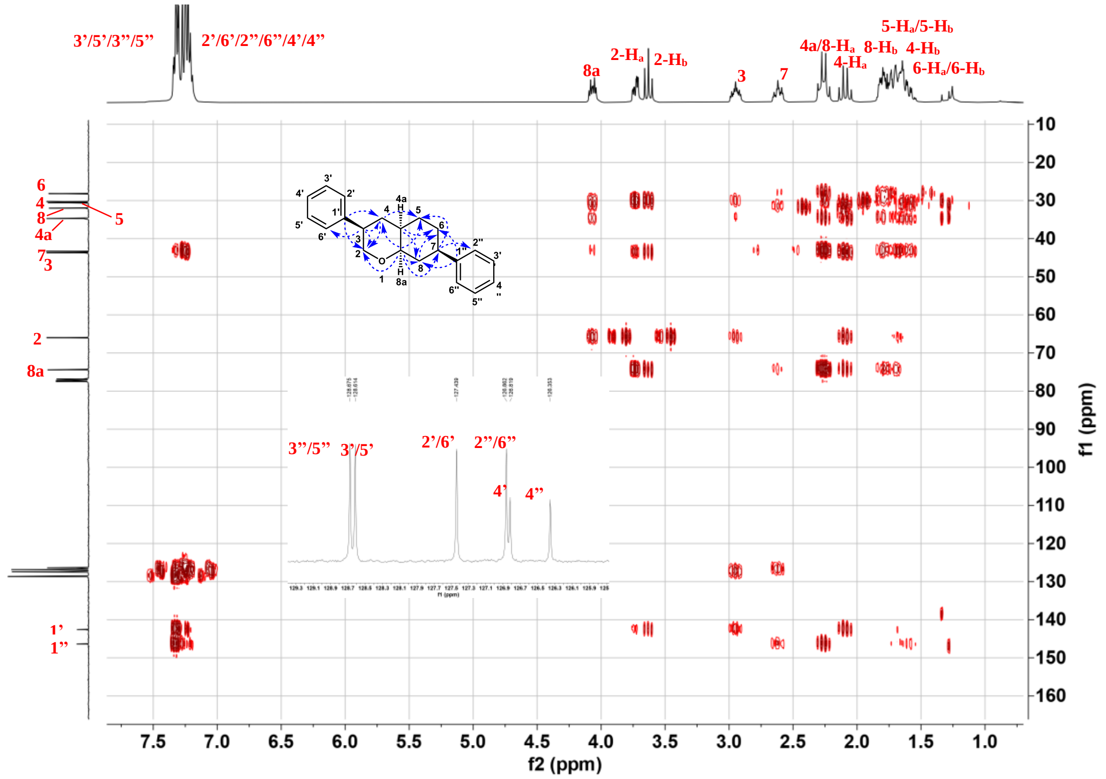

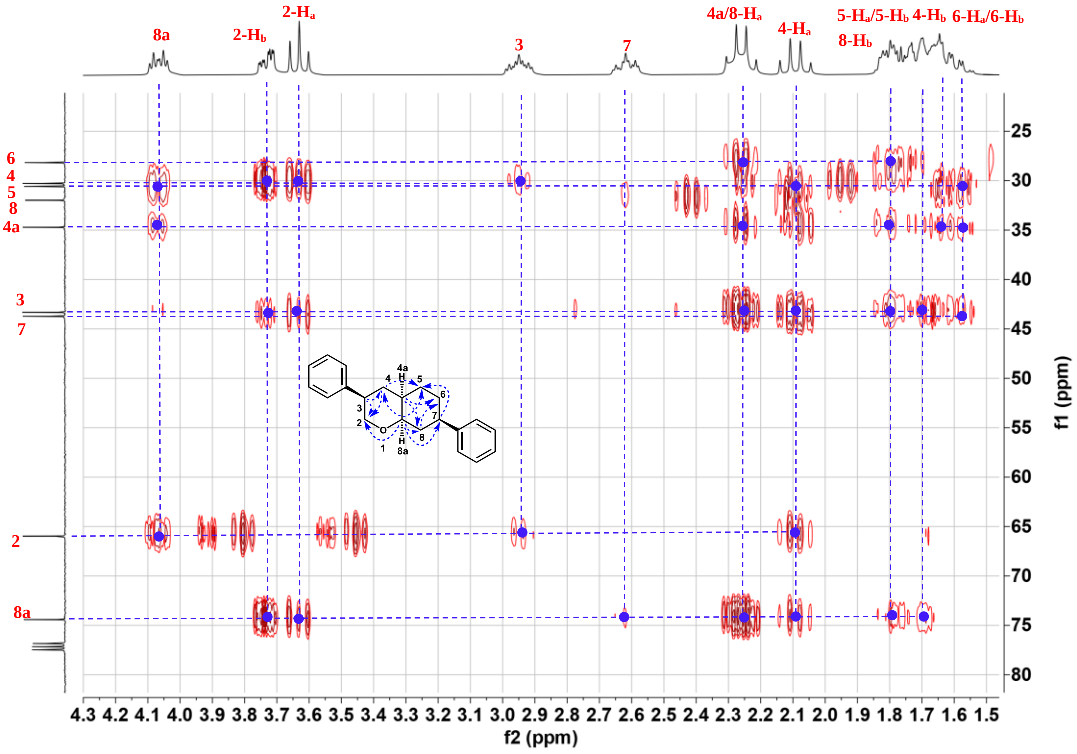
8a
2-Hb
2-Ha
3
7
4a/8-Ha
4-Ha
5-Ha/5-Hb
8-Hb
4-Hb
6-Ha/6-Hb
6
4
5
8
4a
3
7
2
8a
f1 (ppm)
f2 (ppm)

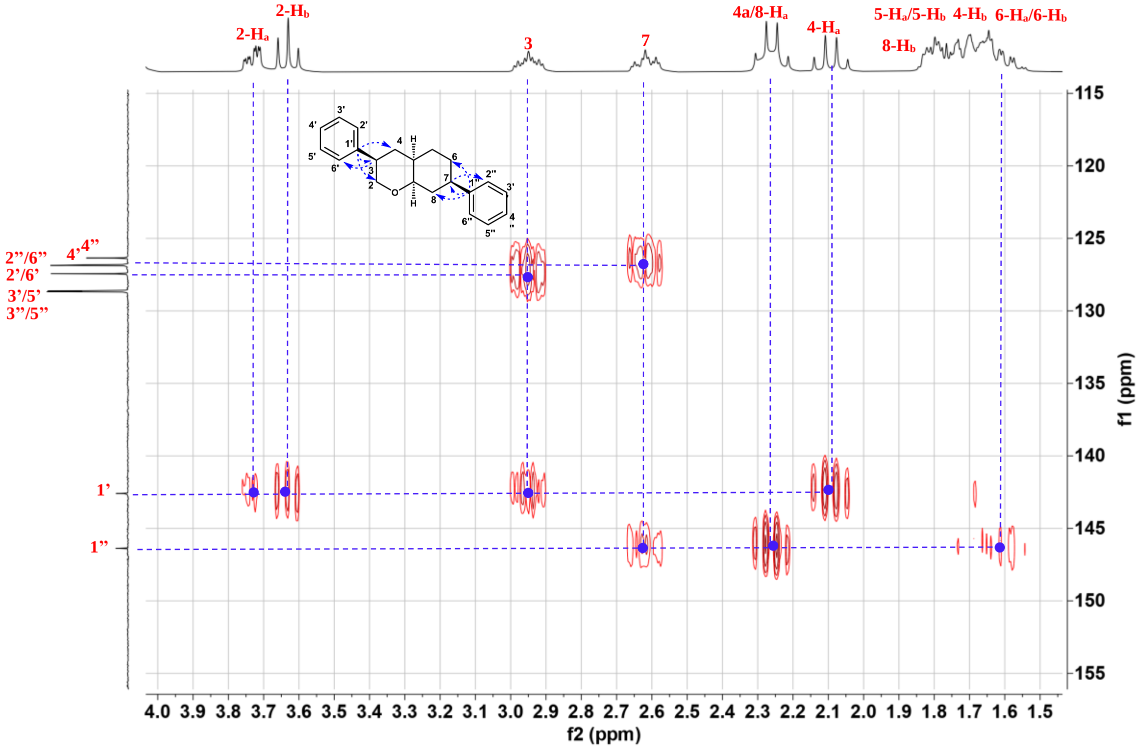
2-Ha
2-Hb
3
7
4a/8-Ha
4-Ha
5-Ha/5-Hb
8-Hb
4-Hb
6-Ha/6-Hb
4'4''
2''/6''
2'/6'
3'/5'
3''/5''
1'
1''
f1 (ppm)
f2 (ppm)

ROESY spectrum of 5 (400 MHz, $CDCl_3$)

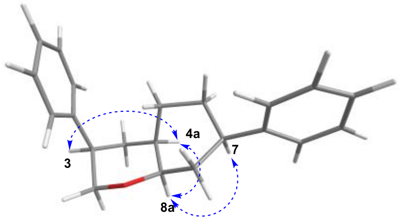

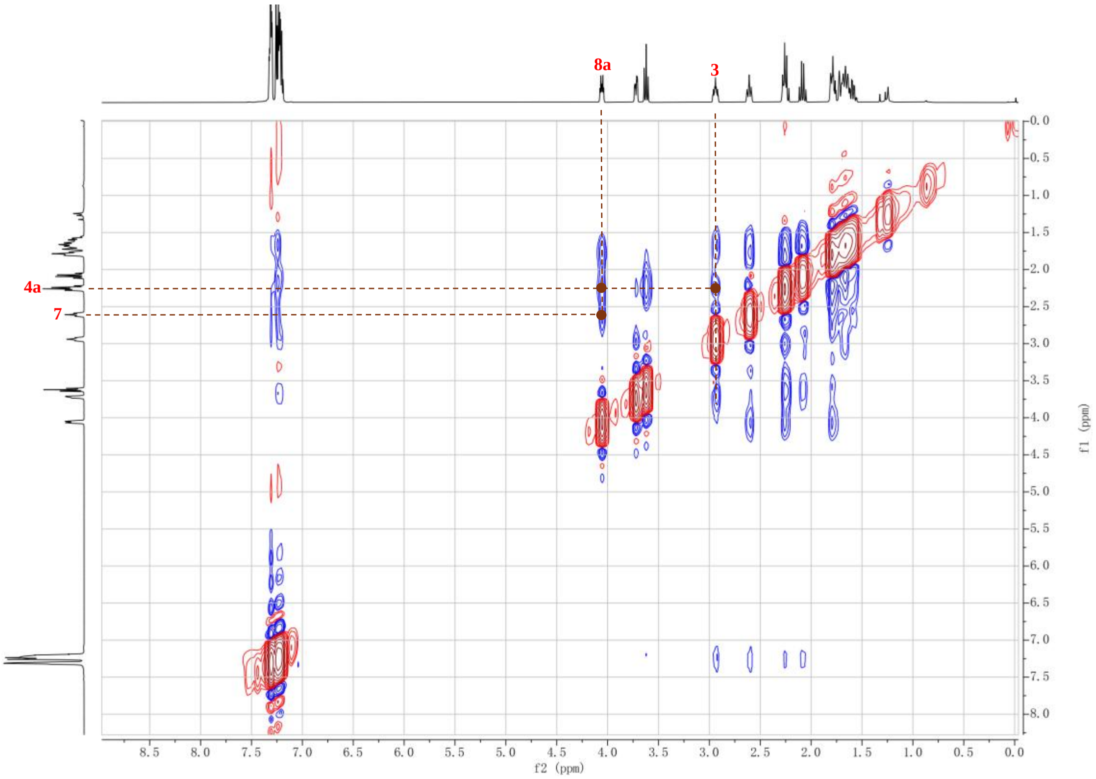

^{1}H NMR of 6 (400 MHz, $CDCl_3$)

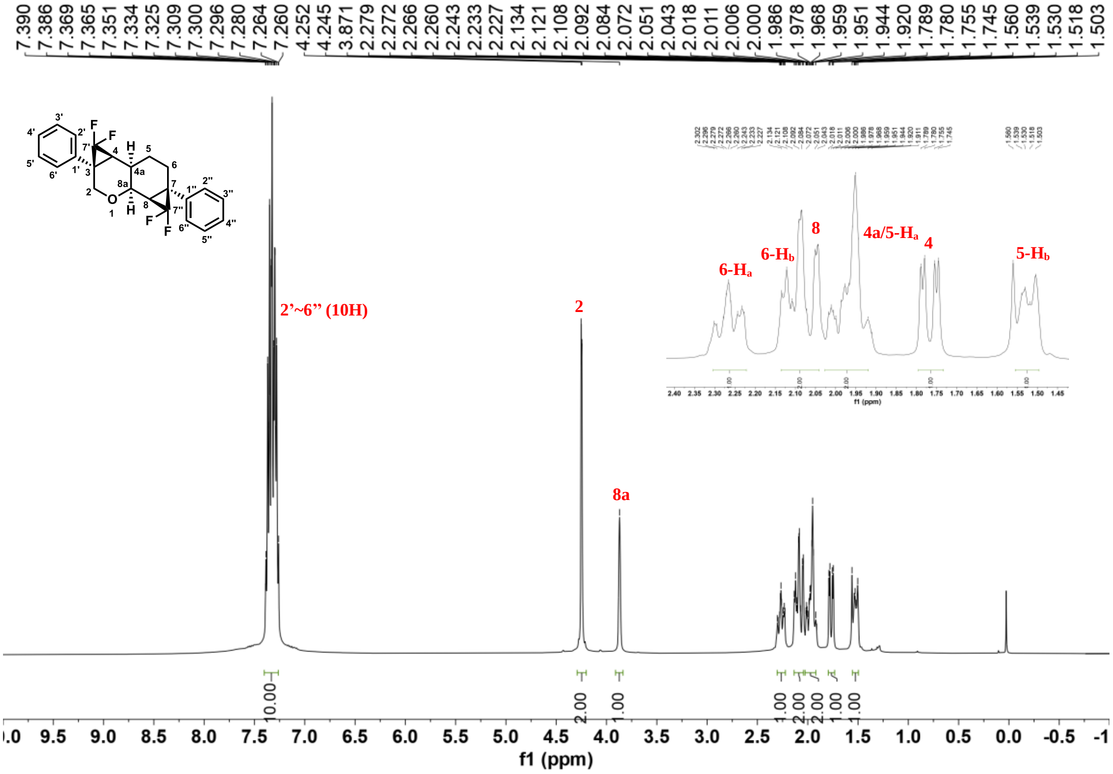

^{13}C NMR of 6 (100 MHz, $CDCl_3$)

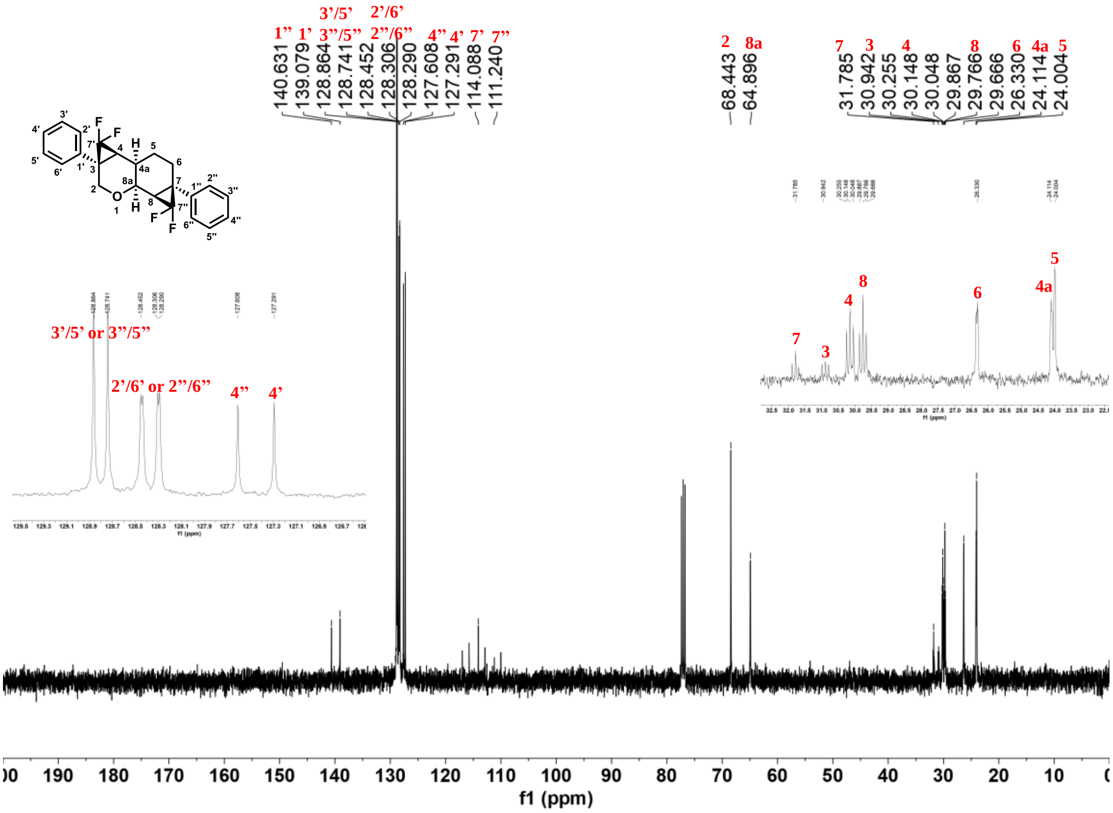

^{19}F NMR of 6 (376 MHz, $CDCl_3$)

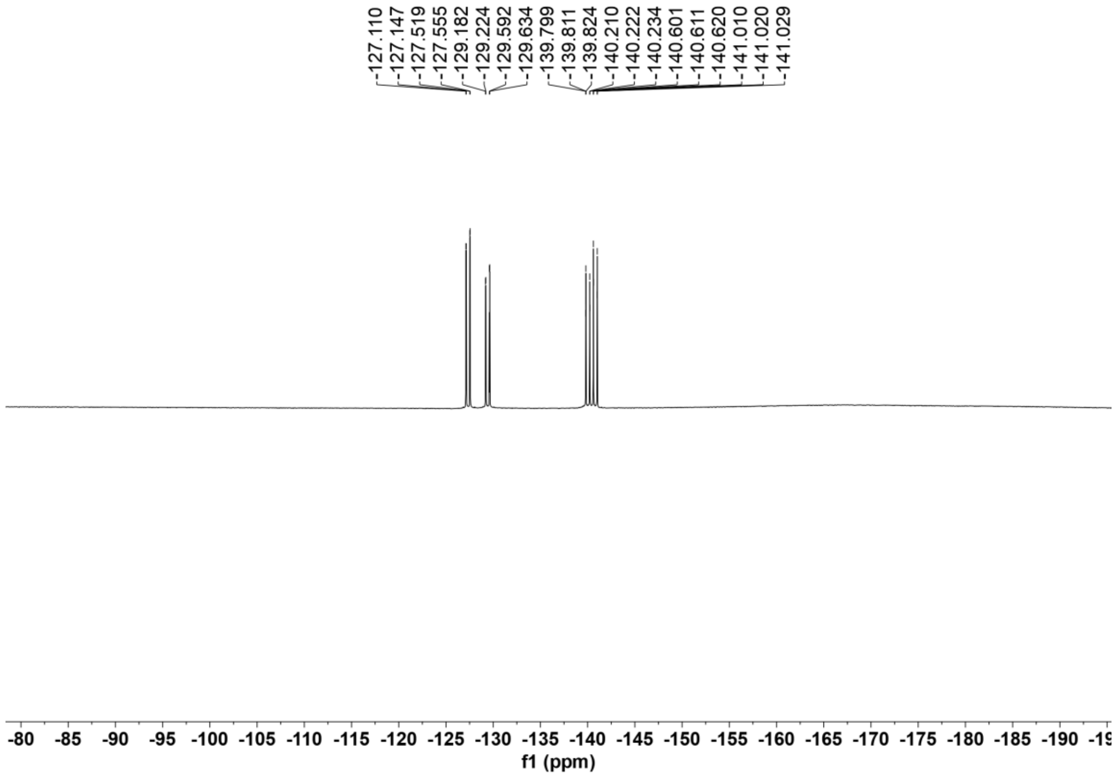

^{1}H-^{1}H COSY spectrum of 6 (400 MHz, $CDCl_3$)

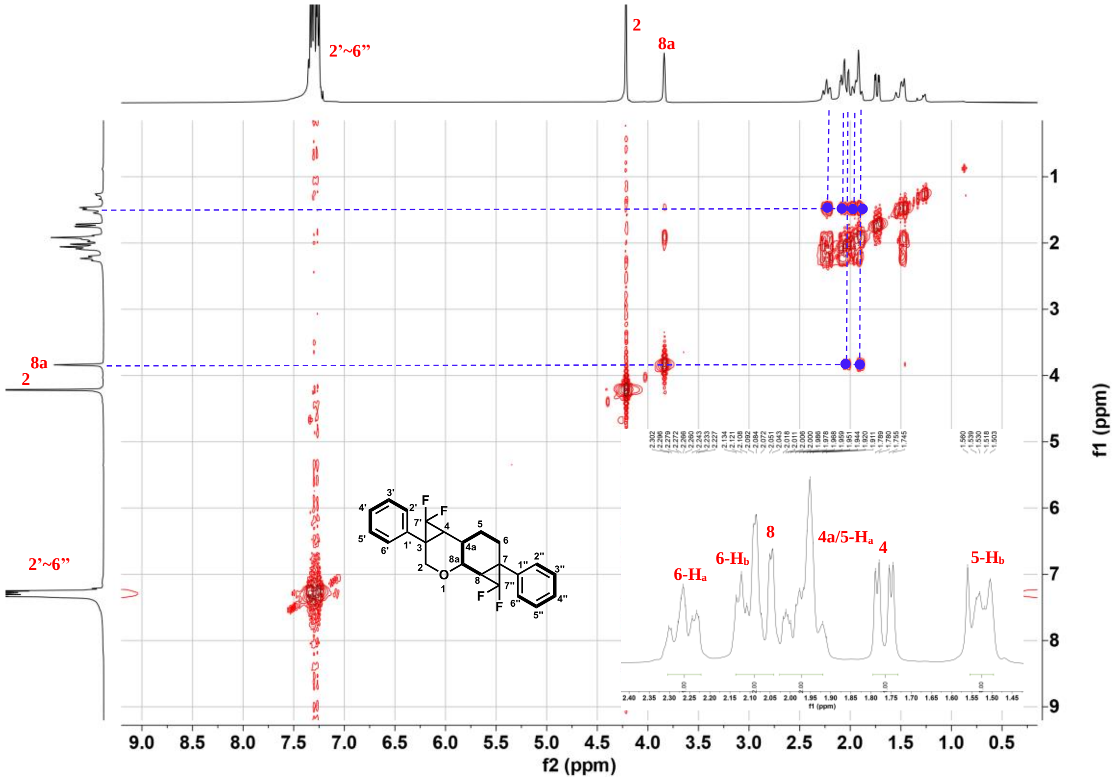

HMQC spectrum of 6 (400/100 MHz, $CDCl_3$)

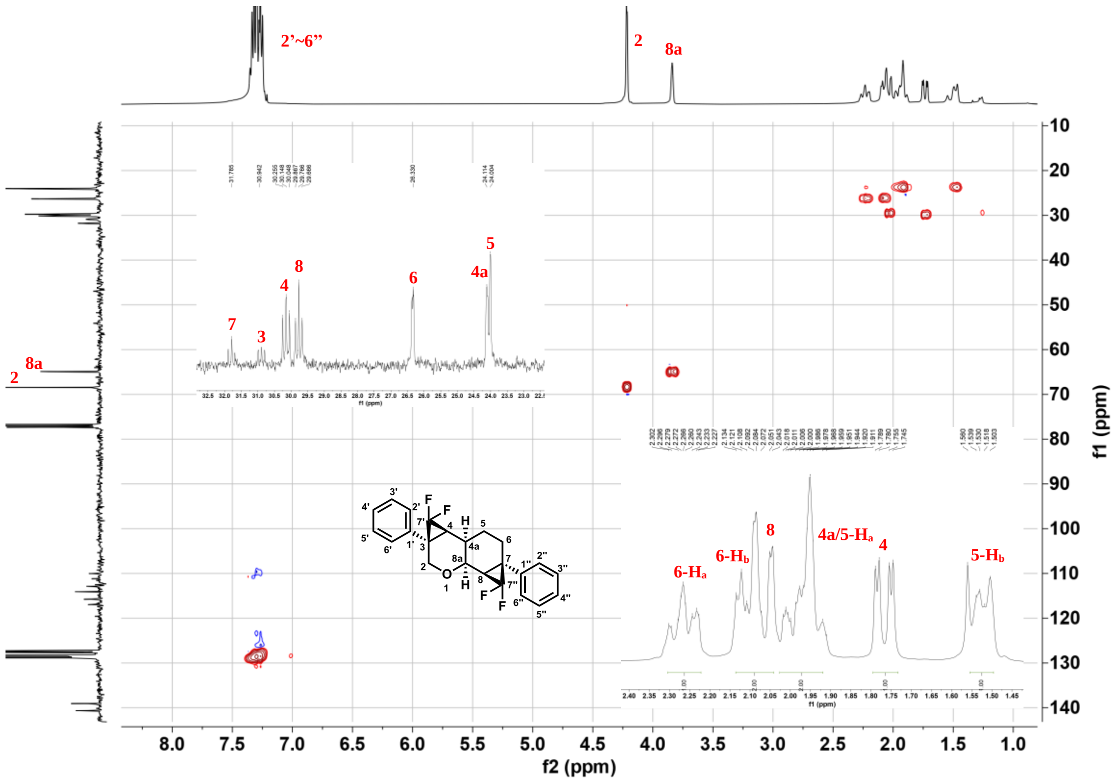

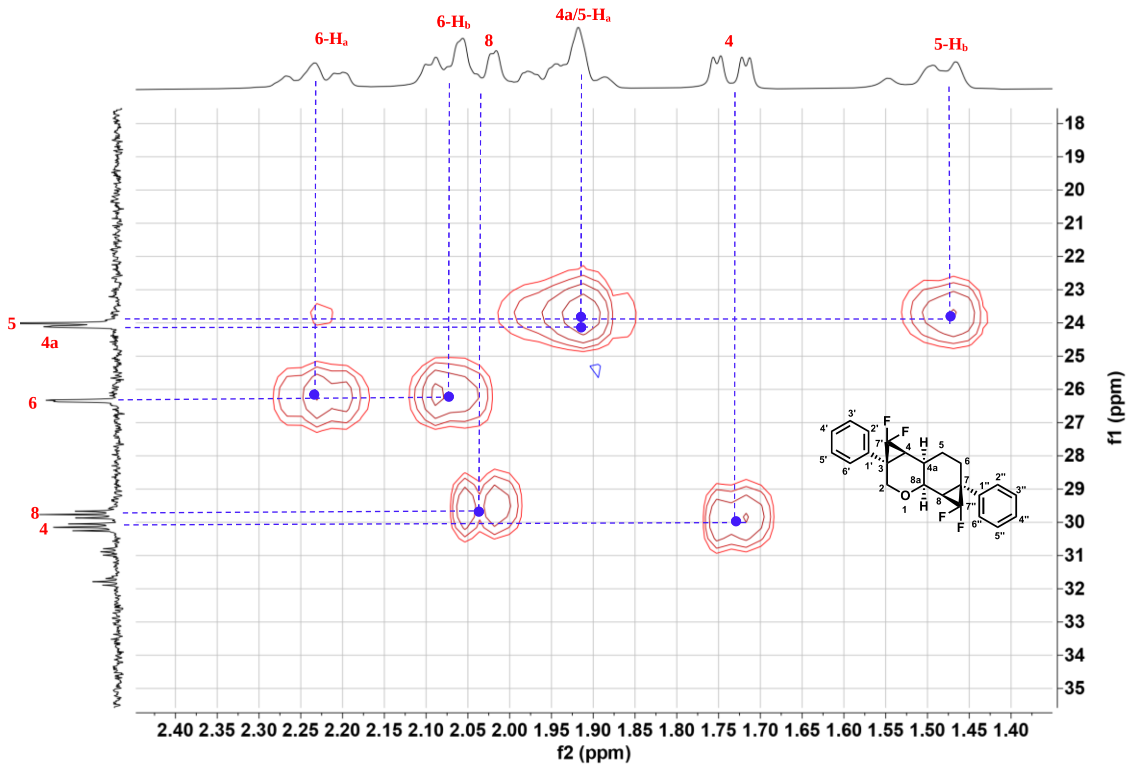

HMBC spectrum of 6 (400/100 MHz, $CDCl_3$)

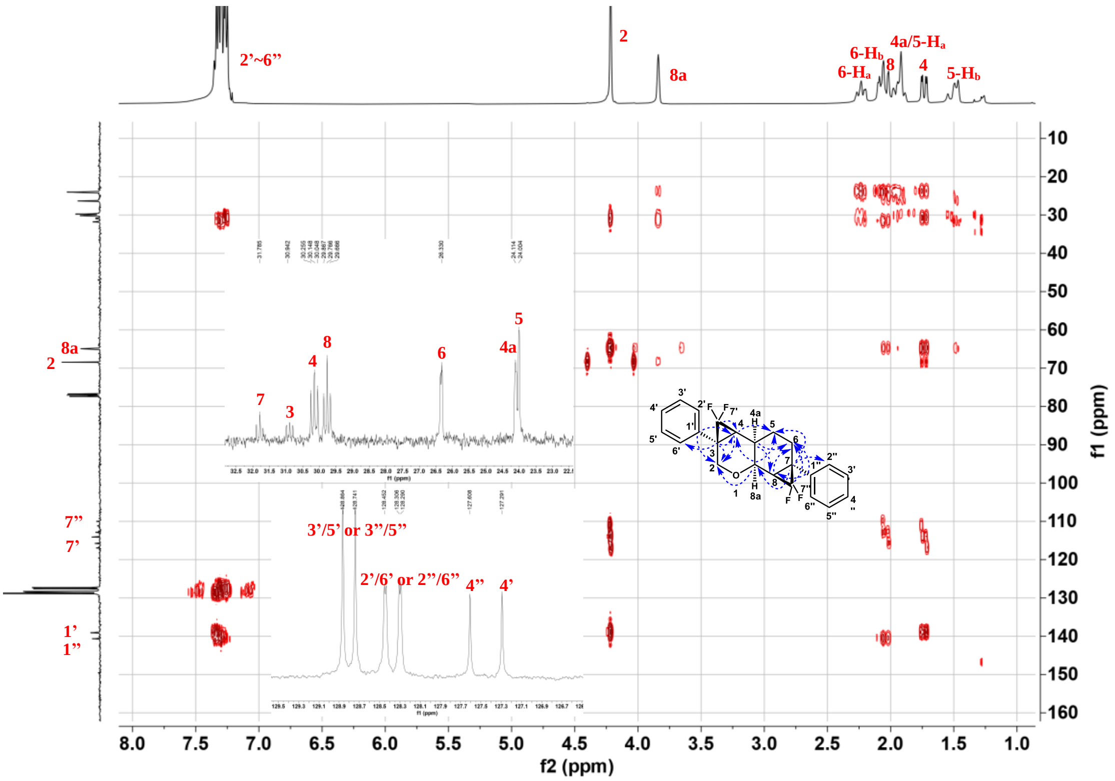

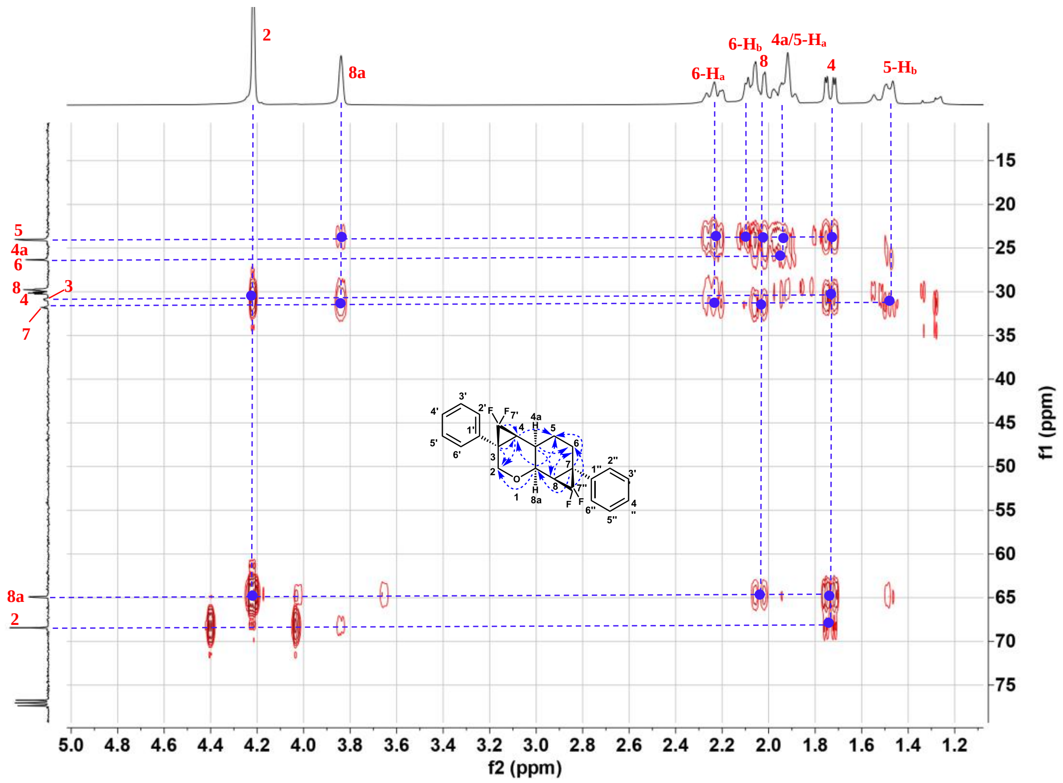

ROESY spectrum of 6 (400 MHz, $CDCl_3$)

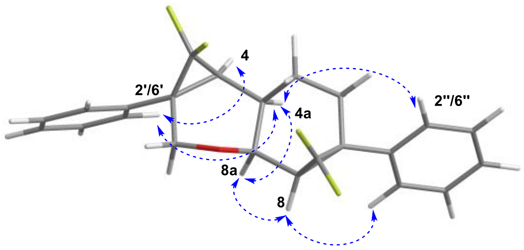

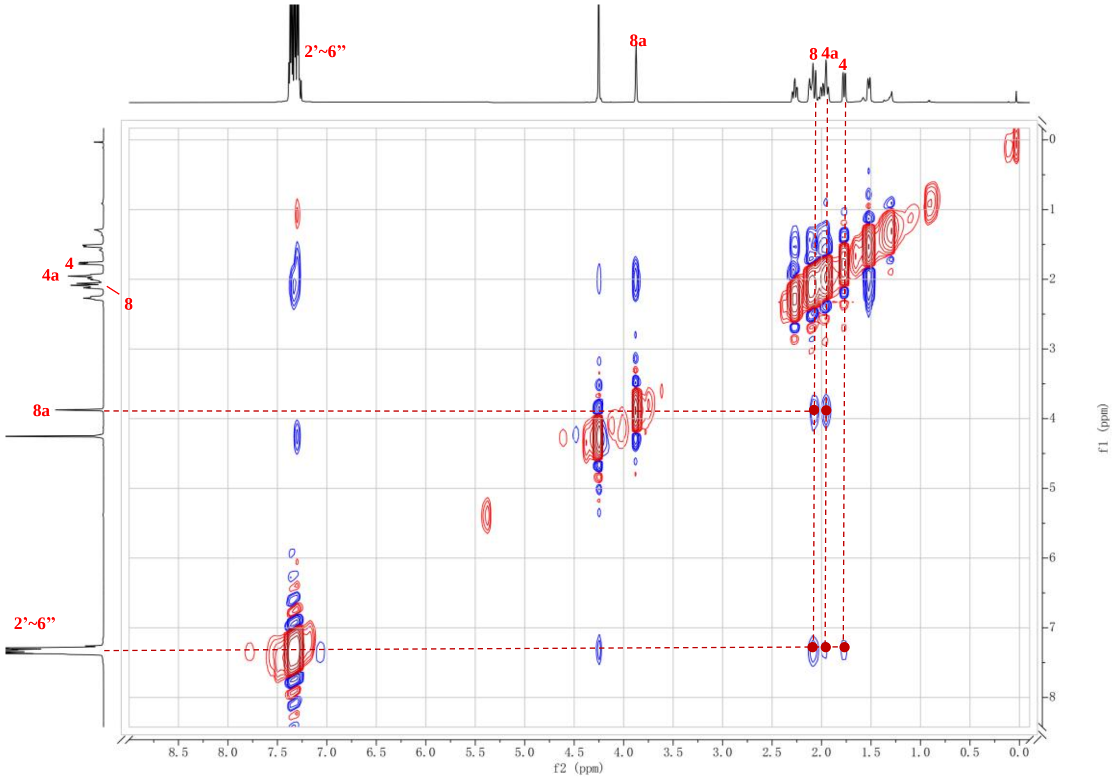

^{1}H NMR of 7 (400 MHz, $CDCl_3$)

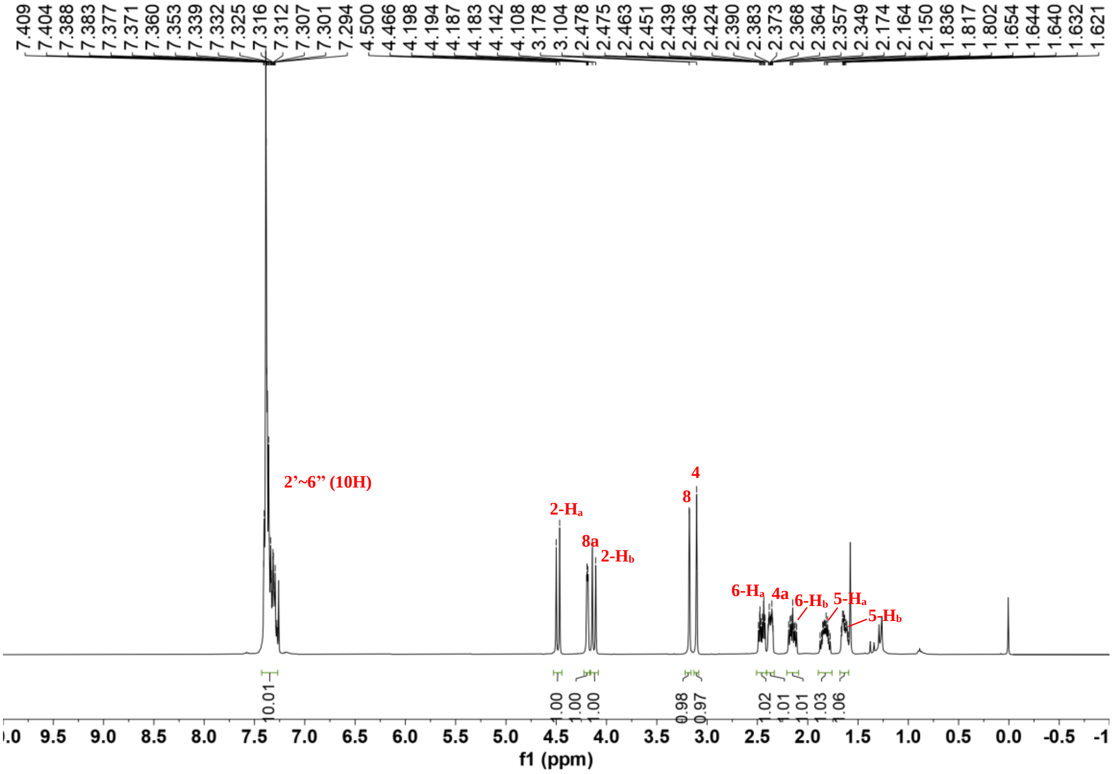

^{13}C NMR of 7 (100 MHz, $CDCl_3$)

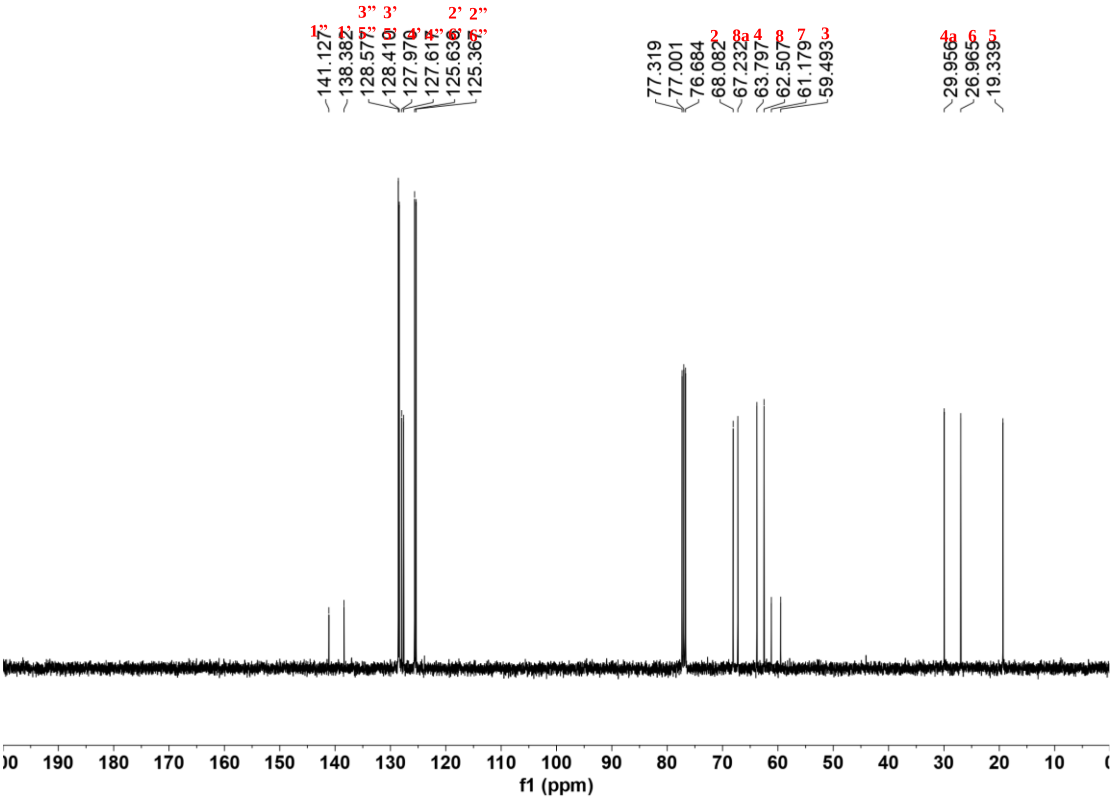

^{1}H-^{1}H COSY spectrum of 7 (400 MHz, $CDCl_3$)

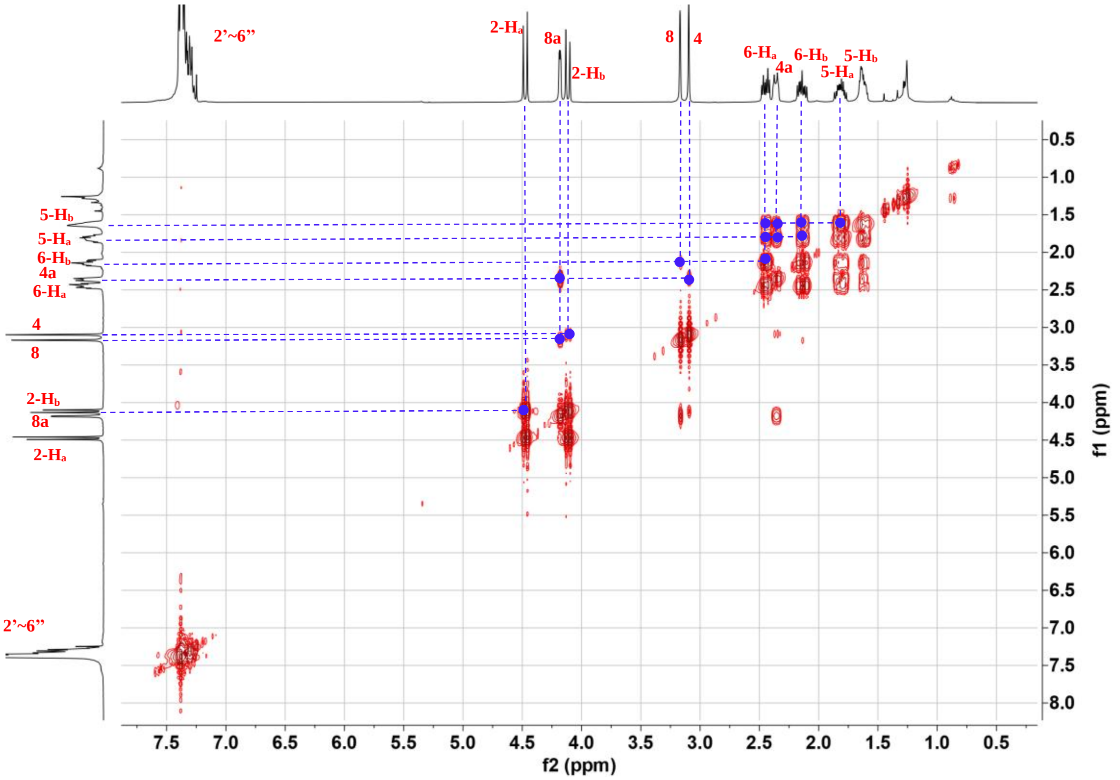

HMQC spectrum of 7 (400/100 MHz, $CDCl_3$)

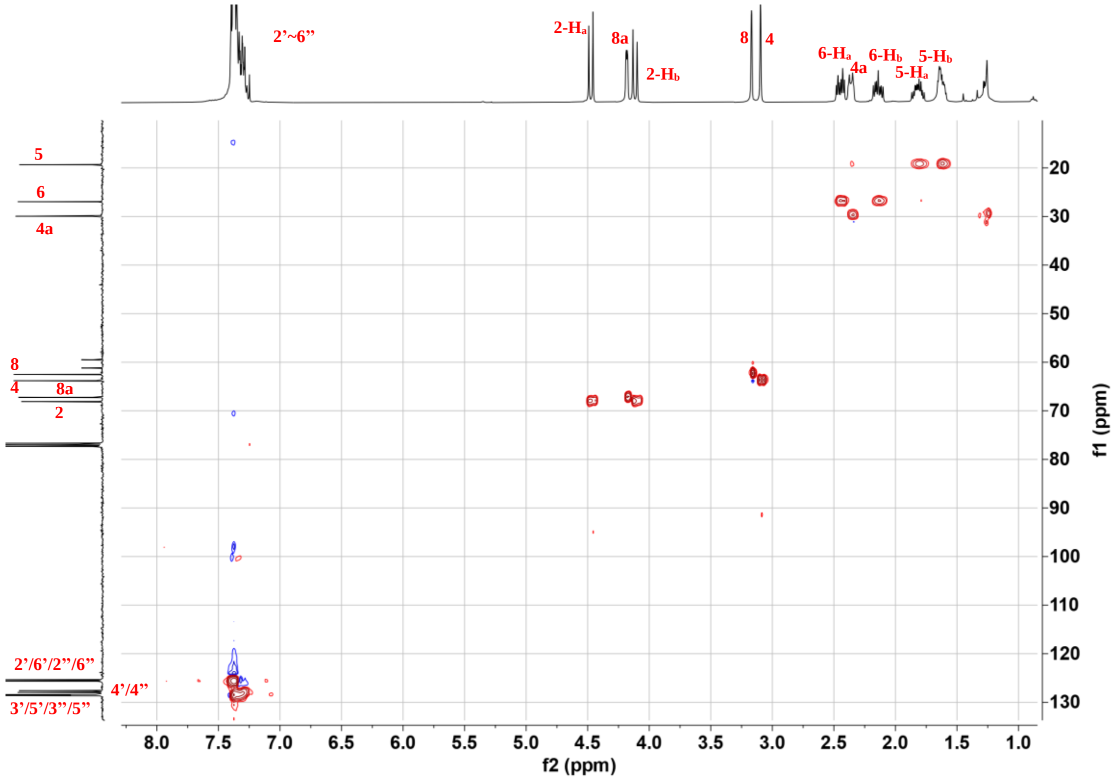

HMBC spectrum of 7 (400/100 MHz, $CDCl_3$)

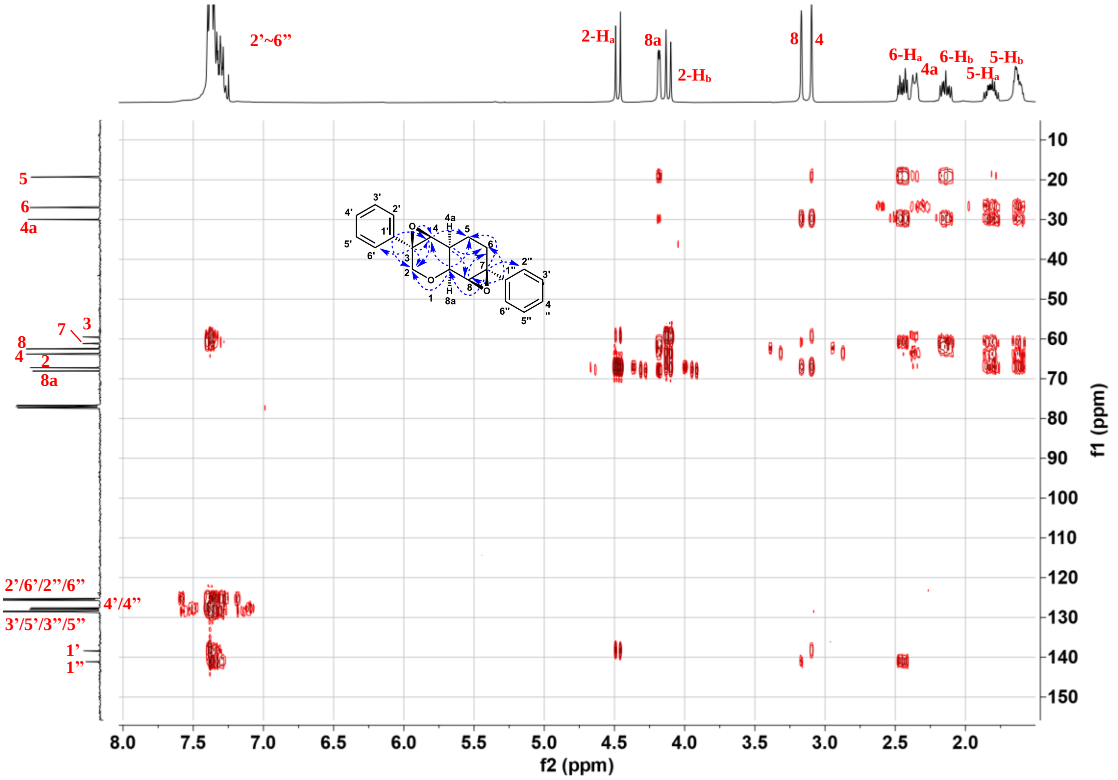

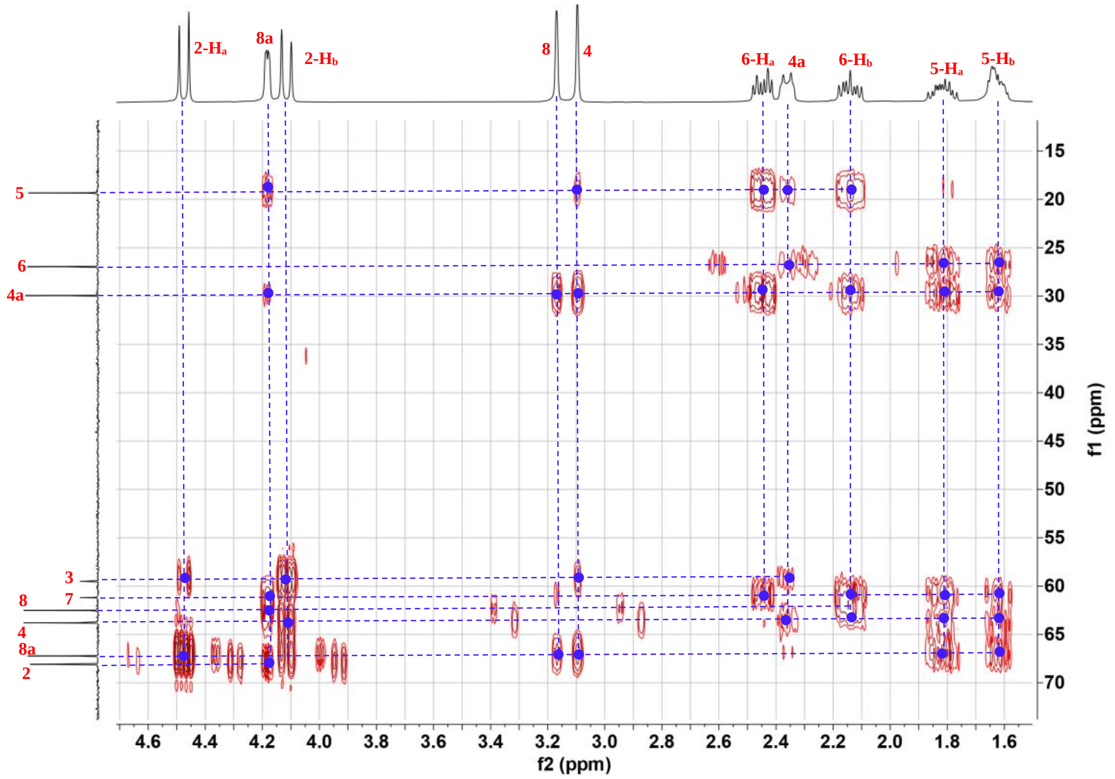

ROESY spectrum of 7 (400 MHz, $CDCl_3$)

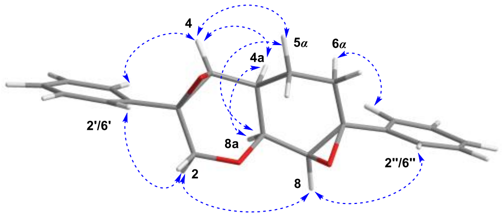

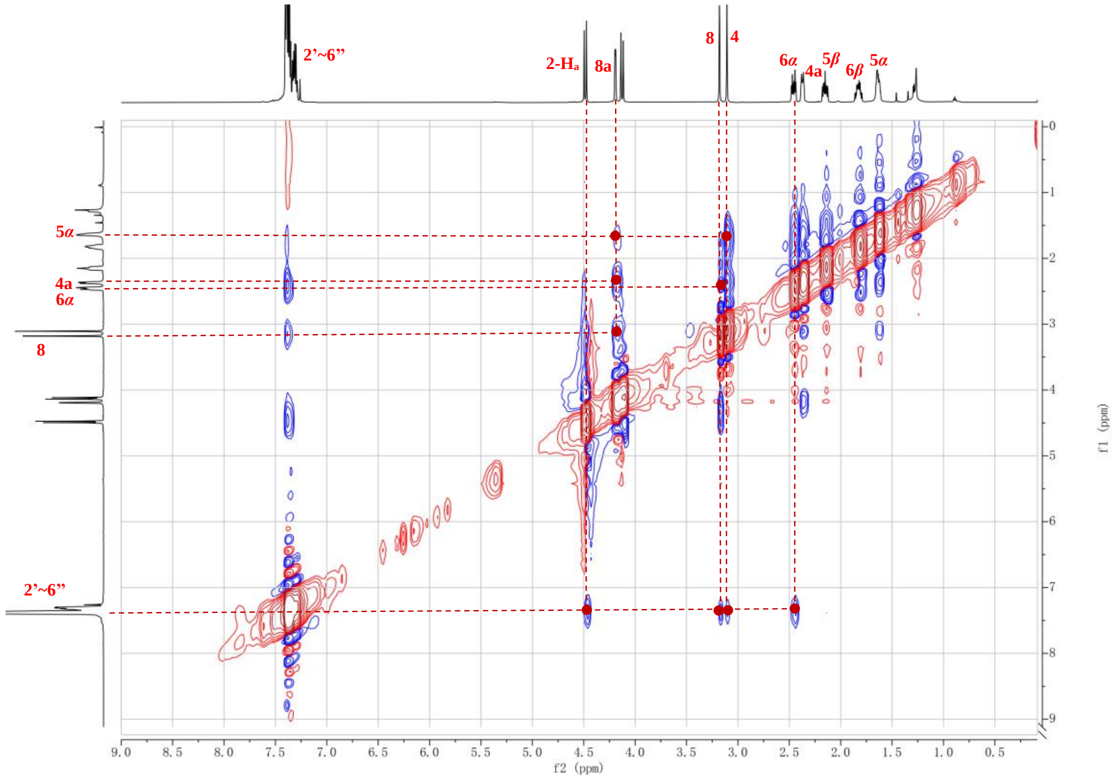

^{1}H NMR of S-3e (400 MHz, $CDCl_3$)

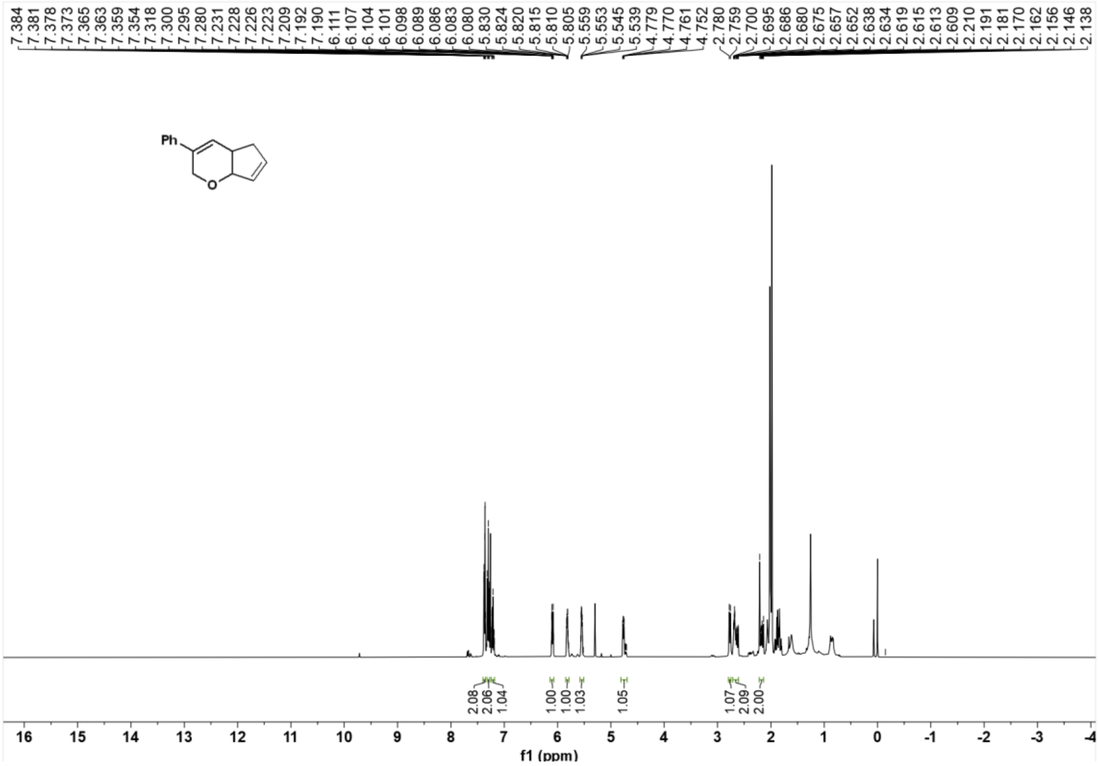

^{13}C NMR of S-3e (100 MHz, $CDCl_3$)

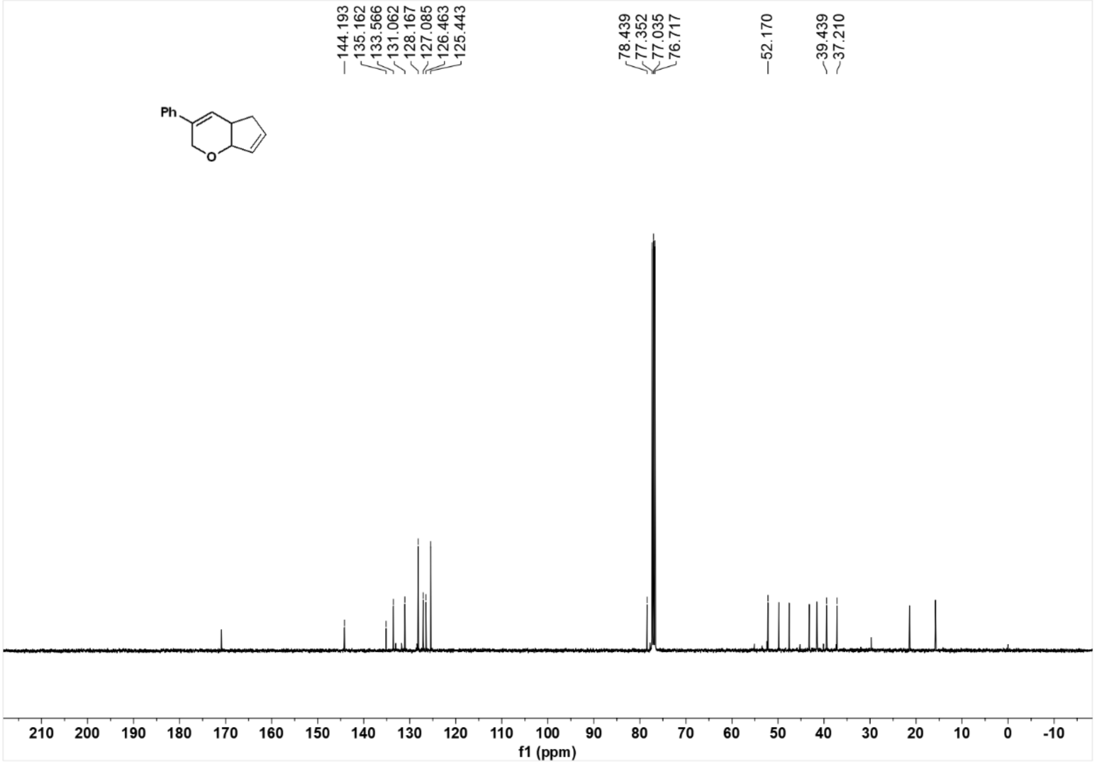

10. References.

1. Zhang, Z.-M. et al. Palladium-Catalyzed Enantioselective Reductive Heck Reactions: Convenient Access to 3,3-Disubstituted 2,3-Dihydrobenzofuran. *Angew. Chem. Int. Ed.* **57**, 10373–10377 (2018).
2. Larock, R. C., Doty, M. J. & Han, X. J. Synthesis of Isocoumarins and α-Pyrones via Palladium-Catalyzed Annulation of Internal Alkynes. *J. Org. Chem.* **64**, 8770–8779 (1999).
3. Trost, B. M., Huang, Z. X. & Murhade, G. M. Catalytic palladium-oxyallyl cycloaddition. *Science*. **362**, 564–568 (2018).
4. Beruben, D., Marek, I., Normant, J. F. & Platzer, N. Stereodefined Substituted Cyclopropyl Zinc Reagents from Gem-Bismetallics. *J. Org. Chem.* **60**, 2488–2501 (1995).
5. Abas, H., Frampton, C. S. & Spivey, A. C. Diels–Alder Reactions of α-Amido Acrylates with N-Cbz-1,2-dihydropyridine and Cyclopentadiene. *J. Org. Chem.* **81**, 9947–9956 (2016).
